# Synthesis of Functionalized Isoquinolone Derivatives via Rh(III)-Catalyzed [4+2]-Annulation of Benzamides with Internal Acetylene-Containing α-CF_3_-α-Amino Carboxylates

**DOI:** 10.3390/molecules27238488

**Published:** 2022-12-02

**Authors:** Daria V. Vorobyeva, Dmitry A. Petropavlovskikh, Ivan A. Godovikov, Fedor M. Dolgushin, Sergey N. Osipov

**Affiliations:** 1 A. N. Nesmeyanov Institute of Organoelement Compounds, Russian Academy of Sciences, 28/1 Vavilova Str., 119334 Moscow, Russia; 2 N. S. Kurnakov Institute of General and Inorganic Chemistry, Russian Academy of Sciences, Leninsky pr. 31, 119071 Moscow, Russia; 3 Plekhanov Russian University of Economics, 36, Stremyanny Per., 117997 Moscow, Russia

**Keywords:** acetylenes, amino acids, C-H activation, annulation, catalysis, isoquinolones

## Abstract

A convenient pathway to a new series of α-CF_3_-substituted α-amino acid derivatives bearing pharmacophore isoquinolone core in their backbone has been developed. The method is based on [4+2]-annulation of *N-*(pivaloyloxy) aryl amides with orthogonally protected internal acetylene-containing α-amino carboxylates under Rh(III)-catalysis. The target annulation products can be easily transformed into valuable isoquinoline derivatives via a successive aromatization/cross-coupling operation.

## 1. Introduction

**Figure 1:**
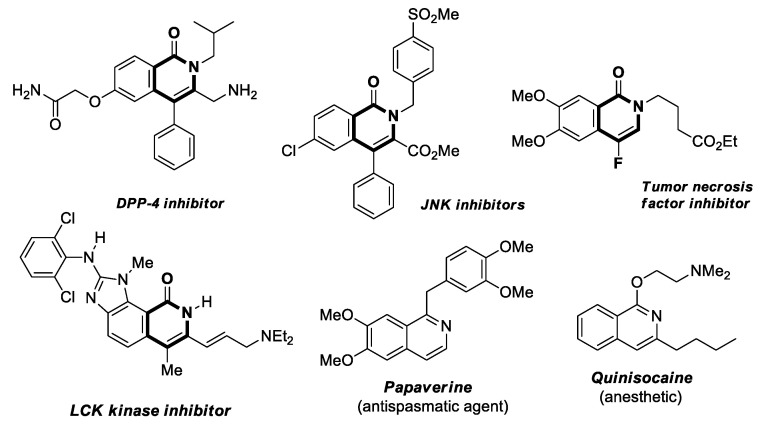
Selected bioactive isoquinolones and isoquinolines.

Isoquinolin-1(2*H*)-ones and analogues are important nitrogen heterocycles possessing diverse bioactivities and presented in many candidates, including antitumor, anti-diabetic, anti-inflammatory and cardiovascular drugs [1,2,3,4,5,6,7] (Figure 1). They are also widely used as key intermediates in variety of organic transformations to access more potent bioactive molecules [8,9,10,11,12,13,14].

Transition-metal-catalyzed annulation reactions via C-H activation have gained great importance as a powerful step- and atom-economical strategy for the preparation of complex molecules from simple starting materials [15,16,17,18]. In particular, [Cp*Rh^III^]-catalyzed annulation of the akynes with (hetero)arenes bearing different directing groups has become one of the most efficient and robust synthetic methodologies for the construction of various functionalized heterocycles [19,20,21,22,23,24,25]. In 2010, Fagnou and co-workers discovered that the *N*-pivaloyloxy group can act both as a directing group and as an internal oxidant through N-O bond cleavage during the synthesis of isoquinolones [26,27]. Mechanistically, this redox-neutral [4+2]-annulation process between alkynes and *N*-pivaloyloxy aryl amides has shown to proceed via the formation of a seven-membered rhodacycle intermediate, which undergoes a C-N bond forming/N-O bond cleaving event to yield the free NH isoquinolones suitable for further useful transformations [28,29,30,31,32,33,34]. Despite significant advances made in the field, the development of effective strategies employing new coupling partners to construct functionally substituted quinolin-2(*1H*)-ones remains of great demand.

On the other hand, modern peptide-based drug design very often aims on selective derivatizing of the peptide backbone through the introduction of additional functional groups or proven heterocyclic pharmacophores in order to modulate the required properties [35,36,37,38,39,40,41]. In this respect, α-fluoroalkyl-containing α-amino acids are of particular interest since they find widespread bio-organic applications as biological tracers, mechanistic probes, and enzyme inhibitors as well as medical applications including blood pressure control, allergies, and tumor growth [42,43,44,45,46]. The incorporation of fluorinated α-amino acids into key positions of bioactive peptides is one of the most common strategies to improve their pharmacokinetic profiles, conformational and proteolytic stability, and membrane permeability [43,44,45,46,47,48,49,50]. Therefore, the development of new representatives of α-fluoromethyl-α-amino acids, including those decorated with pharmacophore heterocycle rings, is of high interest.

**Scheme 1:**
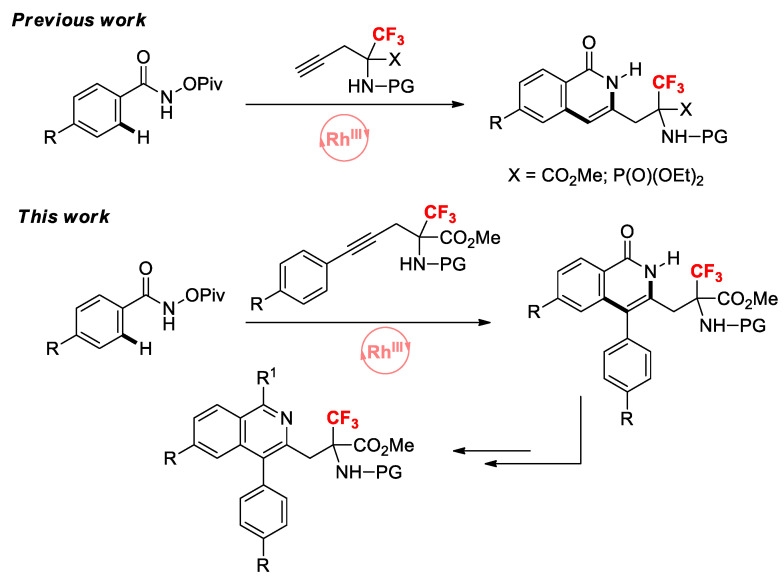
Previous and present work.

Recently, we have elaborated an efficient strategy for the preparation of novel α-CF_3_-α-amino acid derivatives and their phosphorus counterparts with isoquinolone moiety in their backbone based on [Cp*Rh^III^]-catalyzed [4+2]-annulation of terminal propargyl-containing α-CF_3_-substituted α-amino carboxylates and α-propargyl-α-amino phosphonates with *N*-pivaloyloxy aryl amides [51] (Scheme 1).

In continuation of our current research on metal-catalyzed C-H bond activation [52,53,54,55], here we want to disclose a convenient regio-selective approach to new isoquinolone-containing α-amino acid derivatives derived from internal aryl acetylenes bearing protected α-CF_3_-α-amino carboxylate framework under rhodium(III)-catalysis, and their further synthetic transformations into highly functionalized isoquinolines (Scheme 1).

## 2. Results and Discussion

We commenced our study by examining a model reaction between phenyl hydroxamate **1a** and the readily available internal phenyl acetylene **2a** [56] bearing an *N*-Boc-protected α-CF_3_-α-amino ester group for the screening of optimal conditions for the annulation. The rhodium catalytic system [Cp*RhCl_2_]_2_/CsOAc, as the most competent catalyst for such type of transformation, was initially tested to activate the process. As a result, the reaction was found to smoothly proceed in the presence of 5 mol% [Cp*RhCl_2_]_2_ and 2.0 equiv. of cesium acetate in methanol at room temperature for 4 h to give the corresponding isoquinolone derivative **3a** in 70% NMR yield (Table 1, entry 1), along with noticeable amounts of starting materials. Encouraged by this result, we screened some solvents and bases for the reaction (entries 2–5). The best conversion of the starting materials and isolated yield of **3a** (measured by ^19^F NMR spectroscopy) were achieved by the usage of 2,2,2-trifluoroethanol (TFE) and CsOAc (entry 2). Subsequent reduction of catalyst and additive loading (entries 2–6) has revealed the optimal reaction conditions: [Cp*RhCl_2_]_2_ (3 mol%) and CsOAc (1 equiv.), r.t., 2 h in MeOH (entry 8). Iridium-, cobalt- and ruthenium-based complexes have proved to be absolutely inactive in the process (entries 10–12). The reaction does not take place in the absence of any catalyst or base, expectedly (entries 13, 14).

**Scheme 2:**
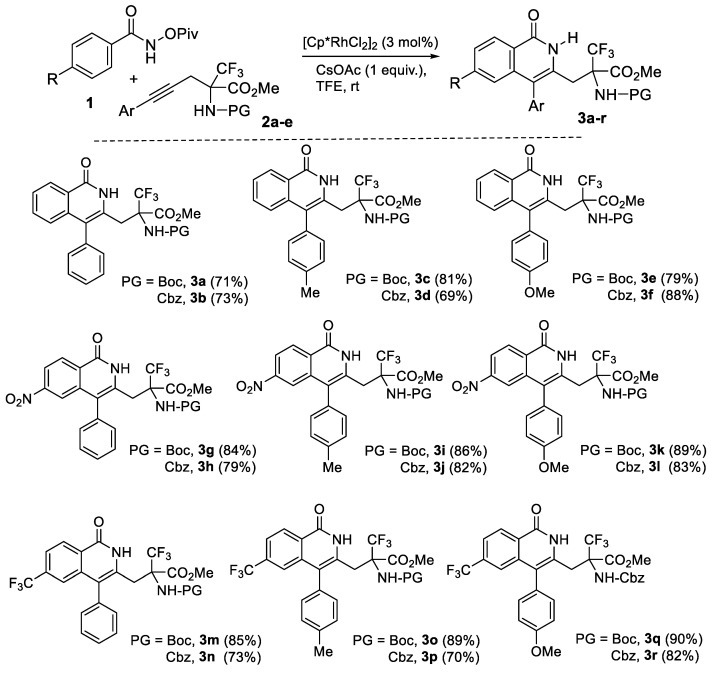
Synthesis of isoquinolone-containing α-amino carboxylates **3**.

With these found conditions in hand, different aryl hydroxamates were involved in [4+2]-annulation with different internal acetylene-containing amino ester **2a–e** (Scheme 2). The latter were easily synthesized via an addition of Grignard reagent (CH_2_=C=CH-MgBr), generated from propargyl bromide, to orthogonally protected α-CF_3_-α-imino carboxylates followed by Sonogashira coupling with aryl halogenides according to the previously described protocol [56]. As a result, a series of the corresponding isoquinolinone-containing α-CF_3_-amino carboxylates **3a–r** were obtained in good yields and high degree of regio-selectivity. The observed selectivity of the [4+2]-annulation process of **1** with α-(arylpropargyl)-α-amino esters **2a–e** bearing donor substituents in aryl group could be probably explained by alkyne insertion into initially formed 5-membered rhodacycle intermediate [26,27], according to its inherent polarity (for proposed mechanism see Supplementary Materials, Scheme S1). The nature of the substituents in hydroxamate component did not significantly affect the outcome of the reaction in all investigated cases (Scheme 2).

**Scheme 3:**
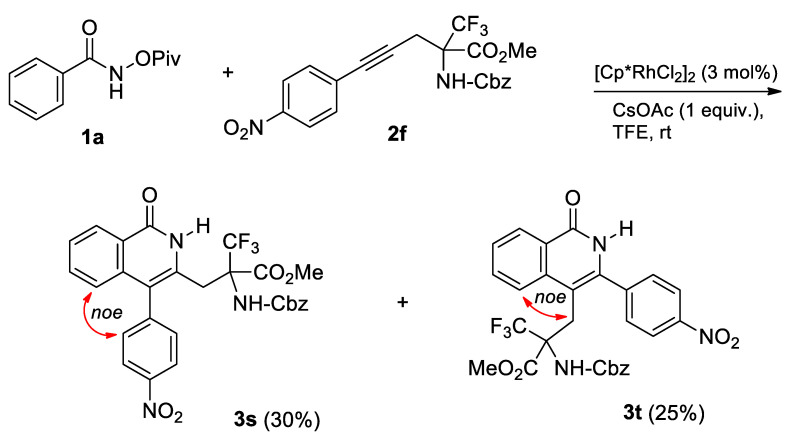
Reaction of **1a** with para-nitrophenyl acetylene **2f**.

However, the presence of an electron-withdrawing nitro group in *para*-position of aryl substituent of acetylene component leads to a mixture of the corresponding regioisomers **3s** and **3t** in a ratio of 3:2 respectively, which were easily separated by column chromatography on silica gel. The absence of selectivity in this case can be likely addressed to a change in the polarity of the triple bond due to the influence of strong acceptor group. The structure of each regio-isomer was assigned by 2D NOESY experiments (see Supplementary Materials). Thus, an intensive cross peak between proton of CH_2_ group of amino acid residue and ortho-proton of phenyl ring was found in the spectrum of isomer **3t** that has not been observed for compound **3s**; instead, a cross peak between the ortho-protons of close located phenyl moieties appeared (Scheme 3).

**Figure 2:**
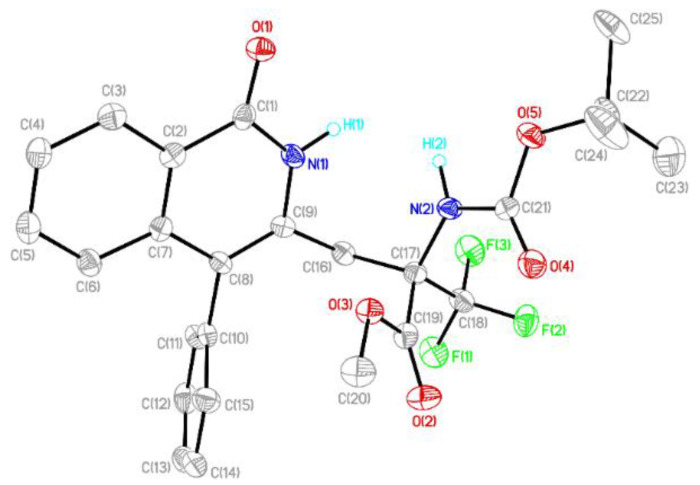
ORTEP representation of the molecular structure of **3a** (CCDC 2217085). Thermal ellipsoids are drawn at the 30% probability level).

All synthesized compounds were fully characterized by physicochemical methods. In addition, the structure of isoquinolone **3a** was confirmed by X-ray crystallographic analysis (Figure 2).

**Scheme 4:**
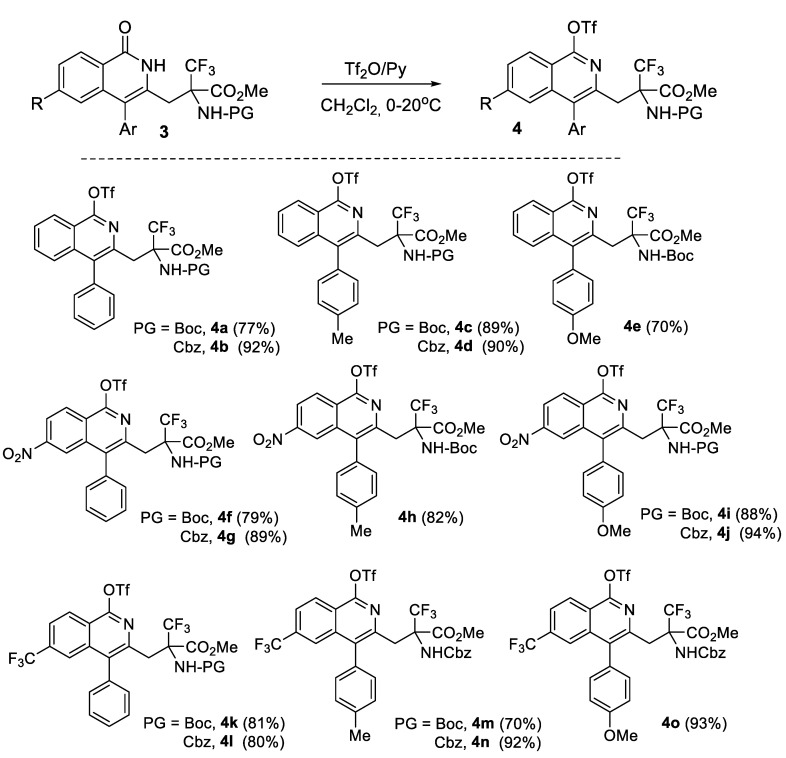
Synthesis of 1-OTf-substituted isoquinolines **4**.

Considering the fact that isoquinolines are key structural elements of many bioactive compounds including drugs [57,58,59] (see Figure 1), we were interested in further investigation of synthetic potential of obtained isoquinolones as universal precursors of the corresponding isoquinoline derivatives decorated with amino acid residues. Thus, we found that the isoquinolones **3** could easily undergo an aromatization into the isoquinolines under treatment with triflic anhydride in the presence of pyridine. The reactions proceed in CH_2_Cl_2_ under mild conditions and go to completion within 15 min at ambient temperature furnishing the corresponding 1-OTf-substituted isoquinolines **4a–o** in good to high yields (Scheme 4).

**Scheme 5:**
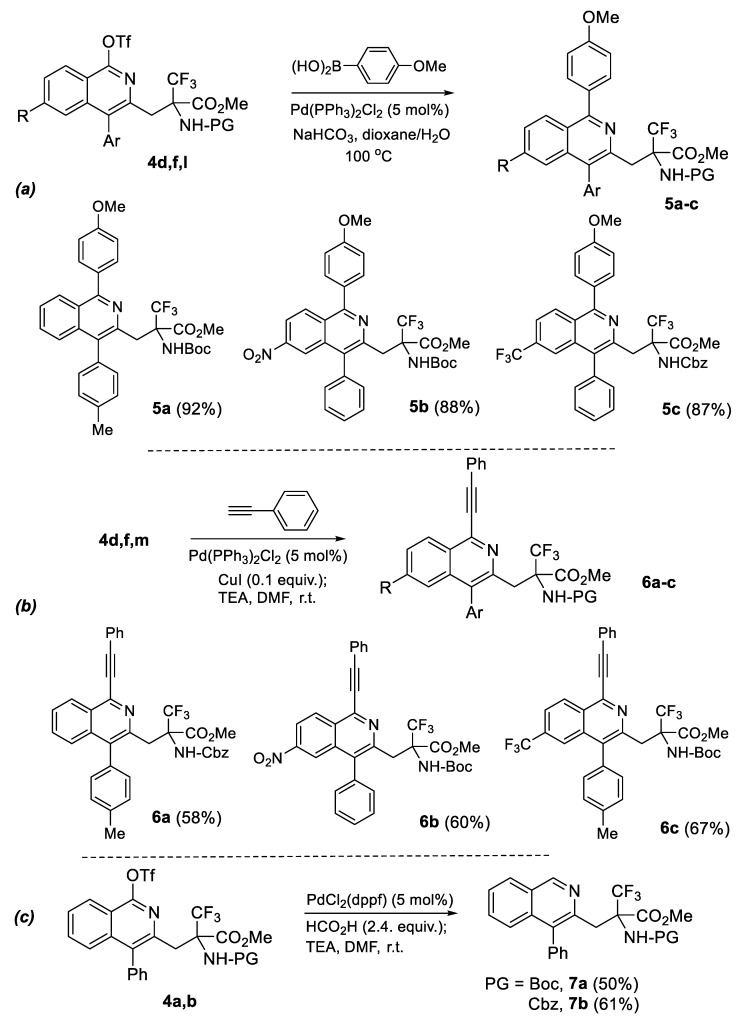
Transformations of the OTf-substituted isoquinolines **4** under Pd-catalysis: (**a**) Suzuki reaction; (**b**) Sonogashira reaction; (**c**) removal of OTf group.

The well-known pseudo-halogen nature of the TfO group pushed us to explore some further useful transformations of the synthesized isoquinoline derivatives **4**, such as Pd-catalysed cross-coupling reactions. As a result, it turned out that the compounds **4** could serve as suitable cross-coupling partners in Suzuki reaction with various aryl boronic acids. It was revealed that the reactions of **4d**,**f**,**l** with 4-metoxy phenyl boronic acid readily proceeded in dioxane-water mixture under catalysis with Pd(PPh_3_)_2_Cl_2_/NaHCO_3_ system at 100 °C, leading to the formation of the expected cross-coupling products **5a–c** in high yields (Scheme 5a).

The same isoquinoline-containing α-amino acid derivatives **4d,f,m** were included in coupling with phenyl acetylene under the classical Sonogashira reaction conditions to afford the corresponding products **6a–c** in good yields (Scheme 5b). Finally, we found that the OTf group could be successfully removed in the presence of catalytic amounts of PdCl_2_(dppf) complex and excess of formic acid to give isoquinolines **7a,b** in acceptable yields (Scheme 5c).

**Scheme 6:**
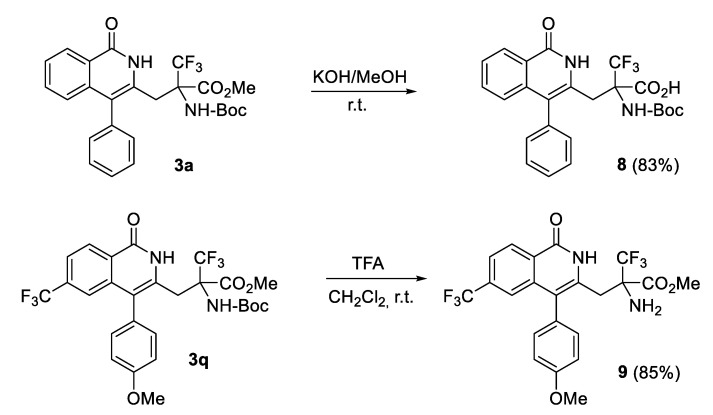
Removal of protective groups.

In addition, in order to demonstrate a feasibility for the further synthetic applications of the compounds obtained, e.g., in peptide synthesis or other useful derivatizations, we have performed selective deprotections of the *N*-PG-α-amino esters. Thus, the ester **3a** was saponified using 5% solution of potassium hydroxide in methanol to get free carboxylic acid **8** in high yield. The Boc-protective group of compound **3q** was selectively removed by the treatment of its solution in methylene chloride with excess of trifluoroacetic acid at room temperature furnishing free amino ester **9** in 85% yield (Scheme 6).

## 3. Materials and Methods

### 3.1. General Information

All solvents used in the reactions were freshly distilled from appropriate drying agents before use. All other reagents were distilled as necessary. The corresponding starting acetylenes were easily synthesized via the previously described protocol [56]. Analytical TLC was performed with Merck silica gel 60 F_254_ plates; visualization was accomplished with UV light or spraying with Ce(SO_4_)_2_ solution in 5% H_2_SO_4_. Chromatography was carried out using Merck silica gel (Kieselgel 60, 0.063–0.200 mm) and petroleum ether/ethyl acetate as an eluent. The NMR spectra were obtained with Bruker AV-300, AV-400, AV-500 and Inova-400 spectrometers operating at 300, 400, and 500 MHz, respectively, for ^1^H (TMS reference), at 101 and 126 for ^13^C {^1^H}, and at 282 and 376 MHz for ^19^F (CCl_3_F reference). Analytical data (C, H, N content) were obtained with a Carlo Erba model 1106 microanalyzer.

#### 3.1.1. General Procedure for C-H Activation/Annulation of Aryl Hydroxamate with Acetylenes. Synthesis of the Compounds **3a–r**

A dried 10 mL Shlenk tube equipped with a magnetic stirring bar was subsequently charged with a corresponding acetylene (0.1 g, 0.27 mmol, 1.0 equiv.), TFE (2 mL), corresponding aryl hydroxamate (0.06 g, 0.27 mmol, 1.0 equiv.), [Cp^*^RhCl_2_]_2_ (4.9 mg, 8.0 µmol, 3 mol%) and CsOAc (0.05 g, 0.27 mmol, 1.0 equiv.) under Ar. The reaction mixture was stirred at room temperature for 4 h until the completion of the reaction, monitored by TLC and ^19^F NMR. By this time, the product precipitate had been formed and then was isolated from the reaction mixture by filtration.

#### 3.1.2. General Procedure for the Synthesis of TfO-Derivatives **4a–o**

To a solution of the corresponding isoquinolone (0.1 g, 0.2 mmol, 1 equiv.) in dry dichloromethane (15 mL), pyridine (1.5 equiv.) and Tf_2_O (1.5 equiv.) were added at 0 °C. After having been stirred at 0 °C for 30 min, the reaction mixture was treated with water and extracted with dichloromethane. The organic layer was washed with saturated NaHCO_3_ (aq.), dried over anhydrous MgSO_4_, filtered, and evaporated to dryness. The residue was purified by column chromatography on silica gel (eluent petroleum ether/ethyl acetate = 15/1) to give the desired product.

#### 3.1.3. General Procedure for the Suzuki Reaction. Synthesis of the Compounds **5a–c**

A 25 mL round-bottom flask equipped with a magnetic stir bar and a reflux condenser was charged with corresponding triflate-derivative (0.1 g, 0.16 mmol, 1 equiv.), corresponding boronic acid (0.02 g, 0.16 mmol, 1 equiv.), 1,4-dioxane—water mixture (3:1, 4 mL), NaHCO_3_ (0.04 g, 0.5 mmol, 3 equiv.), and Pd(PPh_3_)_2_Cl_2_ (1.1 mg, 1.6 µmol, 1 mol %). The resulting mixture was deaerated with argon and refluxed at 100 °C under argon for 1 h until the completion (monitored by TLC). On completion, the mixture was poured into water (7 mL) and extracted with dichloromethane (3 × 5 mL). The combined organic phases were washed with brine, dried over anhydrous MgSO_4_, filtered, and and evaporated to dryness. The crude product was purified by column chromatography (eluent petroleum ether/ethyl acetate = 10/1) to give the desired product.

#### 3.1.4. General Procedure for the Sonogashira Reaction. Synthesis of the Compounds **6a–c**

A dried 10 mL Shlenk tube was charged with a magnetic stir bar, DMF (5.5 mL) and corresponding triflate derivative (0.1 g, 0.15 mmol, 1.0 equiv.), and the solution was degassed three times. Then, (Ph_3_P)_2_PdCl_2_ (5.2 mg, 7.5 µmol, 5 mol%) was added and the degassing procedure repeated. After that, Et_3_N (0.28 mL) and phenyl acetylene (0.02 g, 0.22 mmol, 1.5 equiv.) was added and the mixture was degassed. Then, copper iodide (2.8 mg, 0.01 mmol, 10 mol%) was added and the reaction was stirred at room temperature overnight. After the completion (monitored by TLC) the reaction mixture was poured into 1M HCl (20 mL) and extracted with ethyl acetate (3 × 10 mL). The organic layers were dried over anhydrous MgSO_4_, filtered and evaporated to dryness. The crude product was purified by column chromatography (eluent petroleum ether/ethyl acetate = 10/1) to give the desired product.

#### 3.1.5. General Procedure for the Reduction of Triflate-Group. Synthesis of the Compounds **7a,b**

To a dried 10 mL Shlenk tube equipped with a magnetic stir bar corresponding triflate (0.1 g, 0.16 mmol, 1.0 equiv.), (dppf)PdCl_2_ (5.8 mg, 8.0 µmol, 5 mol%), TEA (0.05 mL, 0.48 mmol, 3.0 equiv.), DMF (2.5 mL) and HCOOH (0.01 mL, 0.38 mmol, 2.4 equiv.) were added. The mixture was stirred at room temperature for 3 h. After the completion of the reaction (monitored by TLC), the solution was poured into 1M HCl (10 mL) and extracted with Et_2_O (3 × 10 mL). The organic layers were washed with water and dried over anhydrous MgSO_4_, filtered and evaporated to dryness. The crude product was purified by column chromatography (eluent petroleum ether/ethyl acetate = 8/1) to give the desired product.

#### 3.1.6. General Procedure for Ester Hydrolysis. Synthesis of the Compound **8**

The corresponding isoquinolone 3a (0.3 g, 0.6 mmol) was dissolved in 5% KOH/MeOH-H_2_O (1:1) (13 mL) and stirred at room temperature for 2 h. After evaporation of solvents under reduced pressure, water (15 mL) was added to a residue, and the suspension was washed with diethyl ester (3 × 10 mL) before being acidified with HCl conc. until pH 3–4 and extracted with ethyl acetate (3 × 7 mL). The ethyl acetate extracts were combined and dried over anhydrous MgSO_4_, filtered, and evaporated to dryness.

#### 3.1.7. General Procedure for the Removing of the Boc-Protecting Group. Synthesis of the Compound **9**

A solution of Boc-protected isoquinolone 3q (0.25 g, 0.42 mmol) in a biphasic mixture of trifluoroacetic acid/dichlormethane (4 mL/10 mL) was stirred at room temperature for 1.5 h. After evaporation of solvents under reduced pressure, water (15 mL) was added to the residue and the resulting water solution was neutralized with saturated solution of sodium bicarbonate until pH 7. Then the mixture was extracted with diethyl ether (3 × 10 mL). The organic layer was dried over anhydrous MgSO_4_ and evaporated to dryness.

### 3.2. Characterization Data for the Products

*Methyl 2-(tert-butoxycarbonylamino)-3,3,3-trifluoro-2-((1-oxo-4-phenyl-1,2-dihydroiso quinolin-3-yl)methyl)propanoate* (**3a**).

Yield 71% as a white solid. M.p. 213–215 °C. ^1^H NMR (400 MHz, DMSO-*d*_6_): *δ* 10.87 (s, 1H, NH), 8.26 (d, *J* = 7.9 Hz, 1H, Ar), 8.15 (s, 1H, NH), 7.62 (t, *J* = 7.6 Hz, 1H, Ar), 7.54–7.43 (m, 4H, Ar), 7.26 (d, *J* = 7.3 Hz, 1H, Ar), 7.10 (d, *J* = 7.5 Hz, 1H, Ar), 6.97 (d, *J* = 8.2 Hz, 1H, Ar), 3.48 (s, 3H, OCH_3_), 3.18 (d, *J* = 14.8 Hz, 1H, CH_2_), 2.91 (d, *J* = 14.7 Hz, 1H, CH_2_), 1.37 (s, 9H 3 CH_3_). ^13^C{^1^H} NMR (126 MHz, DMSO-*d_6_*): *δ* 164.8, 161.9, 159.2, 138.3 and 137.1, 137.1, 135.3 and 135.0, 133.5 and 133.1, 132.2 and 132.1, 131.3, 130.6 and 130.5, 129.8 and 129.5, 128.9 and 128.8, 128.4 and 128.2, 127.1 and 127.0, 125.6 and 125.5, 125.3 and 125.2, 124.6 (q, *J* = 285.6 Hz, CF_3_), 118.9, 115.8, 81.2 and 80.7, 62.7 (q, *J* = 29.1 Hz, >C<), 53.0, 31.8 and 31.5, 28.3. ^19^F NMR (376 MHz, DMSO-*d_6_*): *δ −*74.13 (s, 3F, CF_3_). Elemental analysis calcd (%) for C_25_H_25_F_3_N_2_O_5_: C, 61.22; H, 5.14; N, 5.71; found: C, 61.18; H, 5.01; N, 5.65.

*Methyl 2-(benzyloxycarbonylamino)-3,3,3-trifluoro-2-((1-oxo-4-phenyl-1,2-dihydroiso quinolin-3-yl)methyl)propanoate* (**3b**).

Yield 73% as a white solid. M.p. 168–170 °C. ^1^H NMR (400 MHz, acetone-*d_6_*): *δ* 10.45 (s, 1H, NH), 8.37 (d, *J* = 7.9 Hz, 1H, Ar), 7.65–7.47 (m, 6H, Ar), 7.29–7.21 (m, 6H, Ar, 1H, NH), 7.04 (d, *J* = 8.1 Hz, 1H, Ar), 4.96 (s, 2H, OCH_2_), 3.67 (s, 3H, OCH_3_), 3.47 (d, *J* = 15.2 Hz, 1H, CH_2_), 3.33 (d, *J* = 15.3 Hz, 1H, CH_2_). ^13^C{^1^H} NMR (126 MHz, DMSO-*d_6_*): *δ* 164.0, 161.4, 154.4, 137.8, 136.0, 134.5, 132.6, 131.6, 131.5, 130.8, 129.0, 128.5, 128.4, 128.2, 128.0, 127.9, 126.7, 126.6, 126.3 (q, *J* = 289.5 Hz, CF_3_), 125.2, 124.8, 118.6, 66.5, 64.0 (q, *J* = 27.0 Hz, >C<), 52.7. ^19^F NMR (376 MHz, acetone-*d_6_*): *δ −*75.08 (s, 3F, CF_3_). Elemental analysis calcd (%) for C_28_H_23_F_3_N_2_O_5_: C, 64.12; H, 4.42; N, 5.34; found: C, 64.44; H, 4.66; N, 5.71.

*Methyl 2-(tert-butoxycarbonylamino)-3,3,3-trifluoro-2-((1-oxo-4-p-tolyl-1,2-dihydroiso quinolin-3-yl)methyl)propanoate* (**3c**).

Yield 81% as a white solid. M.p. 224–226 °C. ^1^H NMR (500 MHz, DMSO-*d*_6_): *δ* 10.83 (s, 1H, NH), 8.25 (d, *J* = 7.9 Hz, 1H, Ar), 8.13 (s, 1H, NH), 7.61–7.59 (m, 1H, Ar), 7.52–7.49 (m, 1H, Ar), 7.32 (d, *J* = 7.8 Hz, 1H, Ar), 7.29 (d, *J* = 7.8 Hz, 1H, Ar), 7.13 (d, *J* = 7.7 Hz, 1H, Ar), 6.99–6.96 (m, 2H, Ar), 3.48 (s, 3H, OCH_3_), 3.20 (d, *J* = 15.0 Hz, 1H, CH_2_), 2.92 (d, *J* = 15.0 Hz, 1H, CH_2_), 2.38 (s, 3H, CH_3_), 1.37 (s, 9H 3 CH_3_). ^13^C{^1^H} NMR (126 MHz, DMSO-*d_6_*): *δ* 164.4, 161.4, 153.9, 138.1, 137.1, 132.6, 131.6, 131.5, 131.4, 130.7, 129.6, 129.0, 126.6, 126.5, 125.2, 124.8, 124.1 (q, *J* = 288.0 Hz, CF_3_), 118.4, 80.2, 63.9 (q, *J* = 23.3 Hz, >C<), 52.5, 31.1, 27.8, 20.9. ^19^F NMR (376 MHz, CDCl_3_): *δ −*75.21 (s, 3F, CF_3_). Elemental analysis calcd (%) for C_26_H_27_F_3_N_2_O_5_: C, 61.90; H, 5.39; N, 5.55; found: C, 62.04; H, 5.55; N, 5.63.

*Methyl 2-(benzyloxycarbonylamino)-3,3,3-trifluoro-2-((1-oxo-4-p-tolyl-1,2-dihydroiso quinolin-3-yl)methyl)propanoate* (**3d**).

Yield 69% as a white solid. M.p. 184–185 °C. ^1^H NMR (500 MHz, CDCl_3_): *δ* 12.45 (s, 1H, NH), 8.46 (d, *J* = 7.9 Hz, 1H, Ar), 7.57 (t, *J* = 7.7 Hz, 1H, Ar), 7.47 (t, *J* = 7.2 Hz, 2H, Ar), 7.32–7.28 (m, 2H, Ar), 7.15 (d, *J* = 8.0 Hz, 2H, Ar), 7.08–7.05 (m, 3H, Ar), 7.01 (d, *J* = 7.7 Hz, 1H, Ar), 6.93 (s, 1H, Ar, 1H, NH), 4.82 (s, 2H, OCH_2_), 3.59 (s, 3H, OCH_3_), 3.47 (d, *J* = 15.1 Hz, 1H, CH_2_), 3.28 (d, *J* = 15.2 Hz, 1H, CH_2_), 2.45 (s, 3H, CH_3_). ^13^C{^1^H} NMR (126 MHz, CDCl_3_): *δ* 165.0, 164.0, 155.1, 138.9, 138.2, 135.8, 133.0, 131.6, 131.4, 130.9, 130.8, 129.9, 129.6, 128.3, 127.9, 127.6, 127.4, 127.1, 126.0, 124.4, 124.1 (q, *J* = 287.5 Hz, CF_3_), 121.9, 67.1, 64.9 (q, *J* = 27.6 Hz, >C<), 53.3, 31.9, 21.5. ^19^F NMR (282 MHz, CDCl_3_): *δ −*75.48 (s, 3F, CF_3_). Elemental analysis calcd (%) for C_29_H_25_F_3_N_2_O_5_: C, 64.68; H, 4.68; N, 5.20; found: C, 64.79; H, 4.85; N, 5.51.

*Methyl 2-(tert-butoxycarbonylamino)-3,3,3-trifluoro-2-((4-(4-methoxyphenyl)-1-oxo-1,2-dihydroisoquinolin-3-yl)methyl)propanoate* (**3e**).

Yield 79%. M.p. 220–221 °C. ^1^H NMR (500 MHz, DMSO-*d*_6_): *δ* 10.84 (s, 1H, NH), 8.25 (d, *J* = 7.9 Hz, 1H, Ar), 8.13 (s, 1H, NH), 7.61 (t, *J* = 7.6 Hz, 1H, Ar), 7.50 (t, *J* = 7.5 Hz, 1H, Ar), 7.17 (d, *J* = 7.8 Hz, 1H, Ar), 7.09–6.99 (m, 4H, Ar), 3.82 (s, 3H, OCH_3_), 3.49 (s, 3H, OCH_3_), 3.22 (d, *J* = 14.9 Hz, 1H, CH_2_), 2.91 (d, *J* = 15.0 Hz, 1H, CH_2_), 1.37 (s, 9H, 3 CH_3_). ^13^C{^1^H} NMR (126 MHz, DMSO-*d*_6_): *δ* 164.4, 161.4, 158.7, 153.9, 138.3, 132.8, 132.5, 132.0, 131.8, 126.6, 126.5, 126.4, 125.2, 124.8, 124.1 (q, *J* = 286.9 Hz, CF_3_) 118.2, 114.4, 113.9, 80.2, 63.9 (q, *J* = 23.9 Hz, >C<), 55.1, 52.5, 31.1, 27.8. ^19^F NMR (376 MHz, CDCl_3_): *δ* −75.07 (s, 3F, CF_3_). Elemental analysis calcd (%) for C_26_H_27_F_3_N_2_O_6_: C, 60.00; H, 5.23; N, 5.38; found: C, 60.27; H, 5.55; N, 5.53.

*Methyl 2-(benzyloxycarbonylamino)-3,3,3-trifluoro-2-((4-(4-methoxyphenyl)-1-oxo-1,2-dihydroisoquinolin-3-yl)methyl)propanoate* (**3f**).

Yield 88% as a white solid. M.p. 200–201 °C. ^1^H NMR (500 MHz, DMSO-*d_6_*): *δ* 10.83 (s, 1H, NH), 8.61 (s, 1H, NH), 8.25 (d, *J* = 8.0 Hz, 1H, Ar), 7.61 (t, *J* = 7.6 Hz, 1H, Ar), 7.51 (t, *J* = 7.5 Hz, 1H, Ar), 7.37 (s, 5H, Ar), 7.17 (d, *J* = 8.4 Hz, 1H, Ar), 7.07 (d, *J* = 7.1 Hz, 1H, Ar), 7.04–6.99 (m, 3H, Ar), 5.09 (d, *J* = 12.2 Hz, 1H, OCH_2_), 5.02 (d, *J* = 12.2 Hz, 1H, OCH_2_), 3.82 (s, 3H, OCH_3_), 3.49 (s, 3H, OCH_3_), 3.25 (d, *J* = 15.0 Hz, 1H, CH_2_), 2.95 (d, *J* = 14.9 Hz, 1H, CH_2_). ^13^C{^1^H} NMR (126 MHz, DMSO-*d_6_*): *δ* 164.1, 161.4, 158.7, 154.5, 138.2, 136.0, 132.7, 132.6, 132.0, 131.6, 128.5, 128.2, 128.0, 126.7, 126.5, 126.4, 125.2, 125.0 (q, *J* = 290.1 Hz, CF_3_), 124.8, 118.3, 114.3, 113.9, 66.5, 64.0 (q, *J* = 25.8 Hz, >C<), 55.1, 52.7, 31.2. ^19^F NMR (376 MHz, DMSO-*d_6_*): *δ −*73.99 (s, 3F, CF_3_). Elemental analysis calcd (%) for C_29_H_25_F_3_N_2_O_6_: C, 62.81; H, 4.54; N, 5.05; found: C, 63.07; H, 4.78; N, 5.28.

*Methyl 2-(tert-butoxycarbonylamino)-3,3,3-trifluoro-2-((6-nitro-1-oxo-4-phenyl-1,2-dihydroisoquinolin-3-yl)methyl)propanoate* (**3g**).

Yield 84% as a white solid. M.p. 173–175 °C. ^1^H NMR (400 MHz, DMSO-*d_6_*): *δ* 11.33 (s, 1H, NH), 8.49 (d, *J* = 8.8 Hz, 1H, Ar), 8.24 (d, *J* = 9.0 Hz, 1H, Ar), 8.17 (br. s, 1H, NH), 7.75 (s, 1H, Ar), 7.60–7.50 (m, 3H, Ar), 7.33 (d, *J* = 7.1 Hz, 1H, Ar), 7.17 (d, *J* = 7.4 Hz, 1H, Ar), 3.50 (s, 3H, OCH_3_), 3.21 (d, *J* = 14.9 Hz, 1H, CH_2_), 2.96 (d, *J* = 14.9 Hz, 1H, CH_2_), 1.37 (s, 9H, 3 CH_3_). ^13^C{^1^H} NMR (126 MHz, DMSO-*d_6_*): *δ* 164.4, 160.6, 153.9, 149.9, 138.5, 133.5, 131.6, 131.0, 130.2, 129.7, 129.4, 129.3, 128.8, 128.6, 128.3, 124.1 (q, *J* = 288.3 Hz, CF_3_), 121.4, 120.2, 118.3, 80.4, 63.8 (q, *J* = 26.9 Hz, >C<), 52.7, 27.9. ^19^F NMR (376 MHz, DMSO-*d_6_*): *δ −*74.30 (s, 3F, CF_3_). Elemental analysis calcd (%) for C_25_H_24_F_3_N_3_O_7_: C, 56.08; H, 4.52; N, 7.85; found: C, 56.04; H, 4.55; N, 7.81.

*Methyl 2-(benzyloxycarbonylamino)-3,3,3-trifluoro-2-((6-nitro-1-oxo-4-phenyl-1,2-dihydroisoquinolin-3-yl)methyl)propanoate* (**3h**).

Yield 79% as a yellow solid. M.p. 206–208 °C. ^1^H NMR (300 MHz, DMSO-*d_6_*): *δ* 11.30 (s, 1H, NH), 8.63 (s, 1H, NH), 8.49 (d, *J* = 8.8 Hz, 1H, Ar), 8.25 (d, *J* = 8.8 Hz, 1H, Ar), 7.74 (s, 1H, Ar), 7.61–7.53 (m, 3H, Ar), 7.37 (s, 6H, Ar), 7.17 (d, *J* = 7.4 Hz, 1H, Ar), 5.08 (d, *J* = 12.2 Hz, 1H, OCH_2_), 5.01 (d, *J* = 12.2 Hz, 1H, OCH_2_), 3.50 (s, 3H, OCH_3_), 3.20 (d, *J* = 15.9 Hz, 1H, CH_2_), 3.00 (d, *J* = 15.2 Hz, 1H, CH_2_). ^13^C{^1^H} NMR (126 MHz, DMSO-*d_6_*): *δ* 164.4, 160.6, 153.9, 149.9, 138.5, 133.5, 131.6, 131.0, 130.2, 129.7, 129.4, 129.3, 129.1, 129.0, 128.8, 128.6, 128.3, 124.1 (q, *J* = 288.9 Hz, CF_3_), 120.2, 118.3, 114.8, 80.4, 63.8 (q, *J* = 23.6 Hz, >C<), 52.7, 31.3, 27.9. ^19^F NMR (282 MHz, DMSO-*d_6_*): *δ −*74.17 (s, 3F, CF_3_). Elemental analysis calcd (%) for C_28_H_22_F_3_N_3_O_7_: C, 59.05; H, 3.89; N, 7.38; found: C, 59.31; H, 4.08; N, 7.52.

*Methyl 2-(tert-butoxycarbonylamino)-3,3,3-trifluoro-2-((6-nitro-1-oxo-4-p-tolyl-1,2-dihydroisoquinolin-3-yl)methyl)propanoate* (**3i**).

Yield 86% as a white solid. M.p. 211–212 °C. ^1^H NMR (400 MHz, CDCl_3_): *δ* 11.56 (s, 1H, NH), 8.60 (d, *J* = 8.8 Hz, 1H, Ar), 8.22 (d, *J* = 8.8 Hz, 1H, Ar), 7.97 (s, 1H, Ar), 7.36 (d, *J* = 7.7 Hz, 2H, Ar), 7.15 (d, *J* = 7.9 Hz, 1H, Ar), 7.09 (d, *J* = 7.8 Hz, 1H), 6.15 (s, 1H, NH), 3.68 (s, 3H, OCH_3_), 3.49 (d, *J* = 14.9 Hz, 1H, CH_2_), 3.33 (d, *J* = 15.0 Hz, 1H, CH_2_), 2.47 (s, 3H, CH_3_), 1.18 (s, 9H, 3 CH_3_). ^13^C{^1^H} NMR (101 MHz, CDCl_3_): *δ* 165.8, 161.9, 154.3, 150.5, 139.6, 138.9, 133.8, 131.1, 130.7, 130.5, 130.4, 130.2, 129.6, 128.6, 123.7 (q, *J* = 288.3 Hz, CF_3_), 121.3, 120.8, 120.4, 81.8, 64.9 (q, *J* = 28.1 Hz, >C<), 53.8, 31.9, 27.9, 21.4. ^19^F NMR (376 MHz, CDCl_3_) *δ* −74.97 (s, 3F, CF_3_). Elemental analysis calcd (%) for C_26_H_26_F_3_N_3_O_7_: C, 56.83; H, 4.77; N, 7.65; found: C, 56.71; H, 4.57; N, 7.63.

*Methyl 2-(benzyloxycarbonylamino)-3,3,3-trifluoro-2-((6-nitro-1-oxo-4-p-tolyl-1,2-dihydroisoquinolin-3-yl)methyl)propanoate* (**3j**).

Yield 82% as a yellow solid. M.p. 215–216 °C. ^1^H NMR (500 MHz, DMSO-*d_6_*): *δ* 11.26 (s, 1H, NH), 8.61 (s, 1H, NH), 8.48 (d, *J* = 8.8 Hz, 1H, Ar), 8.24 (d, *J* = 8.8 Hz, 1H, Ar), 7.76 (s, 1H, Ar), 7.39 (m, 7H, Ar), 7.19 (d, *J* = 7.8 Hz, 1H, Ar), 7.05 (d, *J* = 7.7 Hz, 1H, Ar), 5.08 (d, *J* = 12.3 Hz, 1H, OCH_2_), 5.02 (d, *J* = 12.3 Hz, 1H, OCH_2_), 3.49 (s, 3H, OCH_3_), 3.27 (d, *J* = 15.3 Hz, 1H, CH_2_), 3.00 (d, *J* = 15.1 Hz, 1H, CH_2_), 2.42 (s, 3H, CH_3_). ^13^C{^1^H} NMR (126 MHz, DMSO-*d_6_*): *δ* 164.1, 160.4, 154.4, 149.9, 138.6, 137.8, 135.9, 134.4, 131.3, 130.8, 130.4, 129.9, 129.3, 129.2, 128.5, 128.3, 128.2, 128.0, 123.9 (q, *J* = 287.4 Hz, CF_3_), 120.3, 120.2, 118.3, 66.5, 64.0 (q, *J* = 26.7 Hz, >C<), 54.9, 52.8, 31.3. ^19^F NMR (282 MHz, CDCl_3_): *δ −*73.84 (s, 3F, CF_3_). Elemental analysis calcd (%) for C_29_H_24_F_3_N_3_O_7_: C, 59.69; H, 4.15; N, 7.20; found: C, 59.93; H, 4.43; N, 7.35.

*Methyl 2-(tert-butoxycarbonylamino)-3,3,3-trifluoro-2-((4-(4-methoxyphenyl)-6-nitro-1-oxo-1,2-dihydroisoquinolin-3-yl)methyl)propanoate* (**3k**).

Yield 89% as a white solid. M.p. 198–200 °C. ^1^H NMR (500 MHz, acetone-*d*_6_): *δ* 10.16 (s, 1H, NH), 8.54 (d, *J* = 8.7 Hz, 1H, Ar), 8.24 (d, *J* = 7.7 Hz, 1H, Ar), 7.92 (s, 1H, Ar), 7.38 (d, *J* = 7.8 Hz, 1H, Ar), 7.28 (d, *J* = 7.9 Hz, 1H, Ar), 7.16 (t, *J* = 6.7 Hz, 2H, Ar), 7.08 (s, 1H, NH), 3.91 (s, 3H, OCH_3_), 3.77 (s, 3H, OCH_3_), 3.50 (d, *J* = 14.9 Hz, 1H, CH_2_), 3.37 (d, *J* = 14.9 Hz, 1H, CH_2_), 1.27 (s, 9H, 3 CH_3_). ^13^C{^1^H} NMR (126 MHz, acetone-*d_6_*): *δ* 167.7, 161.1, 160.9, 155.4, 151.4, 140.8, 139.9, 135.9, 133.7, 133.6, 130.2, 127.0, 124.8 (q, *J* = 287.0 Hz, CF_3_), 121.7, 120.9, 119.7, 115.8, 115.5, 114.8, 81.8, 66.2 (q, *J* = 27.0 Hz, >C<) 55.8, 54.2, 28.3. ^19^F NMR (376 MHz, CDCl_3_): *δ* −75.08 (s, 3F, CF_3_). Elemental analysis calcd (%) for C_26_H_26_F_3_N_3_O_8_: C, 55.22; H, 4.63; N, 7.43; found: C, 55.31; H, 4.57; N, 7.27.

*Methyl 2-(benzyloxycarbonylamino)-3,3,3-trifluoro-2-((4-(4-methoxyphenyl)-6-nitro-1-oxo-1,2-dihydroisoquinolin-3-yl)methyl)propanoate* (**3l**).

Yield 83% as a yellow solid. M.p.207–209 °C. ^1^H NMR (500 MHz, DMSO-*d_6_*): *δ* 11.25 (s, 1H, NH), 8.60 (s, 1H, NH), 8.48 (d, *J* = 8.7 Hz, 1H, Ar), 8.23 (d, *J* = 8.8 Hz, 1H, Ar), 7.78 (s, 1H, Ar), 7.37 (s, 5H, Ar), 7.24 (s, 1H, Ar), 7.14–7.07 (m, 3H, Ar), 5.08 (d, *J* = 12.2 Hz, 1H, OCH_2_), 5.02 (d, *J* = 12.4 Hz, 1H, OCH_2_), 3.84 (s, 3H, OCH_3_), 3.50 (s, 3H, OCH_3_), 3.28 (d, *J* = 15.7 Hz, 1H, CH_2_), 3.00 (d, *J* = 15.1 Hz, 1H, CH_2_). ^13^C{^1^H} NMR (126 MHz, DMSO-*d_6_*): *δ* 164.0, 160.4, 159.1, 154.5, 149.9, 138.9, 136.0, 134.6, 132.7, 132.2, 129.2, 128.5, 128.4, 128.3, 128.1, 125.2, 123.9 (q, *J* = 287.9 Hz, CF_3_), 120.3, 120.2, 118.1, 114.7, 114.2, 66.6, 64.0 (q, *J* = 26.5 Hz, >C<), 55.2, 52.9, 31.3. ^19^F NMR (282 MHz, DMSO-*d_6_*): *δ −*74.07 (s, 3F, CF_3_). Elemental analysis calcd (%) for C_29_H_24_F_3_N_3_O_8_: C, 58.10; H, 4.04; N, 7.01; found: C, 58.49; H, 4.30; N, 7.32.

*Methyl 2-(tert-butoxycarbonylamino)-3,3,3-trifluoro-2-((1-oxo-4-phenyl-6-(trifluoromethyl)-1,2-dihydroisoquinolin-3-yl)methyl)propanoate* (**3m**).

Yield 85% as a white solid. M.p. 181–183 °C. ^1^H NMR (400 MHz, DMSO-*d*_6_): *δ* 11.21 (s, 1H, NH), 8.47 (d, *J* = 8.3 Hz, 1H, Ar), 8.17 (br. s, 1H, NH), 7.84 (d, *J* = 8.4 Hz, 1H, Ar), 7.58–7.47 (m, 3H, Ar), 7.31 (d, *J* = 7.2 Hz, 1H, Ar), 7.21 (s, 1H, Ar), 7.14 (d, *J* = 7.4 Hz, 1H, Ar), 3.50 (s, 3H, OCH_3_), 3.19 (d, *J* = 15.0 Hz, 1H, CH_2_), 2.95 (d, *J* = 15.0 Hz, 1H, CH_2_), 1.37 (s, 9H, 3 CH_3_). ^13^C{^1^H} NMR (126 MHz, DMSO-*d*_6_): *δ* 164.4, 160.7, 153.9, 138.1, 134.0, 133.6, 132.4 (q, *J* = 31.9 Hz, C*_Ar_*-CF_3_), 131.5, 130.9, 130.1, 129.4, 128.8, 128.6, 128.5, 127.3, 124.1 (q, *J* = 288.1 Hz, CF_3_), 123.7 (q, *J* = 273.0 Hz, CF_3_), 122.5, 121.8, 118.2, 80.4, 63.8 (q, *J* = 26.6 Hz, >C<), 52.7, 27.9. ^19^F NMR (376 MHz, CDCl_3_) *δ* −63.08 (s, 3F, CF_3_), −75.05 (s, 3F, CF_3_). Elemental analysis calcd (%) for C_26_H_24_F_6_N_2_O_5_: C, 55.92; H, 4.33; N, 5.02; found: C, 55.71; H, 4.57; N, 5.27.

*Methyl 2-(benzyloxycarbonylamino)-3,3,3-trifluoro-2-((1-oxo-4-phenyl-6-(trifluoromethyl)-1,2-dihydroisoquinolin-3-yl)methyl)propanoate* (**3n**).

Yield 73% as a white solid. M.p. 195–196 °C. ^1^H NMR (500 MHz, DMSO-*d_6_*): *δ* 11.18 (s, 1H, NH), 8.63 (s, 1H, NH), 8.47 (d, *J* = 8.3 Hz, 1H, Ar), 7.84 (d, *J* = 8.3 Hz, 1H, Ar), 7.57–7.48 (m, 3H, Ar), 7.37–7.33 (m, 5H, Ar), 7.30 (d, *J* = 7.2 Hz, 1H, Ar), 7.20 (s, 1H, Ar), 7.14 (d, *J* = 7.6 Hz, 1H, Ar), 5.08 (d, *J* = 12.3 Hz, 1H, OCH_2_), 5.02 (d, *J* = 12.2 Hz, 1H, OCH_2_), 3.51 (s, 3H, OCH_3_), 3.22 (d, *J* = 15.1 Hz, 1H, CH_2_), 2.99 (d, *J* = 15.1 Hz, 1H, CH_2_). ^13^C{^1^H} NMR (126 MHz, DMSO-*d_6_*): *δ* 164.0, 160.6, 154.4, 138.0, 136.0, 133.8, 133.6, 132.3 (q, *J* = 31.8 Hz, *C_Ar_*-CF_3_), 131.4, 130.9, 129.3, 128.7, 128.6, 128.5, 128.2, 128.1, 127.2, 123.9 (q, *J* = 288.0 Hz, CF_3_), 123.9 (q, *J* = 273.1 Hz, CF_3_), 122.5, 121.8, 118.2, 66.5, 64.0 (q, *J* = 26.6 Hz, >C<), 52.9, 31.3. ^19^F NMR (282 MHz, CDCl_3_): *δ −*62.98 (s, 3F, CF_3_), −75.16 (s, 3F, CF_3_). Elemental analysis calcd (%) for C_29_H_22_F_6_N_2_O_5_: C, 58.79; H, 3.74; N, 4.73; found: C, 59.01; H, 3.81; N, 4.95.

*Methyl 2-(tert-butoxycarbonylamino)-3,3,3-trifluoro-2-((1-oxo-4-p-tolyl-6-(trifluoromethyl)-1,2-dihydroisoquinolin-3-yl)methyl)propanoate* (**3o**).

Yield 89% as a white solid. M.p. 201–203 °C. ^1^H NMR (400 MHz, CDCl_3_) *δ* 11.99 (s, 1H, NH), 8.58 (d, *J* = 8.4 Hz, 1H, Ar), 7.70 (d, *J* = 8.4 Hz, 1H, Ar), 7.40–7.33 (m, 3H, Ar), 7.14–7.08 (m, 2H, Ar), 6.49 (s, 1H, NH), 3.65 (s, 3H, OCH_3_), 3.49 (d, *J* = 15.1 Hz, 1H, CH_2_), 3.32 (d, *J* = 15.1 Hz, 1H, CH_2_), 2.46 (s, 3H, CH_3_), 1.14 (s, 9H, CH_3_). ^13^C{^1^H} NMR (101 MHz, CDCl_3_): *δ* 165.6, 162.9, 154.4, 139.0, 138.7, 134.4 (q, *J* = 32.2 Hz, C*_Ar_*-CF_3_), 132.9, 131.2, 130.8, 130.7, 130.3, 130.0, 128.6, 127.1, 123.9 (q, *J* = 288.2 Hz, CF_3_), 123.7 (q, *J* = 273.0 Hz, CF_3_), 123.1, 122.9, 121.0, 81.5, 64.9 (q, *J* = 27.2 Hz, >C<), 53.5, 31.9, 27.9, 21.5. ^19^F NMR (376 MHz, CDCl_3_) *δ* −63.03 (s, 3F, CF_3_), −75.05 (s, 3F, CF_3_). Elemental analysis calcd (%) for C_27_H_26_F_6_N_2_O_5_: C, 56.64; H, 4.58; N, 4.89; found: C, 56.71; H, 4.57; N, 4.77.

*Methyl 2-(benzyloxycarbonylamino)-3,3,3-trifluoro-2-((1-oxo-4-p-tolyl-6-(trifluoromethyl)-1,2-dihydroisoquinolin-3-yl)methyl)propanoate* (**3p**).

Yield 70% as a white solid. M.p. 213–214 °C. ^1^H NMR (500 MHz, DMSO-*d_6_*): *δ* 11.16 (s, 1H, NH), 8.62 (s, 1H, NH), 8.47 (s, 1H, Ar), 7.83 (s, 1H, Ar), 7.36–7.17 (m, 9H, Ar), 7.02 (s, 1H, Ar), 5.08 (d, *J* = 12.4 Hz, 1H, OCH_2_), 5.02 (d, *J* = 12.5 Hz, 1H, OCH_2_), 3.50 (s, 3H, OCH_3_), 3.24 (d, *J* = 15.5 Hz, 1H, CH_2_), 2.98 (d, *J* = 15.2 Hz, 1H, CH_2_), 2.39 (s, 3H, CH_3_). ^13^C{^1^H} NMR (126 MHz, DMSO-*d_6_*): *δ* 164.1, 160.6, 154.5, 138.2, 137.7, 136.0, 133.8, 132.3 (q, *J* = 31.6 Hz, *C_Ar_*-CF_3_), 131.2, 130.8, 130.5, 129.9, 129.3, 128.6, 128.5, 128.2, 128.1, 127.3, 123.9 (q, *J* = 286.1 Hz, CF_3_), 123.7 (q, *J* = 272.8 Hz, CF_3_), 122.4, 121.8, 118.1, 66.5, 64.0 (q, *J* = 28.3 Hz, >C<), 52.8, 31.3, 20.9. ^19^F NMR (376 MHz, CDCl_3_): *δ −*62.98 (s, 3F, CF_3_), −75.20 (s, 3F, CF_3_). Elemental analysis calcd (%) for C_30_H_24_F_6_N_2_O_5_: C, 59.41; H, 3.99; N, 4.62; found: C, 59.67; H, 4.15; N, 4.89.

*Methyl 2-(tert-butoxycarbonylamino)-3,3,3-trifluoro-2-((4-(4-methoxyphenyl)-1-oxo-6-(trifluoromethyl)-1,2-dihydroisoquinolin-3-yl)methyl)propanoate* (**3q**).

Yield 90% as a white solid. M.p. 206–207 °C. ^1^H NMR (300 MHz, CDCl_3_): *δ* 11.30 (s, 1H, NH), 8.57 (d, *J* = 8.3 Hz, 1H, Ar), 7.69 (d, *J* = 8.4 Hz, 1H, Ar), 7.38 (s, 1H, Ar), 7.19–7.05 (m, 4H, Ar), 6.09 (s, 1H, NH), 3.91 (s, 3H, OCH_3_), 3.70 (s, 3H, OCH_3_), 3.48 (d, *J* = 15.0 Hz, 1H, CH_2_), 3.36 (d, *J* = 14.9 Hz, 1H, CH_2_), 1.19 (s, 9H, 3 CH_3_). ^13^C{^1^H} NMR (126 MHz, CDCl_3_): *δ* 166.0, 162.3, 159.8, 154.4, 139.2, 134.3 (q, *J* = 32.5 Hz, C*_Ar_*-CF_3_), 133.1, 132.5, 132.1, 128.7, 127.3, 126.0, 123.8 (q, *J* = 287.8 Hz, CF_3_), 123.7 (q, *J* = 273.3 Hz, CF_3_), 123.0, 122.9, 120.4, 114.9, 114.8, 81.9, 65.1 (q, *J* = 27.4 Hz, >C<), 55.5, 53.9, 27.9. ^19^F NMR (282 MHz, CDCl_3_): *δ* −62.98 (s, 3F, CF_3_), −75.03 (s, 3F, CF_3_). Elemental analysis calcd (%) for C_27_H_26_F_6_N_2_O_6_: C, 55.10; H, 4.45; N, 4.76; found: C, 55.21; H, 4,57; N, 4.73.

*Methyl 2-(benzyloxycarbonylamino)-3,3,3-trifluoro-2-((4-(4-methoxyphenyl)-1-oxo-6-(trifluoromethyl)-1,2-dihydroisoquinolin-3-yl)methyl)propanoate* (**3r**).

Yield 82% as a white solid. M.p. 199–200 °C. ^1^H NMR (500 MHz, DMSO-*d_6_*): *δ* 11.15 (s, 1H, NH), 8.61 (s, 1H, NH), 8.46 (d, *J* = 8.3 Hz, 1H, Ar), 7.82 (d, *J* = 8.4 Hz, 1H, Ar), 7.36 (s, 5H, Ar), 7.25 (s, 1H, Ar), 7.21 (d, *J* = 7.9 Hz, 1H, Ar), 7.11–7.04 (m, 3H, Ar), 5.08 (d, *J* = 12.3 Hz, 1H, OCH_2_), 5.02 (d, *J* = 12.4 Hz, 1H, OCH_2_), 3.83 (s, 3H, OCH_3_), 3.51 (s, 3H, OCH_3_), 3.26 (d, *J* = 15.5 Hz, 1H, CH_2_), 2.98 (d, *J* = 15.1 Hz, 1H, CH_2_). ^13^C{^1^H} NMR (126 MHz, DMSO-*d_6_*): *δ* 164.1, 160.7, 159.0, 154.5, 138.4, 136.0, 133.9, 132.6, 132.5 (q, *J* = 31.7 Hz, *C_Ar_*-CF_3_), 132.2, 128.5, 128.3, 128.1, 127.3, 125.4, 124.0 (q, *J* = 288.5 Hz, CF_3_), 123.7 (q, *J* = 272.8 Hz, CF_3_), 122.4, 121.9, 117.9, 114.6, 114.1, 66.5, 64.0 (q, *J* = 27.7 Hz, >C<), 55.2, 52.9, 31.3. ^19^F NMR (282 MHz, DMSO-*d_6_*): *δ −*61.70 (s, 3F, CF_3_), −74.06 (s, 3F, CF_3_). Elemental analysis calcd (%) for C_30_H_24_F_6_N_2_O_6_: C, 57.88; H, 3.89; N, 4.50; found: C, 57.79; H, 4.05; N, 4.61.

*Methyl 2-(benzyloxycarbonylamino)-3,3,3-trifluoro-2-((4-(4-nitrophenyl)-1-oxo-1,2-dihydroisoquinolin-3-yl)methyl)propanoate* (**3s**).

Yield 30% as a yellow solid (eluent petroleum ether/ethyl acetate = 5/1). ^1^H NMR (500 MHz, DMSO-*d_6_*): *δ* 10.98 (s, 1H, NH), 8.62 (s, 1H, NH), 8.39 (d, *J* = 8.6 Hz, 1H, Ar), 8.34 (d, *J* = 8.4 Hz, 1H, Ar), 8.29 (d, *J* = 8.0 Hz, 1H, Ar), 7.65–7.60 (m, 2H, Ar), 7.55 (t, *J* = 7.6 Hz, 1H, Ar), 7.42 (d, *J* = 8.5 Hz, 1H, Ar), 7.36 (s, 5H, Ar), 6.94 (d, *J* = 8.1 Hz, 1H, Ar), 5.07 (d, *J* = 12.4 Hz, 1H, OCH_2_), 5.01 (d, *J* = 12.2 Hz, 1H, OCH_2_), 3.50 (s, 3H, OCH_3_), 3.19 (d, *J* = 15.4 Hz, 1H, CH_2_), 3.03 (d, *J* = 15.2 Hz, 1H, CH_2_). ^13^C{^1^H} NMR (126 MHz, DMSO-*d_6_*): *δ* 164.8, 161.9, 154.9, 147.5, 142.5, 137.4, 136.4, 133.9, 133.4, 133.1, 132.5, 128.9, 128.7, 128.5, 127.5, 127.3, 125.3, 125.2, 124.5, 124.3 (q, *J* = 287.3, CF_3_), 123.9, 117.2, 66.9, 64.5 (q, *J* = 26.2 Hz, >C<), 53.4, 31.9. ^19^F NMR (282 MHz, DMSO-*d_6_*): *δ* −73.83 (s, 3F, CF_3_). Elemental analysis calcd (%) for C_28_H_22_F_3_N_3_O_7_: C, 59.05; H, 3.89; N, 7.38; found: C, 59.31; H, 4.07; N, 7.61.

*Methyl 2-(benzyloxycarbonylamino)-3,3,3-trifluoro-2-((4-(4-nitrophenyl)-1-oxo-1,2-dihydroisoquinolin-3-yl)methyl)propanoate* (**3t**).

Yield 25% as a yellow solid (eluent petroleum ether/ethyl acetate = 4/1). ^1^H NMR (400 MHz, acetone-*d_6_*): *δ* 10.46 (s, 1H, NH), 8.36 (d, *J* = 8.0 Hz, 1H, Ar), 8.31 (d, *J* = 8.2 Hz, 2H, Ar), 7.88 (d, *J* = 7.9 Hz, 3H, Ar), 7.78 (t, *J* = 7.8 Hz, 1H, Ar), 7.58 (t, *J* = 7.6 Hz, 1H, Ar), 7.41–7.30 (m, 5H, Ar), 6.49 (s, 1H, NH), 4.99 (d, *J* = 12.4 Hz, 1H, OCH_2_), 4.91 (d, *J* = 12.4 Hz, 1H, OCH_2_), 3.84 (d, *J* = 16.0 Hz, 1H, CH_2_), 3.59 (d, *J* = 16.0 Hz, 1H, CH_2_), 3.27 (s, 3H, OCH_3_). ^13^C{^1^H} NMR (151 MHz, DMSO-*d_6_*): *δ* 165.3, 161.3, 154.5, 147.6, 140.7, 139.8, 137.7, 136.3, 132.2, 132.0, 131.3, 128.4, 128.3, 128.1, 127.9, 127.5, 126.9, 126.7, 126.2 (q, *J* = 288.2 Hz, CF_3_), 125.4, 123.3, 106.3, 66.0, 64.3 (q, *J* = 26.6 Hz, >C<), 52.3, 28.9. ^19^F NMR (376 MHz, acetone-*d_6_*): δ −73.99 (s, 3F, CF_3_). Elemental analysis calcd (%) for C_28_H_22_F_3_N_3_O_7_: C, 59.05; H, 3.89; N, 7.38; found: C, 59.27; H, 4.11; N, 7.40.

*Methyl 2-(tert-butoxycarbonylamino)-3,3,3-trifluoro-2-((4-phenyl-1-(trifluoromethyl-sulfonyloxy)isoquinolin-3-yl)methyl)propanoate* (**4a**).

Yield 77% as a white solid (eluent petroleum ether/ethyl acetate = 15/1). M.p. 152–154 °C. ^1^H NMR (400 MHz, CDCl_3_): *δ* 8.12 (d, *J* = 8.2 Hz, 1H, Ar), 7.73–7.66 (m, 2H, Ar), 7.60–7.56 (m, 1H, Ar), 7.55–7.50 (m, 2H, Ar), 7.41 (t, *J* = 8.8 Hz, 2H, Ar), 7.23–7.20 (m, 1H, Ar), 6.85 (s, 1H, NH), 3.87–3.84 (m, 3H, OCH_3_, 1H, CH_2_), 3.53 (d, *J* = 15.9 Hz, 1H, CH_2_), 1.28 (s, 9H 3 CH_3_). ^13^C{^1^H} NMR (101 MHz, CDCl_3_): *δ* 166.9, 153.7, 150.8, 143.4, 139.9, 134.7, 134.5, 132.2, 130.3, 130.0, 129.3, 128.8, 128.7, 126.6, 126.3, 124.2 (q, *J* = 288.7 Hz, CF_3_), 122.7, 118.7 (q, *J* = 320.2 Hz, CF_3_), 118.5, 80.2, 64.4 (q, *J* = 28.2 Hz, >C<), 53.7, 33.8, 28.0. ^19^F NMR (282 MHz, CDCl_3_): *δ −*73.69 (s, 3F, CF_3_), −73.95 (s, 3F, CF_3_). Elemental analysis calcd (%) for C_26_H_24_F_6_N_2_O_7_S: C, 50.16; H, 3.89; N, 4.50; found: C, 50.07; H, 4.01; N, 4.58.

*Methyl 2-(benzyloxycarbonylamino)-3,3,3-trifluoro-2-((4-phenyl-1-(trifluoromethyl-sulfonyloxy)isoquinolin-3-yl)methyl)propanoate* (**4b**).

Yield 92% as a white solid (eluent petroleum ether/ethyl acetate = 15/1). M.p. 140–142 °C. ^1^H NMR (400 MHz, CDCl_3_): *δ* 8.09 (d, *J* = 8.0 Hz, 1H, Ar), 7.74–7.67 (m, 2H, Ar), 7.57–7.51 (m, 3H, Ar), 7.38 (d, *J* = 8.1 Hz, 1H, Ar), 7.21–7.17 (m, 2H, Ar), 7.07–7.06 (m, 2H, Ar), 6.95–6.93 (m, 3H, Ar), 6.85 (s, 1H, NH), 5.05 (d, *J* = 12.3 Hz, 1H, OCH_2_), 4.84 (d, *J* = 12.2 Hz, 1H, OCH_2_), 4.08 (d, *J* = 16.1 Hz, 1H, CH_2_), 3.92 (s, 3H, OCH_3_), 3.62 (d, *J* = 16.4 Hz, 1H, CH_2_). ^13^C{^1^H} NMR (101 MHz, CDCl_3_): *δ* 166.6, 153.9, 150.6, 142.9, 139.9, 136.5, 134.7, 134.3, 132.0, 130.3, 129.9, 129.2, 128.7, 128.6, 127.9, 127.7, 127.4, 126.3, 124.1 (q, *J* = 289.0 Hz, CF_3_), 122.6, 118.6 (q, *J* = 320.2 Hz, CF_3_), 118.2, 66.7, 64.4 (q, *J* = 27.0 Hz, >C<), 54.2, 32.9, 29.8. ^19^F NMR (376 MHz, CDCl_3_): *δ −*74.11 (s, 3F, CF_3_), −74.67 (s, 3F, CF_3_). Elemental analysis calcd (%) for C_29_H_22_F_6_N_2_O_7_S: C, 53.05; H, 3.38; N, 4.27; found: C, 53.37; H, 3.49; N, 4.52.

*Methyl 2-(tert-butoxycarbonylamino)-3,3,3-trifluoro-2-((4-p-tolyl-1-(trifluoromethyl-sulfonyloxy)isoquinolin-3-yl)methyl)propanoate* (**4c**).

Yield 89% as a white solid (eluent petroleum ether/ethyl acetate = 15/1). M.p. 145–146 °C. ^1^H NMR (500 MHz, CDCl_3_): *δ* 8.12 (d, *J* = 7.4 Hz, 1H, Ar), 7.71–7.66 (m, 2H, Ar), 7.46 (d, *J* = 7.6 Hz, 1H, Ar), 7.38 (d, *J* = 7.5 Hz, 1H, Ar), 7.33 (d, *J* = 7.4 Hz, 1H, Ar), 7.28 (s, 1H, Ar), 7.09 (d, *J* = 7.3 Hz, 1H, Ar), 6.85 (s, 1H, NH), 3.87–3.84 (s, 3H, OCH_3_, 1H, CH_2_), 3.52 (d, *J* = 15.9 Hz, 1H, CH_2_), 2.48 (s, 3H, CH_3_), 1.28 (s, 9H, 3 CH_3_). ^13^C{^1^H} NMR (126 MHz, CDCl_3_): *δ* 167.0, 153.7, 150.7, 143.5, 140.1, 138.6, 134.7, 132.1, 131.7, 130.2, 129.9, 129.8, 129.6, 128.8, 126.5, 124.3 (q, *J* = 288.4 Hz, CF_3_), 122.7, 119.1 (q, *J* = 320.2 Hz, CF_3_), 118.5, 80.2, 64.4 (q, *J* = 27.9 Hz, >C<), 53.7, 33.8, 28.0, 21.5. ^19^F NMR (282 MHz, CDCl_3_): *δ −*73.67 (s, 3F, CF_3_), −73.83 (s, 3F, CF_3_). Elemental analysis calcd (%) for C_27_H_26_F_6_N_2_O_7_S: C, 50.94; H, 4.12; N, 4.40; found: C, 51.04; H, 4.25; N, 4.43.

*Methyl 2-(benzyloxycarbonylamino)-3,3,3-trifluoro-2-((4-p-tolyl-1-(trifluoromethyl-sulfonyloxy)isoquinolin-3-yl)methyl)propanoate* (**4d**).

Yield 90% as a thick oil (eluent petroleum ether/ethyl acetate = 15/1). ^1^H NMR (500 MHz, CDCl_3_): *δ* 8.07 (d, *J* = 8.0 Hz, 1H, Ar), 7.72–7.66 (m, 2H, Ar), 7.40 (d, *J* = 8.2 Hz, 1H, Ar), 7.35 (d, *J* = 7.8 Hz, 1H, Ar), 7.31 (d, *J* = 7.7 Hz, 1H, Ar), 7.08–7.03 (m, 4H, Ar), 6.98–6.92 (m, 3H, Ar), 6.83 (s, 1H, NH), 5.03 (d, *J* = 12.5 Hz, 1H, OCH_2_), 4.84 (d, *J* = 12.6 Hz, 1H, OCH_2_), 4.06 (d, *J* = 16.6 Hz, 1H, CH_2_), 3.90 (s, 3H, OCH_3_), 3.59 (d, *J* = 16.5 Hz, 1H, CH_2_), 2.47 (s, 3H, CH_3_). ^13^C{^1^H} NMR (126 MHz, CDCl_3_): *δ* 166.7, 153.9, 150.5, 143.0, 140.1, 138.5, 136.6, 134.5, 131.9, 131.6, 130.1, 129.9, 129.8, 129.5, 128.6, 128.0, 127.7, 127.4, 126.5, 124.1 (q, *J* = 288.8 Hz, CF_3_), 122.6, 118.7 (q, *J* = 320.2 Hz, CF_3_), 118.3, 66.7, 64.5 (q, *J* = 28.1 Hz, >C<), 54.1, 33.0, 21.5. ^19^F NMR (282 MHz, CDCl_3_): *δ −*74.01 (s, 3F, CF_3_), −74.52 (s, 3F, CF_3_). Elemental analysis calcd (%) for C_30_H_24_F_6_N_2_O_7_S: C, 53.73; H, 3.61; N, 4.18; found: C, 53.98; H, 3.90; N, 4.25.

*Methyl 2-(tert-butoxycarbonylamino)-3,3,3-trifluoro-2-((4-(4-methoxyphenyl)-1-(trifluoro-methylsulfonyloxy)isoquinolin-3-yl)methyl)propanoate* (**4e**).

Yield 70% as a white solid (eluent petroleum ether/ethyl acetate = 15/1). M.p. 112–114 °C. ^1^H NMR (300 MHz, CDCl_3_): *δ* 8.11–8.09 (m, 1H, Ar), 7.71–7.68 (m, 2H, Ar), 7.48–7.47 (m, 1H, Ar), 7.32–7.29 (m, 1H, Ar), 7.11–7.03 (m, 3H, Ar), 6.84 (s, 1H, NH), 3.91 (s, 3H, OCH_3_, 0.5 CH_2_), 3.84 (s, 3H, OCH_3_, 0.5 CH_2_), 3.52 (d, *J* = 16.0 Hz, 1H, CH_2_), 1.27 (s, 9H, 3 CH_3_). ^13^C{^1^H} NMR (126 MHz, CDCl_3_): *δ* 167.0, 159.9, 153.7, 150.7, 143.7, 140.3, 134.4, 132.1, 131.6, 131.2, 128.8, 126.7, 126.4, 124.3 (q, *J* = 288.4 Hz, CF_3_), 122.7, 118.7 (q, *J* = 320.2 Hz, CF_3_), 118.5, 114.6, 114.5, 80.1, 64.4 (q, *J* = 27.5 Hz, >C<), 55.5, 53.7, 33.8, 28.0. ^19^F NMR (282 MHz, CDCl_3_): *δ −*73.70 (s, 3F, CF_3_), −73.89 (s, 3F, CF_3_). Elemental analysis calcd (%) for C_27_H_26_F_6_N_2_O_8_S: C, 49.69; H, 4.02; N, 4.29; found: C, 49.64; H, 4.05; N, 4.23.

*Methyl 2-(tert-butoxycarbonylamino)-3,3,3-trifluoro-2-((6-nitro-4-phenyl-1-(trifluoro methylsulfonyloxy)isoquinolin-3-yl)methyl)propanoate* (**4f**).

Yield 79% as a white solid (eluent petroleum ether/ethyl acetate = 15/1). M.p. 162–164 °C. ^1^H NMR (400 MHz, CDCl_3_): *δ* 8.49 (d, *J* = 9.2 Hz, 1H, Ar), 8.39 (s, 1H, Ar), 8.34 (d, *J* = 9.2 Hz, 1H, Ar), 7.73–7.66 (m, 3H, Ar), 7.53 (s, 1H, Ar), 7.31 (s, 1H, Ar), 6.56 (s, 1H, NH), 4.14 (d, *J* = 16.5 Hz, 1H, CH_2_), 3.95 (s, 3H, OCH_3_), 3.69 (d, *J* = 16.5 Hz, 1H, CH_2_), 1.28 (s, 9H, 3 CH_3_). ^13^C{^1^H} NMR (101 MHz, CDCl_3_): *δ* 166.7, 153.4, 150.4, 149.6, 146.6, 139.5, 135.9, 133.2, 130.1, 129.9, 129.8, 129.7, 129.3, 125.2, 124.1 (q, *J* = 288.3 Hz, CF_3_), 122.4, 122.2, 120.2, 118.7 (q, *J* = 320.0 Hz, S-CF_3_),80.2, 64.2 (q, *J* = 26.6 Hz. >C<), 54.0, 33.5, 27.9. ^19^F NMR (376 MHz, CDCl_3_): *δ −*73.63 (s, 3F, CF_3_), −74.85 (s, 3F, CF_3_). Elemental analysis calcd (%) for C_26_H_23_F_6_N_3_O_9_S: C, 46.78; H, 3.47; N, 6.29; found: C, 46.71; H, 3.49; N, 6.09.

*Methyl 2-(benzyloxycarbonylamino)-3,3,3-trifluoro-2-((6-nitro-4-phenyl-1-(trifluoro methylsulfonyloxy)isoquinolin-3-yl)methyl)propanoate* (**4g**).

Yield 89% as a yellow solid (eluent petroleum ether/ethyl acetate = 15/1). M.p. 123–125 °C. ^1^H NMR (400 MHz, CDCl_3_): *δ* 8.40 (s, 1H, Ar), 8.21 (s, 2H, Ar), 7.57 (s, 3H, Ar), 7.20 (s, 2H, Ar), 7.03 (s, 2H, Ar), 6.88 (s, 3H, Ar), 6.58 (s, 1H, NH), 4.97 (d, *J* = 12.4 Hz, 1H, OCH_2_), 4.76 (d, *J* = 12.6 Hz, 1H, OCH_2_), 4.18 (d, *J* = 16.9 Hz, 1H CH_2_), 3.91 (s, 3H, OCH_3_), 3.66 (d, *J* = 17.0 Hz, 1H, CH_2_). ^13^C{^1^H} NMR (101 MHz, CDCl_3_): *δ* 166.4, 153.6, 150.2, 149.5, 146.0, 139.4, 136.5, 135.7, 133.1, 130.0, 129.8, 129.7, 129.6, 129.2, 127.9, 127.7, 127.6, 125.1, 124.0 (q, *J* = 290.0 Hz, CF_3_), 122.3, 121.9, 119.9, 118.6 (q, *J* = 320.0 Hz, CF_3_), 66.7, 64.3 (q, *J* = 28.5 Hz, CF_3_), 54.4, 32.9. ^19^F NMR (282 MHz, CDCl_3_): *δ −*73.84 (s, 3F, CF_3_), −75.29 (s, 3F, CF_3_). Elemental analysis calcd (%) for C_29_H_21_F_6_N_3_O_9_S: C, 49.65; H, 3.02; N, 5.99; found: C, 49.52; H, 3.01; N, 5.87.

*Methyl 2-(tert-butoxycarbonylamino)-3,3,3-trifluoro-2-((6-nitro-4-p-tolyl-1-(trifluoromethylsulfonyloxy)isoquinolin-3-yl)methyl)propanoate* (**4h**).

Yield 82% as a white solid (eluent petroleum ether/ethyl acetate = 9/1). M.p. 154–155 °C. ^1^H NMR (500 MHz, CDCl_3_): *δ* 8.41 (d, *J* = 9.3 Hz, 1H, Ar), 8.37 (s, 1H, Ar), 8.26 (d, *J* = 9.2 Hz, 1H, Ar), 7.45 (d, *J* = 7.8 Hz, 1H, Ar), 7.38 (d, *J* = 7.8 Hz, 1H, Ar), 7.32 (d, *J* = 7.9 Hz, 1H, Ar), 7.12 (d, *J* = 6.9 Hz, 1H, Ar), 6.49 (s, 1H, NH), 4.08 (d, *J* = 16.4 Hz, 1H, CH_2_), 3.88 (s, 3H, OCH_3_), 3.62 (d, *J* = 16.4 Hz, 1H, CH_2_), 2.51 (s, 3H, CH_3_), 1.22 (s, 9H, CH_3_). ^13^C{^1^H} NMR (126 MHz, CDCl_3_): *δ* 166.7, 153.4, 150.3, 149.6, 146.7, 139.6, 136.2, 130.5, 130.1, 130.0, 129.9, 129.8, 125.1, 124.1 (q, *J* = 288.6 Hz, CF_3_), 122.5, 122.1, 120.2, 118.7 (q, *J* = 320.3 Hz, CF_3_), 114.2, 80.2, 64.2 (q, *J* = 28.1 Hz, >C<), 54.0, 33.5, 27.9, 21.6. ^19^F NMR (282 MHz, CDCl_3_): *δ* −73.56 (s, 3F, CF_3_), −74.68 (s, 3F, CF_3_). Elemental analysis calcd (%) for C_27_H_25_F_6_N_3_O_9_S: C, 47.58; H, 3.70; N, 6.17; found: C, 47.61; H, 3.77; N, 6.15.

*Methyl 2-(tert-butoxycarbonylamino)-3,3,3-trifluoro-2-((4-(4-methoxyphenyl)-6-nitro-1-(trifluoromethylsulfonyloxy)isoquinolin-3-yl)methyl)propanoate* (**4i**).

Yield 88% as a white solid (eluent petroleum ether/ethyl acetate = 9/1). M.p. 149–150 °C. ^1^H NMR (500 MHz, CDCl_3_): *δ* 8.41 (d, *J* = 9.1 Hz, 1H, Ar), 8.38 (s, 1H, Ar), 8.26 (d, *J* = 9.1 Hz, 1H, Ar), 7.37 (d, *J* = 8.4 Hz, 1H, Ar), 7.18–7.14 (m, 2H, Ar), 7.10 (d, *J* = 8.5 Hz, 1H, Ar), 6.48 (s, 1H, NH), 4.10 (d, *J* = 16.3 Hz, 1H, CH_2_), 3.95 (s, 3H, OCH_3_), 3.88 (s, 3H, OCH_3_), 3.63 (d, *J* = 16.4 Hz, 1H, CH_2_), 1.21 (s, 9H, 3 CH_3_). ^13^C{^1^H} NMR (126 MHz, CDCl_3_): *δ* 166.7, 160.4, 153.4, 150.2, 149.6, 146.9, 139.8, 135.9, 131.4, 131.2, 125.1, 125.0, 124.1 (q, *J* = 288.5 Hz, CF_3_), 122.5, 122.0, 120.2, 118.7 (q, *J* = 320.2 Hz, CF_3_) 115.1, 115.0, 80.2, 64.2 (q, *J* = 28.6 Hz, >C<), 55.5, 54.0, 33.5, 27.9. ^19^F NMR (282 MHz, CDCl_3_): *δ* −73.54 (s, 3F, CF_3_), −74.72 (s, 3F, CF_3_). Elemental analysis calcd (%) for C_27_H_25_F_6_N_3_O_10_S: C, 46.49; H, 3.61; N, 6.02; found: C, 46.62; H, 3.64; N, 6.12.

*Methyl 2-(benzyloxycarbonylamino)-3,3,3-trifluoro-2-((4-(4-methoxyphenyl)-6-nitro-1-(trifluoromethylsulfonyloxy)isoquinolin-3-yl)methyl)propanoate* (**4j**).

Yield 94% as a yellow solid (eluent petroleum ether/ethyl acetate = 15/1). M.p. 152–154 °C. ^1^H NMR (400 MHz, CDCl_3_): *δ* 8.41 (d, *J* = 9.2 Hz, 1H, Ar), 8.28 (s, 1H, Ar), 8.19 (d, *J* = 9.1 Hz, 1H, Ar), 7.14–7.02 (m, 6H, Ar), 6.89 (s, 3H, Ar), 6.58 (s, 1H, NH), 4.97 (d, *J* = 12.3 Hz, 1H, OCH_2_), 4.77 (d, *J* = 12.1 Hz, 1H, OCH_2_), 4.21 (d, *J* = 16.4 Hz, 1H CH_2_), 3.95 (s, 3H, OCH_3_), 3.93 (s, 3H, OCH_3_), 3.66 (d, *J* = 16.8 Hz, 1H, CH_2_). ^13^C{^1^H} NMR (126 MHz, CDCl_3_): *δ* 166.4, 160.4, 153.7, 150.0, 149.5, 146.4, 139.7, 136.5, 135.7, 131.4, 131.0, 127.9, 127.6, 127.5, 125.1, 124.9, 124.0 (q, *J* = 285.8 Hz, CF_3_), 122.5, 121.8, 119.9, 118.6 (q, *J* = 320.4 Hz, CF_3_), 115.0, 114.9, 66.7, 64.3 (q, *J* = 28.5 Hz, >C<), 55.5, 54.5, 32.9. ^19^F NMR (282 MHz, CDCl_3_): *δ −*73.83 (s, 3F, CF_3_), −75.22 (s, 3F, CF_3_). Elemental analysis calcd (%) for C_30_H_23_F_6_N_3_O_10_S: C, 49.25; H, 3.17; N, 5.74; found: C, 49.29; H, 3.08; N, 5.84.

*Methyl 2-(tert-butoxycarbonylamino)-3,3,3-trifluoro-2-((4-phenyl-6-(trifluoromethyl)-1-(trifluoromethylsulfonyloxy)isoquinolin-3-yl)methyl)propanoate* (**4k**).

Yield 81% as a white solid (eluent petroleum ether/ethyl acetate = 15/1). M.p. 146–148 °C. ^1^H NMR (500 MHz, CDCl_3_): *δ* 8.25 (d, *J* = 8.8 Hz, 1H, Ar), 7.87 (d, *J* = 8.8 Hz, 1H, Ar), 7.72 (s, 1H, Ar), 7.63 (s, 1H, Ar), 7.57 (s, 2H, Ar), 7.44 (s, 1H, Ar), 7.24 (s, 1H, Ar), 6.59 (s, 1H, NH), 4.01 (d, *J* = 16.1 Hz, 1H, CH_2_), 3.87 (s, 3H, OCH_3_), 3.60 (d, *J* = 16.3 Hz, 1H, CH_2_), 1.24 (s, 9H, 3 CH_3_). ^13^C{^1^H} NMR (126 MHz, CDCl_3_): *δ* 166.8, 153.5, 150.5, 145.6, 139.2, 135.1, 133.8 (q, *J* = 33.5 Hz, C*_Ar_*-CF_3_), 133.7, 130.1, 129.9, 129.6, 129.4, 129.2, 124.6, 124.2 (q, *J* = 288.4 Hz, CF_3_), 124.2, 123.9–123.8 (m), 123.3 (q, *J* = 273.2 Hz, CF_3_), 119.5, 118.7 (q, *J* = 320.2 Hz, CF_3_), 80.2, 64.3 (q, *J* = 28.4 Hz, >C<), 53.9, 33.5, 27.9. ^19^F NMR (282 MHz, CDCl_3_): *δ −*73.69 (s, 3F, CF_3_), −73.89 (s, 3F, CF_3_). Elemental analysis calcd (%) for C_27_H_23_F_9_N_2_O_7_S: C, 46.96; H, 3.36; N, 4.06; found: C, 46.87; H, 3.32; N, 4.05.

*Methyl 2-(benzyloxycarbonylamino)-3,3,3-trifluoro-2-((4-phenyl-6-(trifluoromethyl)-1-(trifluoromethylsulfonyloxy)isoquinolin-3-yl)methyl)propanoate* (**4l**).

Yield 80% as a white solid (eluent petroleum ether/ethyl acetate = 15/1). M.p. 109–111 °C. ^1^H NMR (300 MHz, CDCl_3_): *δ* 8.18 (d, *J* = 8.8 Hz, 1H, Ar), 7.87 (d, *J* = 8.8 Hz, 1H, Ar), 7.60–7.53 (m, 4H, Ar), 7.18–7.12 (m, 2H, Ar), 7.01 (s, 2H, Ar), 6.87 (s, 3H, Ar), 6.62 (s, 1H, NH), 4.99 (d, *J* = 12.5 Hz, 1H, OCH_2_), 4.76 (d, *J* = 12.5 Hz, 1H, OCH_2_), 4.12 (d, *J* = 16.9 Hz, 1H, CH_2_), 3.92 (s, 3H, OCH_3_), 3.63 (d, *J* = 16.8 Hz, 1H, CH_2_). ^13^C{^1^H} NMR (101 MHz, CDCl_3_): *δ* 166.5, 153.7, 150.3, 145.0, 139.1, 136.6, 134.9, 133.6 (q, *J* = 33.1 Hz, *C_Ar_*-CF_3_), 133.5, 130.1, 129.8, 129.5, 129.2, 129.0, 127.8, 127.6, 127.4, 124.3, 124.2, 124.0 (q, *J* = 289.1 Hz, CF_3_), 123.9, 123.4 (q, *J* = 274.3 Hz, CF_3_), 119.2, 118.6 (q, *J* = 320.4 Hz, CF_3_), 66.7, 64.3 (q, *J* = 28.8 Hz, >C<), 54.4, 32.8. ^19^F NMR (282 MHz, CDCl_3_): *δ −*63.22 (s, 3F, CF_3_), −73.92 (s, 3F, CF_3_), −75.08 (s, 3F, CF_3_). Elemental analysis calcd (%) for C_30_H_21_F_9_N_2_O_7_S: C, 49.73; H, 2.92; N, 3.87; found: C, 49.75; H, 2.96; N, 3.83.

*Methyl 2-(tert-butoxycarbonylamino)-3,3,3-trifluoro-2-((4-p-tolyl-6-(trifluoromethyl)-1-(trifluoromethylsulfonyloxy)isoquinolin-3-yl)methyl)propanoate* (**4m**).

Yield 70% as a white solid (eluent petroleum ether/ethyl acetate = 15/1). M.p. 134–136 °C. ^1^H NMR (500 MHz, CDCl_3_): *δ* 8.24 (d, *J* = 8.8 Hz, 1H, Ar), 7.86 (d, *J* = 8.9 Hz, 1H, Ar), 7.77 (s, 1H, Ar), 7.42 (d, *J* = 7.8 Hz, 1H, Ar), 7.36 (d, *J* = 7.7 Hz, 1H, Ar), 7.30 (d, *J* = 7.8 Hz, 1H, Ar), 7.11 (d, *J* = 7.8 Hz, 1H, Ar), 6.61 (s, 1H, NH), 4.01 (d, *J* = 16.2 Hz, 1H, CH_2_), 3.86 (s, 3H, OCH_3_), 3.59 (d, *J* = 16.3 Hz, 1H, CH_2_), 2.50 (s, 3H, CH_3_), 1.24 (s, 9H, 3 CH_3_). ^13^C{^1^H} NMR (126 MHz, CDCl_3_) *δ* 166.8, 153.5, 150.4, 145.7, 139.3, 139.2, 135.4, 133.7 (q, *J* = 32.9 Hz, C*_Ar_*-CF_3_), 130.5, 130.3, 130.0, 129.9, 129.8, 124.5, 124.2 (q, *J* = 288.4 Hz, CF_3_), 124.1, 123.9–123.9 (m), 123.3 (q, *J* = 274.2 Hz, CF_3_), 120.1, 118.7 (q, *J* = 320.2 Hz, CF_3_), 80.2, 64.3 (q, *J* = 28.0 Hz, >C<), 53.9, 33.6, 27.9, 21.5. ^19^F NMR (282 MHz, CDCl_3_) *δ* −63.17 (s, 3F, CF_3_), −73.58 (s, 3F, CF_3_), −74.38 (s, 3F, CF_3_). Elemental analysis calcd (%) for C_28_H_25_F_9_N_2_O_7_S: C, 47.73; H, 3.58; N, 3.98; found: C, 47.71; H, 3,57; N, 3.94.

*Methyl 2-(benzyloxycarbonylamino)-3,3,3-trifluoro-2-((4-p-tolyl-6-(trifluoromethyl)-1-(trifluoromethylsulfonyloxy)isoquinolin-3-yl)methyl)propanoate* (**4n**).

Yield 92% as a white solid (eluent petroleum ether/ethyl acetate = 15/1). M.p. 101–103 °C. ^1^H NMR (300 MHz, CDCl_3_): *δ* 8.17 (d, *J* = 8.8 Hz, 1H, Ar), 7.86 (d, *J* = 8.8 Hz, 1H, Ar), 7.65 (s, 1H, Ar), 7.38 (d, *J* = 7.8 Hz, 1H, Ar), 7.33 (d, *J* = 7.8 Hz, 1H, Ar), 7.06–6.98 (m, 4H, Ar), 6.88 (s, 3H, Ar), 6.63 (s, 1H, NH), 4.99 (d, *J* = 12.5 Hz, 1H, OCH_2_), 4.77 (d, *J* = 12.5 Hz, 1H, OCH_2_), 4.14 (d, *J* = 16.8 Hz, 1H, CH_2_), 3.92 (s, 3H, OCH_3_), 3.63 (d, *J* = 16.8 Hz, 1H, CH_2_), 2.49 (s, 3H, CH_3_). ^13^C{^1^H} NMR (126 MHz, CDCl_3_): *δ* 166.6, 153.7, 150.2, 145.1, 139.3, 139.0, 136.6, 135.2, 133.5 (q, *J* = 32.7 Hz, *C_Ar_*-CF_3_), 130.4, 130.2, 129.9, 129.7, 129.6, 127.8, 127.6, 127.4, 124.2, 124.1, 124.0, 123.4 (q, *J* = 273.2 Hz, CF_3_), 124.1 (q, *J* = 288.4 Hz, CF_3_), 119.2, 118.6 (q, *J* = 319.9 Hz, CF_3_), 66.7, 64.4 (q, *J* = 28.6 Hz, >C<), 54.4, 32.9. ^19^F NMR (282 MHz, CDCl_3_): *δ* = −63.17 (s, 3F, CF_3_), −73.91 (s, 3F, CF_3_), −74.98 (s, 3F, CF_3_). Elemental analysis calcd (%) for C_31_H_23_F_9_N_2_O_7_S: C, 50.41; H, 3.14; N, 3.79; found: C, 50.63; H, 3.32; N, 4.01.

*Methyl 2-(benzyloxycarbonylamino)-3,3,3-trifluoro-2-((4-(4-methoxyphenyl)-6-(trifluoromethyl)-1-(trifluoromethylsulfonyloxy)isoquinolin-3-yl)methyl)propanoate* (**4o**).

Yield 93% as a thick oil (eluent petroleum ether/ethyl acetate = 15/1). ^1^H NMR (500 MHz, CDCl_3_): *δ* 8.17 (d, *J* = 8.8 Hz, 1H, Ar), 7.86 (d, *J* = 8.8 Hz, 1H, Ar), 7.67 (s, 1H, Ar), 7.12–7.01 (m, 6H, Ar), 6.87 (s, 3H, Ar), 6.62 (s, 1H, NH), 4.99 (d, *J* = 12.5 Hz, 1H, OCH_2_), 4.77 (d, *J* = 12.5 Hz, 1H, OCH_2_), 4.16 (d, *J* = 16.7 Hz, 1H, CH_2_), 3.93 (s, 6H, 2 CH_3_), 3.64 (d, *J* = 16.7 Hz, 1H, CH_2_). ^13^C{^1^H} NMR (126 MHz, CDCl_3_): *δ* 166.6, 160.1, 153.7, 150.1, 145.4, 139.5, 136.6, 134.9, 133.5 (q, *J* = 33.0 Hz, *C_Ar_*-CF_3_), 131.4, 131.0, 127.9, 127.6, 127.4, 125.4, 124.2, 124.1, 124.0 (q, *J* = 288.7 Hz, CF_3_), 124.0, 123.4 (q, *J* = 273.2 Hz, CF_3_), 119.2, 118.6 (q, *J* = 320.4 Hz, S-CF_3_), 114.8, 114.7, 66.7, 64.4 (q, *J* = 28.5 Hz, >C<), 55.5, 54.4, 32.9. ^19^F NMR (282 MHz, CDCl_3_): *δ −*63.16 (s, 3F, CF_3_), −73.93 (s, 3F, CF_3_), −75.01 (s, 3F, CF_3_). Elemental analysis calcd (%) for C_31_H_23_F_9_N_2_O_8_S: C, 49.34; H, 3.07; N, 3.71; found: C, 49.44; H, 3.02; N, 3.85.

*Methyl 2-(tert-butoxycarbonylamino)-3,3,3-trifluoro-2-((1-(4-methoxyphenyl)-4-p-tolyl-isoquinolin-3-yl)methyl)propanoate* (**5a**).

Yield 92% as a white solid (eluent petroleum ether/ethyl acetate = 10/1). M.p. 214–215 °C. ^1^H NMR (500 MHz, CDCl_3_): *δ* 8.57 (s, 1H, NH), 8.22 (d, *J* = 8.2 Hz, 1H, Ar), 7.77 (d, *J* = 8.1 Hz, 2H, Ar), 7.56–7.52 (m, 2H, Ar), 7.46 (d, *J* = 8.3 Hz, 1H, Ar), 7.35 (d, *J* = 7.7 Hz, 1H, Ar), 7.31 (d, *J* = 7.8 Hz, 1H, Ar), 7.24 (d, *J* = 7.8 Hz, 1H, Ar), 7.10 (d, *J* = 8.0 Hz, 3H, Ar), 3.92 (s, 3H, OCH_3_), 3.71–3.67 (m, 3H, OCH_3_, 1H, CH_2_), 3.49 (d, *J* = 14.7 Hz, 1H, CH_2_), 2.48 (s, 3H, CH_3_), 1.37 (s, 9H, 3 CH_3_). ^13^C{^1^H} NMR (126 MHz, CDCl_3_): *δ* 167.4, 160.4, 158.2, 154.2, 144.7, 137.9, 137.7, 132.9, 131.9, 131.7, 131.6, 130.5, 130.3, 130.2, 129.7, 129.3, 127.7, 126.9, 126.4, 125.2, 124.5 (q, *J* = 288.0 Hz, CF_3_), 115.3, 114.0, 80.0, 67.2, 64.8 (q, *J* = 25.4 Hz, >C<), 55.6, 53.0, 34.2, 28.3, 21.5. ^19^F NMR (282 MHz, CDCl_3_): *δ* −72.96 (s, 3F, CF_3_). Elemental analysis calcd (%) for C_33_H_33_F_3_N_2_O_5_: C, 66.66; H, 5.59; N, 4.71; found: C, 66.35; H, 5.21; N, 4.43.

*Methyl 2-(tert-butoxycarbonylamino)-3,3,3-trifluoro-2-((1-(4-methoxyphenyl)-6-nitro-4-phenylisoquinolin-3-yl)methyl)propanoate* (**5b**).

Yield 88% as a yellow solid (eluent petroleum ether/ethyl acetate = 10/1). M.p. 180–181 °C. ^1^H NMR (500 MHz, CDCl_3_): *δ* 8.38 (d, *J* = 9.2 Hz, 1H, Ar), 8.33 (d, *J* = 1.7 Hz, 1H, Ar), 8.23 (dв, *J* = 9.2, 2.3 Hz, 1H, Ar), 7.73 (d, *J* = 8.3 Hz, 2H, Ar), 7.67 (br. s, 1H, NH), 7.64–7.61 (m, 1H, Ar), 7.58–7.56 (m, 2H, Ar), 7.42 (d, *J* = 7.0 Hz, 1H, Ar), 7.26–7.25 (m, 1H, Ar), 7.13 (d, *J* = 8.3 Hz, 2H, Ar), 3.94 (s, 3H, OCH_3_), 3.82 (d, *J* = 15.3 Hz, 1H, CH_2_), 3.66 (s, 3H, OCH_3_), 3.59 (d, *J* = 15.5 Hz, 1H, CH_2_), 1.29 (s, 9H, 3 CH_3_). ^13^C{^1^H} NMR (126 MHz, CDCl_3_): *δ* 167.3, 160.9, 158.6, 153.7, 148.3, 147.2, 137.1, 134.6, 132.8, 131.7, 130.5, 130.2, 130.0–129.9 (m), 129.5, 129.1, 129.0, 126.7, 124.4 (q, *J* = 287.3 Hz, CF_3_), 122.4, 120.0, 114.2, 100.1, 80.2, 64.7 (q, *J* = 27.7 Hz, >C<), 55.6, 53.4, 34.1, 28.2. ^19^F NMR (282 MHz, CDCl_3_): δ −73.71 (s, 3F, CF_3_). Elemental analysis calcd (%) for C_32_H_30_F_3_N_3_O_7_: C, 61.44; H, 4.83; N, 6.72; found: C, 61.18; H, 4.61; N, 6.56.

*Methyl 2-(benzyloxycarbonylamino)-3,3,3-trifluoro-2-((1-(4-methoxyphenyl)-4-phenyl-6-(trifluoromethyl)isoquinolin-3-yl)methyl)propanoate* (**5c**).

Yield 87% as a white solid (eluent petroleum ether/ethyl acetate = 10/1). M.p. 107–108 °C. ^1^H NMR (400 MHz, CDCl_3_): *δ* 8.30 (d, *J* = 7.8 Hz, 1H, Ar), 7.92 (s, 1H, NH), 7.68–7.66 (m, 2H, Ar), 7.62–7.50 (m, 5H, Ar), 7.22–7.20 (m, 2H, Ar), 7.14–7.05 (m, 5H, Ar), 6.95–6.94 (m, 2H, Ar), 5.07 (d, *J* = 11.9 Hz, 1H, OCH_2_), 4.88 (d, *J* = 12.0 Hz, 1H, OCH_2_), 3.88 (s, 3H, OCH_3_, 1H, CH_2_), 3.65 (s, 3H, OCH_3_), 3.59 (d, *J* = 16.1 Hz, 1H, CH_2_). ^13^C{^1^H} NMR (126 MHz, CDCl_3_): *δ* 167.2, 160.6, 158.5, 154.2, 145.8, 136.7, 136.5, 134.9, 132.2, 131.8 (q, *J* = 32.3 Hz, *C_Ar_*-CF_3_), 131.7, 130.6, 130.5, 130.1, 129.3, 129.1, 128.9, 128.7, 128.3, 127.9, 127.8, 126.0, 125.5, 124.3 (q, *J* = 287.9 Hz, CF_3_), 123.9 (q, *J* = 273.0 Hz, CF_3_), 123.8–123.7 (m), 122.4, 114.0, 66.9, 64.8 (q, *J* = 28.5 Hz, >C<), 55.6, 53.6, 33.8. ^19^F NMR (376 MHz, CDCl_3_): *δ* −62.99 (s, 3F, CF_3_), −73.68 (s, 3F, CF_3_). Elemental analysis calcd (%) for C_36_H_28_F_6_N_2_O_5_: C, 63.34; H, 4.13; N, 4.10; found: C, 63.18; H, 4.01; N, 4.25.

*Methyl 2-(benzyloxycarbonylamino)-3,3,3-trifluoro-2-((4-(phenylethynyl)-1-p-tolylnaphthalen-2-yl)methyl)propanoate* (**6a**).

Yield 58% as a white solid (eluent petroleum ether/ethyl acetate = 10/1). M.p. 180–182 °C. ^1^H NMR (400 MHz, CDCl_3_): *δ* 8.52 (d, *J* = 8.4 Hz, 1H, Ar), 8.03 (s, 1H, NH), 7.70–7.61 (m, 4H, Ar), 7.44–7.40 (m, 4H, Ar), 7.33–7.29 (m, 2H, Ar), 7.16–7.12 (m, 2H, Ar), 7.08–7.02 (m, 5H, Ar), 5.07 (d, *J* = 12.5 Hz, 1H, OCH_2_), 4.96 (d, *J* = 12.8 Hz, 1H, OCH_2_), 3.85 (s, 3H, OCH_3_, 1H, CH_2_,), 3.56 (d, *J* = 15.5 Hz, 1H, CH_2_), 2.47 (s, 3H, CH_3_). ^13^C{^1^H} NMR (126 MHz, CDCl_3_): δ 167.4, 154.3, 145.5, 141.9, 138.1, 136.6, 136.5, 133.1, 132.5, 132.3, 130.7, 130.4, 129.9, 129.6, 129.5, 129.3, 128.7, 128.3, 127.9, 127.8, 127.7, 127.4, 126.9, 126.3, 124.4 (q, *J* = 287.2 Hz, CF_3_), 122.3, 94.0, 86.8, 66.7, 64.9 (q, *J* = 27.8 Hz, >C<), 53.6, 33.9, 21.5. ^19^F NMR (282 MHz, CDCl_3_): δ −73.59 (s, 3F, CF_3_). Elemental analysis calcd (%) for C_37_H_29_F_3_N_2_O_4_: C, 71.37; H, 4.69; N, 4.50; found: C, 71.14; H, 4.48; N, 4.27.

*Methyl 2-(tert-butoxycarbonylamino)-3,3,3-trifluoro-2-((7-nitro-1-phenyl-4-(phenyl-ethynyl)naphthalen-2-yl)methyl)propanoate* (**6b**).

Yield 60% as a white solid (eluent petroleum ether/ethyl acetate = 10/1). M.p. 98–99 °C. ^1^H NMR (500 MHz, CDCl_3_): *δ* 8.68 (d, *J* = 8.0 Hz, 1H, Ar), 8.37 (d, *J* = 7.8 Hz, 1H, Ar), 8.32 (s, 1H, Ar), 7.75 (s, 2H, Ar), 7.62 (s, 2H, Ar), 7.58 (s, 2H, Ar), 7.48 (s, 3H, Ar), 7.41 (s, 1H, Ar), 7.26 (br. s, 1H, NH), 3.89 (m, 3H, OCH_3_, 1H, CH_2_), 3.59 (d, *J* = 14.8 Hz, 1H, CH_2_), 1.30 (s, 9H, 3 CH_3_). ^13^C{^1^H} NMR (126 MHz, CDCl_3_): *δ* 167.4, 153.6, 148.9, 148.2, 145.3, 144.9, 134.3, 134.0, 132.5, 130.5, 130.1, 129.5, 129.4, 129.3, 129.1, 128.9, 128.3, 124.4 (q, *J* = 290.4 Hz, CF_3_), 122.6, 121.7, 121.0, 90.4, 85.9, 66.6, 64.7 (q, *J* = 27.2 Hz, >C<), 60.6, 53.6, 29.8, 28.2. ^19^F NMR (282 MHz, CDCl_3_): *δ* −73.71 (s, 3F, CF_3_). Elemental analysis calcd (%) for C_33_H_28_F_3_N_3_O_6_: C, 63.97; H, 4.56; N, 6.78; found: C, 63.83; H, 4.41; N, 6.54.

*Methyl 2-(tert-butoxycarbonylamino)-3,3,3-trifluoro-2-((4-(phenylethynyl)-1-p-tolyl-7-(trifluoromethyl)naphthalen-2-yl)methyl)propanoate* (**6c**).

Yield 67% as a white solid (eluent petroleum ether/ethyl acetate = 15/1). M.p. 198–200 °C. ^1^H NMR (400 MHz, CDCl_3_): *δ* 8.65 (d, *J* = 8.7 Hz, 1H, Ar), 7.82 (d, *J* = 8.8 Hz, 1H, Ar), 7.77–7.74 (m, 3H, Ar), 7.61 (s, 1H, NH), 7.47–7.46 (m, 3H, Ar), 7.38 (d, *J* = 7.7 Hz, 1H, Ar), 7.33 (d, *J* = 7.6 Hz, 1H, Ar), 7.23 (d, *J* = 8.0 Hz, 1H, Ar), 7.08 (d, *J* = 7.7 Hz, 1H, Ar), 3.84 (s, 3H, OCH_3_, 1H, CH_2_), 3.54 (d, *J* = 15.3 Hz, 1H, CH_2_), 2.49 (s, 3H, CH_3_), 1.32 (s, 9H, 3 CH_3_). ^13^C{^1^H} NMR (126 MHz, CDCl_3_): *δ* 167.5, 153.8, 147.6, 142.1, 138.7, 135.8, 133.7, 132.4, 132.3 (q, *J* = 32.3 Hz, *C_Ar_*-CF_3_), 131.4, 130.3, 130.0, 129.9, 129.8, 129.6, 128.8, 128.6, 128.4, 124.3 (q, *J* = 288.3 Hz, CF_3_), 123.9–123.9 (m), 123.7 (q, *J* = 273.0 Hz, CF_3_), 123.3, 121.9, 94.9, 86.3, 80.1, 64.7 (q, *J* = 27.3 Hz, >C<), 53.4, 34.2, 28.2, 21.5. ^19^F NMR (282 MHz, CDCl_3_): *δ −*62.99 (s, 3F, CF_3_), −73.60 (s, 3F, CF_3_). Elemental analysis calcd (%) for C_35_H_30_F_6_N_2_O_4_: C, 64.02; H, 4.61; N, 4.27; found: C, 64.29; H, 4.41; N, 4.25.

*Methyl 2-(tert-butoxycarbonylamino)-3,3,3-trifluoro-2-((4-phenylisoquinolin-3-yl)methyl)-propanoate* (**7a**).

Yield 50% as a white solid (eluent petroleum ether/ethyl acetate = 5/1). M.p. 124–126 °C. ^1^H NMR (400 MHz, CDCl_3_): *δ* 9.19 (s, 1H, Ar), 8.01–7.99 (m, 1H, Ar), 7.94 (br. s, 1H, NH), 7.59–7.48 (m, 5H, Ar), 7.31 (s, 2H, Ar), 7.19 (m, 1H, Ar), 3.78 (s, 3H, OCH_3_), 3.66 (d, *J* = 15.9 Hz, 1H, CH_2_), 3.47 (d, *J* = 15.2 Hz, 1H, CH_2_), 1.35 (s, 9H, 3 CH_3_). ^13^C{^1^H} NMR (101 MHz, CDCl_3_): *δ* 167.8, 153.9, 150.6, 145.7, 136.2, 135.9, 132.8, 130.8, 130.5, 130.1, 128.9, 128.6, 128.3, 127.6, 127.1, 125.7, 124.5 (q, *J* = 288.4 Hz, CF_3_), 80.1, 66.5, 64.6 (q, *J* = 24.0 Hz, >C<), 53.2, 34.5, 28.2. ^19^F NMR (376 MHz, CDCl_3_): *δ* −72.99 (s, 3F, CF_3_). Elemental analysis calcd (%) for C_25_H_25_F_3_N_2_O_4_: C, 63.28; H, 5.31; N, 5.90; found: C, 63.08; H, 5.01; N, 5.75.

*Methyl 2-(benzyloxycarbonylamino)-3,3,3-trifluoro-2-((4-phenylisoquinolin-3-yl)methyl)-propanoate* (**7b**).

Yield 61% as a white solid (eluent petroleum ether/ethyl acetate = 8/1). M.p. 136–137 °C. ^1^H NMR (400 MHz, CDCl_3_): *δ* 9.13 (s, 1H, Ar), 7.98 (s, 1H, Ar, 1H, NH), 7.2–7.58 (m, 2H, Ar), 7.53–7.48 (m, 3H, Ar), 7.35 (d, *J* = 7.8 Hz, 1H, Ar), 7.23 (d, *J* = 7.6 Hz, 1H, Ar), 7.20–7.10 (m, 6H, Ar), 5.09 (d, *J* = 12.5 Hz, 1H, OCH_2_), 4.94 (d, *J* = 12.5 Hz, 1H, OCH_2_), 3.81 (s, 3H, OCH_3_, 1H, CH_2_), 3.55 (d, *J* = 15.6 Hz, 1H, CH_2_). ^13^C{^1^H} NMR (126 MHz, CDCl_3_): *δ* 167.6, 154.3, 150.4, 145.3, 136.6, 136.2, 135.8, 132.8, 130.7, 130.5, 130.1, 128.9, 128.6, 128.4, 128.2, 127.9, 127.7, 127.2, 127.1, 125.7, 124.4 (q, *J* = 286.9 Hz, CF_3_), 66.7, 64.8 (q, *J* = 28.2 Hz, >C<), 53.5, 34.0. ^19^F NMR (282 MHz, CDCl_3_): *δ* −73.27 (s, 3F, CF_3_). Elemental analysis calcd (%) for C_28_H_23_F_3_N_2_O_4_: C, 66.14; H, 4.56; N, 5.51; found: C, 66.31; H, 4.89; N, 5.76.

*2-(tert-Butoxycarbonylamino)-3,3,3-trifluoro-2-((1-oxo-4-phenyl-1,2-dihydroisoquinolin-3-yl)methyl)propanoic acid* (**8**).

Yield 83% as a white solid. M.p. 176–177 °C. ^1^H NMR (300 MHz, DMSO-*d_6_*): *δ* 10.84 (s, 1H, NH), 8.26 (d, *J* = 7.7 Hz, 1H, Ar), 8.00 (s, 1H, Ar), 7.60 (t, *J* = 7.5 Hz, 1H, Ar), 7.51–7.42 (m, 4H, Ar), 7.25–7.23 (m, 2H, Ar), 6.92 (d, *J* = 8.2 Hz, 1H, Ar), 3.36 (br. s, 1H, OH), 3.10 (d, *J* = 14.8 Hz, 1H, CH_2_), 2.87 (d, *J* = 14.8 Hz, 1H, CH_2_), 1.37 (s, 9H, 3 CH_3_). ^13^C{^1^H} NMR (126 MHz, DMSO-*d_6_*): *δ* 165.3, 161.4, 153.7, 138.2, 134.6, 132.5, 132.2, 132.0, 130.8, 128.7, 128.5, 127.8, 126.6, 126.3, 125.2, 124.7, 124.4 (q, *J* = 287.6 Hz, CF_3_), 118.6, 79.8, 64.0 (q. *J* = 30.8 Hz, >C<), 31.2, 27.9. ^19^F NMR (282 MHz, acetone-*d*_6_): *δ* −74.93 (s, 3F, CF_3_). Elemental analysis calcd (%) for C_24_H_23_F_3_N_2_O_5_: C, 60.50; H, 4.87; N, 5.88; found: C, 63.38; H, 5.03; N, 5.77.

*2-(tert-Butoxycarbonylamino)-3,3,3-trifluoro-2-((1-oxo-4-phenyl-1,2-dihydroisoquinolin-3-yl)methyl)propanoic acid* (**9**).

Yield 85% as a white solid. M.p. 184–185 °C. ^1^H NMR (400 MHz, DMSO-*d*_6_): *δ* 8.43 (d, *J* = 8.3 Hz, 1H, Ar), 7.79 (d, *J* = 8.4 Hz, 1H, Ar), 7.25 (s, 1H, Ar), 7.20 (d, *J* = 7.8 Hz, 2H, Ar), 7.16–7.09 (m, 2H, Ar, 1H, NH), 3.85 (s, 3H, OCH_3_), 3.61 (s, 3H, OCH_3_), 3.31 (s, 2H, NH_2_), 3.06 (d, *J* = 15.0 Hz, 1H, CH_2_), 2.93 (d, *J* = 14.9 Hz, 1H, CH_2_). ^13^C{^1^H} NMR (126 MHz, CDCl_3_): *δ* 166.6, 161.4, 159.7, 139.2, 134.3, 134.1 (q. *J* = 32.4 Hz, *C_Ar_*-CF_3_), 132.2, 131.9, 128.7, 127.5, 126.2, 124.0 (q. *J* = 286.9 Hz, CF_3_), 123.8 (q. *J* = 273.0 Hz, CF_3_), 122.9–122.9 (m), 122.5–122.5 (m), 118.0, 114.8, 114.6, 64.6 (q. *J* = 27.8 Hz, >C<), 55.5, 54.1, 31.2. ^19^F NMR (282 MHz, CDCl_3_): *δ* −62.95 (s, 3F, CF_3_), −79.00 (s, 3F, CF_3_). Elemental analysis calcd (%) for C_22_H_18_F_6_N_2_O_4_: C, 54.10; H, 3.71; N, 5.74; found: C, 54.22; H, 3.99; N, 5.82.

### 3.3. X-ray Structure Determination of ***3a***

A single-crystal X-ray diffraction experiment was carried out with a Bruker SMART APEX II diffractometer (graphite monochromated Mo-K_α_ radiation, *λ* = 0.71073 Å, *ω*-scan technique). The structure was solved with direct methods and refined by the full-matrix least-squares technique against *F^2^*,with the anisotropic thermal parameters for all non-hydrogen atoms using the SHELXL [60] program package. Hydrogen atoms of the NH groups were located in the different Fourier maps and freely refined without constraints. The remaining hydrogen atoms were placed in calculated positions and refined using a riding model with *U_iso_*(H) = 1.5*U_eq_*(C) for hydrogen atoms of methyl groups and *U_iso_*(H) = 1.2*U_eq_*(C) for other carbon atoms. The crystal data and structure refinement details are presented in Supplementary Materials (Table S1). Single-crystal X-ray diffraction analysis was performed using the equipment of the JRC PMR IGIC RAS.

## 4. Conclusions

In conclusion, we have elaborated a convenient pathway to a new series of α-CF_3_-substituted α-amino acid derivatives bearing a pharmacophore isoquinolone core in their backbone. The method is based on [4+2]-annulation of *N-*(pivaloyloxy) aryl amides with orthogonally protected internal acetylene-containing α-amino carboxylates under Rh(III)-catalysis. The reaction smoothly proceeds at an ambient temperature in trifluoroethanol in the presence of 3 mol/% of rhodium dimer complex (Cp*RhCl_2_)_2_ and 1 equiv. of cesium acetate to afford the target products in good yields. The latter compounds proved to be suitable substrates for further conversion to valuable isoquinoline derivatives via a subsequent aromatization/cross-coupling synthetic operation. The biological activity of the obtained compounds is currently being studied.

## Figures and Tables

**Table 1:** Optimization of [4+2]-annulation of aryl hydroxamate **1a** with acetylene **2a**.

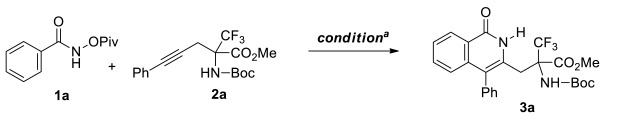				
Entry	Catalyst (mol. %)	Additive (equiv.)	Solvent	Yield ^b^ (%)
1	[Cp*RhCl_2_]_2_ (5)	CsOAc (2)	MeOH	70(60 ^c^)
2	[Cp*RhCl_2_]_2_ (5)	CsOAc (2)	TFE	87(72 ^c^)
3	[Cp*RhCl_2_]_2_ (5)	CsOAc (2)	toluene	65
4	[Cp*RhCl_2_]_2_ (5)	NaOAc (2)	MeOH	73
5	[Cp*RhCl_2_]_2_ (5)	KOAc (2)	MeOH	69
6	[Cp*RhCl_2_]_2_ (3)	CsOAc (2)	MeOH	73
7	[Cp*RhCl_2_]_2_ (3)	CsOAc (2)	TFE	80
8	[Cp*RhCl_2_]_2_ (3)	**CsOAc (1)**	**TFE**	**84(71 ^c^)**
9	[Cp*RhCl_2_]_2_ (3)	CsOAc (1)	MeOH	68
10	[Cp*IrCl_2_]_2_ (5)	CsOAc (2)	MeOH	NR
11	[Cp*CoI_2_]_2_ (5)	CsOAc (2)	MeOH	NR
12	[(*p*-cymene)RuCl_2_]_2_ (5)	CsOAc (2)	MeOH	NR
13	-	CsOAc (2)	MeOH	NR
14	[Cp*RhCl_2_]_2_ (5)	-	MeOH	NR

^a^ Reagents and conditions: aryl hydroxamate **1a** (0.2 mmol), acetylene **2a** (0.2 mmol), solvent (2 mL), r.t.; ^b^ Determined by ^19^F NMR spectroscopy; ^c^ Isolated yield.

## Data Availability

Data are contained within the article and Supplementary Materials.

## Supplementary Materials

The following supporting information can be downloaded at: https://www.mdpi.com/article/10.3390/molecules27238488/s1, Figures S1–S92. ^1^H and ^13^C NMR spectra of compounds. Figure S93. H-bonded dimer in the crystal of **3a**. Scheme S1. Proposed mechanism. Table S1. Crystal data, data collection and structure refinement parameters for **3a**.

## Sample Availability

Samples of all of the compounds are available from the authors.

## References

[1] Chen H.-Y., He L.-J., Li S.-Q., Zhang Y.-J., Huang J.-H., Qin H.-X., Wang J.-L., Li Q.-Y., Yang D.-L. (2019). A derivate of benzimidazole-isoquinolinone induces SKP2 transcriptional inhibition to exert anti-tumor activity in glioblastoma cells. Molecules.

[2] He L.-J., Yang D.-L., Li S.-Q., Zhang Y.-J., Tang Y., Lei J., Frett B., Lin H.-K., Li H.-Y., Chen Z.-Z. (2018). Facile construction of fused benzimidazole-isoquinolinones that induce cell-cycle arrest and apoptosis in colorectal cancer cells. Bioorg. Med. Chem..

[3] Tang Z., Niu S., Liu F., Lao K., Miao J., Ji J., Wang X., Yan M., Zhang L., You Q. (2014). Synthesis and biological evaluation of 2,3-diaryl isoquinolinone derivatives as anti-breast cancer agents targeting ERα and VEGFR-2. Bioorg. Med. Chem. Lett..

[4] Scalia M., Satriano C., Greca R., Stella A.M.G., Rizzarelli E., Spina-Purrello V. (2013). PARP-1 inhibitors DPQ and PJ-34 negatively modulate proinflammatory commitment of human glioblastoma cells. Neurochem. Res..

[5] Zhang Z., You Z., Dobrowsky R.T., Rick T., Blagg B.S.J. (2018). Synthesis and evaluation of a ring-constrained Hsp90 C-terminal inhibitor that exhibits neuroprotective activity. Bioorg. Med. Chem. Lett..

[6] Nair J.J., Rarova L., Strnad M., Bastida J., van Staden J. (2012). Apoptosis-inducing effects of distichamine and narciprimine, rare alkaloids of the plant family Amaryllidaceae. Bioorg. Med. Chem. Lett..

[7] Du J., Xi L., Lei B., Liu H., Yao X. (2011). Structural requirements of isoquinolones as novel selective c-Jun N-terminal kinase 1 inhibitors: 2D and 3D QSAR analyses. Chem. Biol. Drug Des..

[8] Guo S., Wang F., Sun L., Zhang X., Fan X. (2018). Palladium-catalyzed oxidative cyclocarbonylation of isoquinolones with CO via C-H/N-H bond cleavage: Easy access to isoindolo[2,1-b]isoquinoline-5,7-dione derivatives. Adv. Synth. Catal..

[9] Thirupataiah B., Reddy G.S., Ghule S.S., Kumar J.S., Mounika G., Hossain K.A., Mudgal J., Mathew J.E., Shenoy G.G., Parsa K.V.L. (2020). Synthesis of 11,12-dihydro benzo[c]phenanthridines via a Pd-catalyzed unusual construction of isocoumarin ring/FeCl3-mediated intramolecular arene-allyl cyclization: First identification of a benzo[c]phenanthridine based PDE4 inhibitor. Bioorg. Chem..

[10] Guo S., Sun L., Wang F., Zhang X., Fan X. (2018). Rh(III)-Catalyzed oxidative annulation of isoquinolones with diazoketoesters featuring an in situ deacylation: Synthesis of isoindoloisoquinolones and their transformation to Rosettacin analogues. J. Org. Chem..

[11] Coles-Taylor B.L., McCallum S.M., Scott Lee J., Michel B.W. (2018). Accessing 4-oxy-substituted isoquinolinones via C-H activation and regioselective migratory insertion with electronically biased ynol ethers. Org. Biomol. Chem..

[12] Wu J.-Q., Zhang S.-S., Gao H., Qi Z., Zhou C.-J., Ji W.-W., Liu Y., Chen Y., Li Q., Li X. (2017). Experimental and theoretical studies on rhodium-catalyzed coupling of benzamides with 2,2-difluorovinyl tosylate: Diverse synthesis of fluorinated heterocycles. J. Am. Chem. Soc..

[13] Salvati E., Botta L., Amato J., Saverio Di Leva F., Zizza P., Gioiello A., Pagano B., Graziani G., Tarsounas M., Randazzo A. (2017). Lead discovery of dual G-quadruplex stabilizers and poly(ADP-ribose) polymerases (PARPs) inhibitors: A new avenue in anticancer treatment. J. Med. Chem..

[14] Wu Y., Sun P., Zhang K., Yang T., Yao H., Lin A. (2016). Rh(III)-Catalyzed redox-neutral annulation of primary benzamides with diazo compounds: Approach to isoquinolinones. J. Org. Chem..

[15] Newton C.G., Wang S.-G., Oliveira C.C., Cramer N. (2017). Catalytic enantioselective transformations involving C-H bond cleavage by transition-metal complexes. Chem. Rev..

[16] Park Y., Kim Y., Chang S. (2017). Transition metal-catalyzed C-H amination: Scope, mechanism, and applications. Chem. Rev..

[17] Chen J.-R., Hu X.-Q., Lu L.-Q., Xiao W.-J. (2015). Formal [4+1] annulation reactions in the synthesis of carbocyclic and heterocyclic systems. Chem. Rev..

[18] Ackermann L. (2014). Carboxylate-assisted ruthenium-catalyzed alkyne annulations by C-H/Het-H bond functionalizations. Acc. Chem. Res..

[19] Upadhyay N.S., Thorat V.H., Sato R., Annamalai P., Chuang S.-C., Cheng C.-H. (2017). Synthesis of isoquinolones via Rh-catalyzed C-H activation of substituted benzamides using air as the sole oxidant in water. Green Chem..

[20] Reddy Chidipudi S., Burns D.J., Khan I., Lam H.W. (2015). Enantioselective synthesis of spiroindenes by enol-directed rhodium(III)-catalyzed C-H functionalization and spiroannulation. Angew. Chem. Int. Ed..

[21] Zhang X., Si W., Bao M., Asao N., Yamamoto Y., Jin T. (2014). Rh(III)-Catalyzed regioselective functionalization of C-H bonds of naphthylcarbamates for oxidative annulation with alkynes. Org. Lett..

[22] Chuang S.-C., Gandeepan P., Cheng C.-H. (2013). Synthesis of isoquinolines via Rh(III)-catalyzed C-H activation using hydrazone as a new oxidizing directing group. Org. Lett..

[23] Pham M.V., Ye B., Cramer N. (2012). Access to sultams by rhodium(III)-catalyzed directed C-H activation. Angew. Chem. Int. Ed..

[24] Hyster T.K., Rovis T. (2010). Rhodium-catalyzed oxidative cycloaddition of benzamides and alkynes via C-H/N-H Activation. J. Am. Chem. Soc..

[25] Song G., Chen D., Pan C.-L., Crabtree R.H., Li X. (2010). Rh-Catalyzed oxidative coupling between primary and secondary benzamides and alkynes: Synthesis of polycyclic amides. J. Org. Chem..

[26] Guimond N., Gouliaras C., Fagnou K. (2010). Rhodium(III)-Catalyzed isoquinolone synthesis: The N-O bond as a handle for C-N bond formation and catalyst turnover. J. Am. Chem. Soc..

[27] Guimond N., Gorelsky S.I., Fagnou K. (2011). Rhodium(III)-Catalyzed heterocycle synthesis using an internal oxidant: Improved reactivity and mechanistic studies. J. Am. Chem. Soc..

[28] Mochida S., Umeda N., Hirano K., Satoh T., Miura M. (2010). Rhodium-catalyzed oxidative coupling/cyclization of benzamides with alkynes via C-H bond cleavage. Chem. Lett..

[29] Xu X., Liu Y., Park C.-M. (2012). Rhodium(III)-Catalyzed intramolecular annulation through C-H activation: Total synthesis of (±)-antofine, (±)-septicine, (±)-tylophorine, and rosettacin. Angew. Chem. Int. Ed..

[30] Wang H., Grohmann C., Nimphius C., Glorius F. (2012). Mild Rh(III)-Catalyzed C-H activation and annulation with alkyne MIDA Boronates: Short, efficient synthesis of heterocyclic boronic acid derivatives. J. Am. Chem. Soc..

[31] Huckins J.R., Bercot E.A., Thiel O.R., Hwang T.-L., Bio M.M. (2013). Rh(III)-Catalyzed C-H activation and double directing group strategy for the regioselective synthesis of naphthyridinones. J. Am. Chem. Soc..

[32] Yu D.-G., de Azambuja F., Glorius F. (2014). α-MsO/TsO/Cl ketones as oxidized alkyne equivalents: Redox-neutral rhodium(III)-catalyzed C-H activation for the synthesis of N-heterocycles. Angew. Chem. Int. Ed..

[33] Wang F., Qi Z., Zhao Y., Zhai S., Zheng G., Mi R., Huang Z., Zhu X., He X., Li X. (2020). Rhodium(III)-catalyzed atroposelective synthesis of biaryls by C-H activation and intermolecular coupling with sterically hindered alkynes. Angew. Chem. Int. Ed..

[34] Chen J., Zhang L., Zheng X., Zhou J., Zhong T., Yu C. (2020). Synthesis of isoquinolinone derivatives by Rh(III)-catalyzed C-H functionalization of N-ethoxybenzamides. Synthetic Commun..

[35] Ohata J., Martin S.C., Ball Z.T. (2019). Metal-mediated functionalization of natural peptides and proteins: Panning for bioconjugation gold. Angew. Chem. Int. Ed..

[36] Zhang C., Vinogradova E.V., Spokoyny A.M., Buchwald S.L., Pentelute B.L. (2019). Arylation chemistry for bioconjugation. Angew. Chem. Int. Ed..

[37] Rodríguez J., Martínez-Calvo M. (2020). Transition-metal-mediated modification of biomolecules. Chem. Eur. J..

[38] Haase C., Seitz O. (2006). Chemical Synthesis of Glycopeptides. Top. Curr. Chem..

[39] Wang Y., Cheetham A.G., Angacian G., Su H., Xie L., Cui H. (2017). Peptide-drug conjugates as effective prodrug strategies for targeted delivery. Adv. Drug Deliv. Rev..

[40] Sunna A., Care A. (2017). Peptides and Peptide-based Biomaterials and their Biomedical Applications.

[41] Hoppenz P., Els-Heindl S., Beck-Sickinger A.G. (2020). Peptide-drug conjugates and their targets in advanced cancer therapies. Front. Chem..

[42] Ojima I. (2009). Fluorine in Medicinal Chemistry and Chemical Biology.

[43] Yoder N.C., Kumar K. (2002). Fluorinated amino acids in protein design and engineering. Chem. Soc. Rev..

[44] Jaeckel C., Koksch B. (2005). Fluorine in peptide design and protein engineering. Eur. J. Org. Chem..

[45] Salwiczek M., Nyakatura E.K., Gerling U.I., Ye S., Koksch B. (2012). Fluorinated amino acids: Compatibility with native protein structures and effects on protein–protein interactions. Chem. Soc. Rev..

[46] Marsh E.N.G. (2014). Fluorinated proteins: From design and synthesis to structure and stability. Acc. Chem. Res..

[47] Berger A.A., Völler J., Budisa N., Koksch B. (2017). Deciphering the fluorine code—The many hats fluorine wears in a protein environment. Acc. Chem. Res..

[48] Huhmann S., Koksch B. (2018). Fine-tuning the proteolytic stability of peptides with fluorinated amino acids. Eur. J. Org. Chem..

[49] Moschner J., Stulberg V., Fernandes R., Huhmann S., Leppkes J., Koksch B. (2019). Approaches to obtaining fluorinated α-amino acids. Chem. Rev..

[50] Smits R., Cadicamo C.D., Burger K., Koksch B. (2008). Synthetic strategies to α-trifluoromethyl and α-difluoromethyl substituted α-amino acids. Chem. Soc. Rev..

[51] Vorobyeva D.V., Petropavlovskikh D.A., Godovikov I.A., Nefedov S.E., Osipov S.N. (2021). Rh(III)-Catalyzed C-H activation/annulation of aryl hydroxamates with CF_3_-containing α-propargyl α-amino acid derivatives. Eur. J. Org. Chem..

[52] Iagafarova I.E., Vorobyeva D.V., Peregudov A.S., Osipov S.N. (2015). CF_3_-Carbenoid C-H functionalization of (hetero)arenes under chelation-controlled RhIII catalysis. Eur. J. Org. Chem..

[53] Iagafarova I.E., Vorobyeva D.V., Loginov D.A., Peregudov A.S., Osipov S.N. (2017). Rh(III)-catalyzed CF3-carbenoid C-7 functionalization of indolines. Eur. J. Org. Chem..

[54] Vorobyeva D.V., Vinogradov M.M., Nelyubina Y.V., Loginov D.A., Peregudov A.S., Osipov S.N. (2018). Rhodium(III)-catalyzed CF3-carbenoid C-H functionalization of 6-arylpurines. Org. Biomol. Chem..

[55] Petropavlovskikh D.A., Vorobyeva D.V., Godovikov I.A., Nefedov S.E., Filippov O.A., Osipov S.N. (2021). Lossen rearrangement by Rh(III)-catalyzed C-H activation/annulation of aryl hydroxamates with alkynes: Access to quinolone-containing amino acid derivatives. Org. Biomol. Chem..

[56] Shchetnikov G.T., Zotova M.A., Bruneau C., Dixneuf P.H., Osipov S.N. (2010). Synthesis of α-Alkynyl-β,β,β-trifluoroalanine Derivatives by Sonogashira Cross-Coupling Reaction. Eur. J. Org. Chem..

[57] Ullyot G.E. (1952). Basic ether-substituted isoquinolines. US Patent.

[58] Merck G. (1848). Vorläufige Notiz über eine neue organische Base im Opium. Liebigs Ann. Chem..

[59] Graulich A., Scuvée-Moreau J., Seutin V., Liégeois J.-F. (2005). Orthogonal Synthesis of Indolines and Isoquinolines via Aryne Annulation. J. Med. Chem..

[60] Sheldrick G.M. (2015). Crystal Structure Refinement with SHELXL. Acta Cryst..

