# Supplementary material for: Synthesis of Functionalized Isoquinolone Derivatives via Rh(III)-Catalyzed [4+2]-Annulation of Benzamides with Internal Acetylene-Containing α-CF3-α-Amino Carboxylates
Source: Molecules. 2022 Dec 2;27(23):8488. doi: 10.3390/molecules27238488 (PMC9736582; doi:10.3390/molecules27238488)
Supplement: Supplementary file 1 [file molecules-27-08488-s001.zip › molecules-2042301-supplementary.pdf]

Table of contents

1. ^{1}H and ^{13}C NMR Spectra of Compounds S2-S92
2. Proposed mechanism S93
3. X-ray Diffraction Study S94

^{1}H NMR and ^{13}C NMR Spectra.

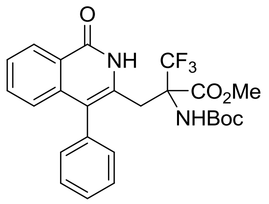

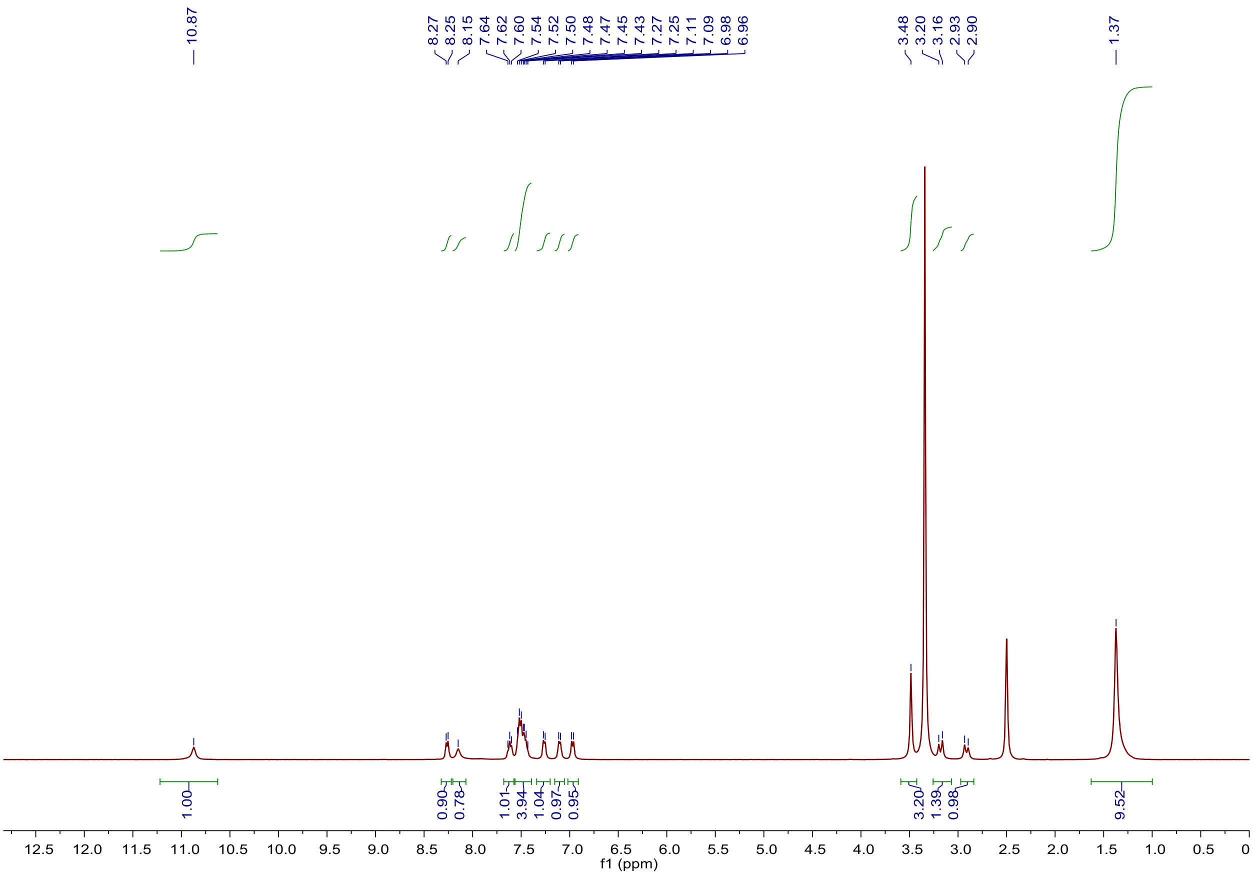

Figure S1. ^{1}H spectrum of **3a** in DMSO-d_6

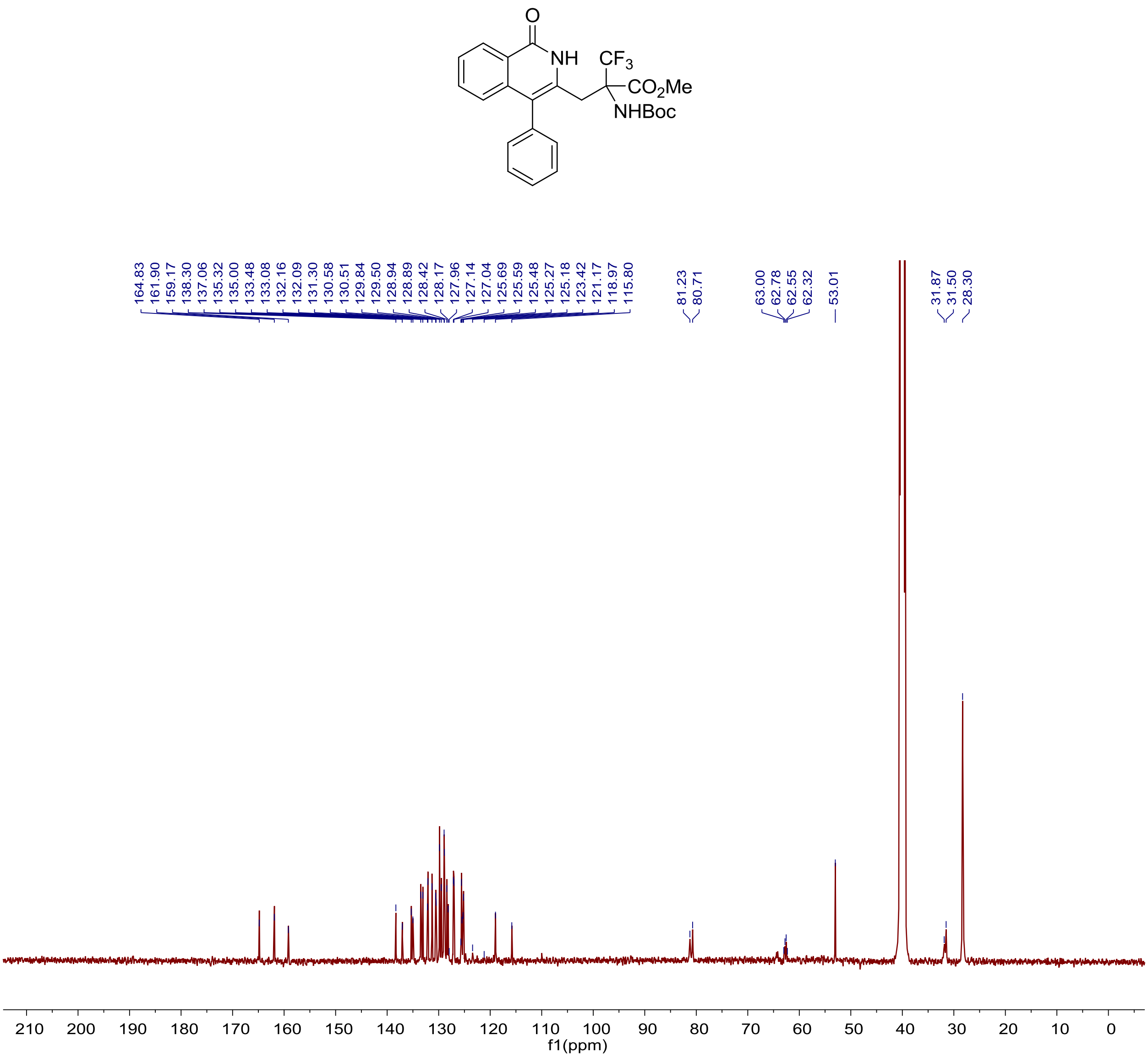

Figure S2. ^{13}C spectrum of **3a** in DMSO-d_6

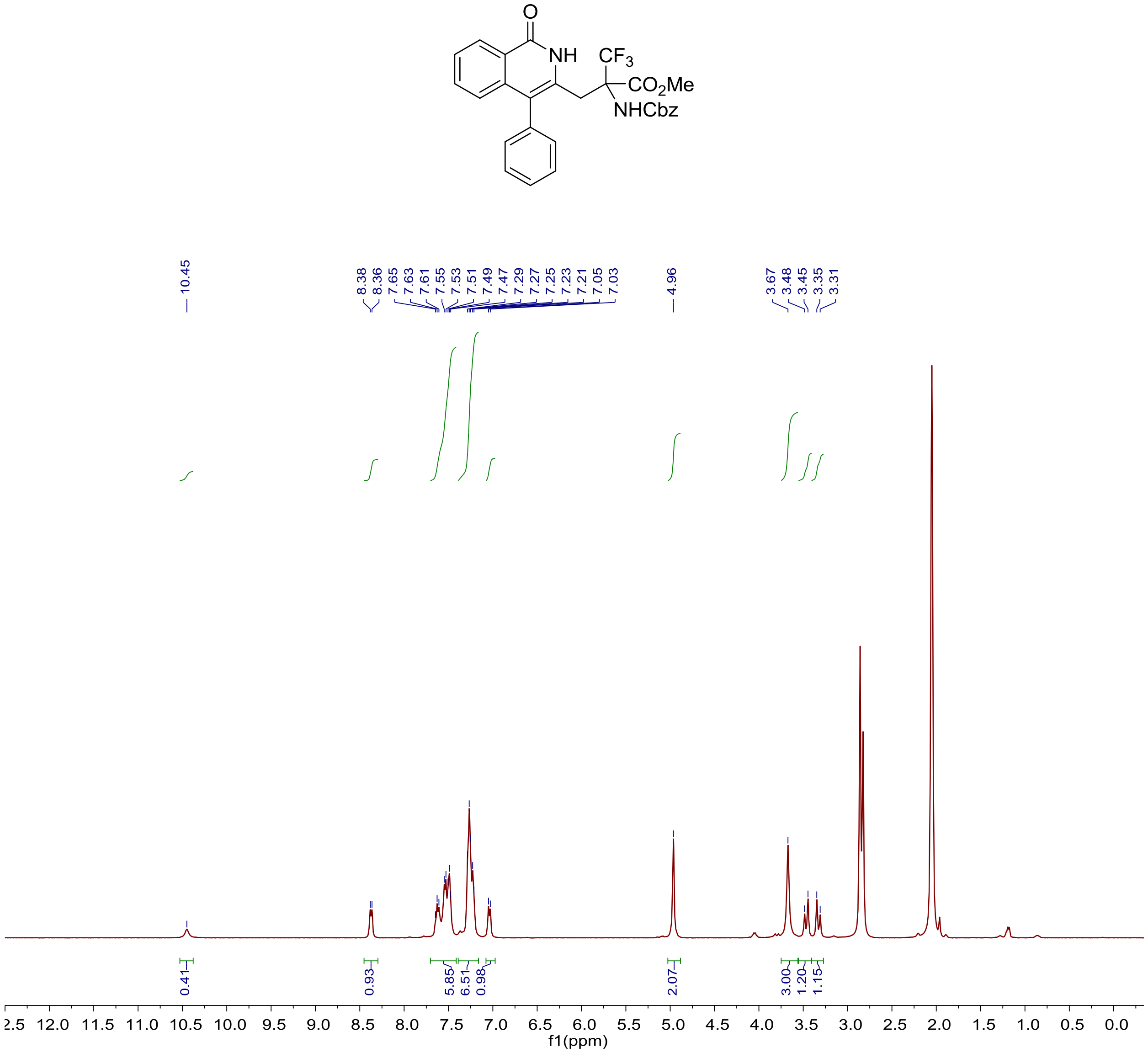

Figure S3. ^{1}H spectrum of **3b** in acetone-d_6

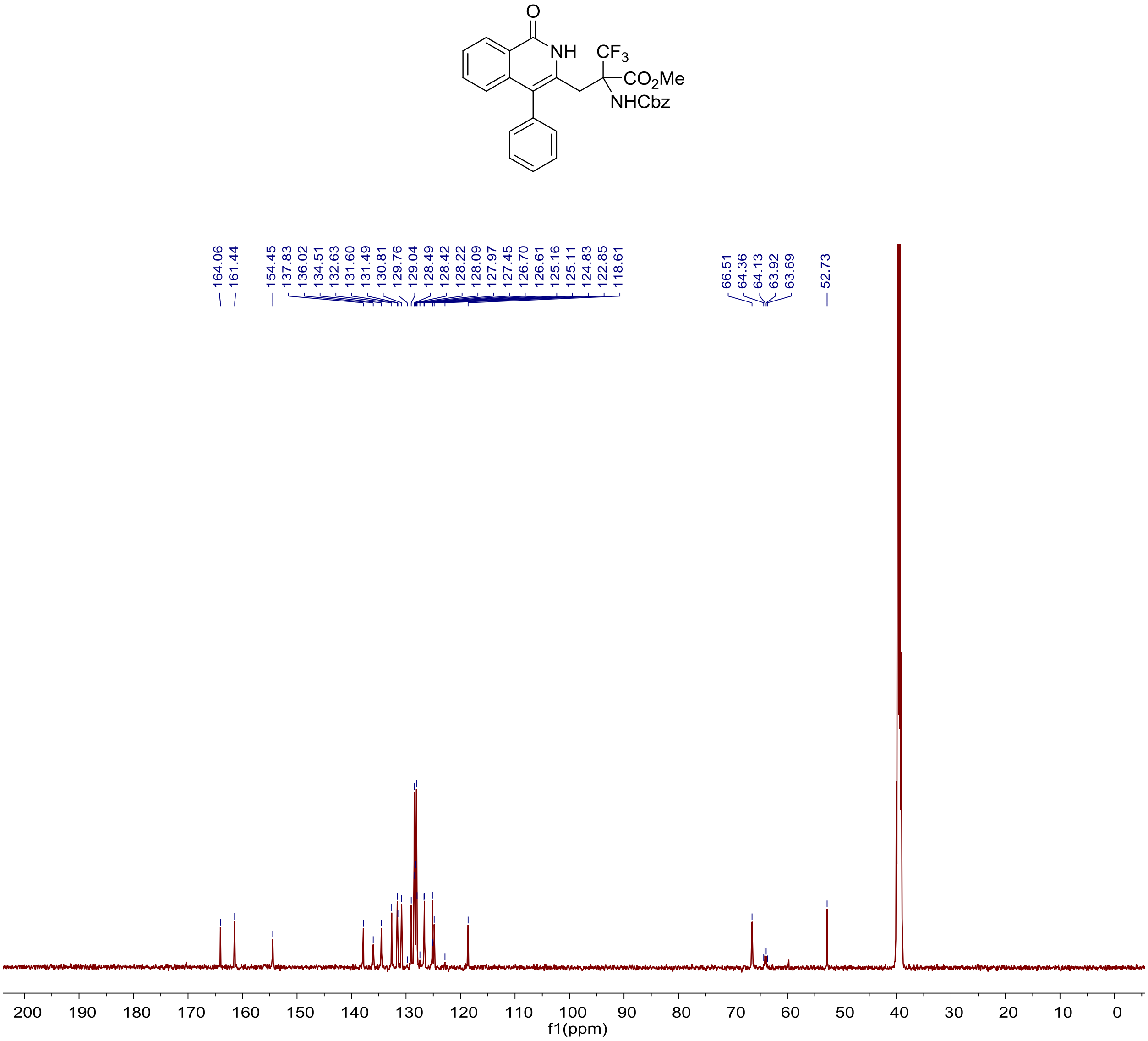

Figure S4. ^{13}C spectrum of **3b** in DMSO-d_6

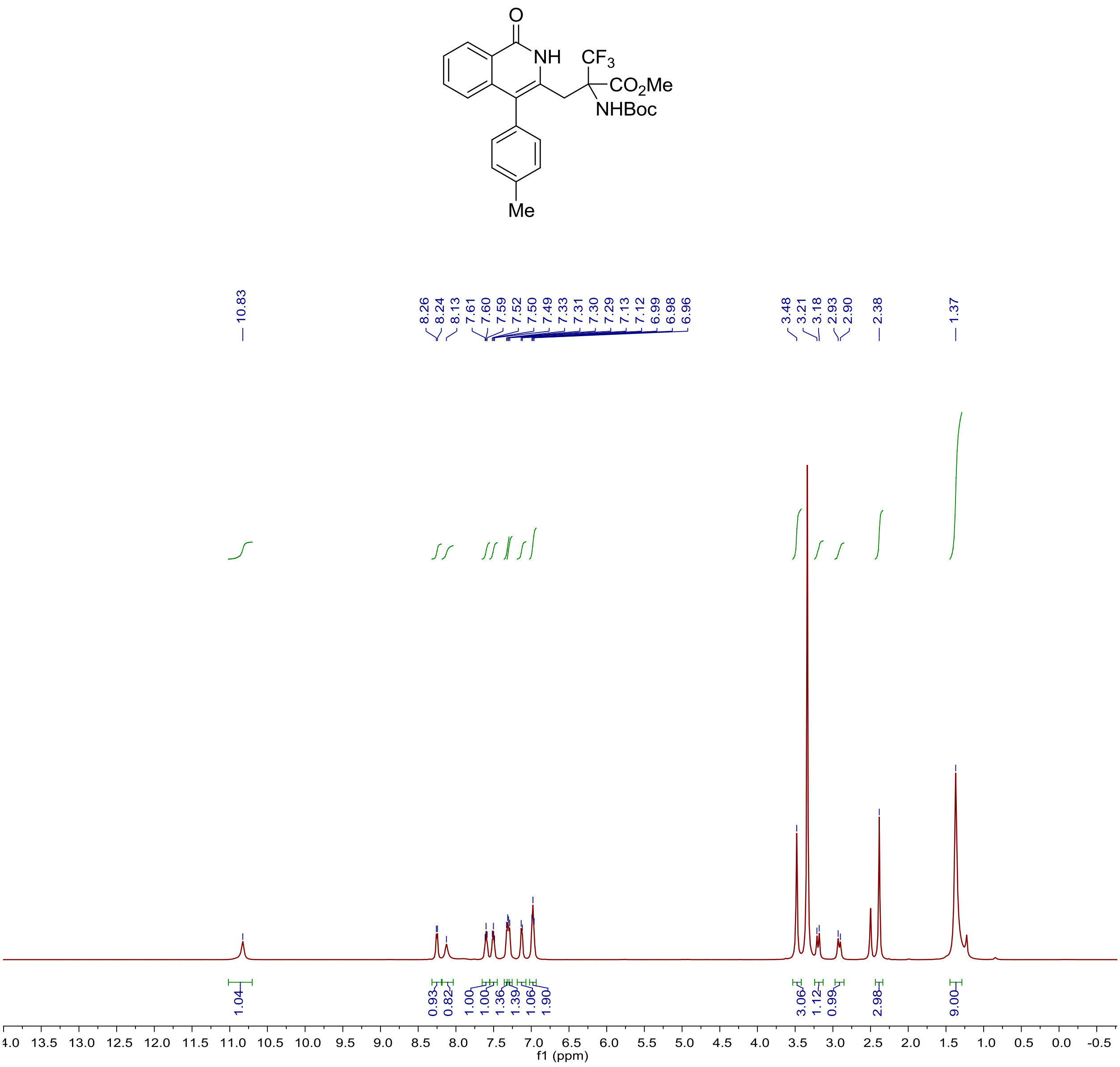

Figure S5. ^{1}H spectrum of **3c** in DMSO-d_6

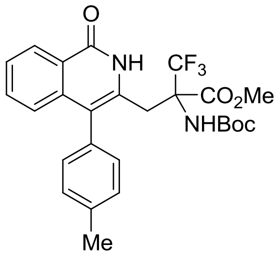

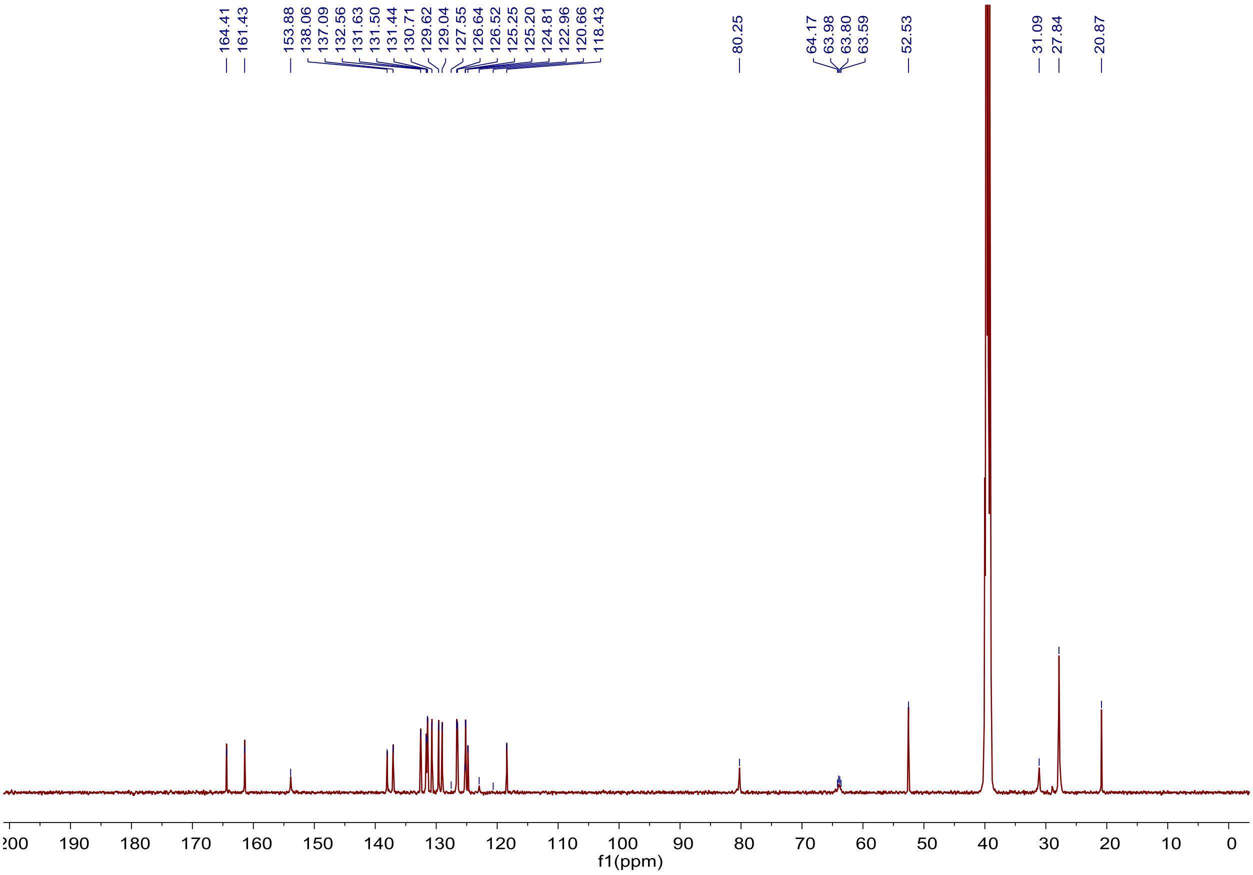

Figure S6. ^{13}C spectrum of **3c** in DMSO-d_6

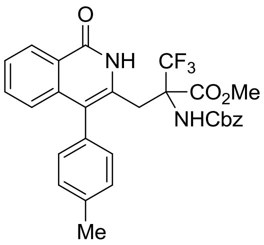

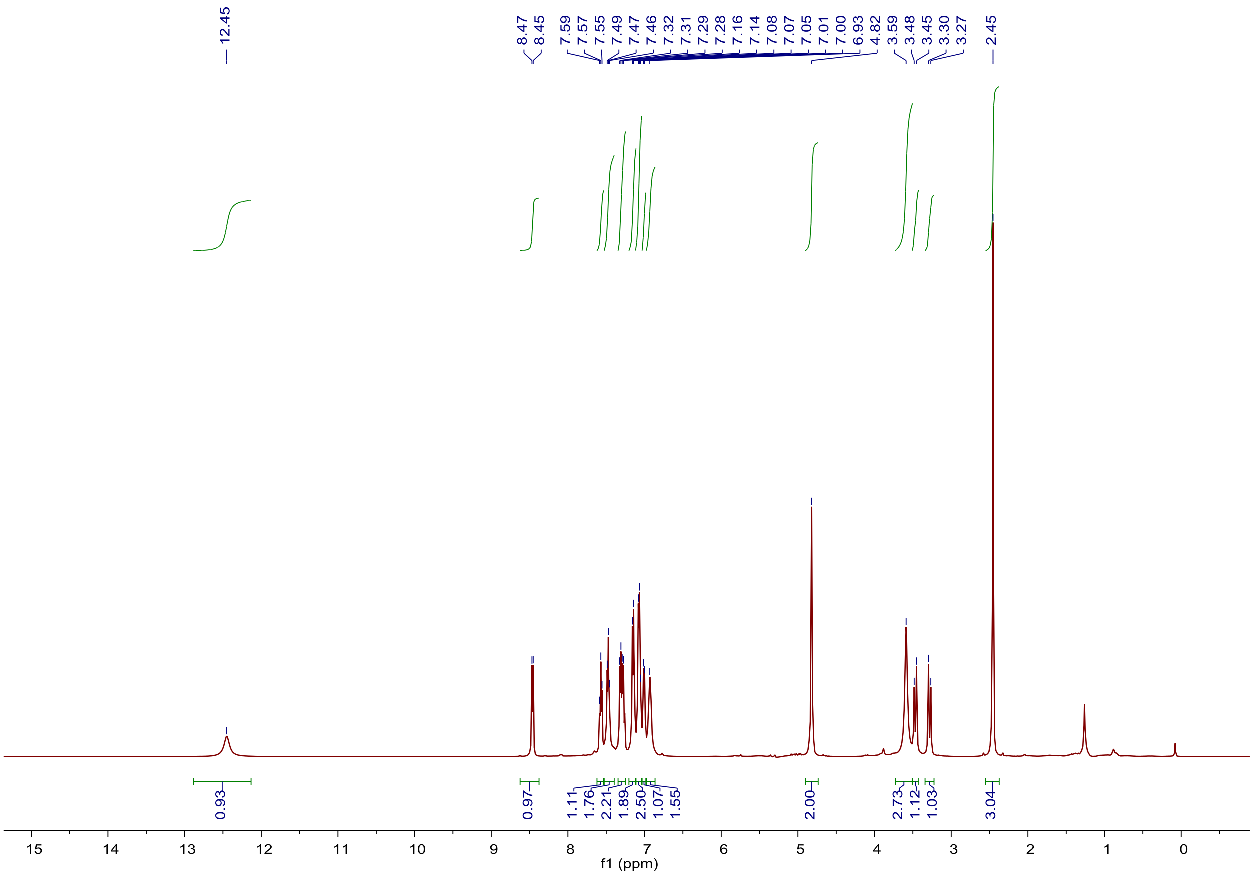

Figure S7. ^{1}H spectrum of **3d** in $CDCl_3$

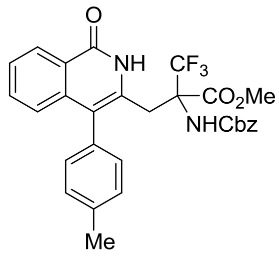

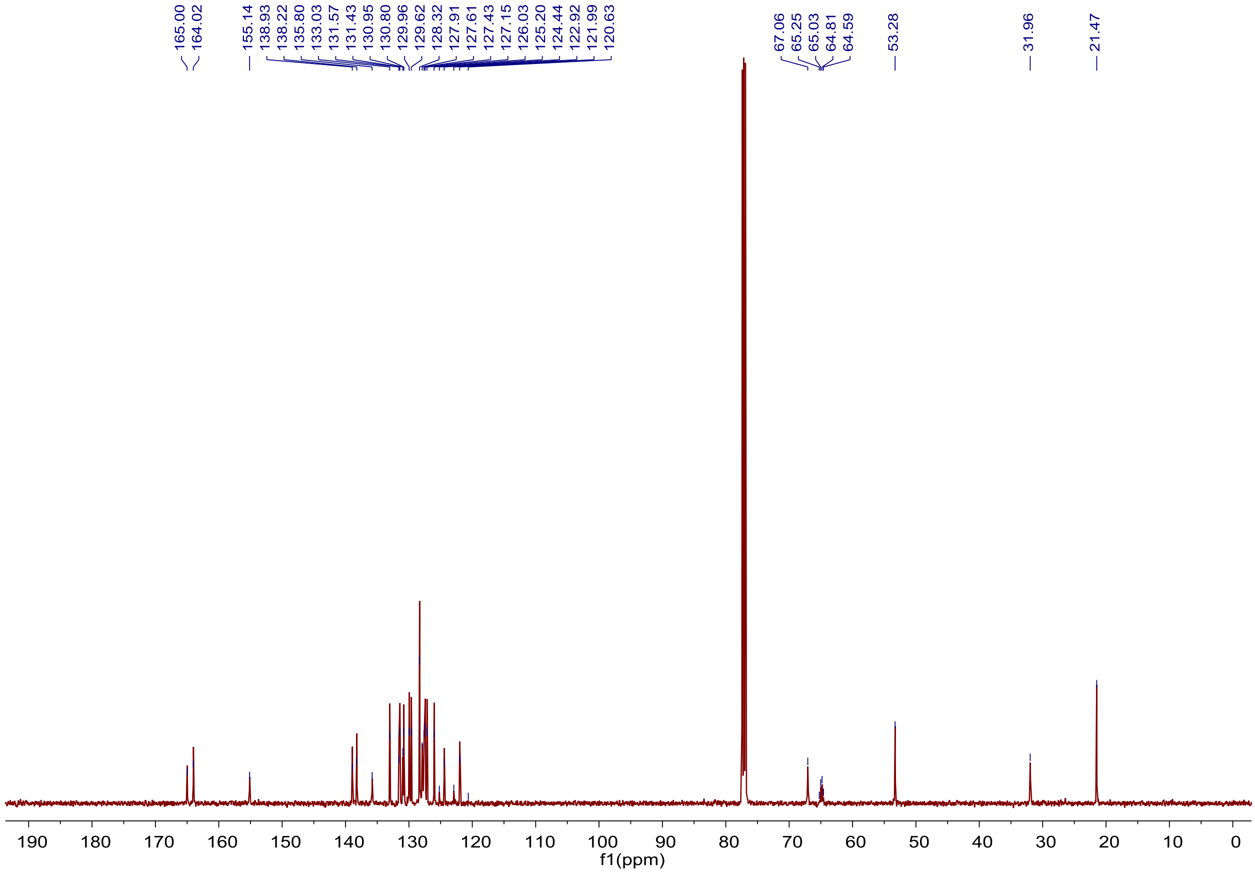

Figure S8. ^{13}C spectrum of **3d** in $CDCl_3$

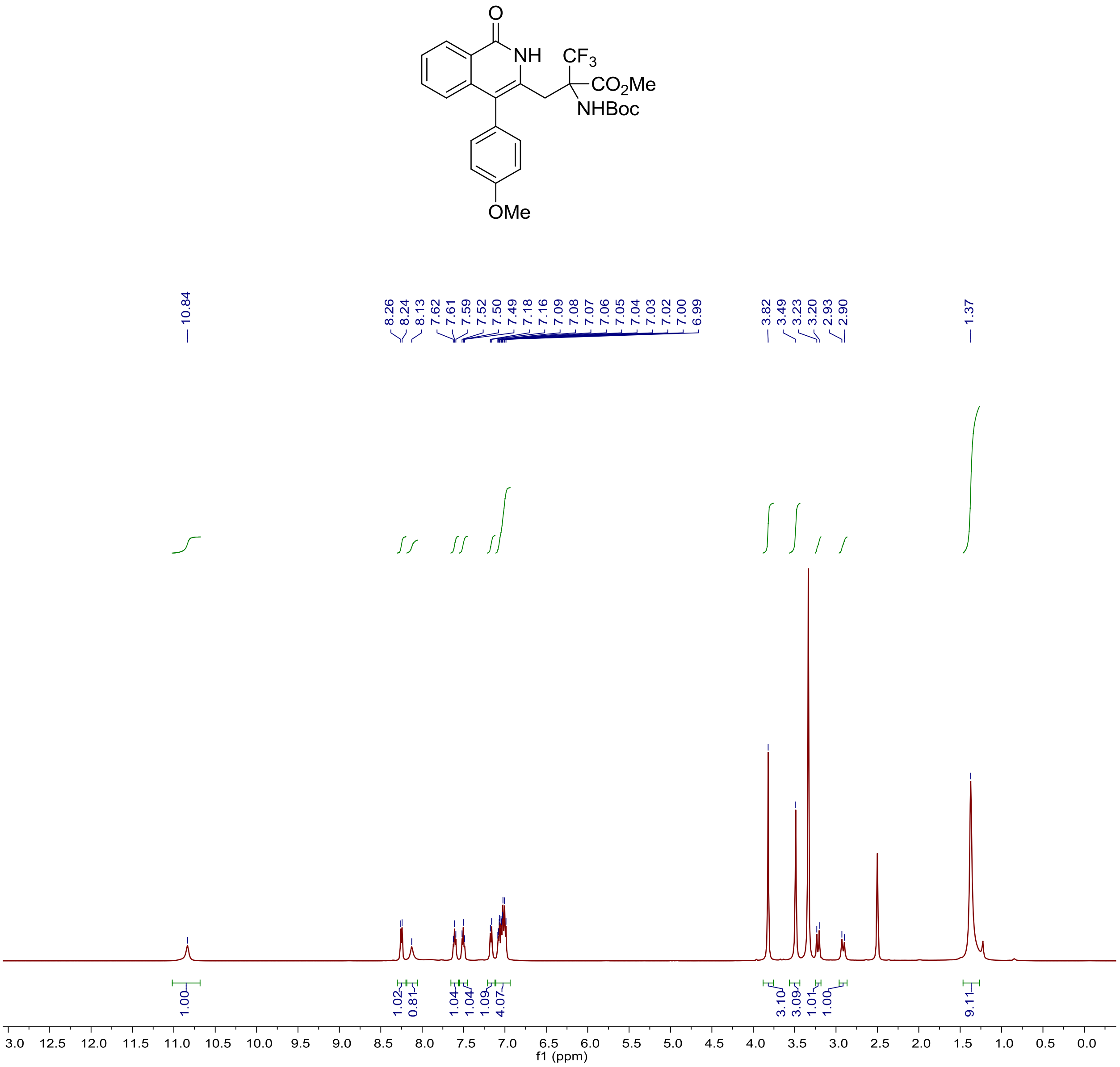

Figure S9. ^{1}H spectrum of **3e** in DMSO-d_6

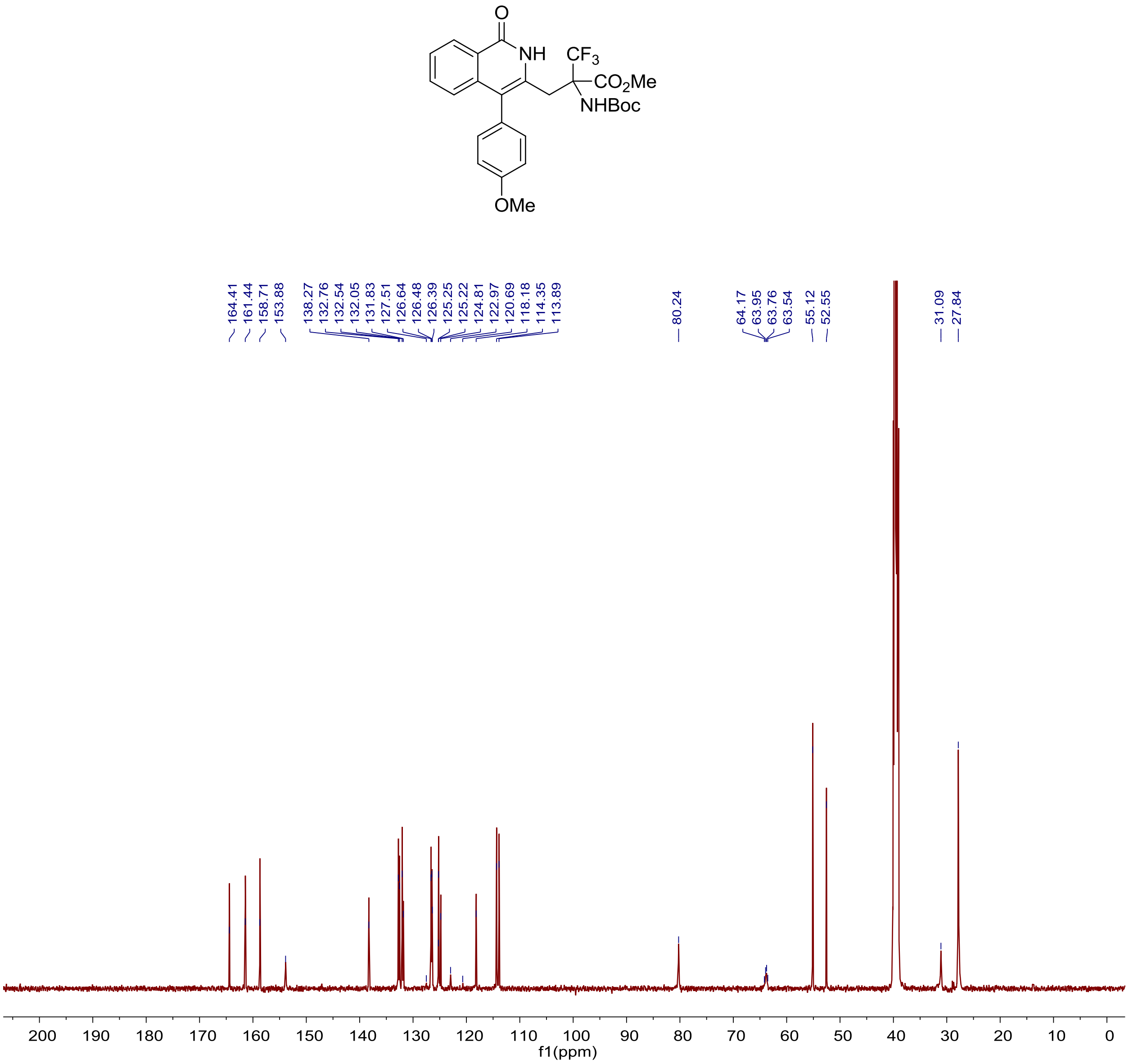

Figure S10. ^{13}C spectrum of **3e** in DMSO-d_6

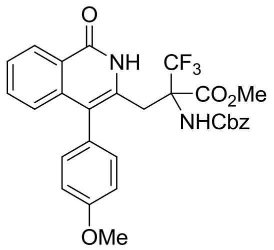

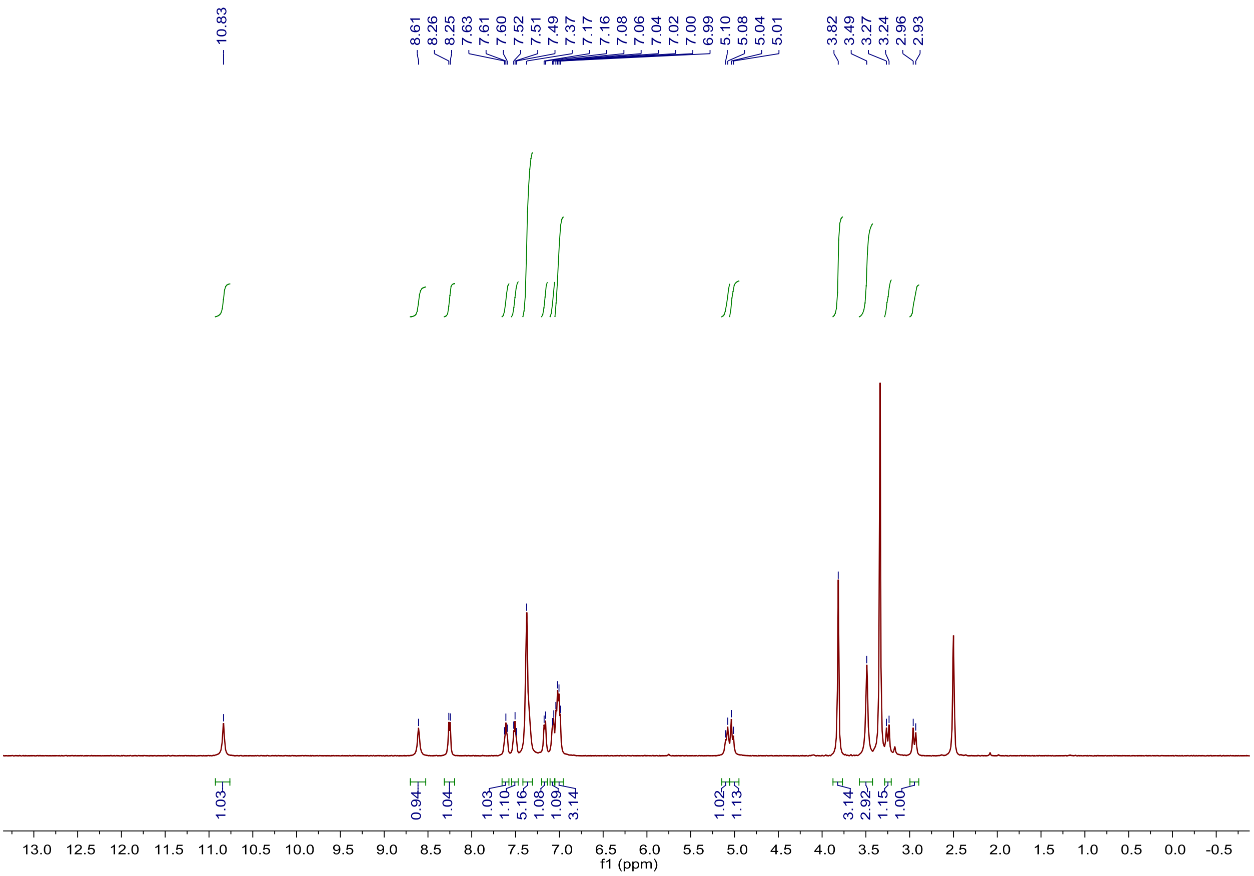

Figure S11. ^{1}H spectrum of **3f** in DMSO-d_6

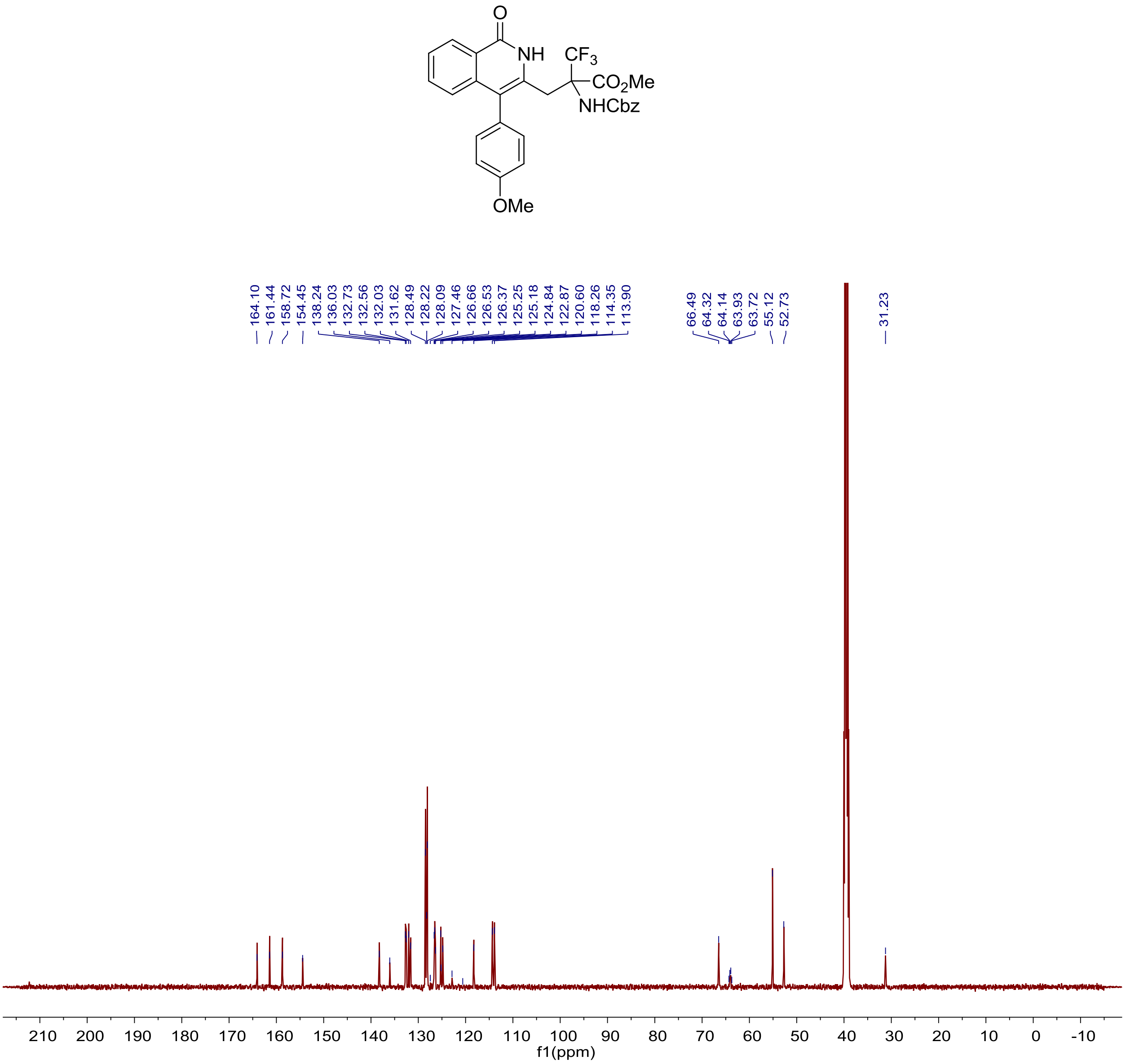

Figure S12. ^{13}C spectrum of **3f** in DMSO-d_6

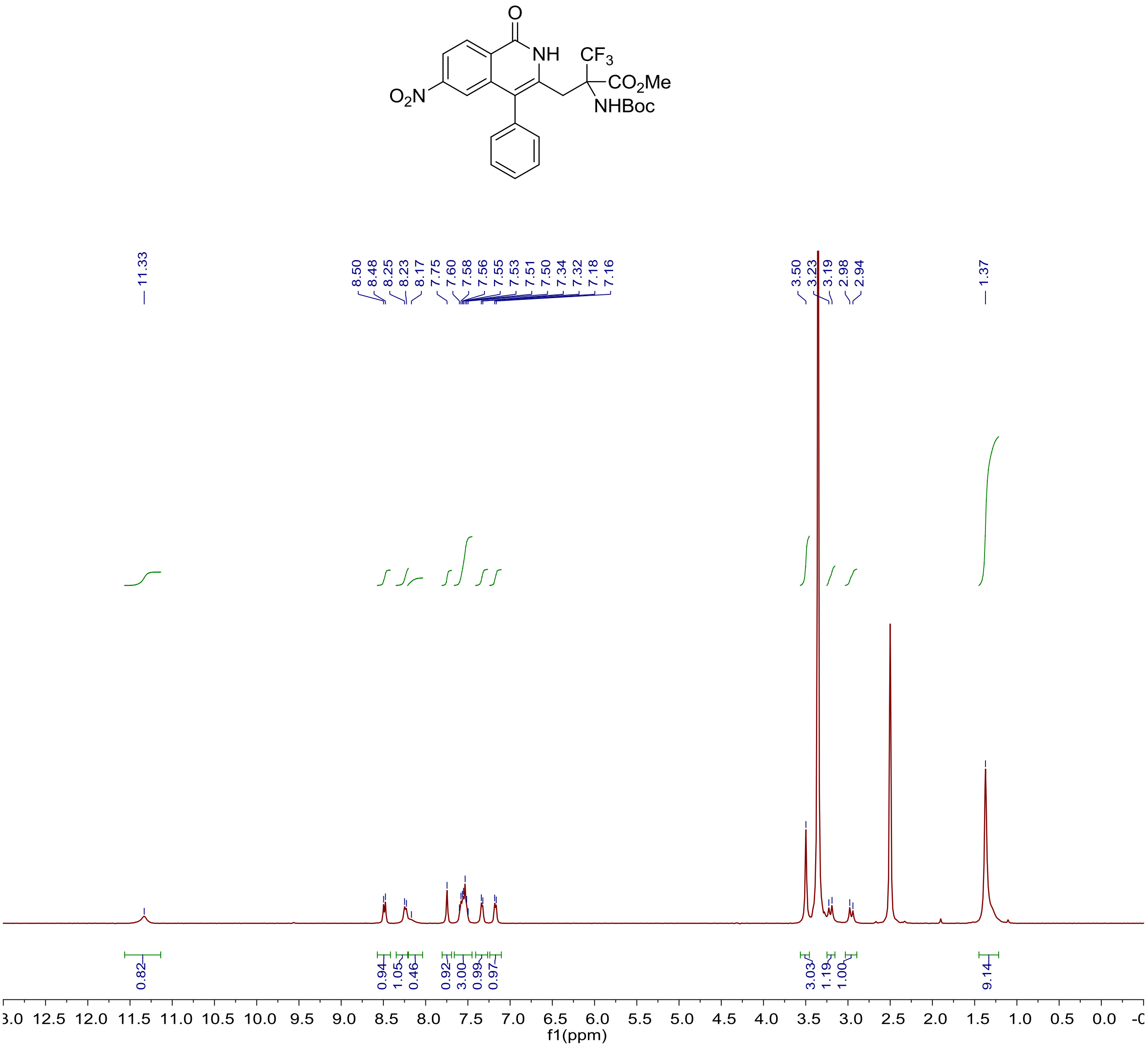

Figure S13. ^{1}H spectrum of **3g** in DMSO-d_6

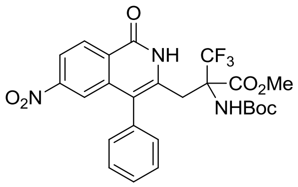

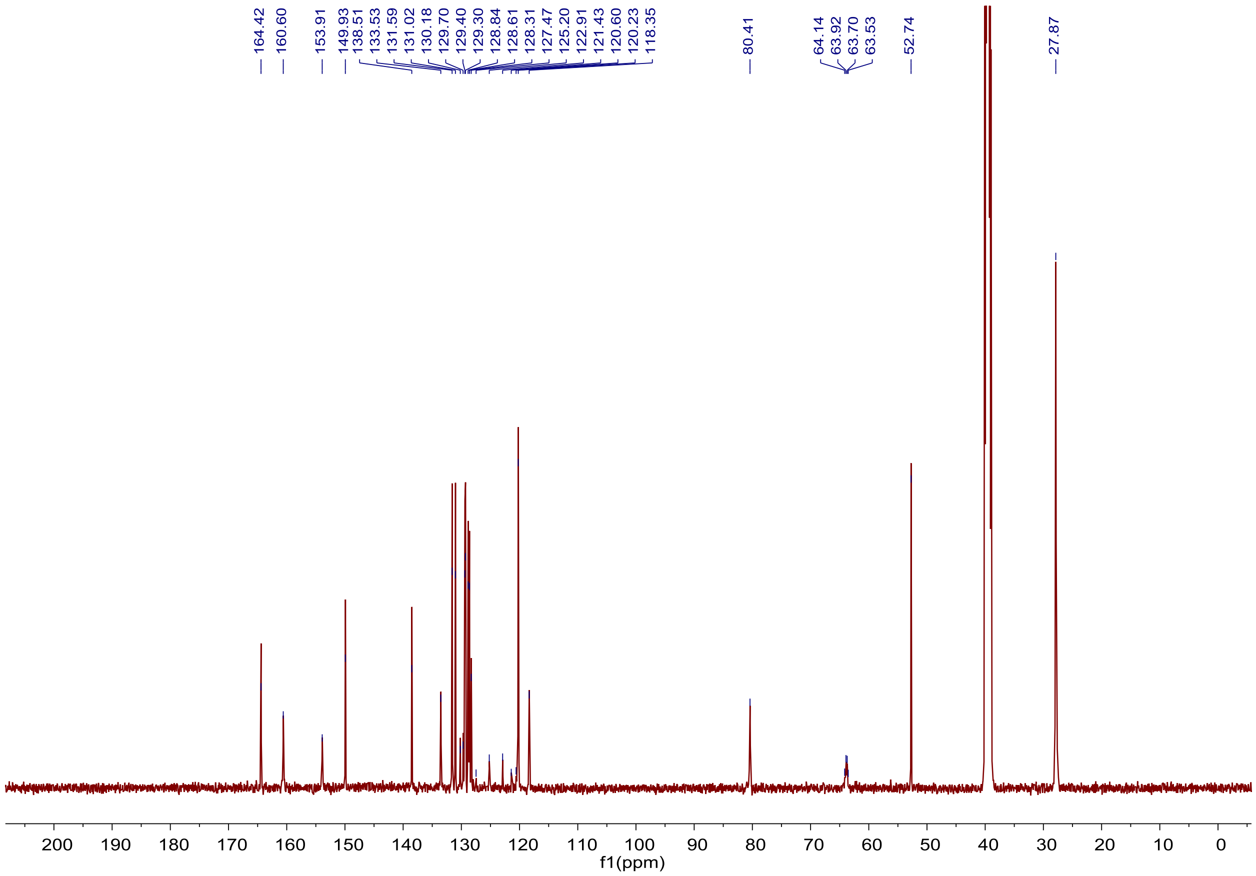

Figure S14. ^{13}C spectrum of **3g** in DMSO-d_6

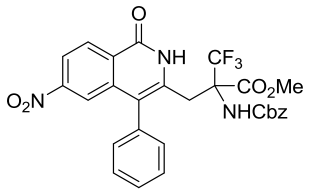

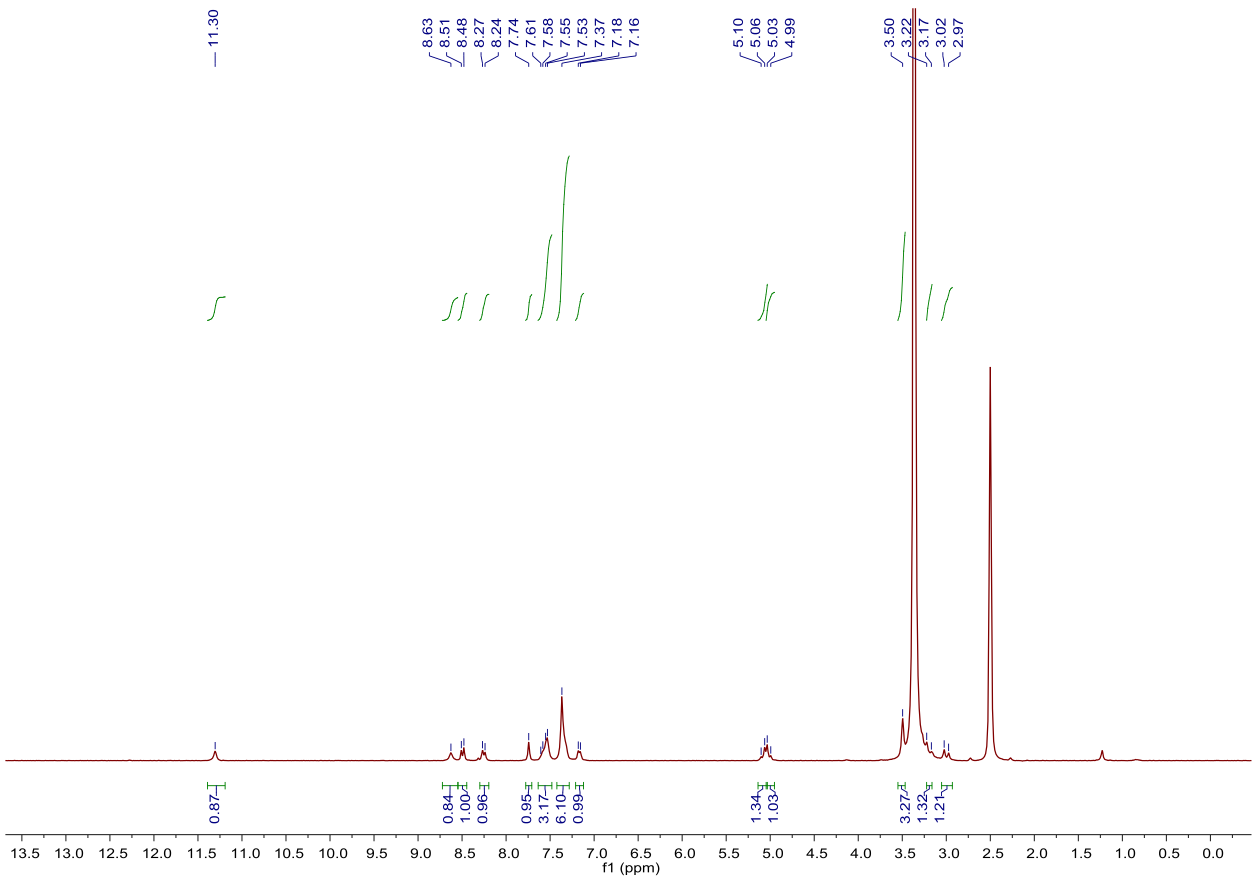

Figure S15. ^{1}H spectrum of **3h** in DMSO-d_6

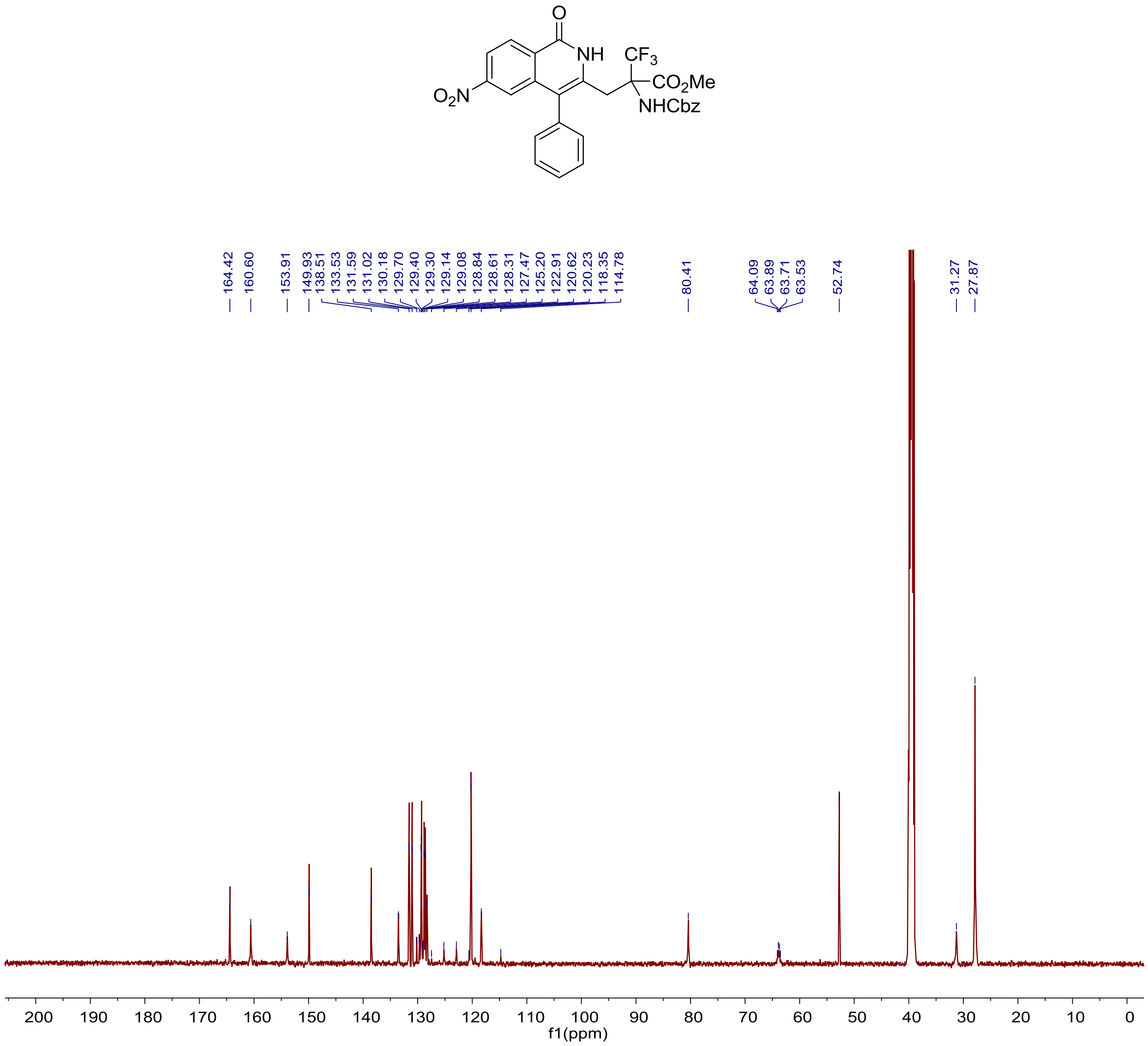

Figure S16. ^{13}C spectrum of **3h** in DMSO-d_6

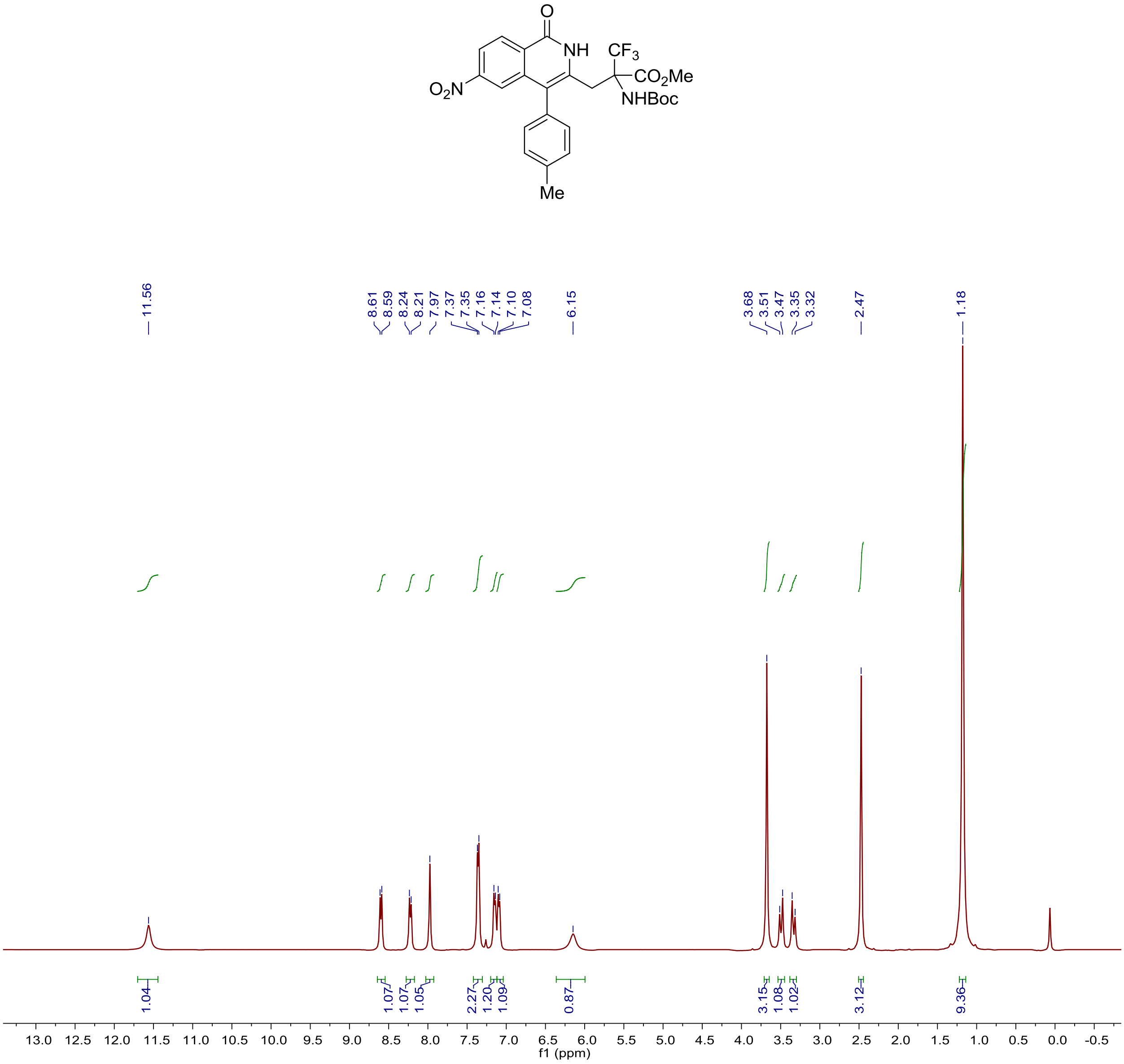

Figure S17. ^{1}H spectrum of **3i** in $CDCl_3$

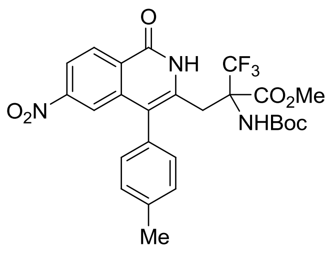

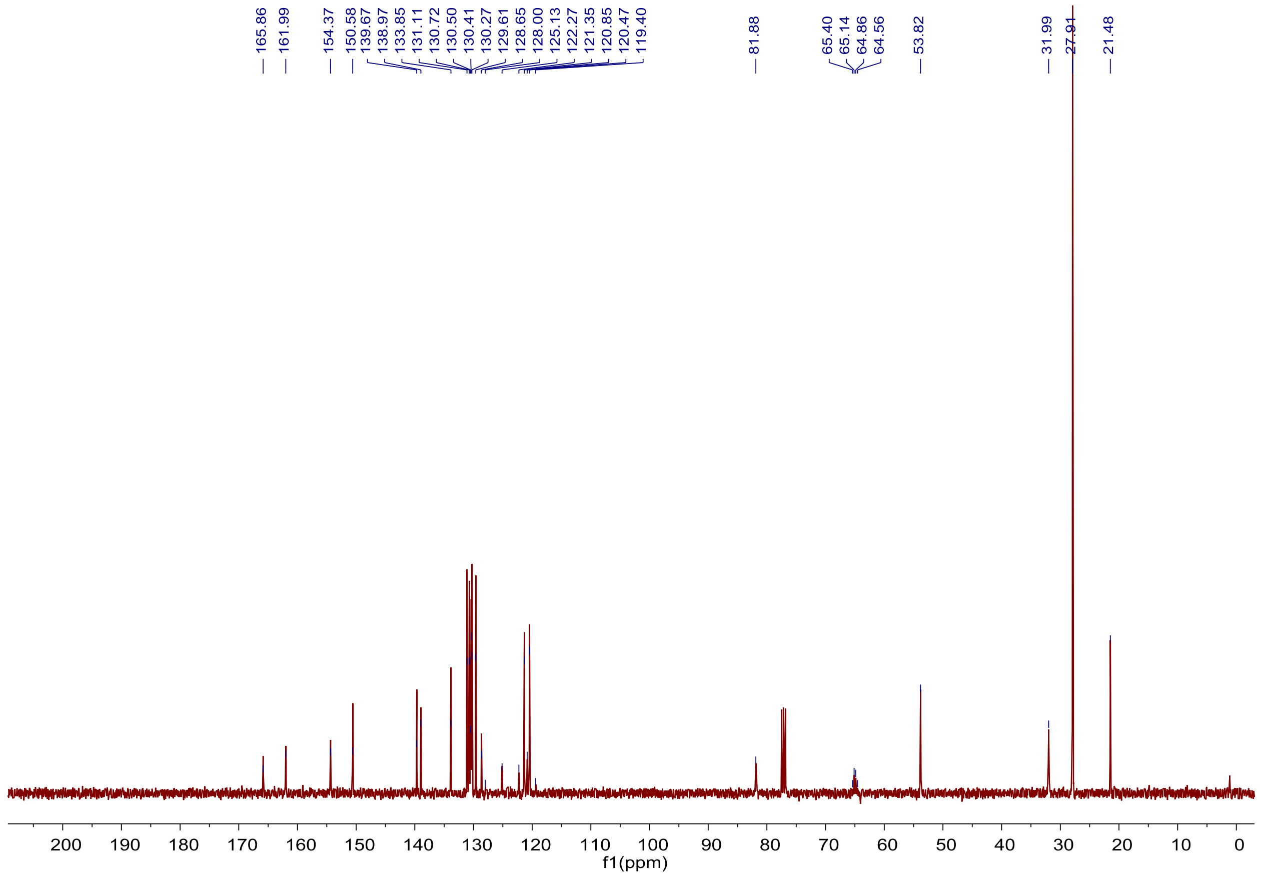

Figure S18. ^{13}C spectrum of **3i** in $CDCl_3$

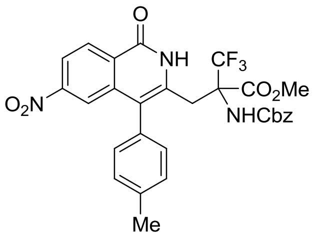

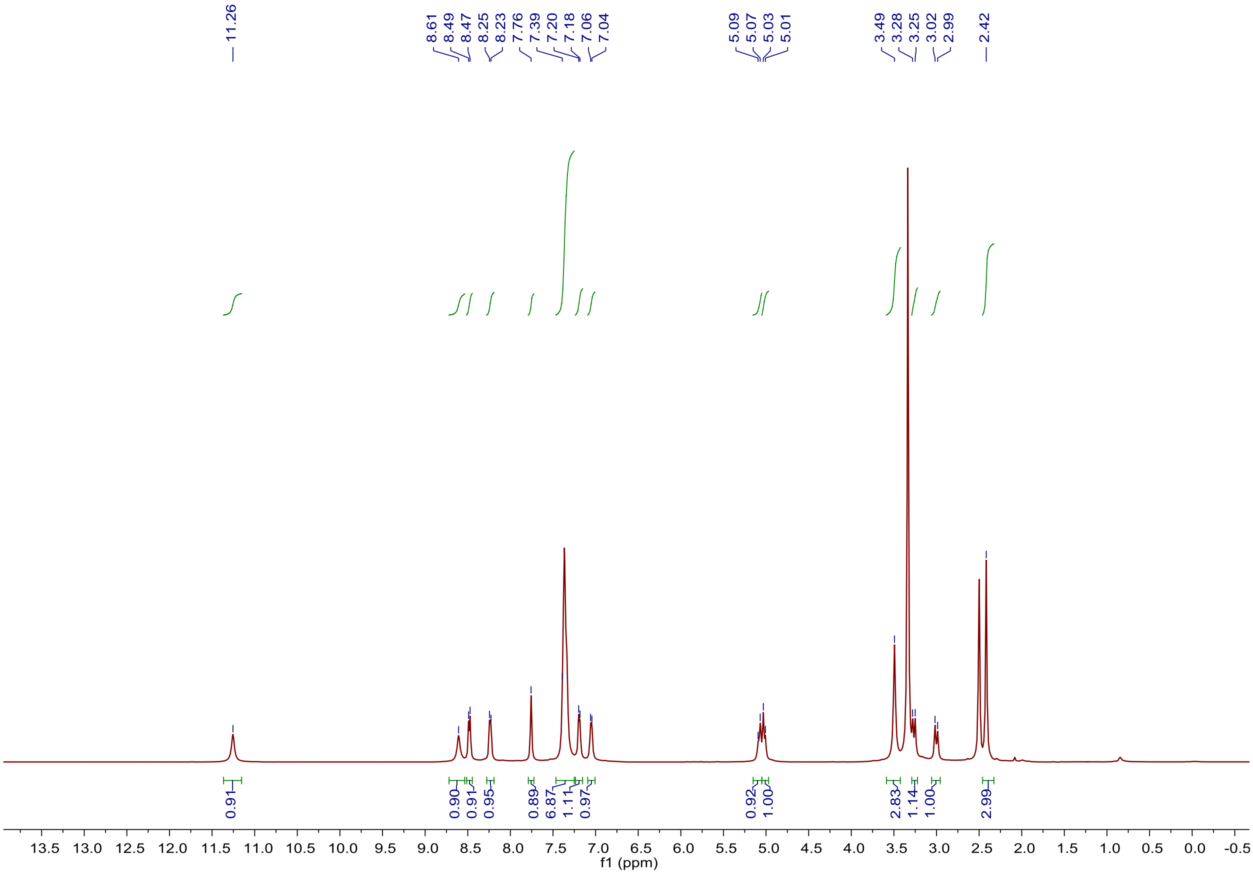

Figure S19. ^{1}H spectrum of **3j** in DMSO-d_6

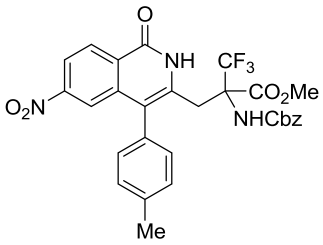

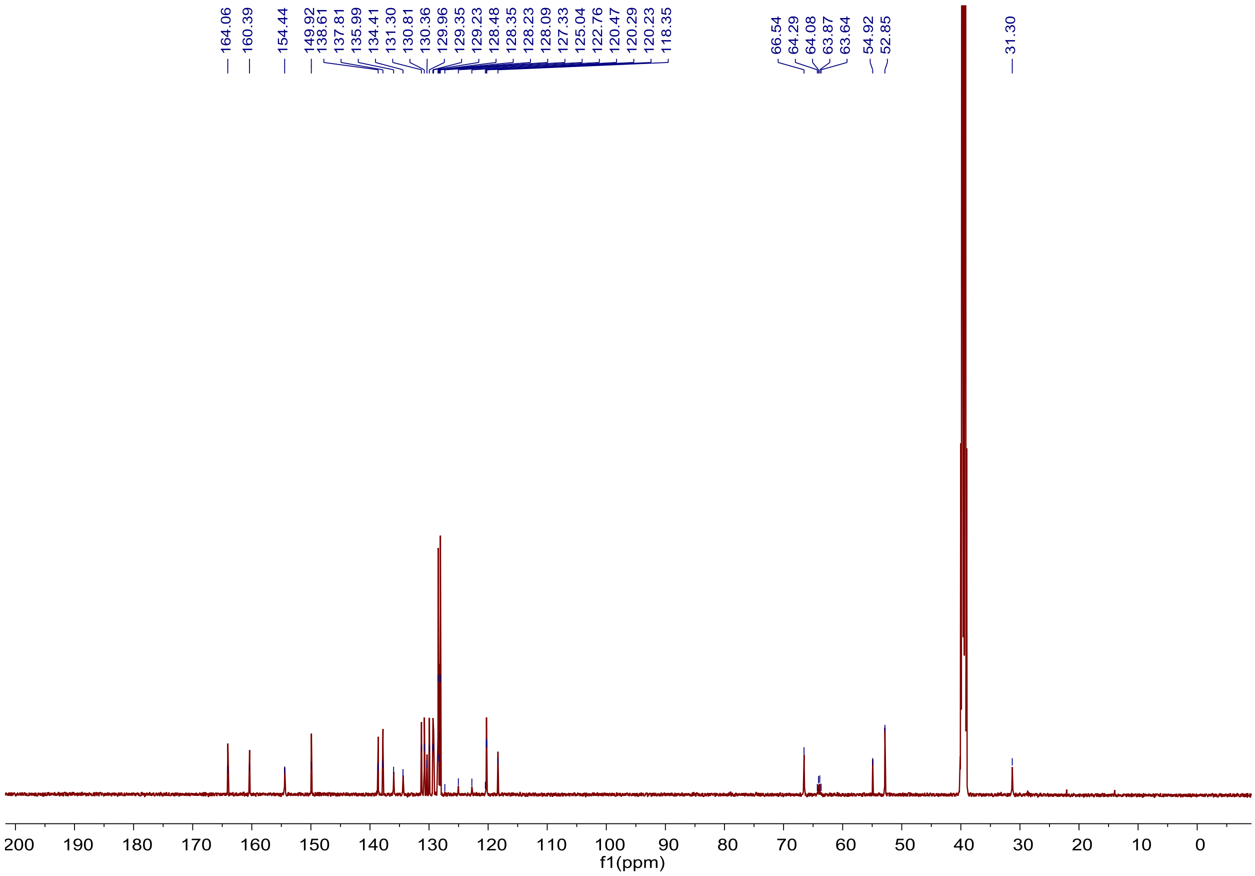

Figure S20. ^{13}C spectrum of **3j** in DMSO-d_6

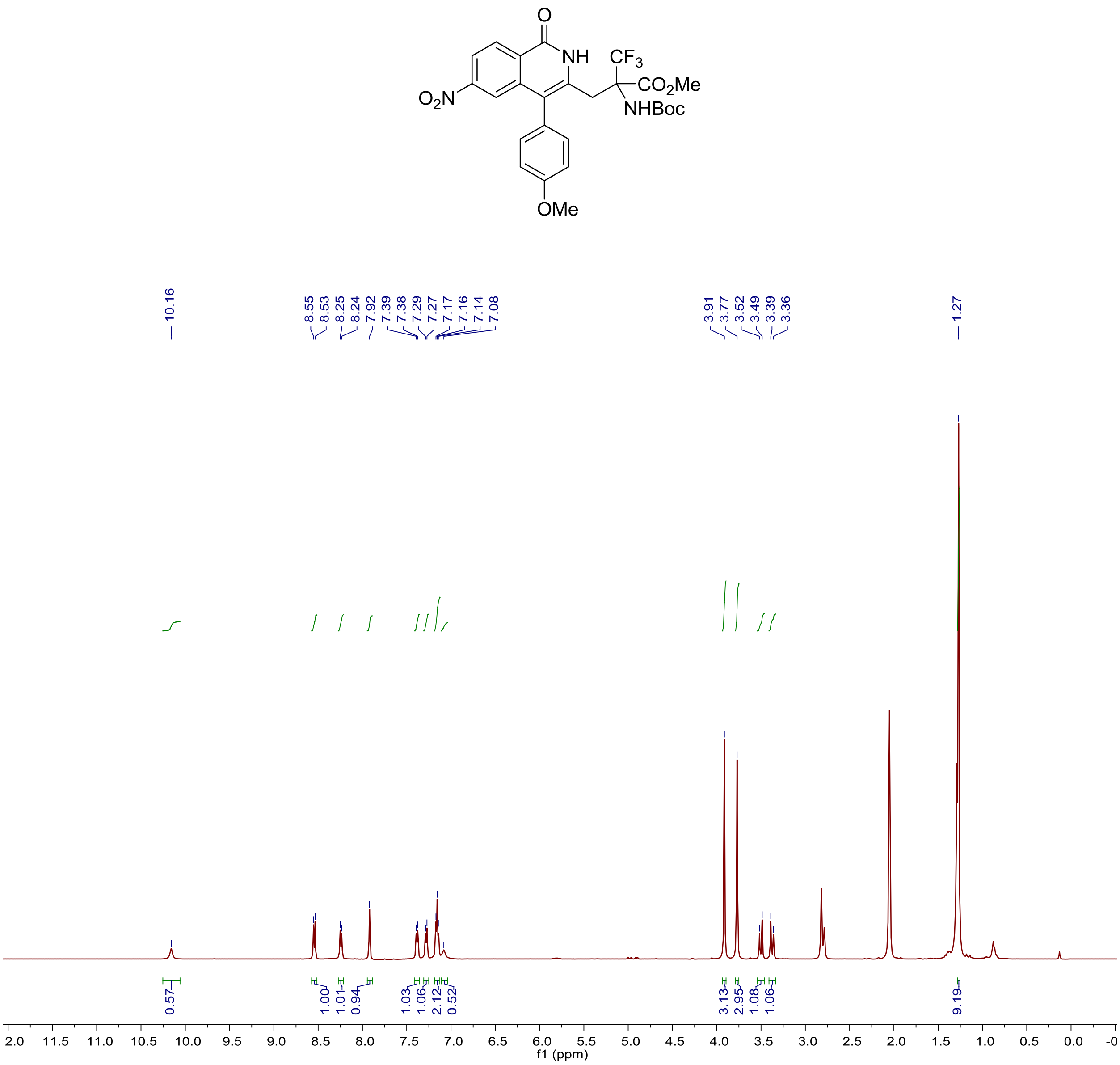

Figure S21. ^{1}H spectrum of **3k** in acetone-d_6

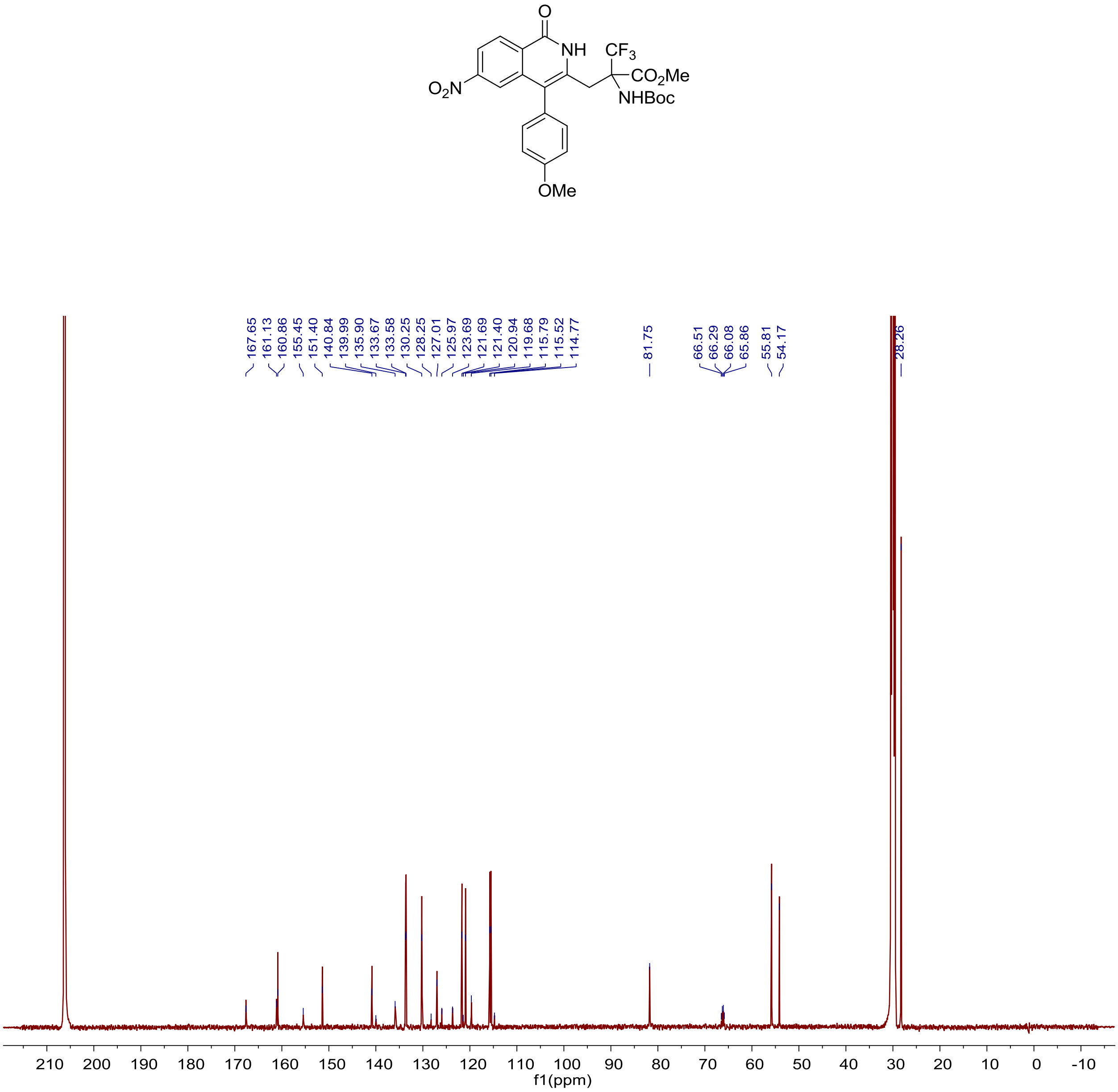

Figure S22. ^{13}C spectrum of **3k** in acetone-d_6

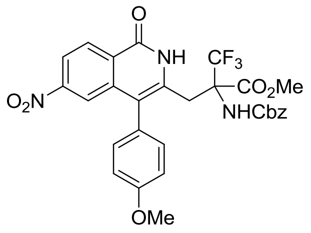

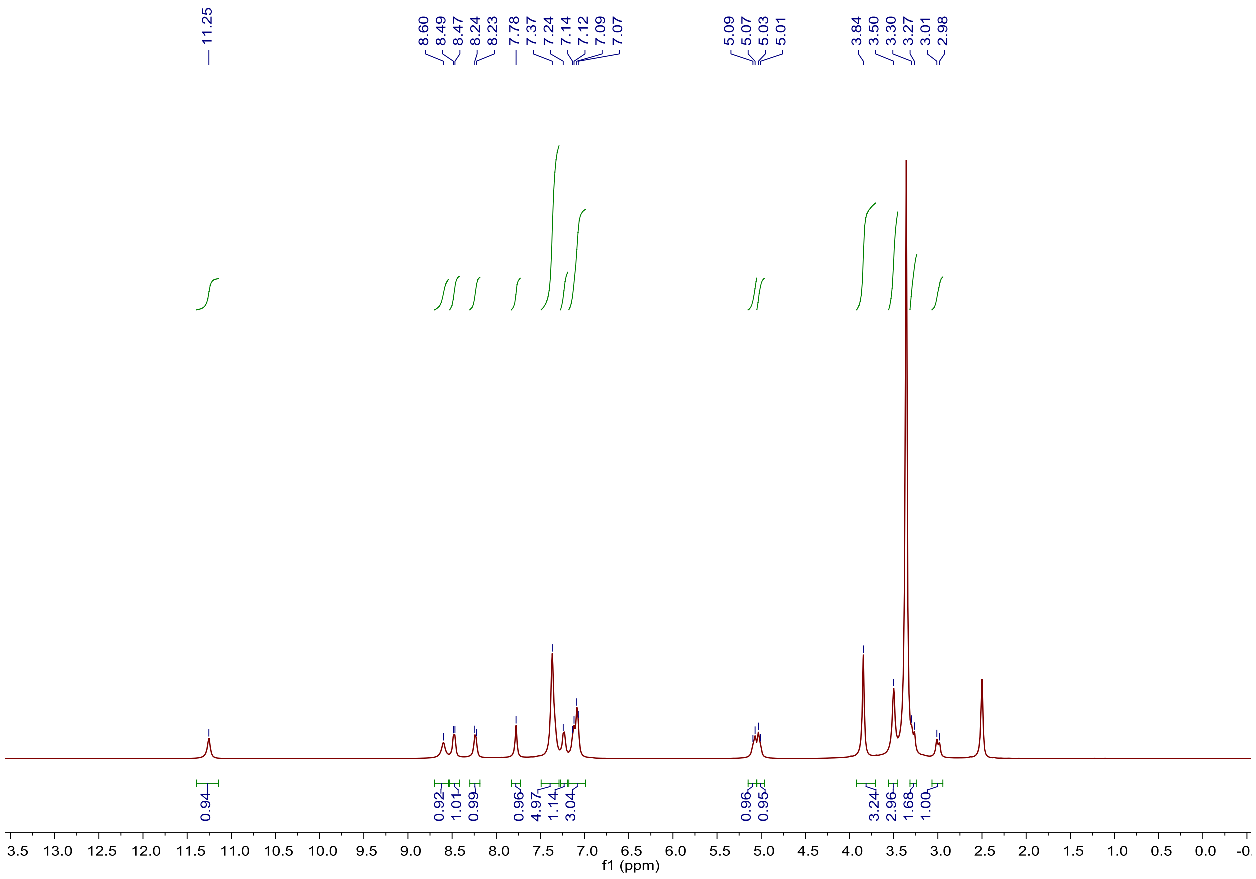

Figure S23. ^{1}H spectrum of **3l** in DMSO-d_6

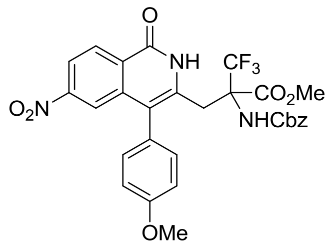

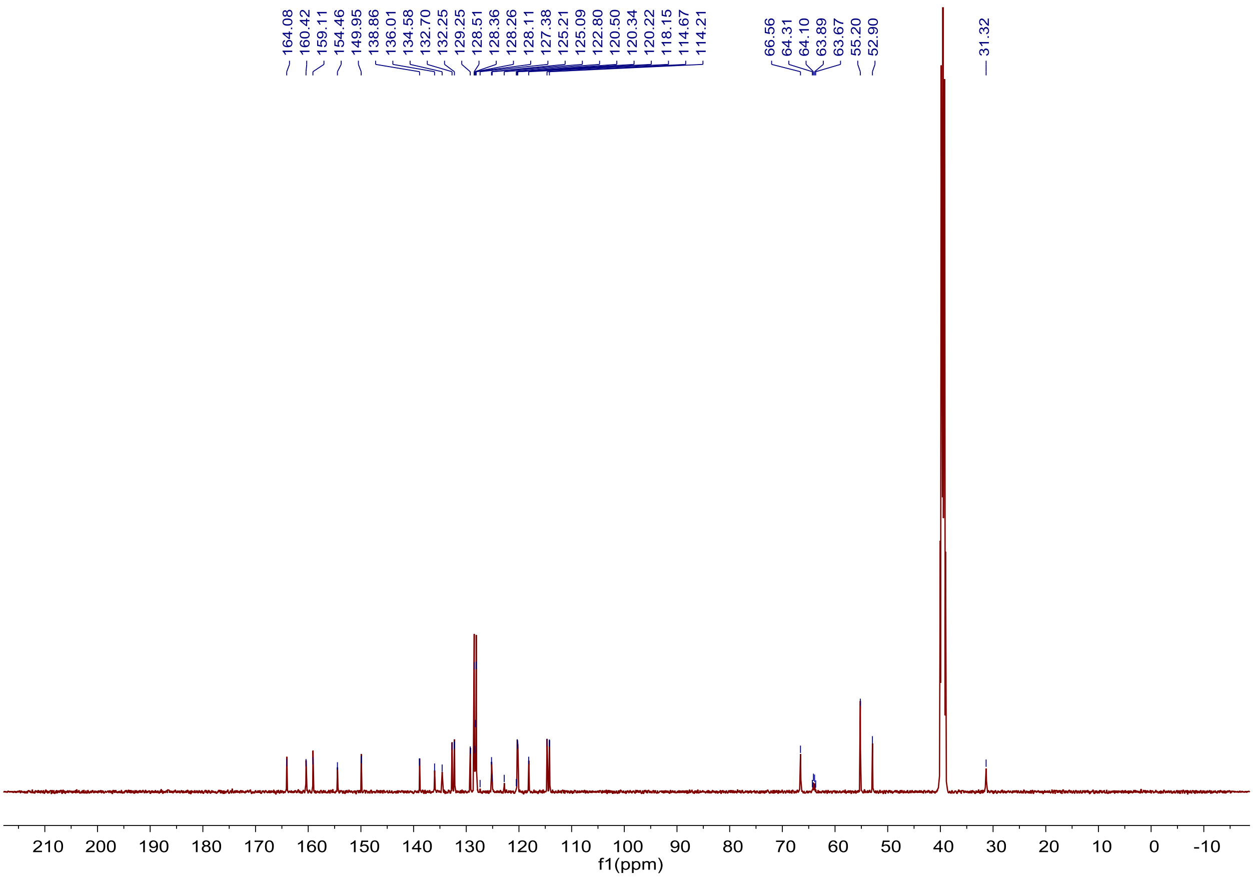

Figure S24. ^{13}C spectrum of **3l** in DMSO-d_6

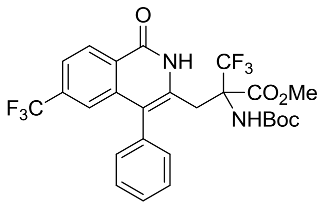

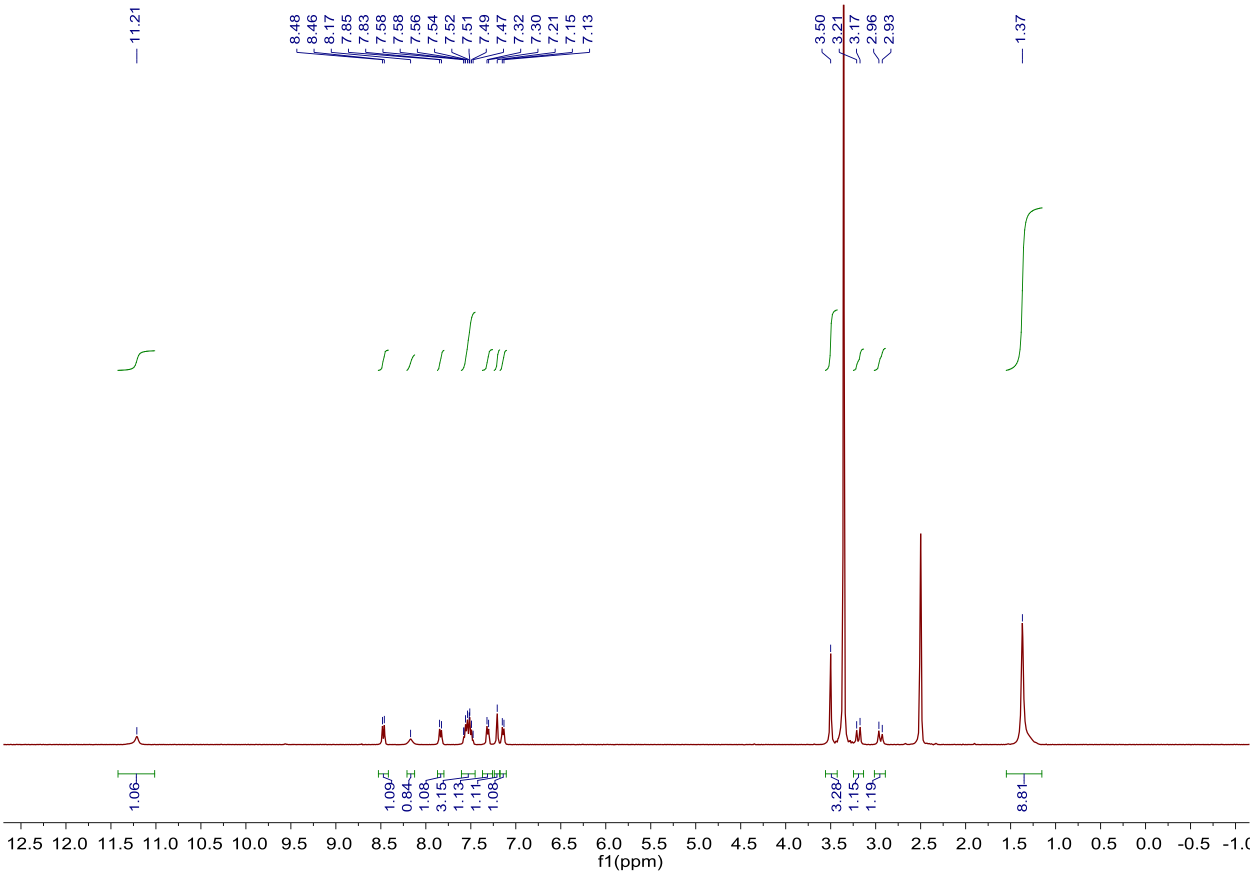

Figure S25. ^{1}H spectrum of **3m** in DMSO-d_6

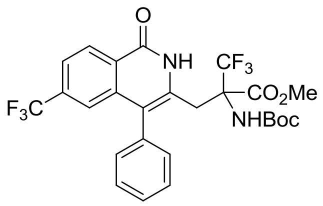

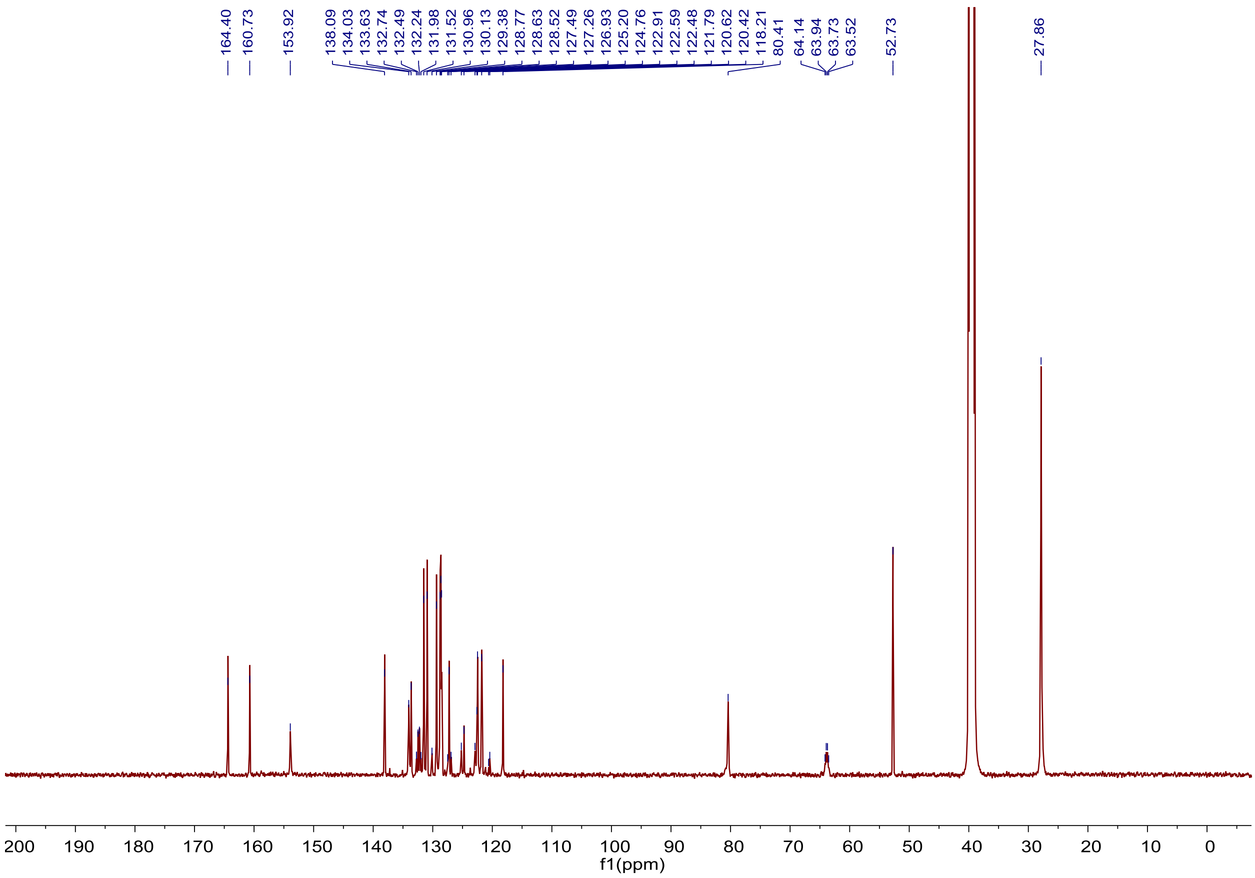

Figure S26. ^{13}C spectrum of **3m** in DMSO-d_6

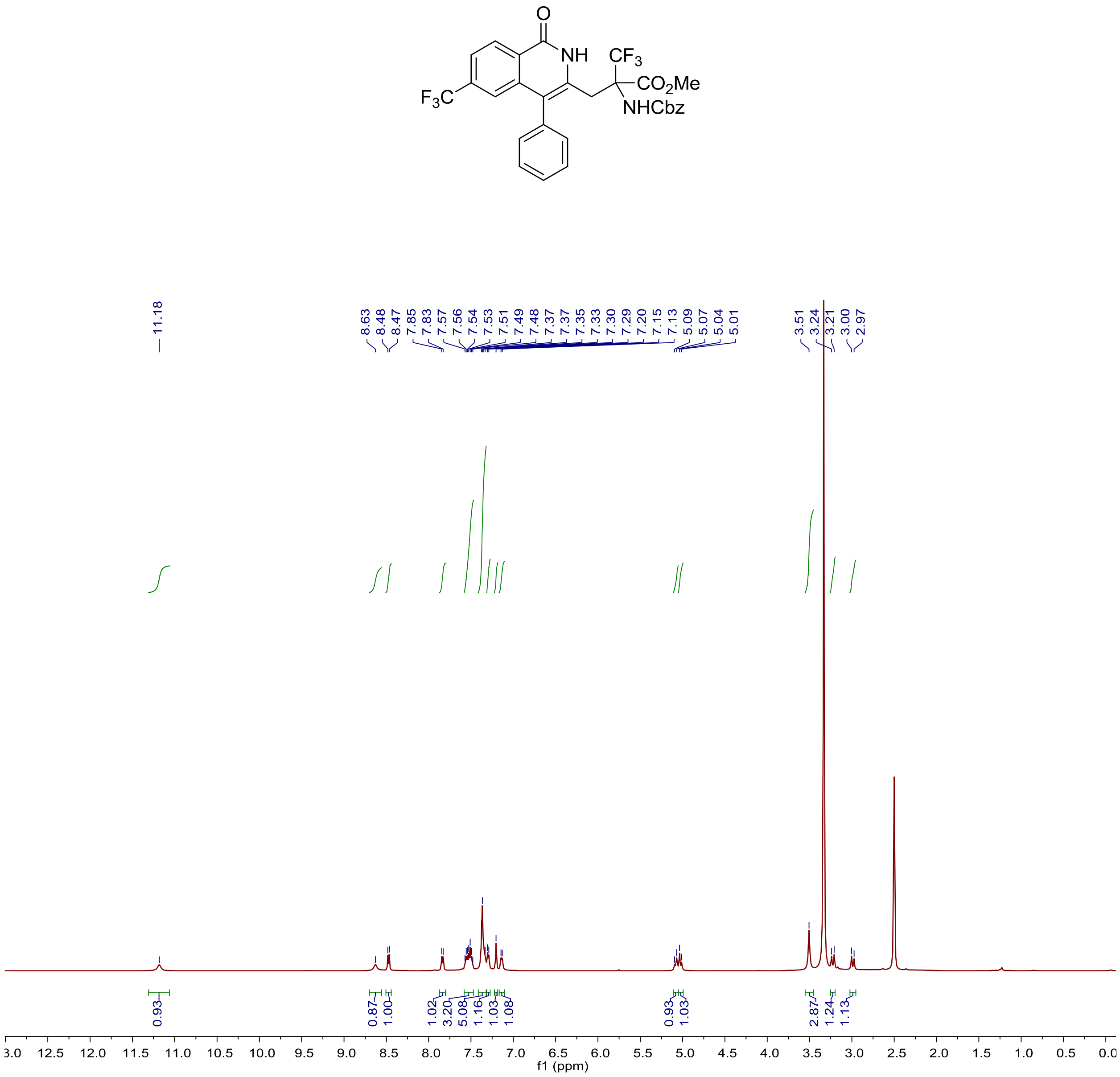

Figure S27. ^{1}H spectrum of **3n** in DMSO-d_6

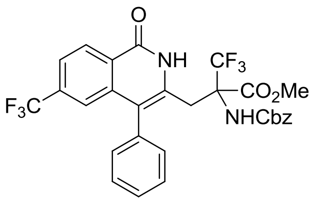

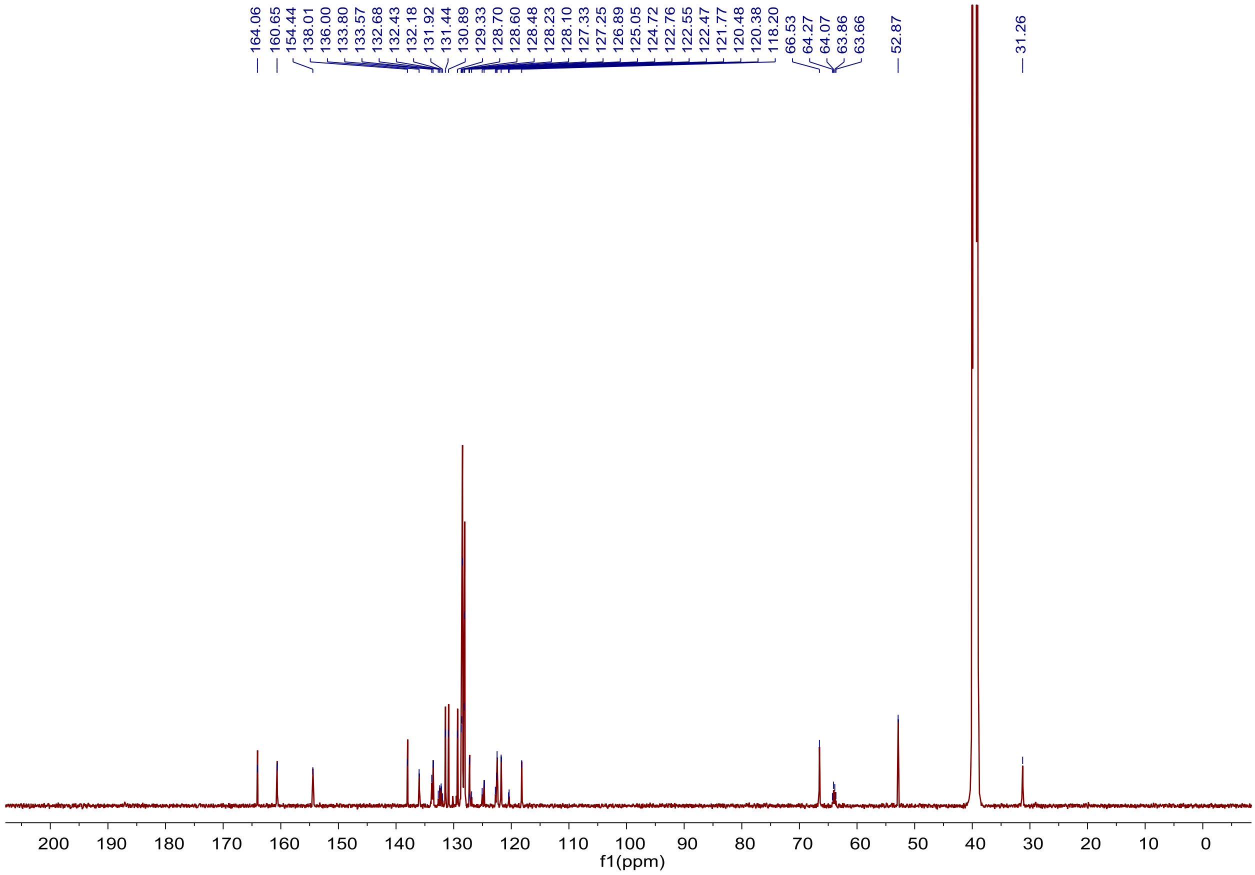

Figure S28. ^{13}C spectrum of **3n** in DMSO-d_6

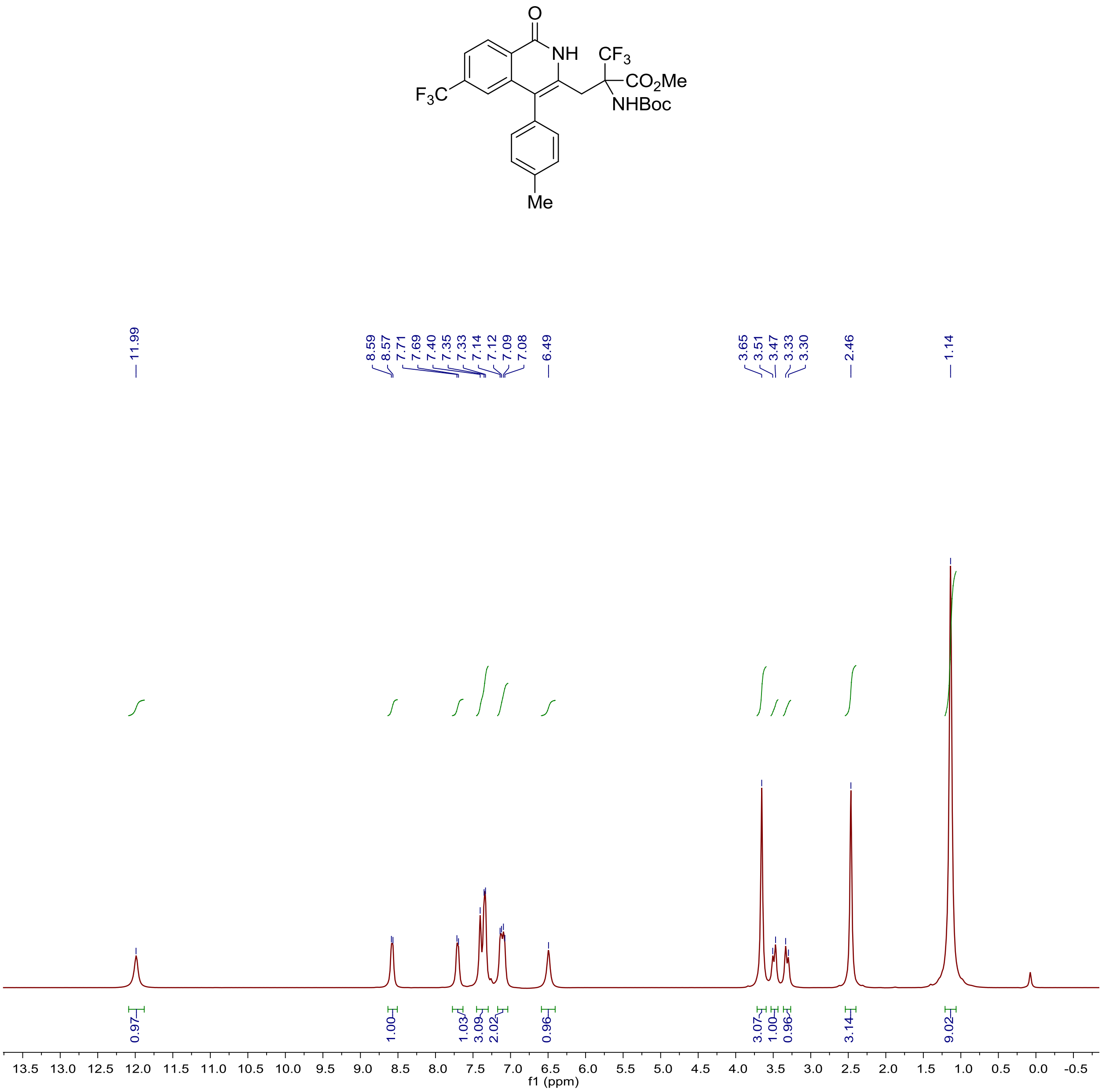

Figure S29. ^{1}H spectrum of **3o** in $CDCl_3$

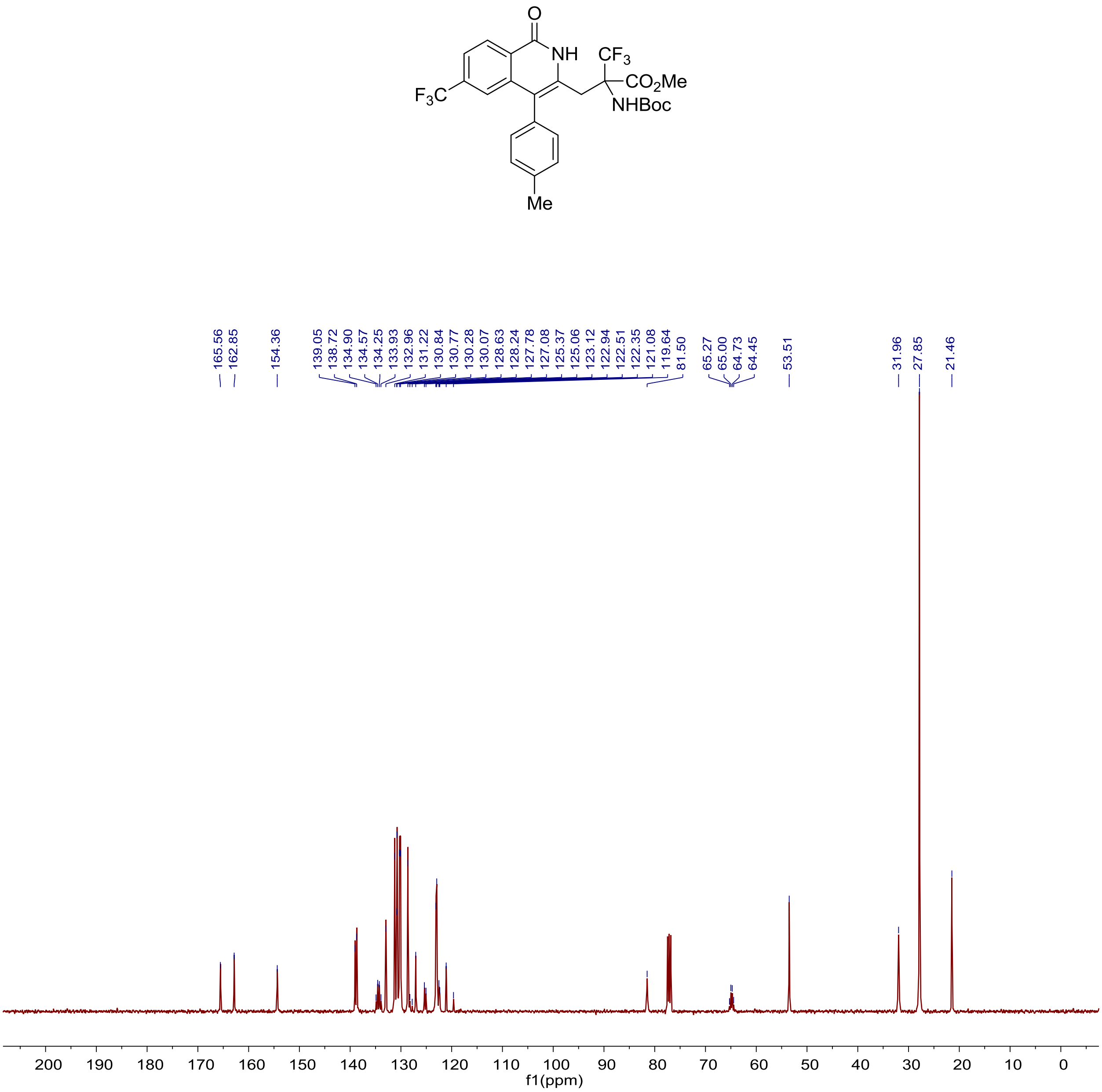

Figure S30. ^{13}C spectrum of **3o** in $CDCl_3$

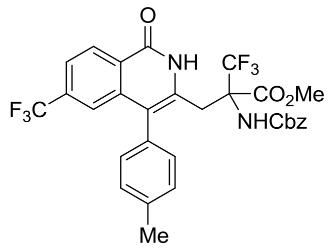

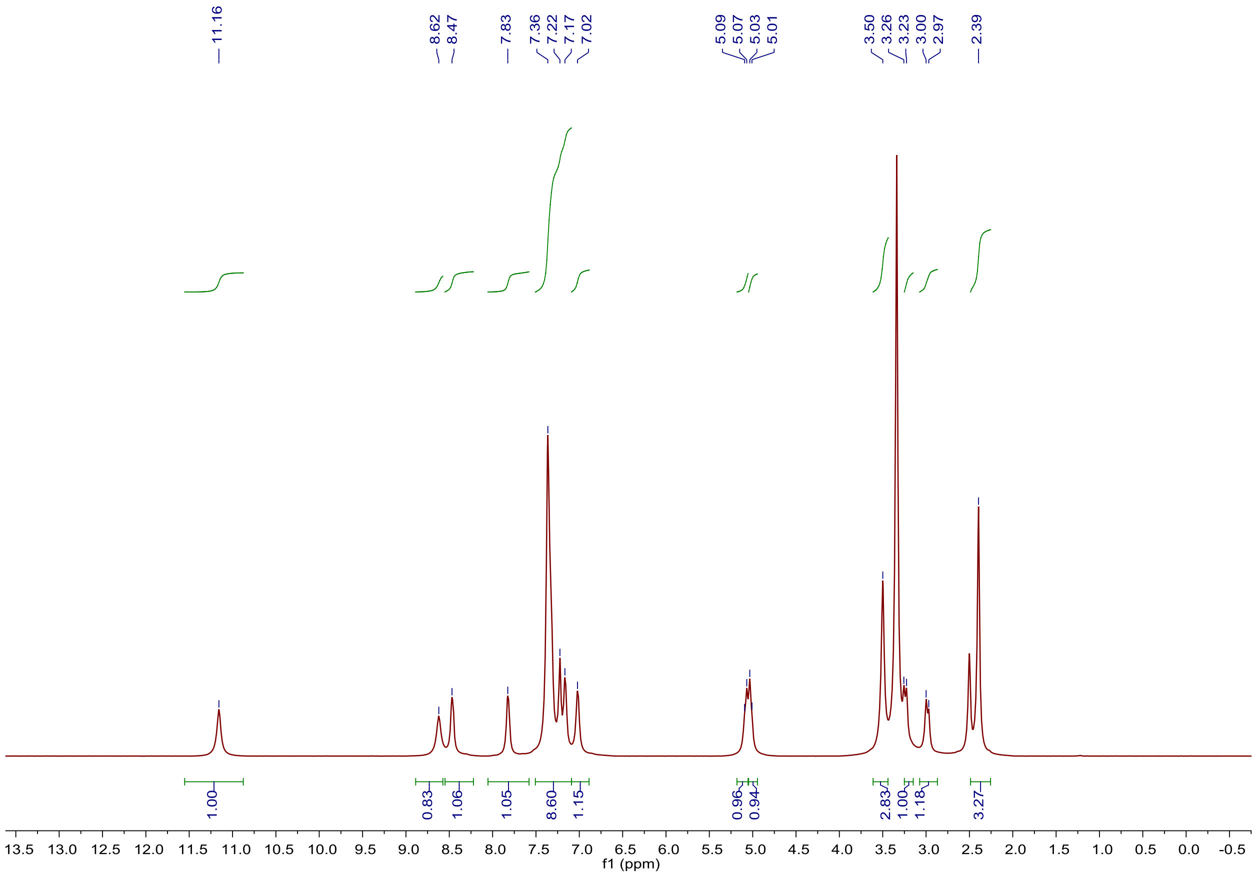

Figure S31. ^{1}H spectrum of **3p** in DMSO-d_6

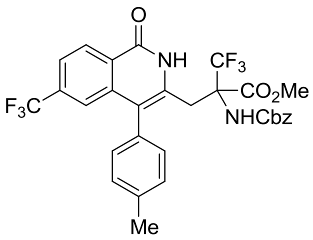

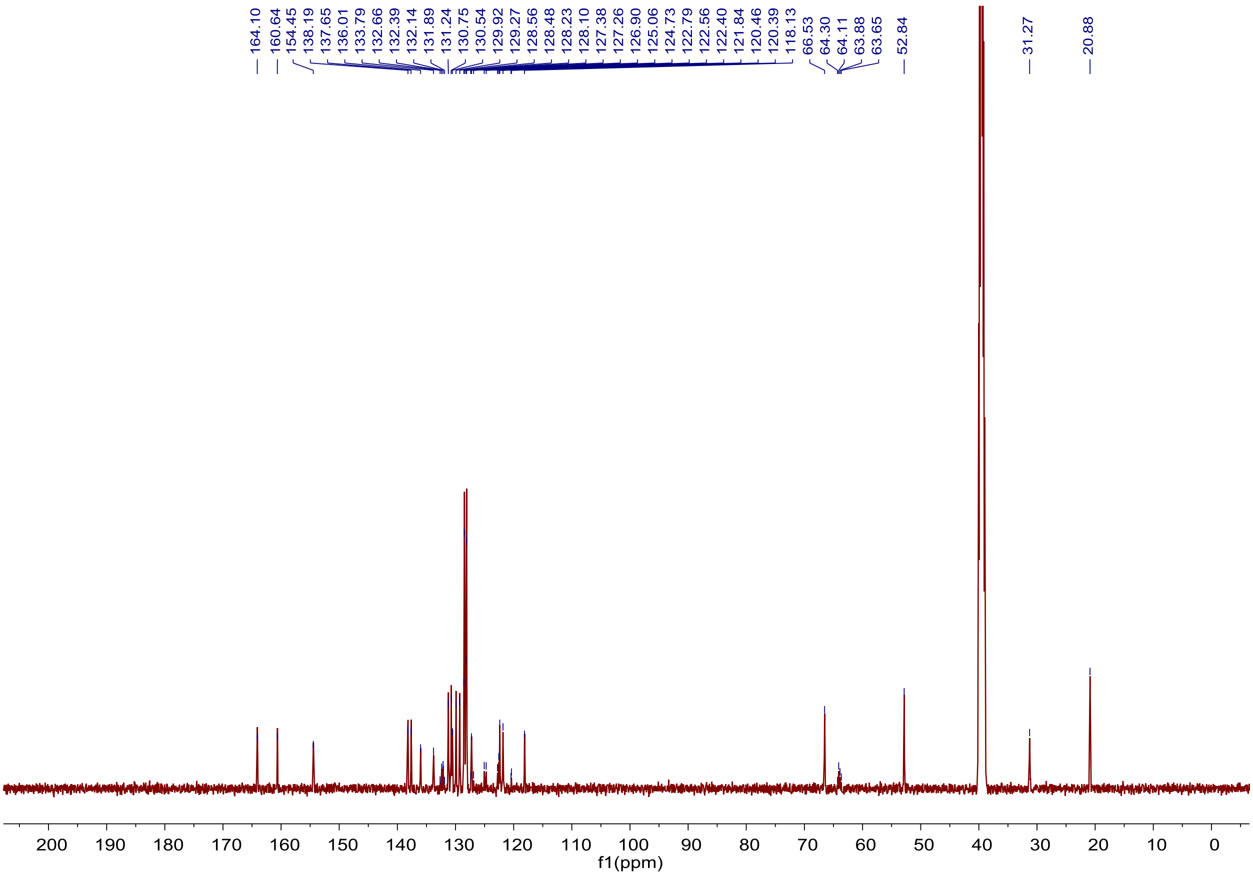

Figure S32. ^{13}C spectrum of **3p** in DMSO-d_6

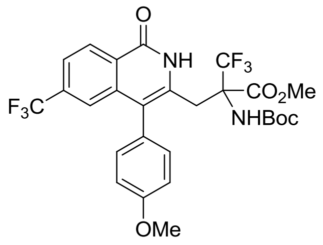

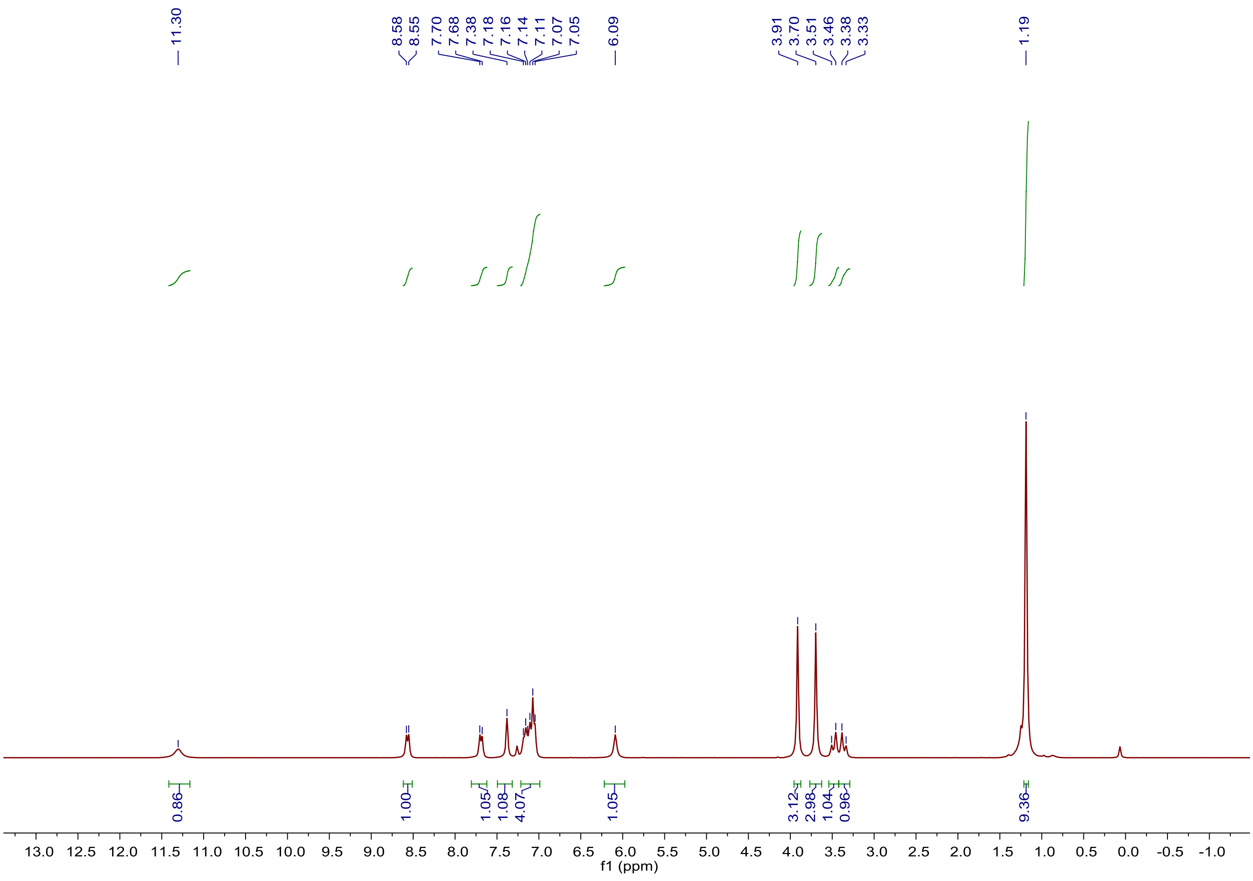

Figure S33. ^{1}H spectrum of **3q** in $CDCl_3$

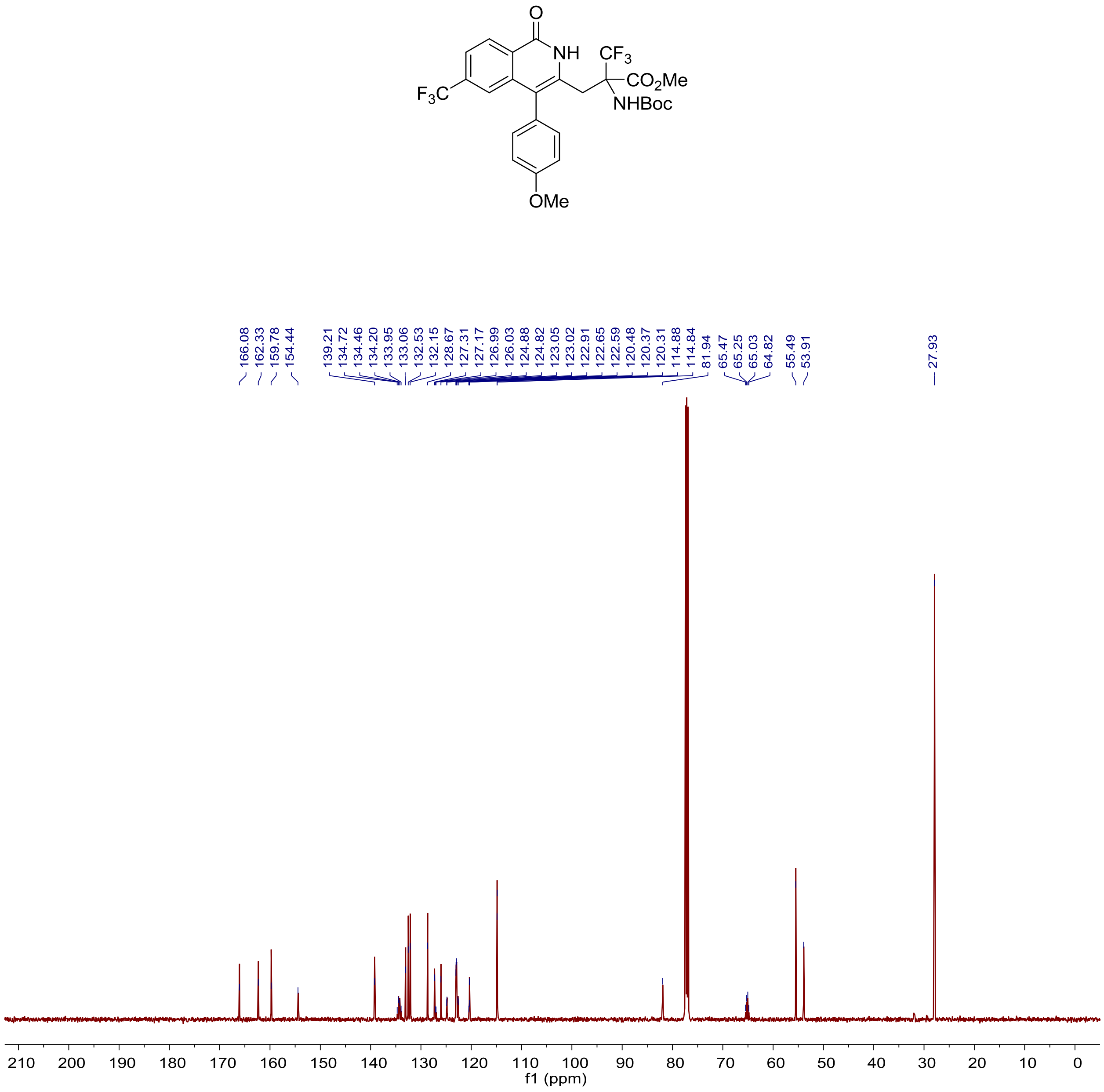

Figure S34. ^{13}C spectrum of **3q** in $CDCl_3$

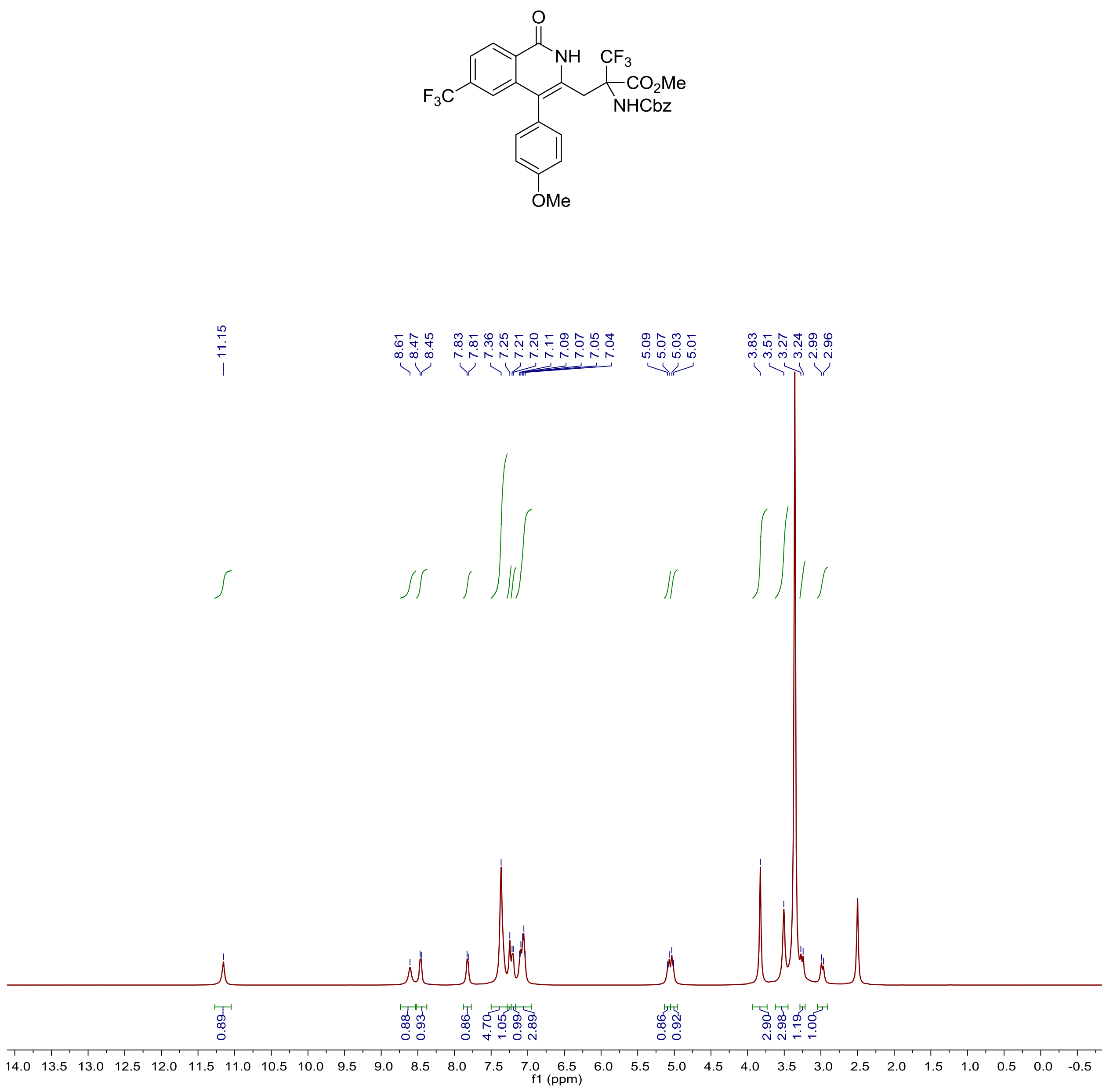

Figure S35. ^{1}H spectrum of **3r** in DMSO-d_6

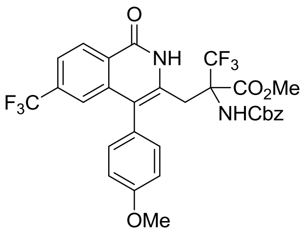

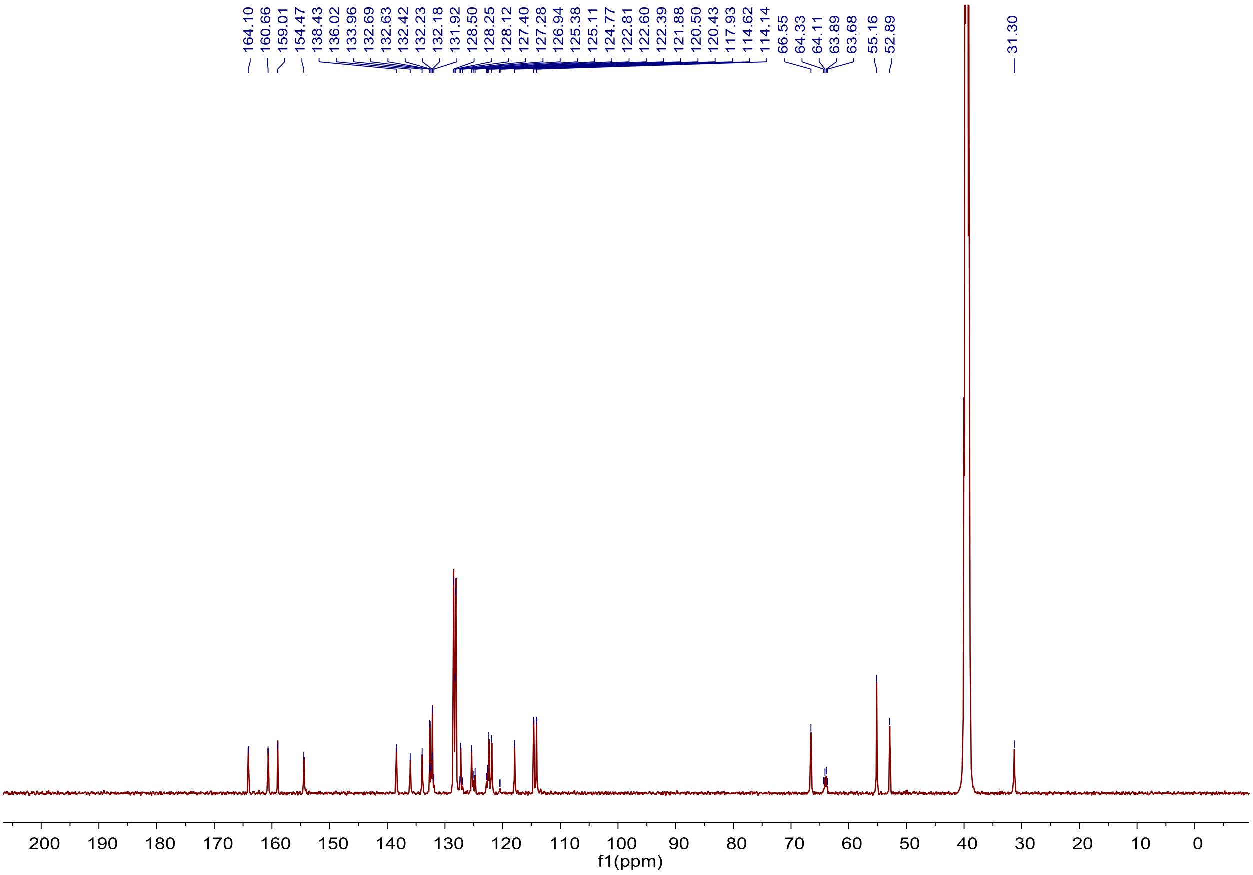

Figure S36. ^{13}C spectrum of **3r** in DMSO-d_6

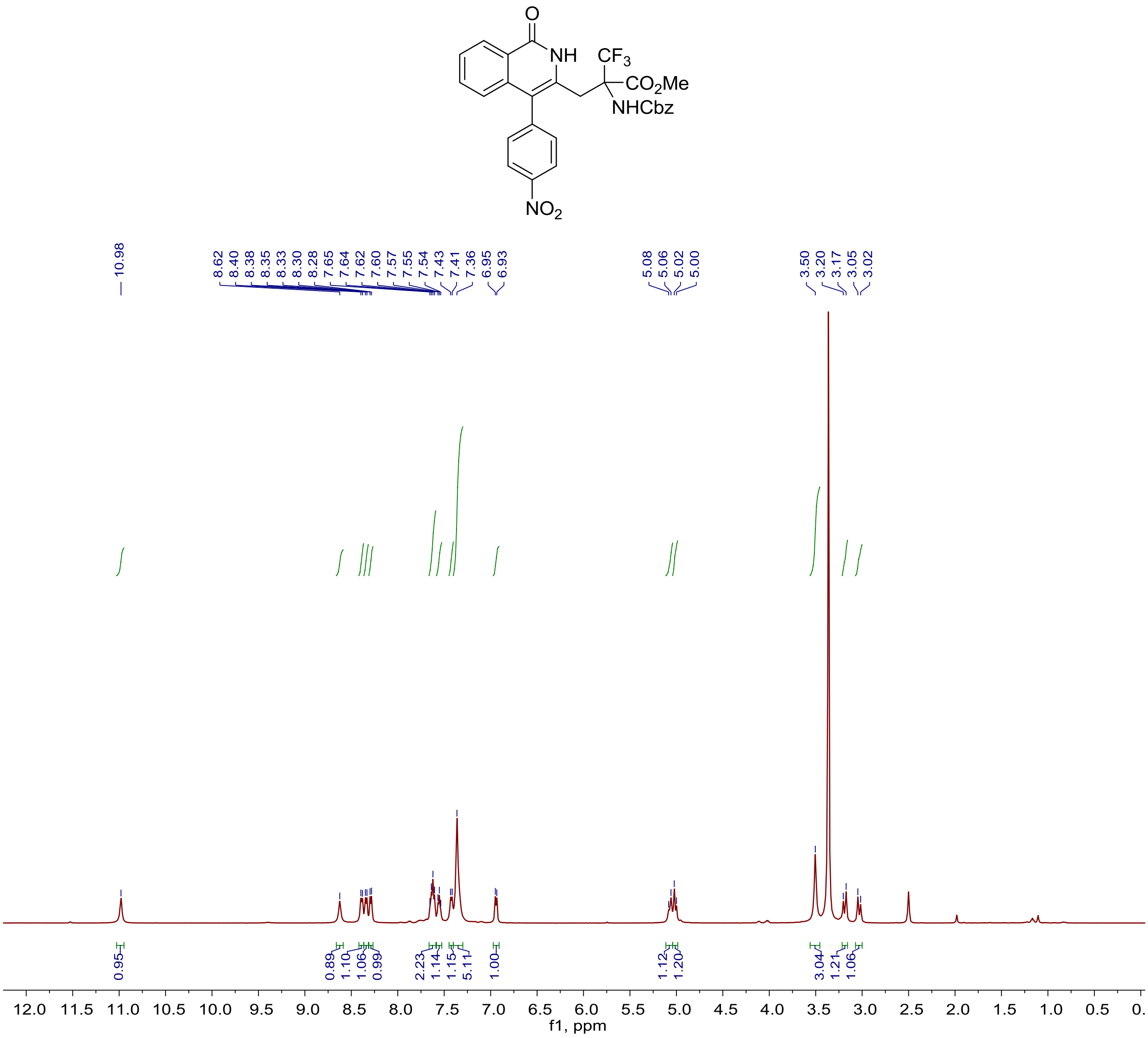

Figure S37. ^{1}H spectrum of **3s** in DMSO-d_6

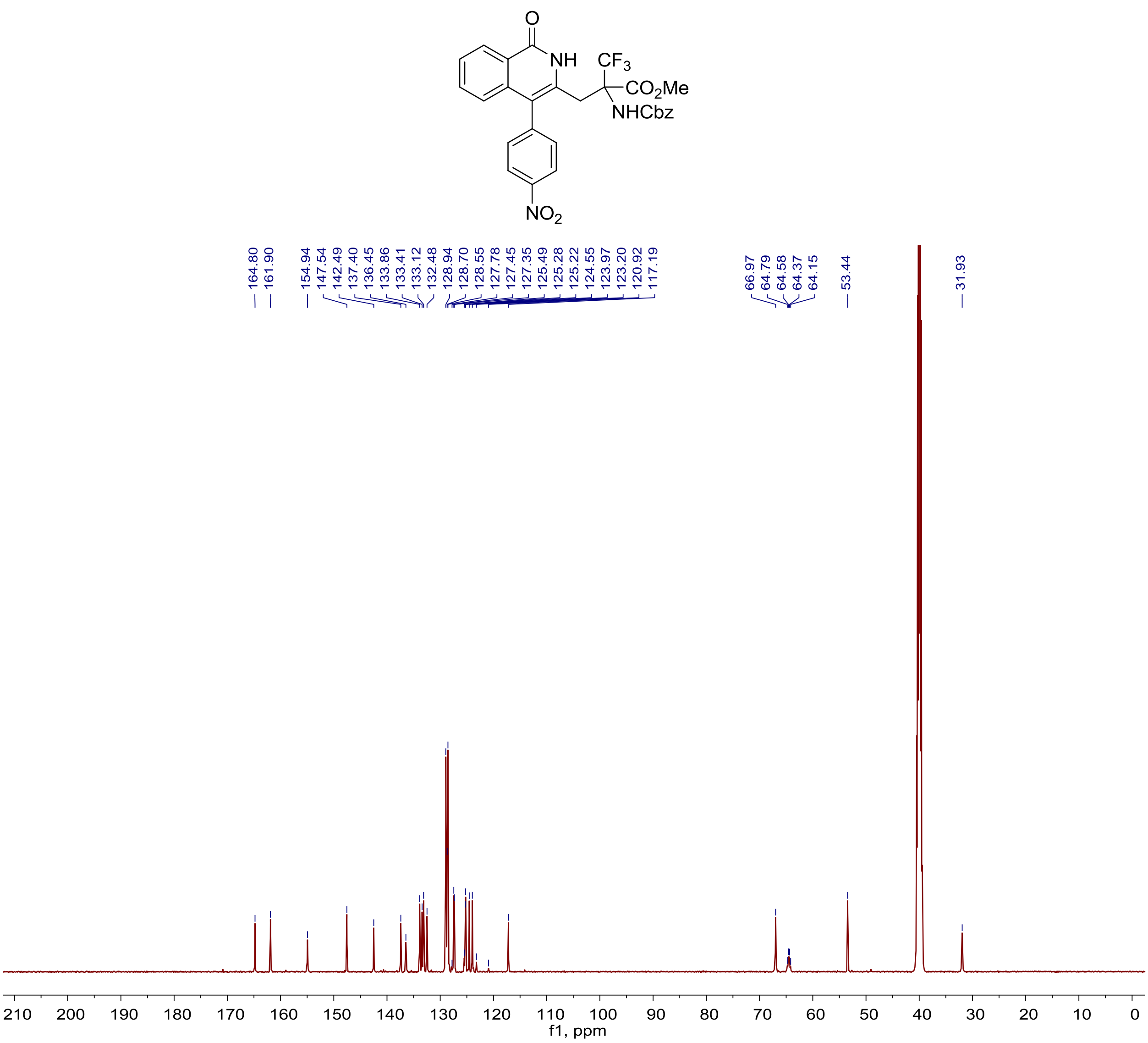

Figure S38. ^{13}C spectrum of **3s** in DMSO-d_6

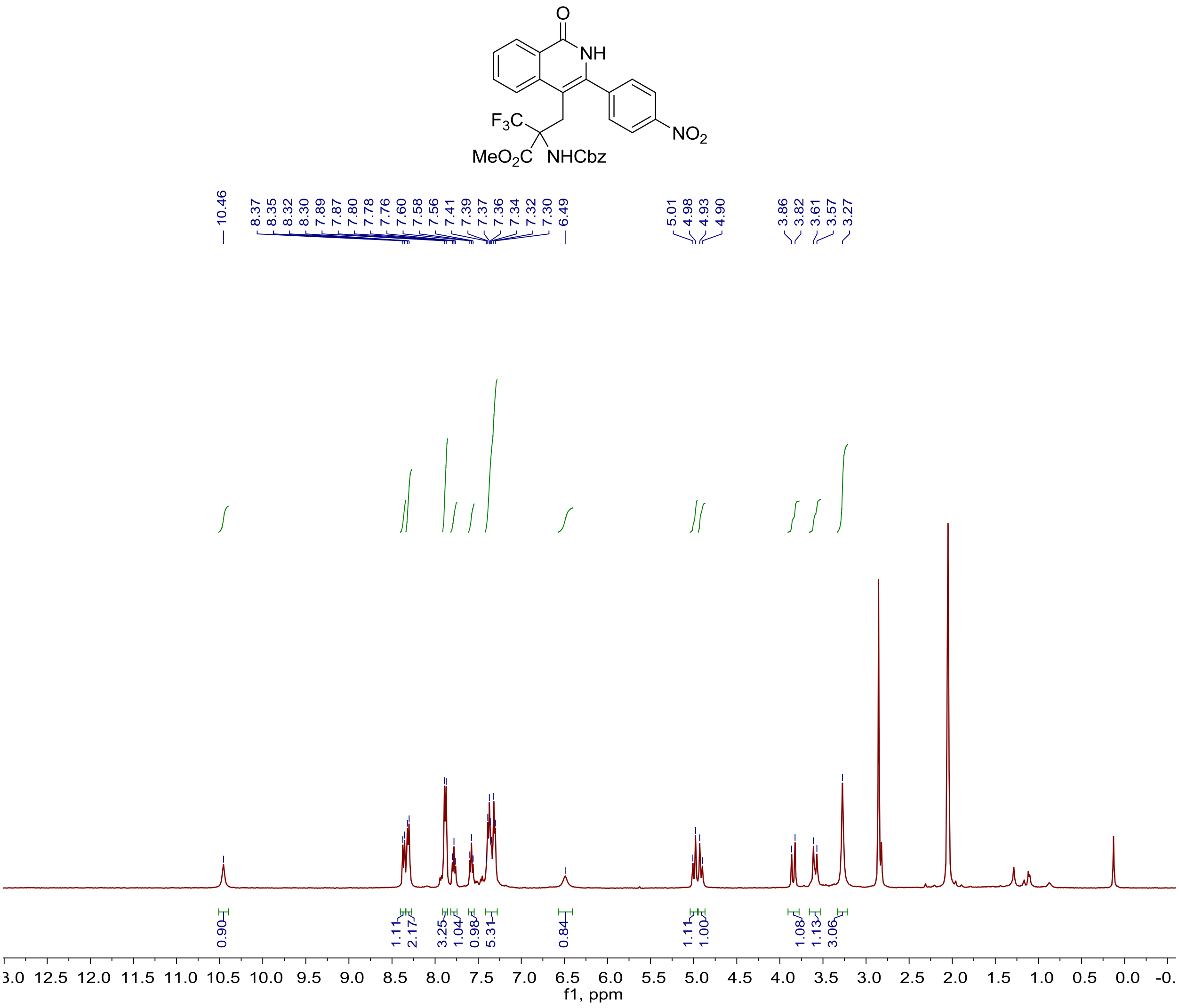

Figure S39. ^{1}H spectrum of **3t** in acetone-d_6

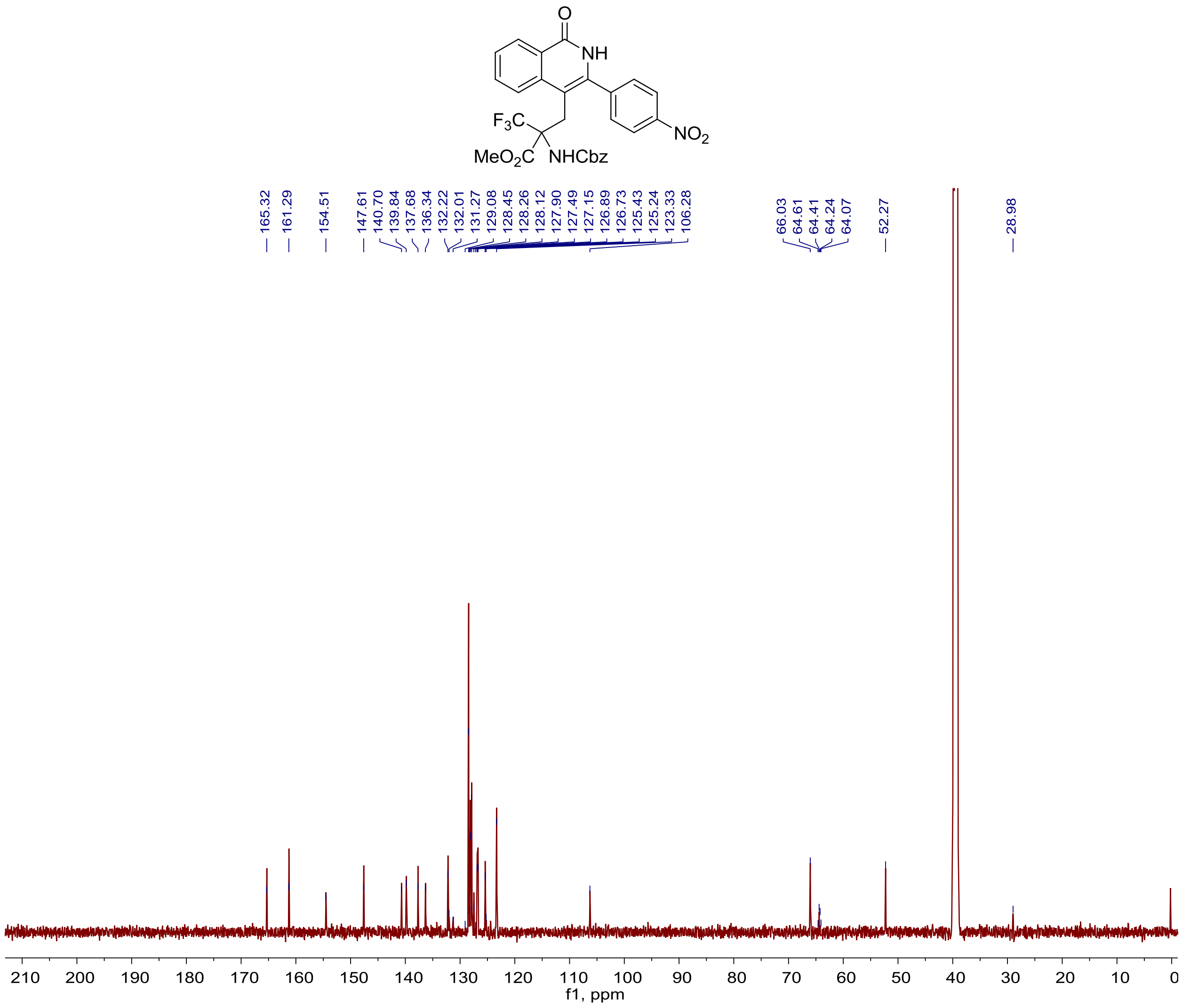

Figure S40. ^{13}C spectrum of **3t** in DMSO-d_6

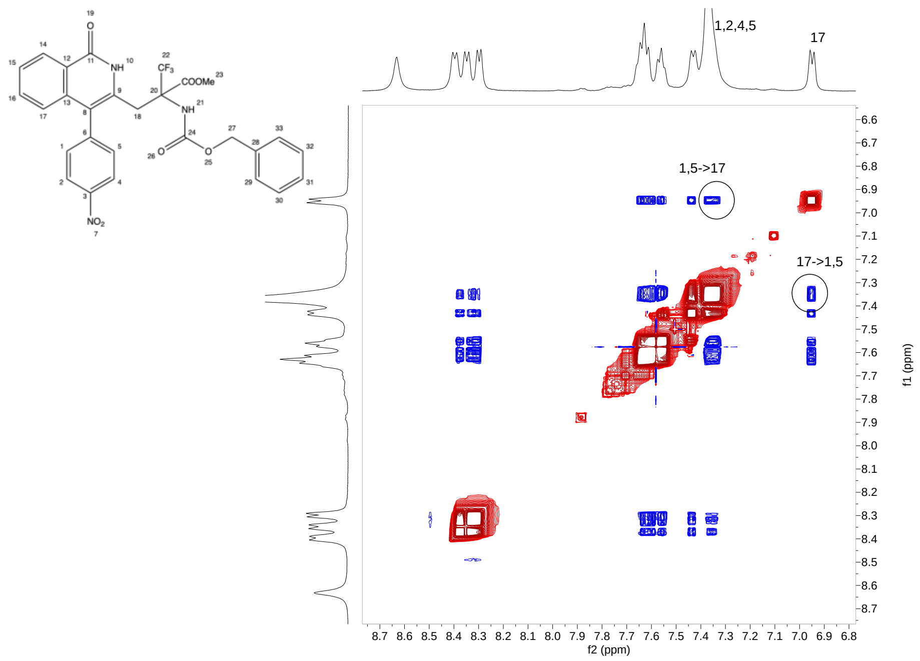

Figure S41. ROESY NMR of **3s** in DMSO-d_6

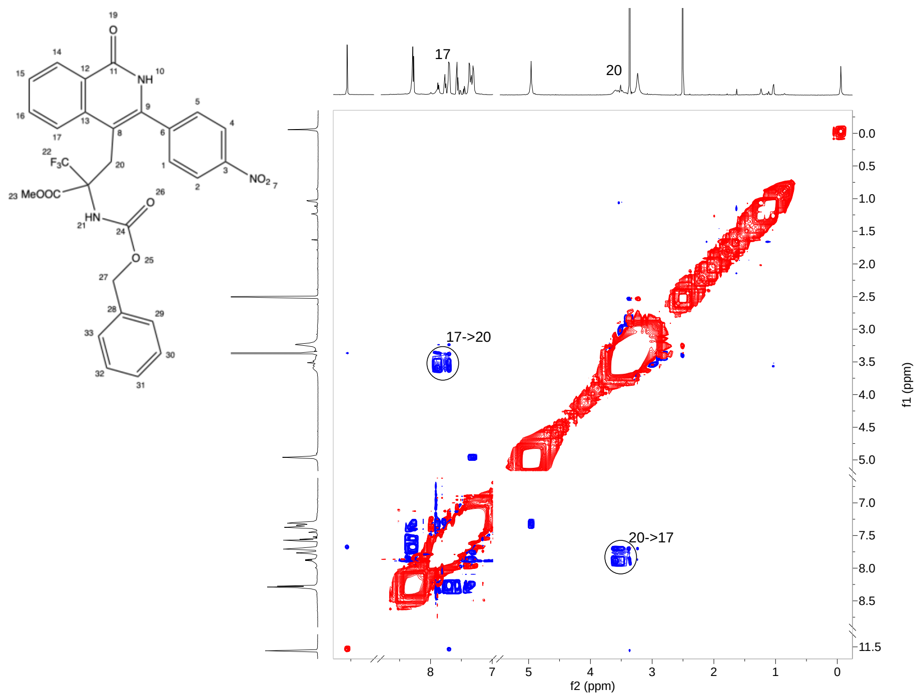

Figure S42. ROESY NMR of **3t** in DMSO-d_6

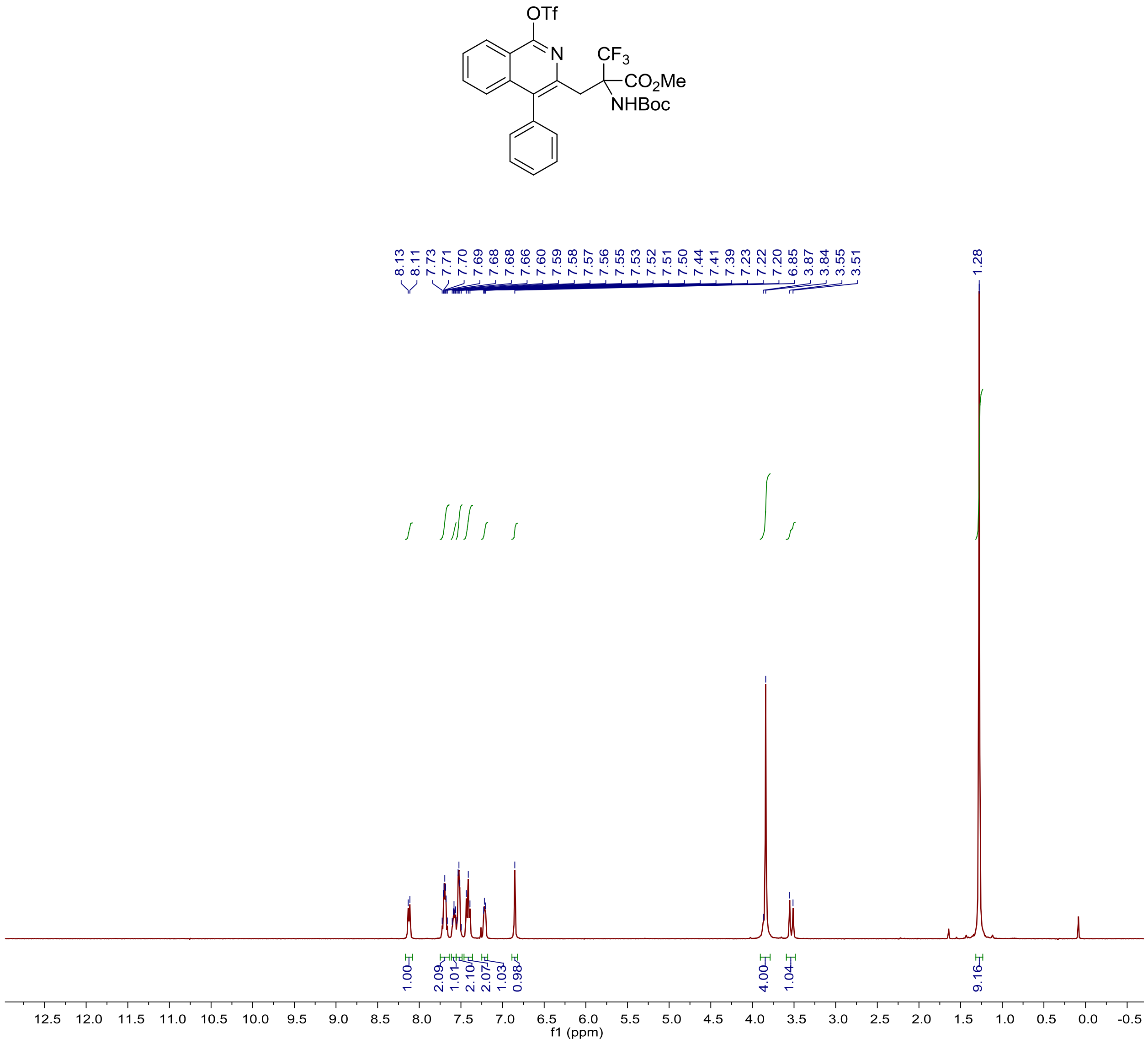

Figure S43. ^{1}H spectrum of **4a** in $CDCl_3$

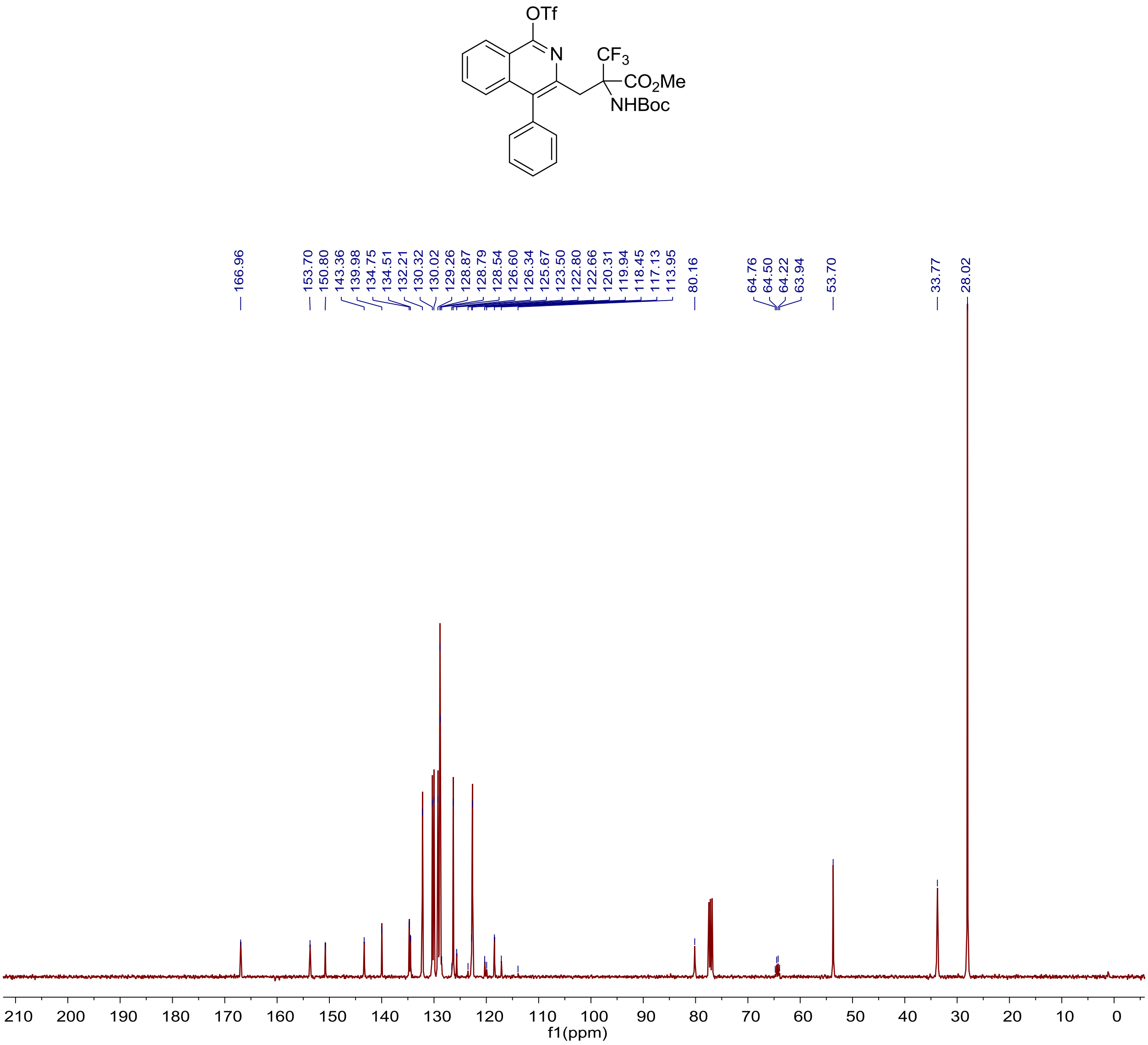

Figure S44. ^{13}C spectrum of **4a** in $CDCl_3$

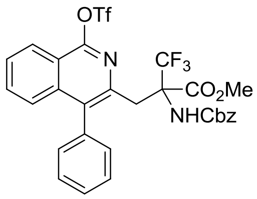

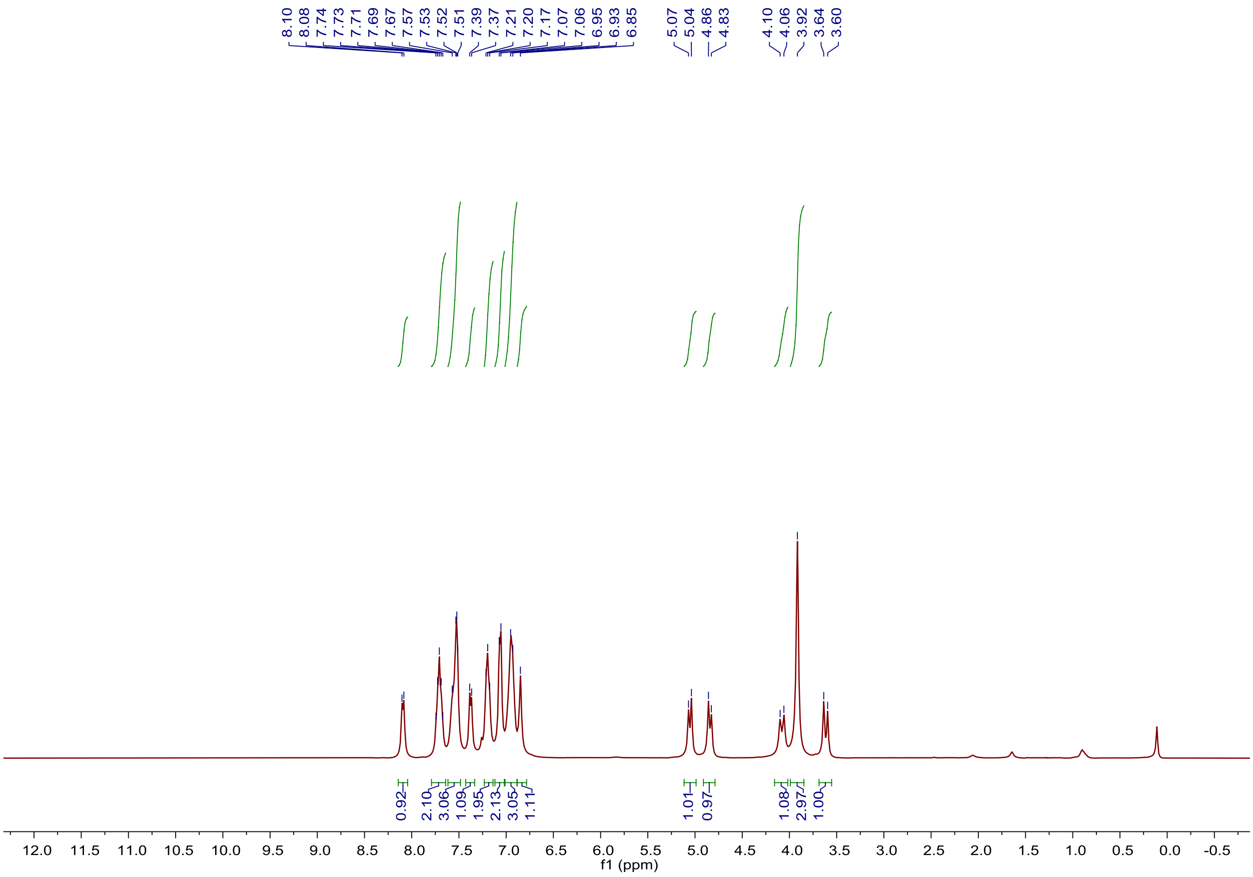

Figure S45. ^{1}H spectrum of **4b** in $CDCl_3$

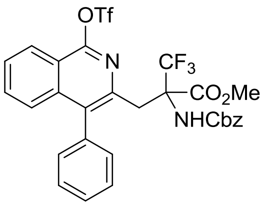

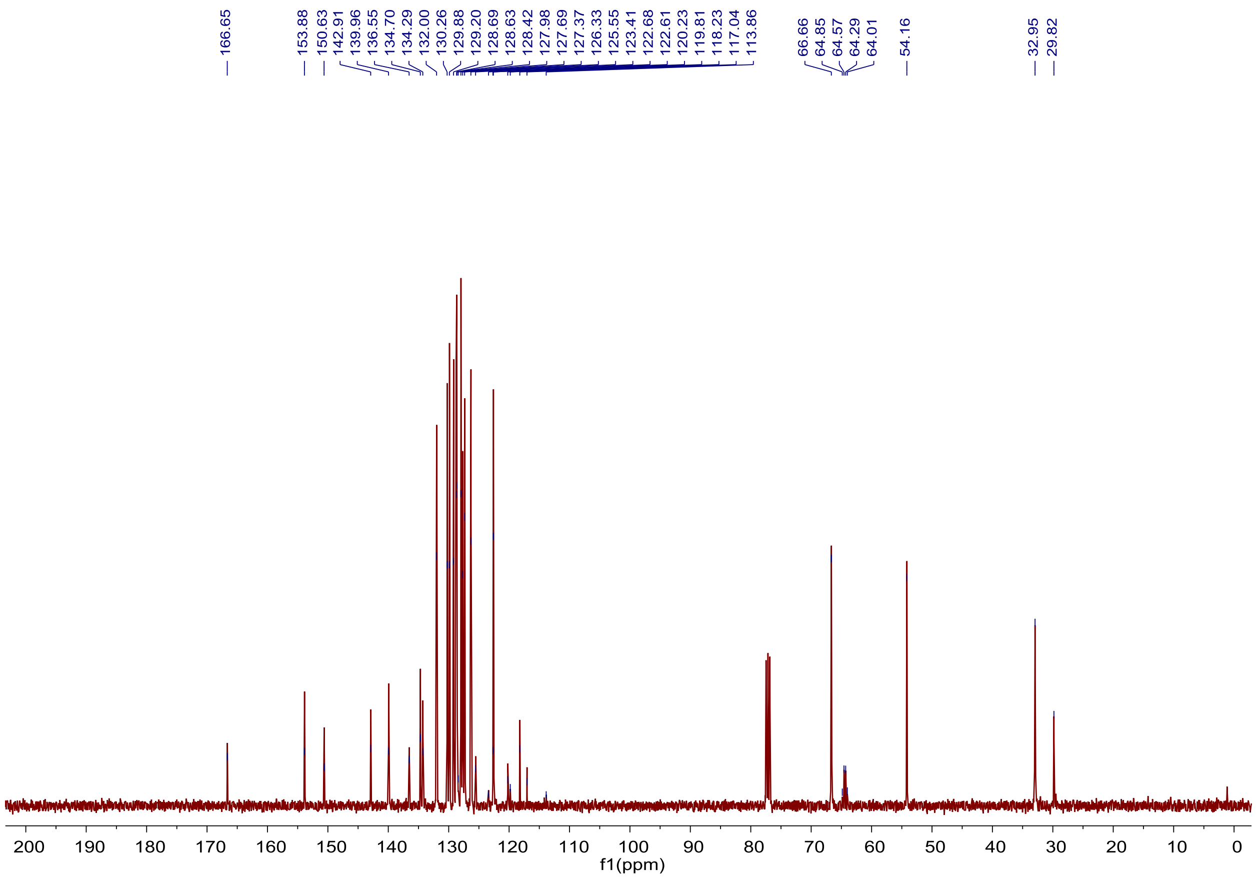

Figure S46. ^{13}C spectrum of **4b** in $CDCl_3$

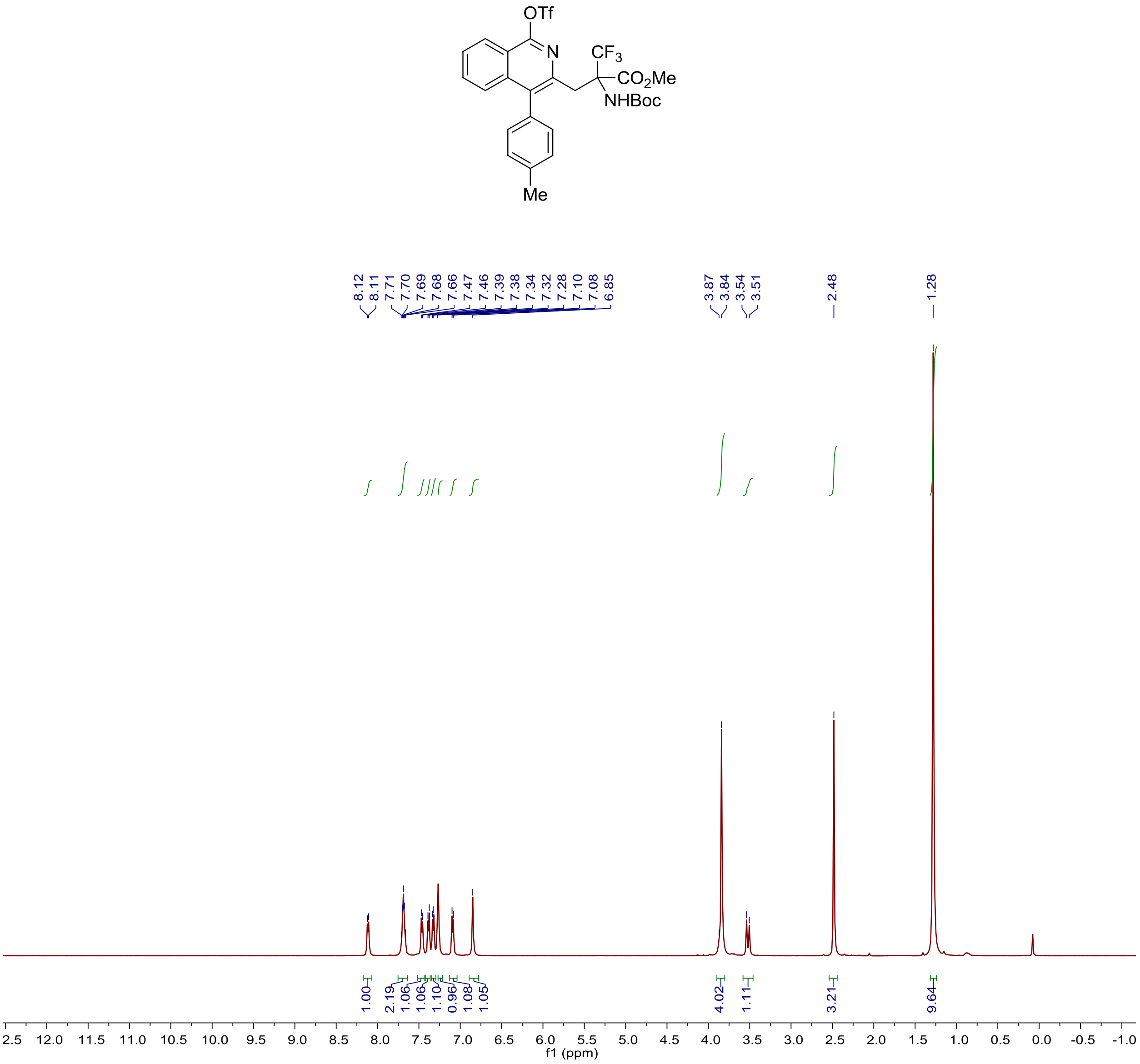

Figure S47. ^{1}H spectrum of **4c** in $CDCl_3$

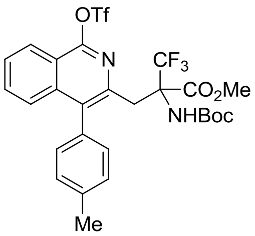

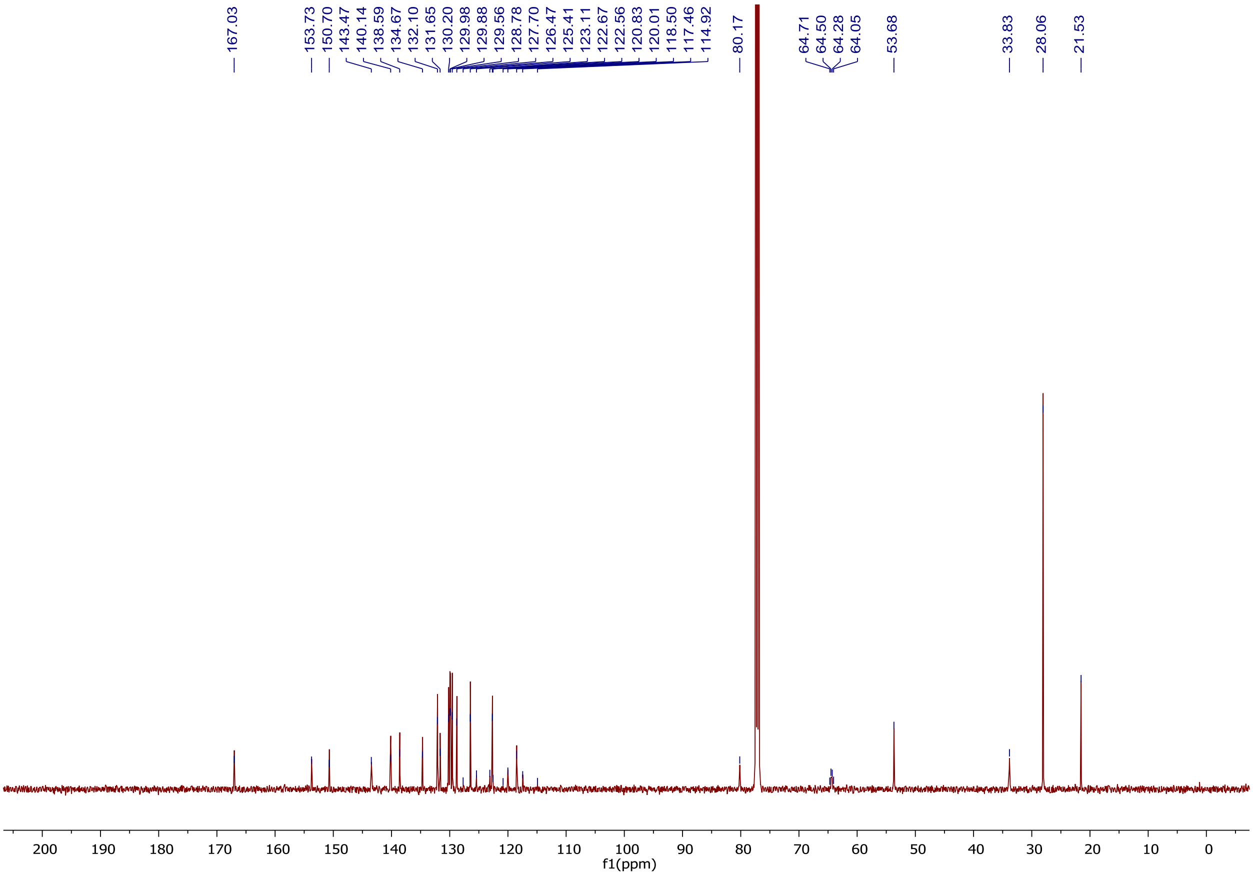

Figure S48. ^{13}C spectrum of **4c** in $CDCl_3$

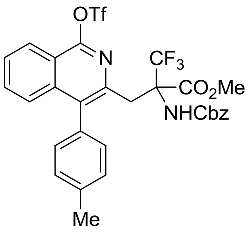

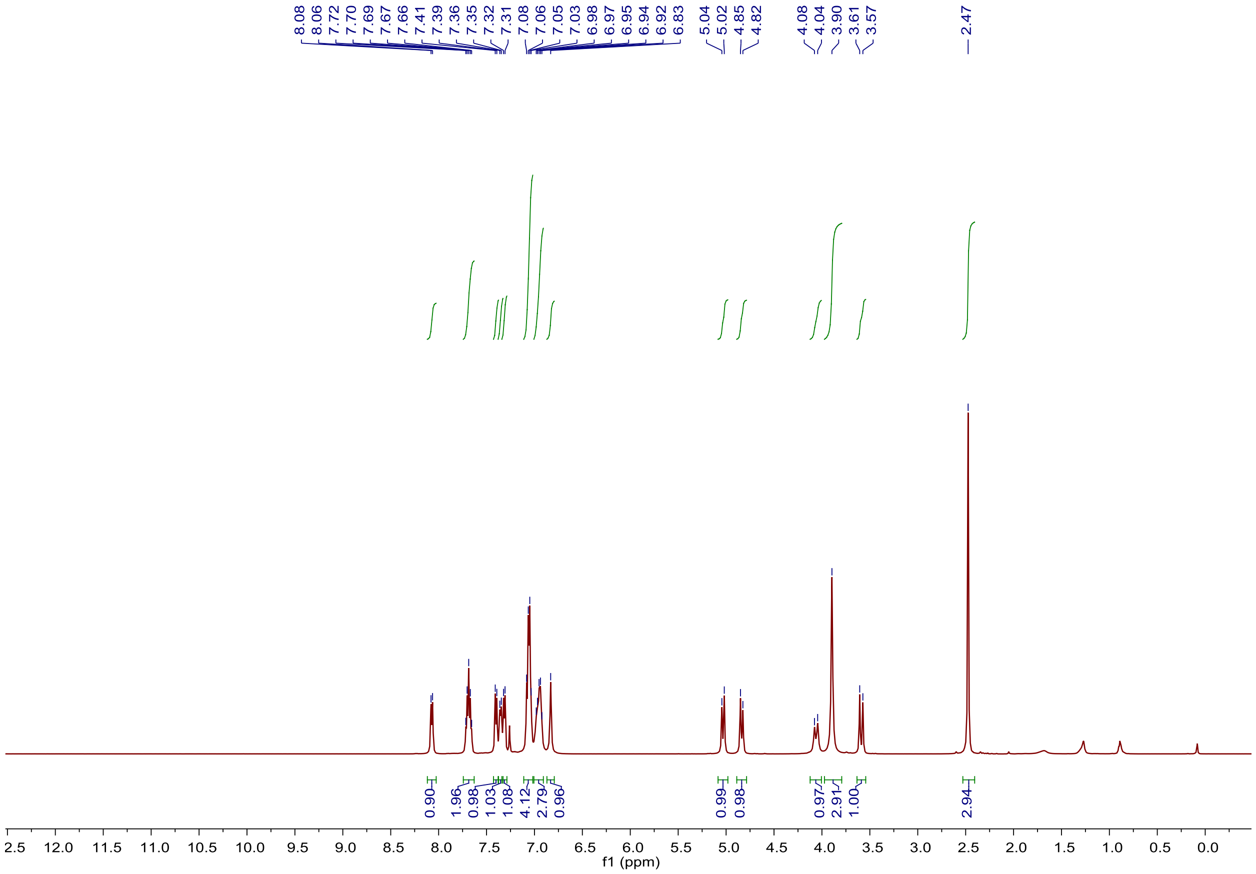

Figure S49. ^{1}H spectrum of **4d** in $CDCl_3$

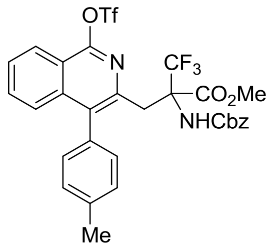

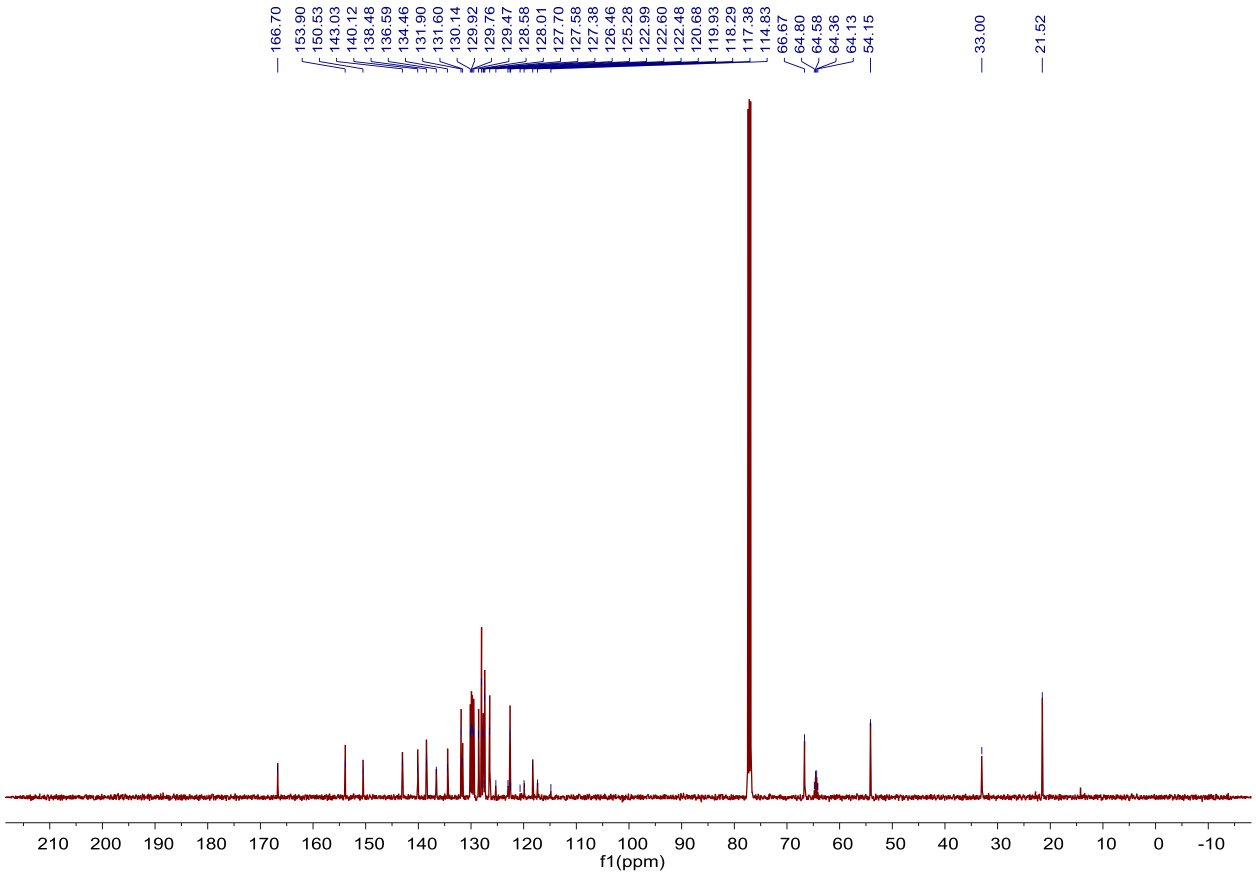

Figure S50. ^{13}C spectrum of **4d** in $CDCl_3$

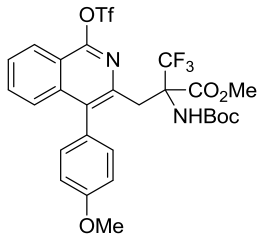

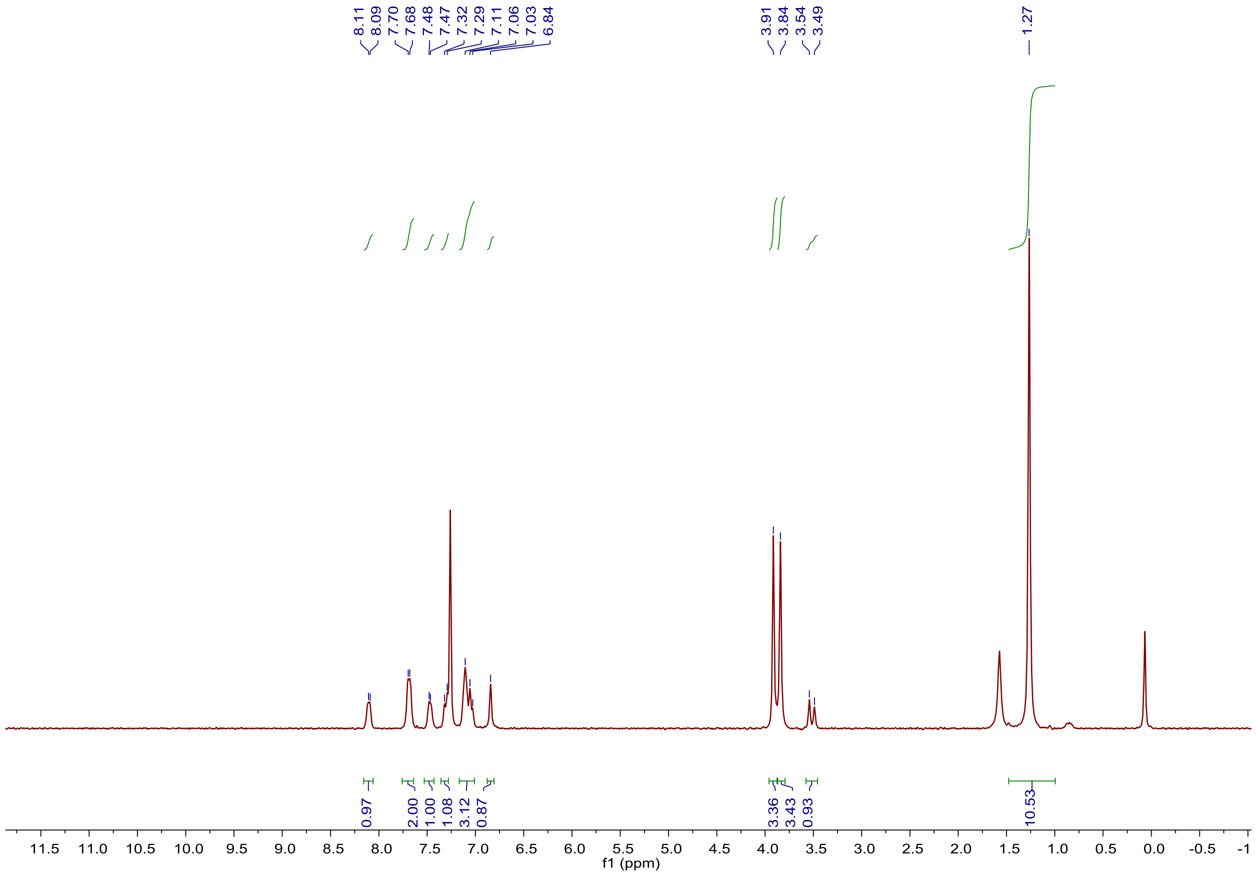

Figure S51. ^{1}H spectrum of **4e** in $CDCl_3$

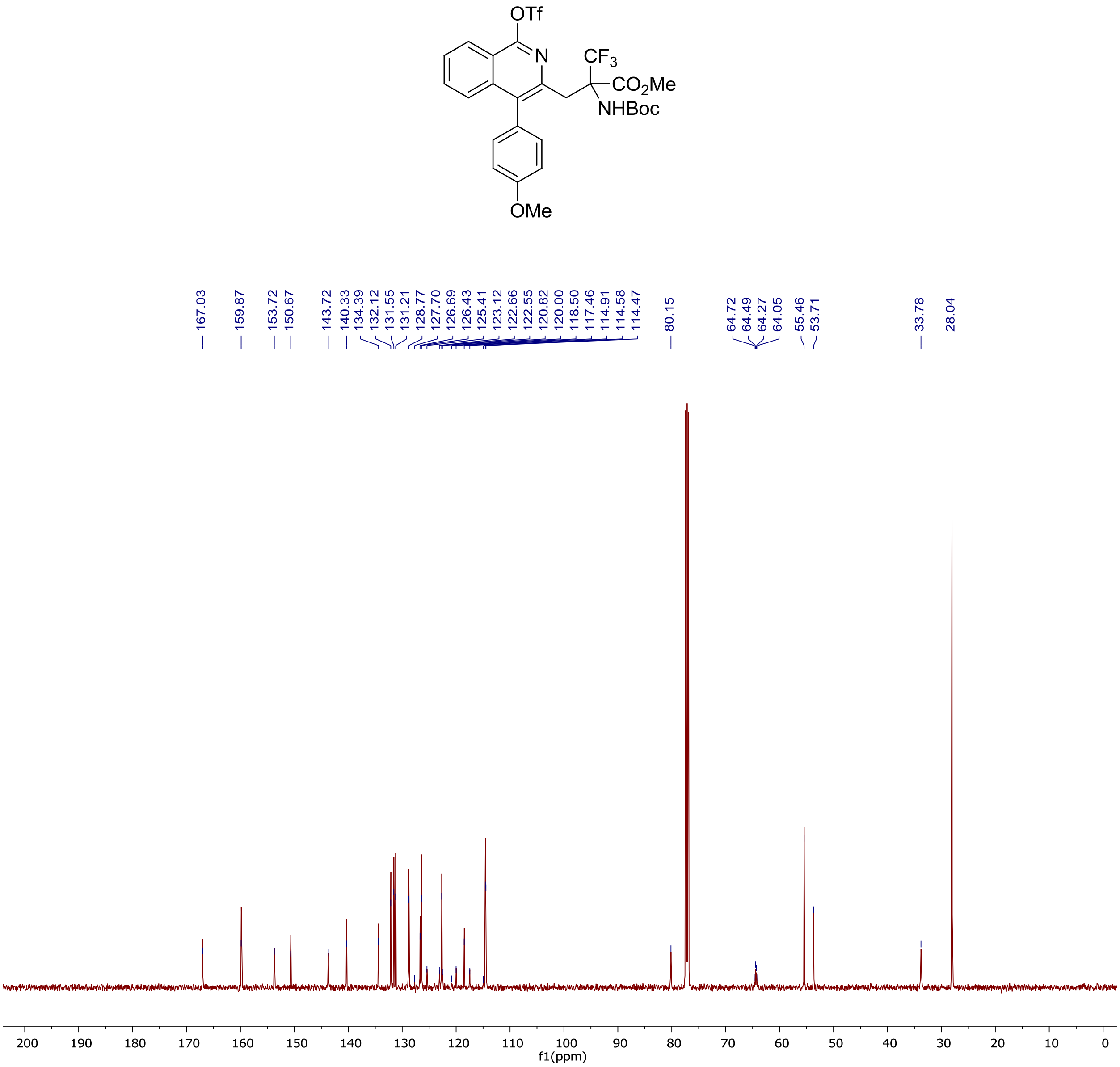

Figure S52. ^{13}C spectrum of **4e** in $CDCl_3$

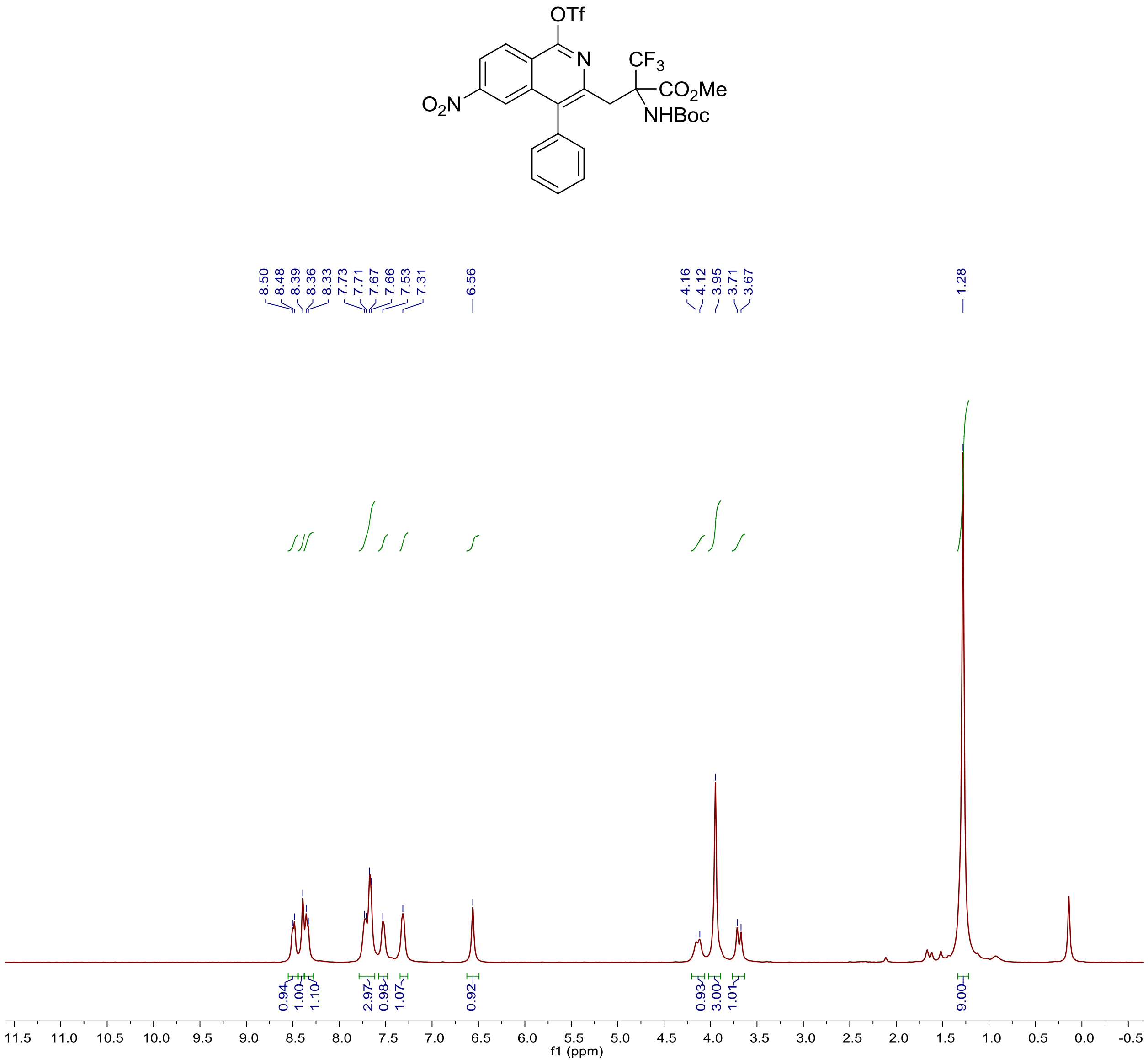

Figure S53. ^{1}H spectrum of **4f** in $CDCl_3$

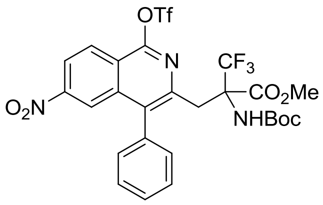

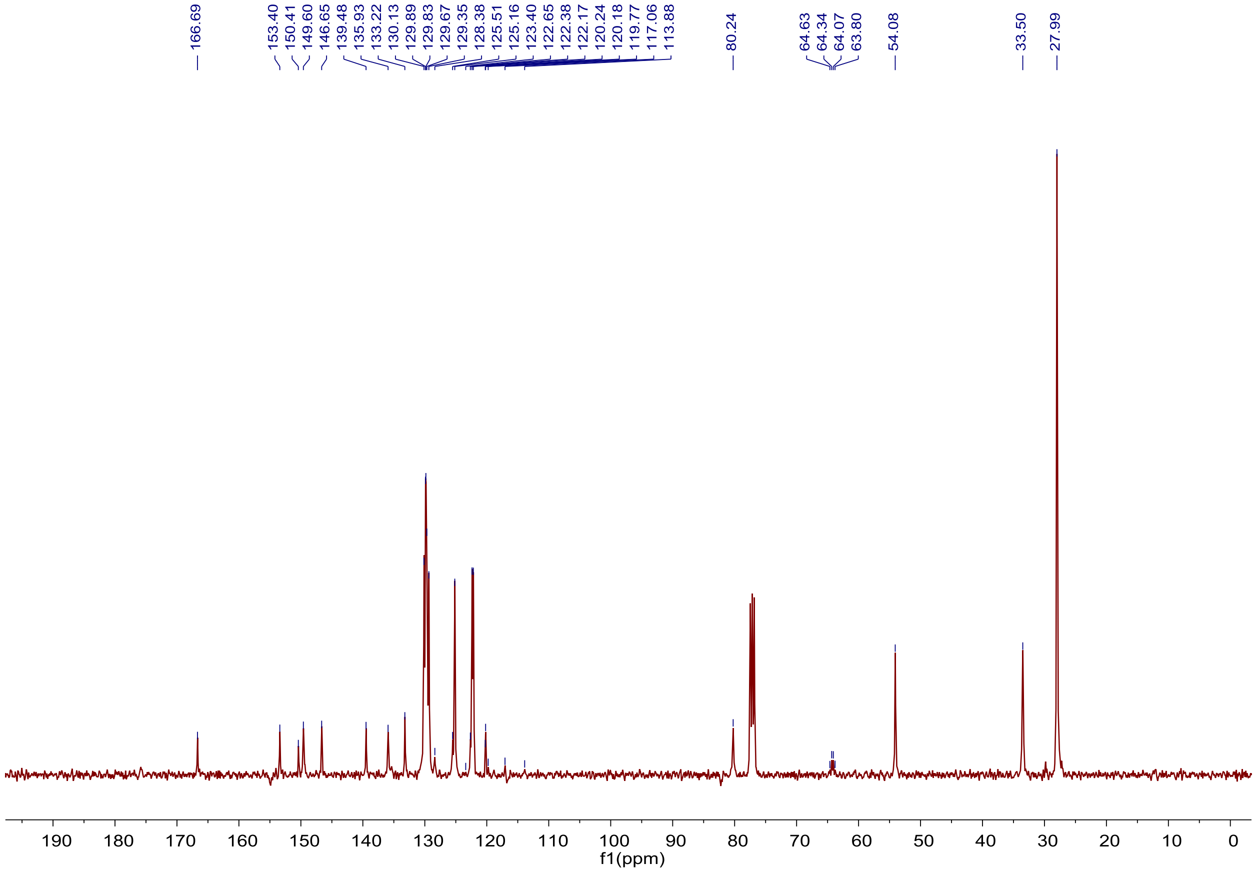

Figure S54. ^{13}C spectrum of **4f** in $CDCl_3$

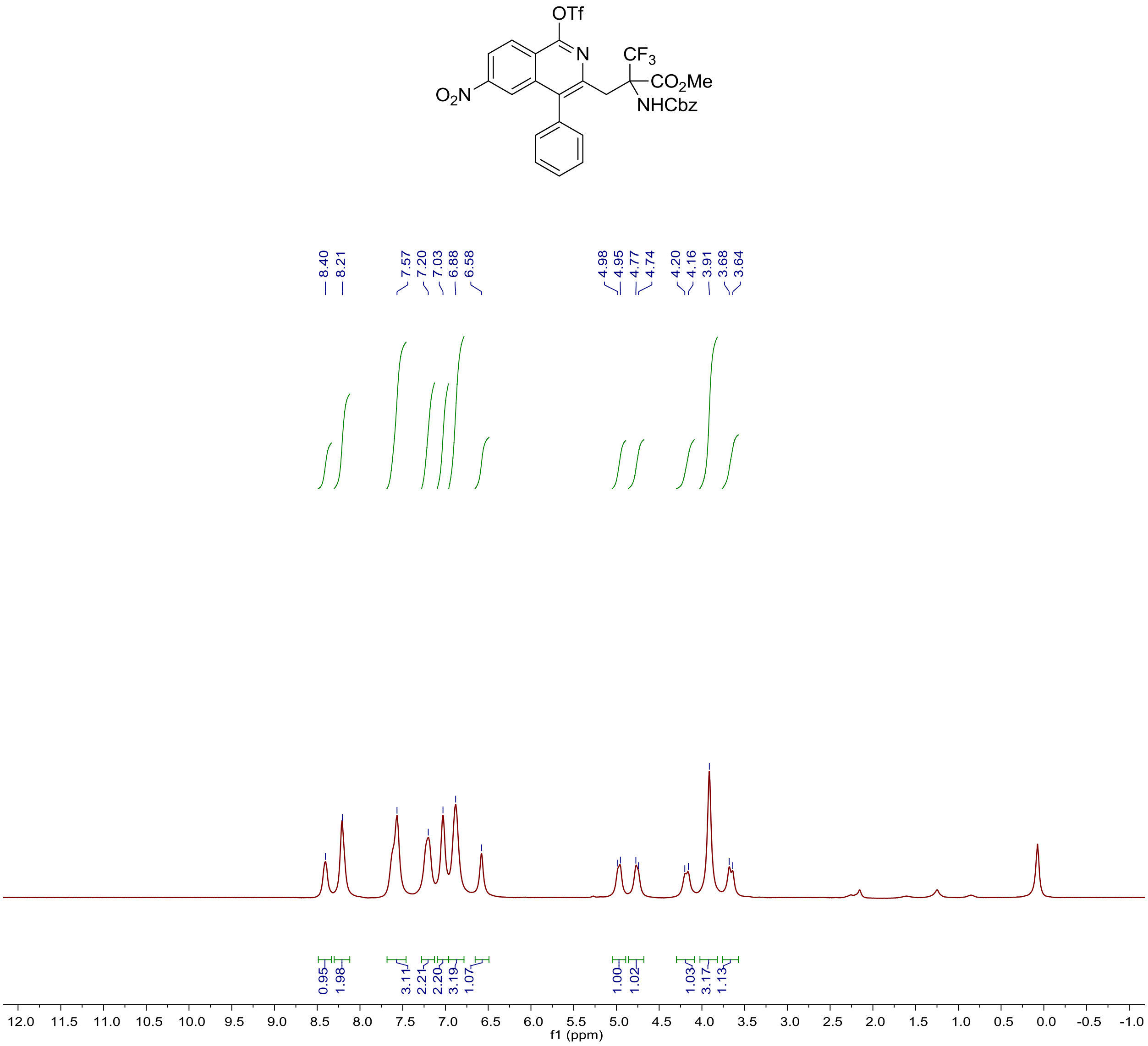

Figure S55. ^{1}H spectrum of **4g** in $CDCl_3$

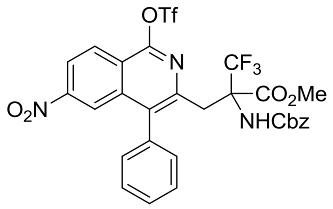

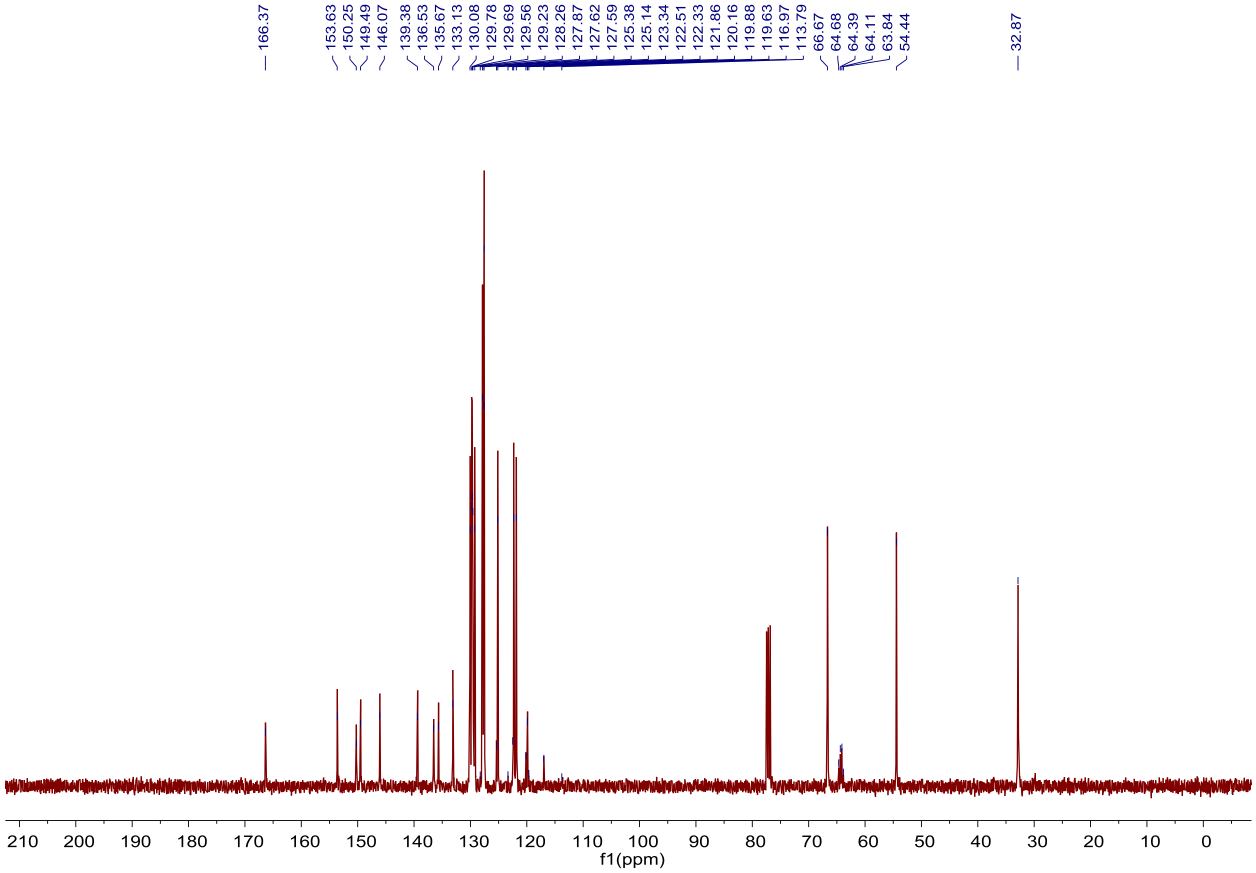

Figure S56. ^{13}C spectrum of **4g** in $CDCl_3$

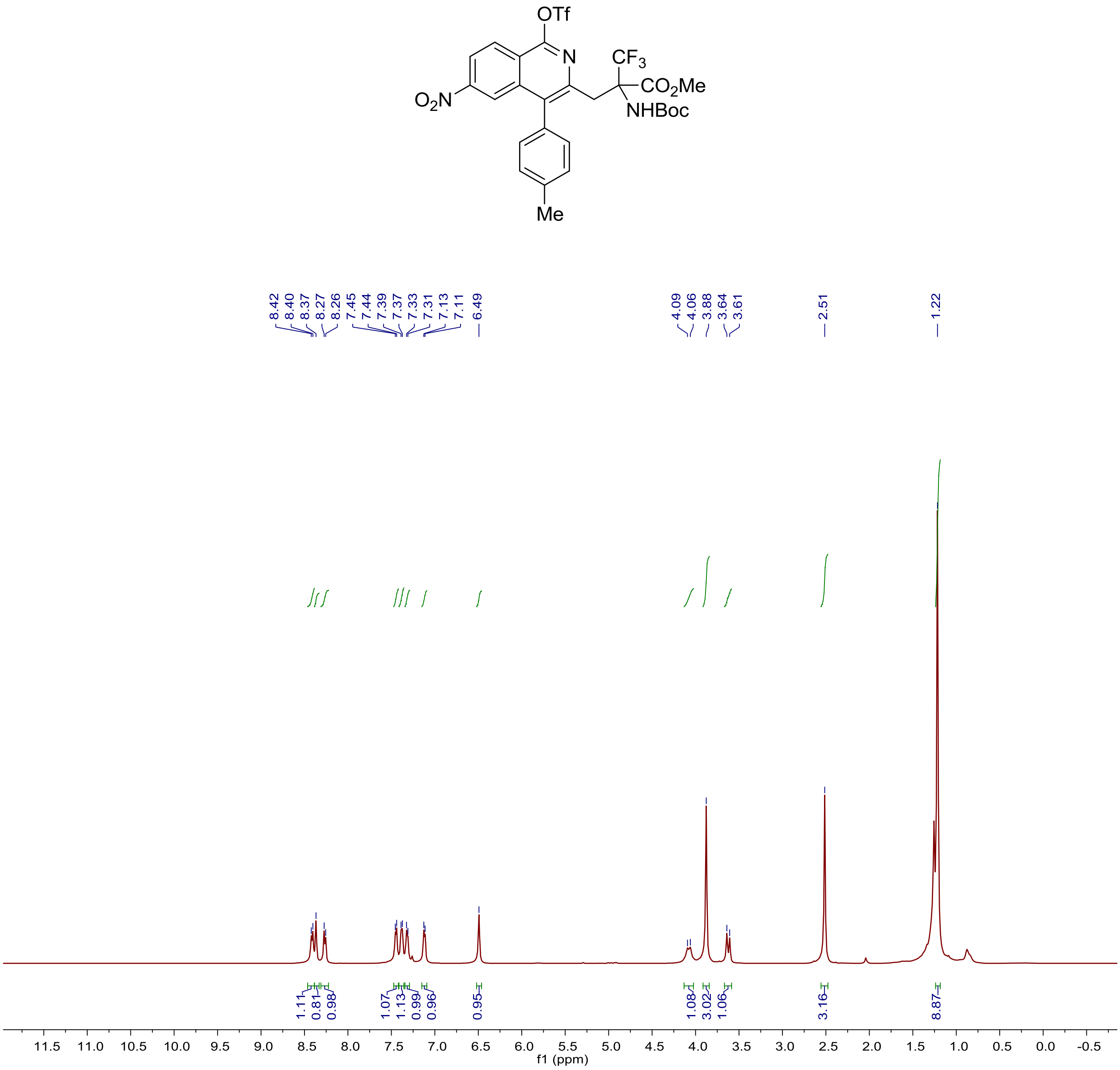

Figure S57. ^{1}H spectrum of **4h** in $CDCl_3$

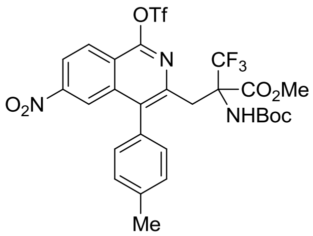

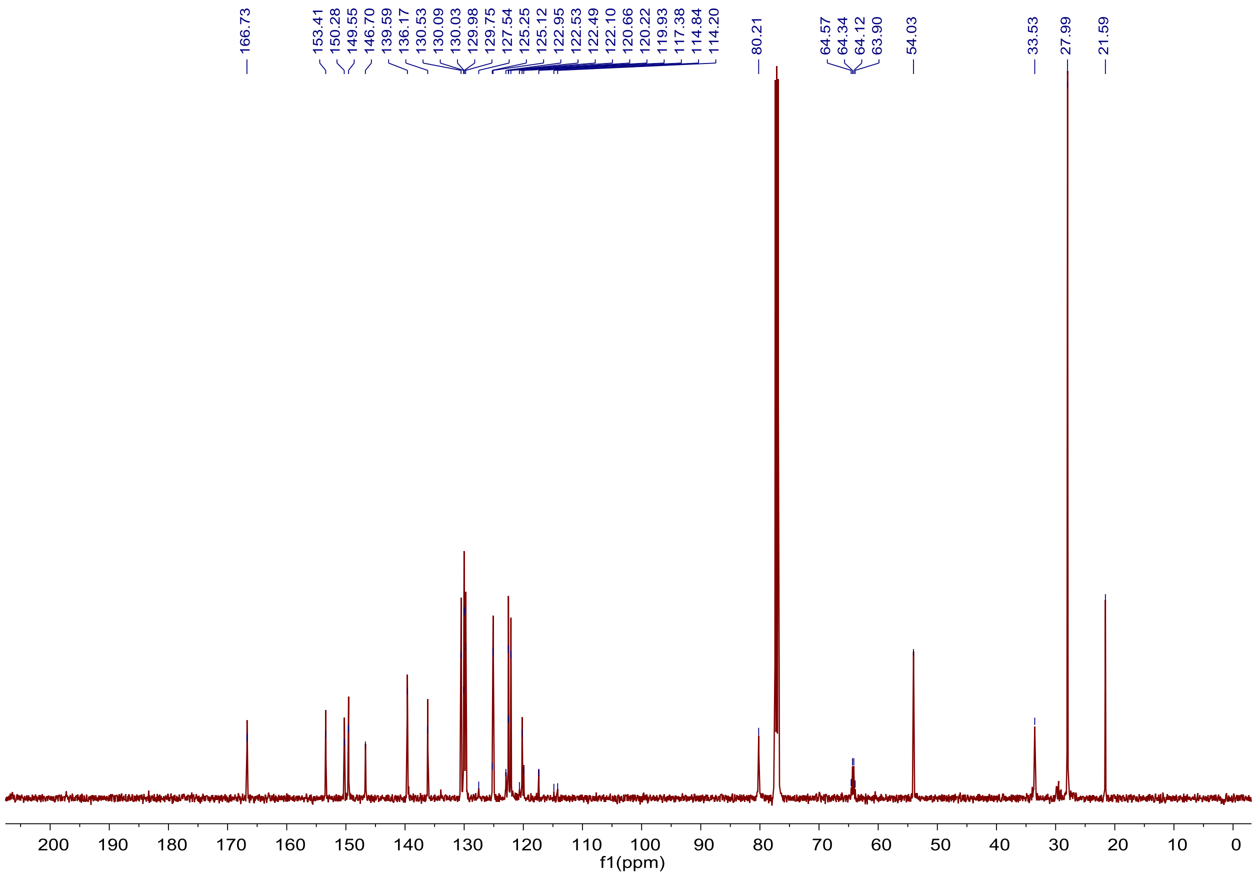

Figure S58. ^{13}C spectrum of **4h** in $CDCl_3$

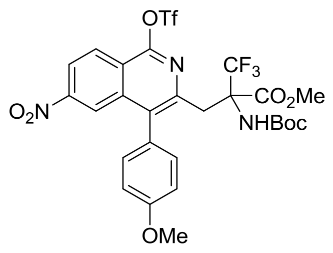

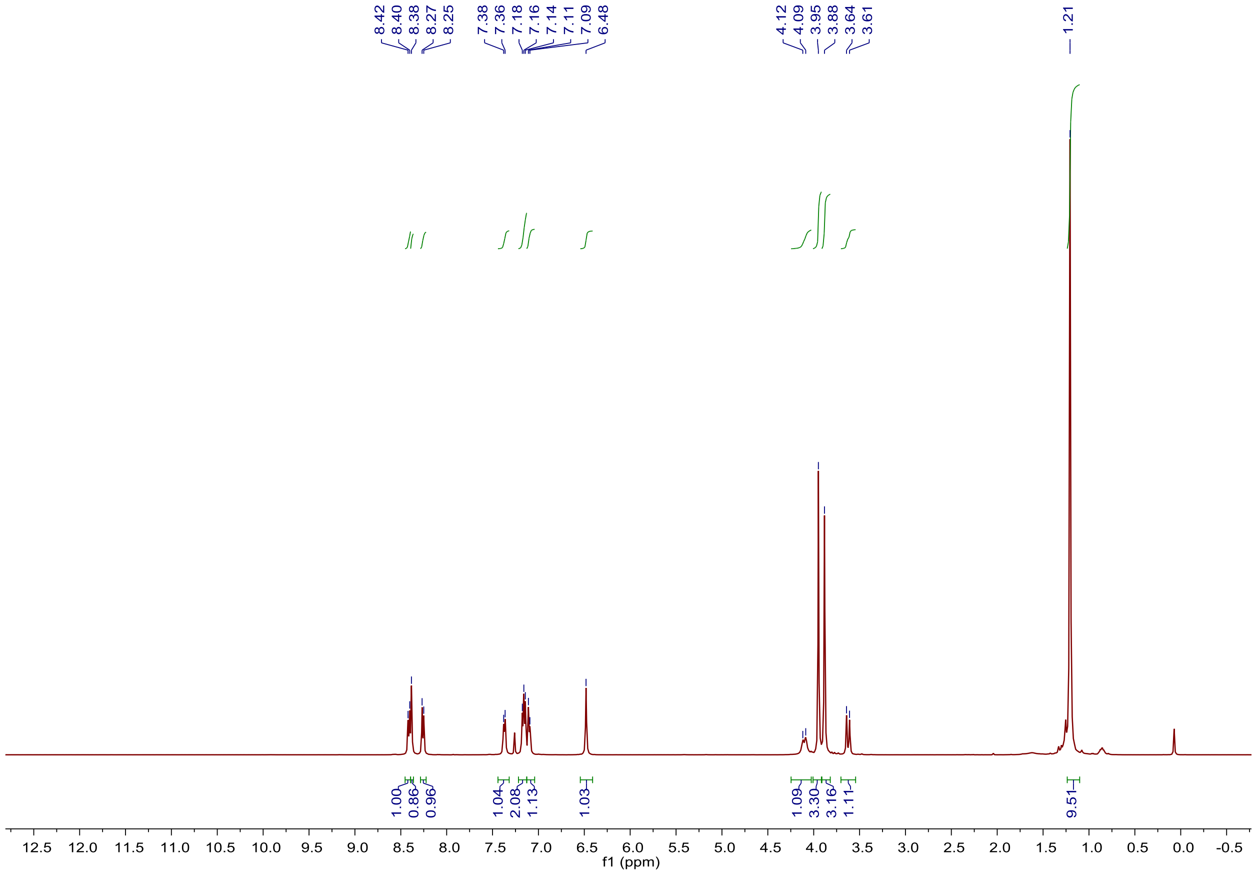

Figure S59. ^{1}H spectrum of **4i** in $CDCl_3$

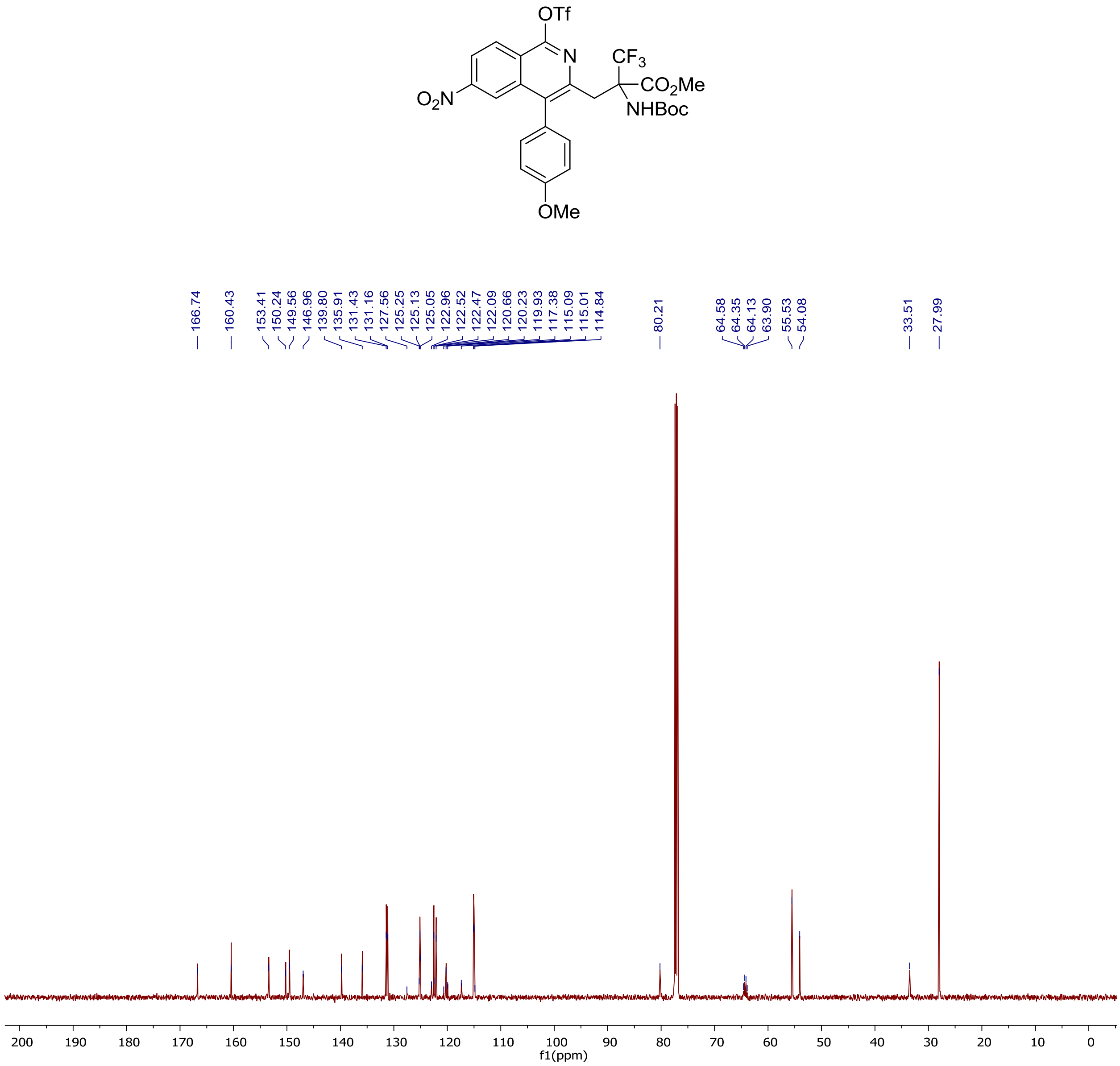

Figure S60. ^{13}C spectrum of **4i** in $CDCl_3$

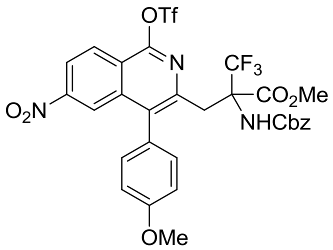

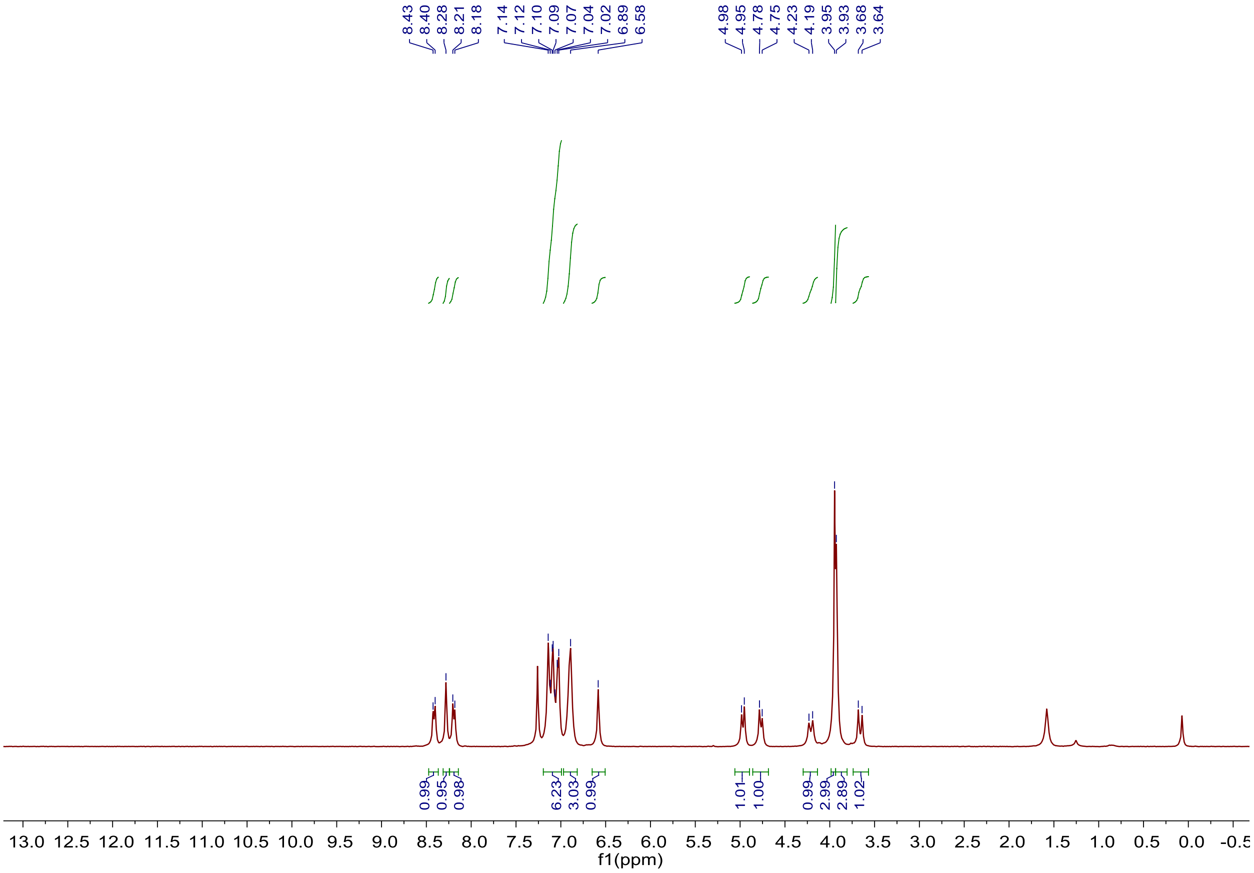

Figure S61. ^{1}H spectrum of **4j** in $CDCl_3$

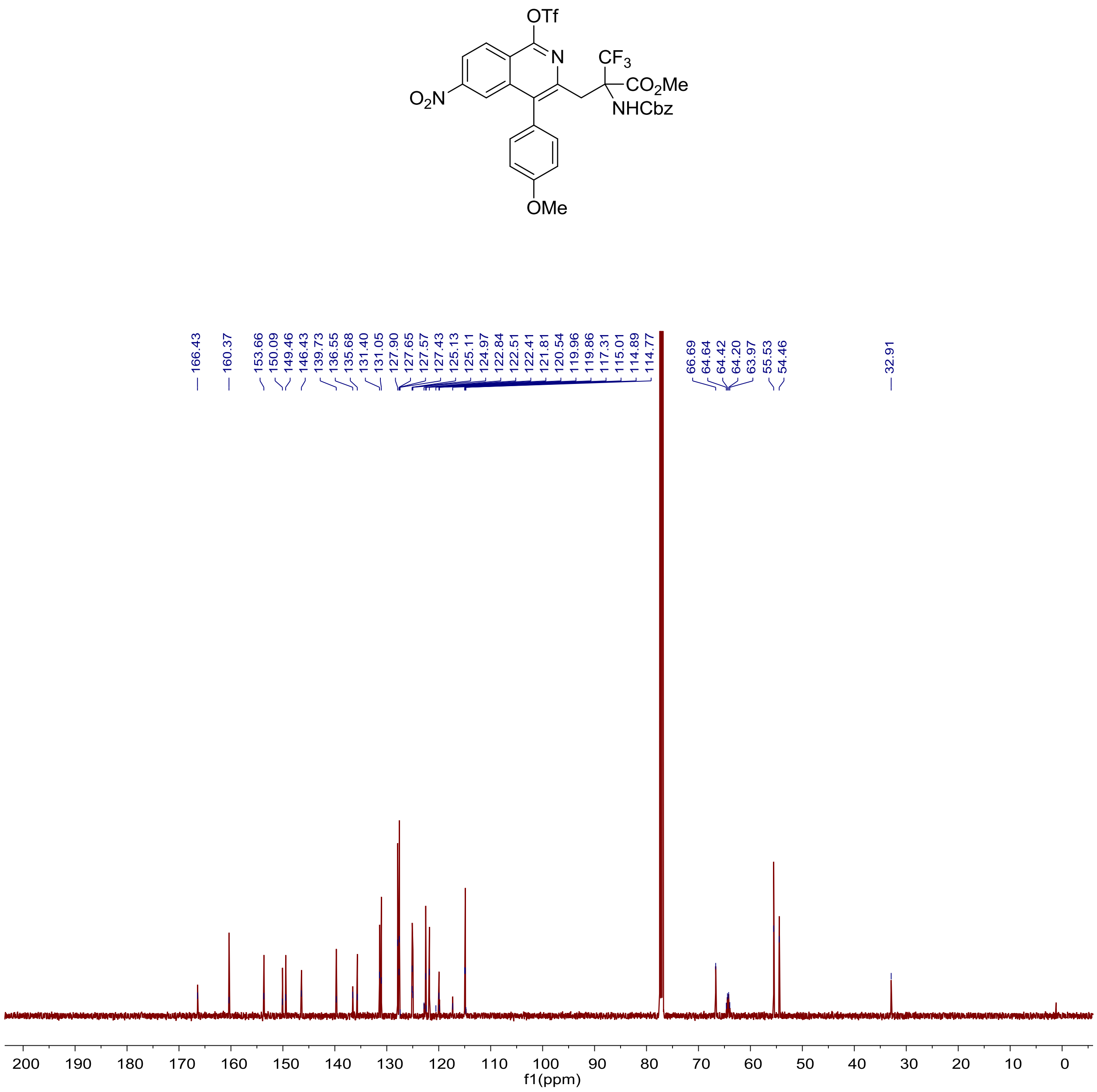

Figure S62. ^{13}C spectrum of **4j** in $CDCl_3$

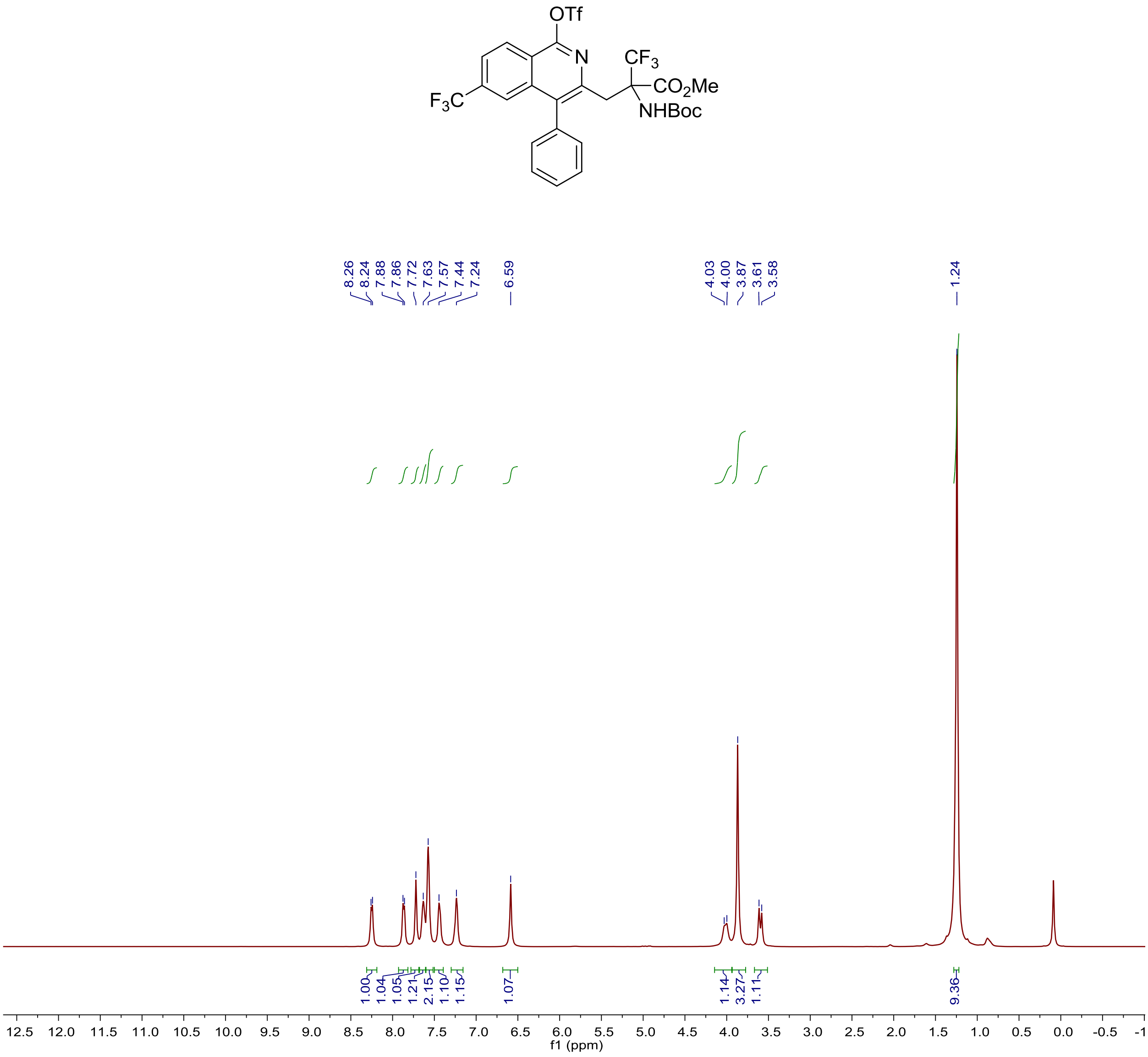

Figure S63. ^{1}H spectrum of **4k** in $CDCl_3$

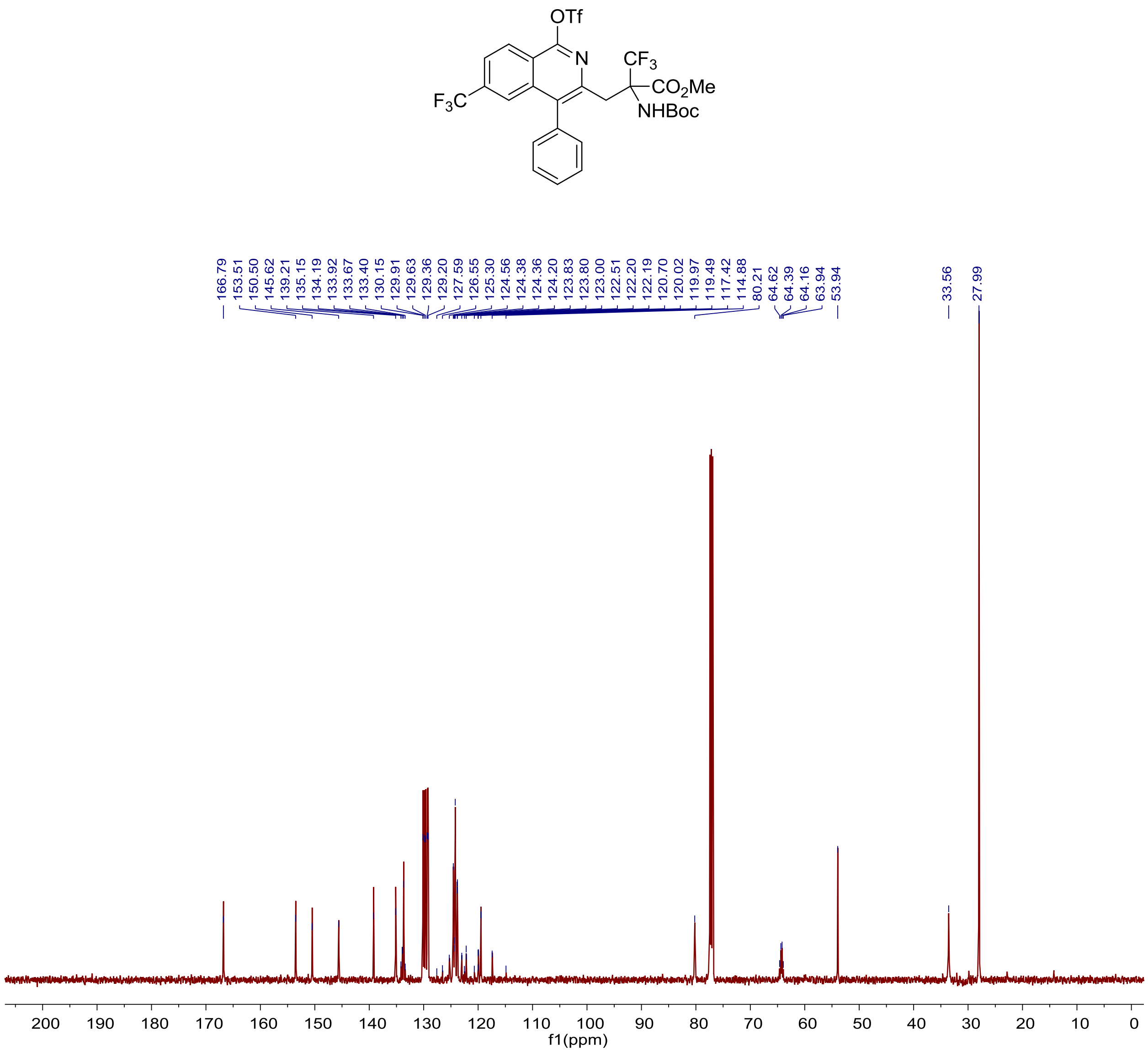

Figure S64. ^{13}C spectrum of **4k** in $CDCl_3$

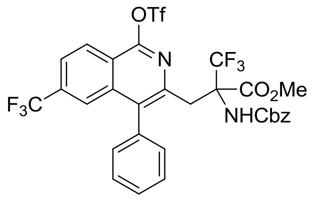

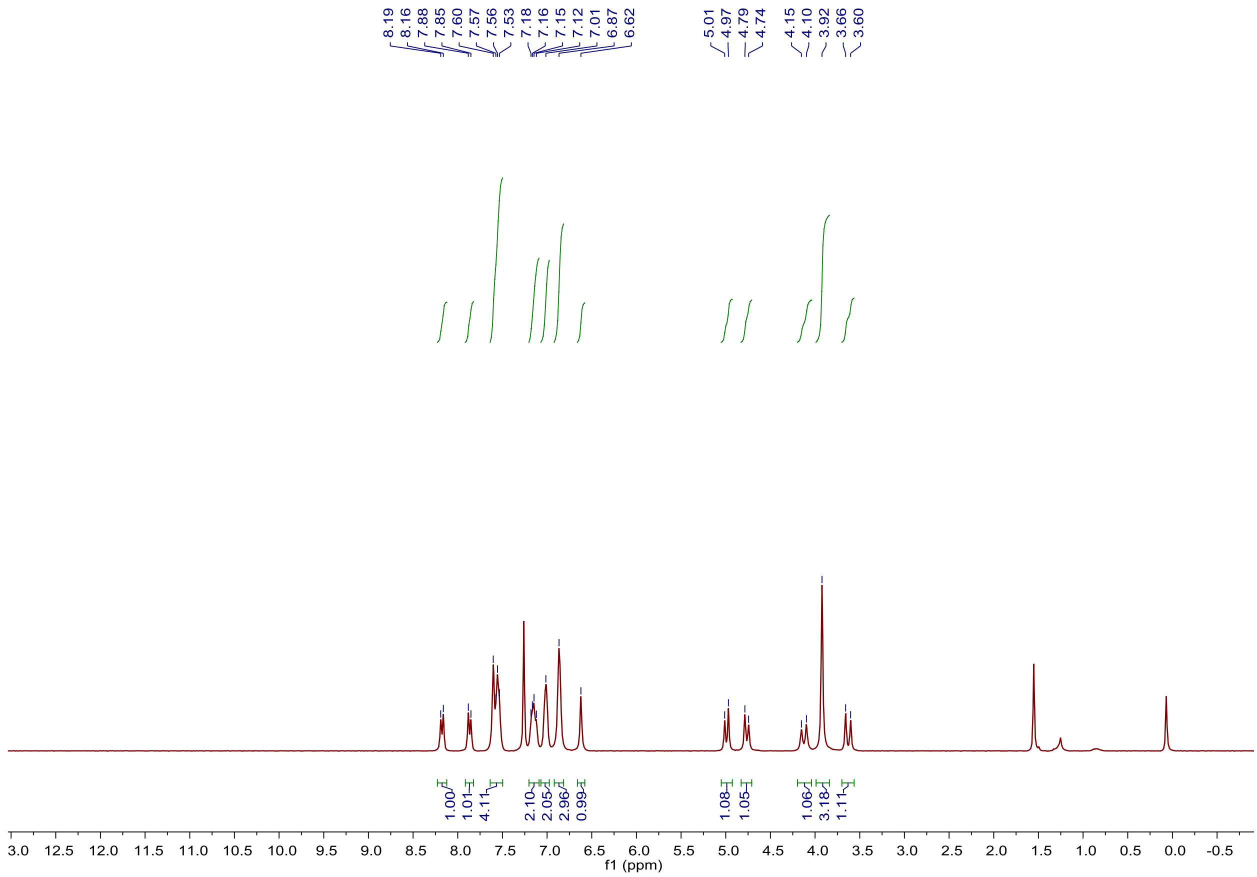

Figure S65. ^{1}H spectrum of **4l** in $CDCl_3$

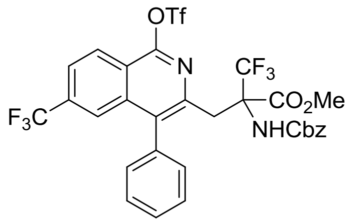

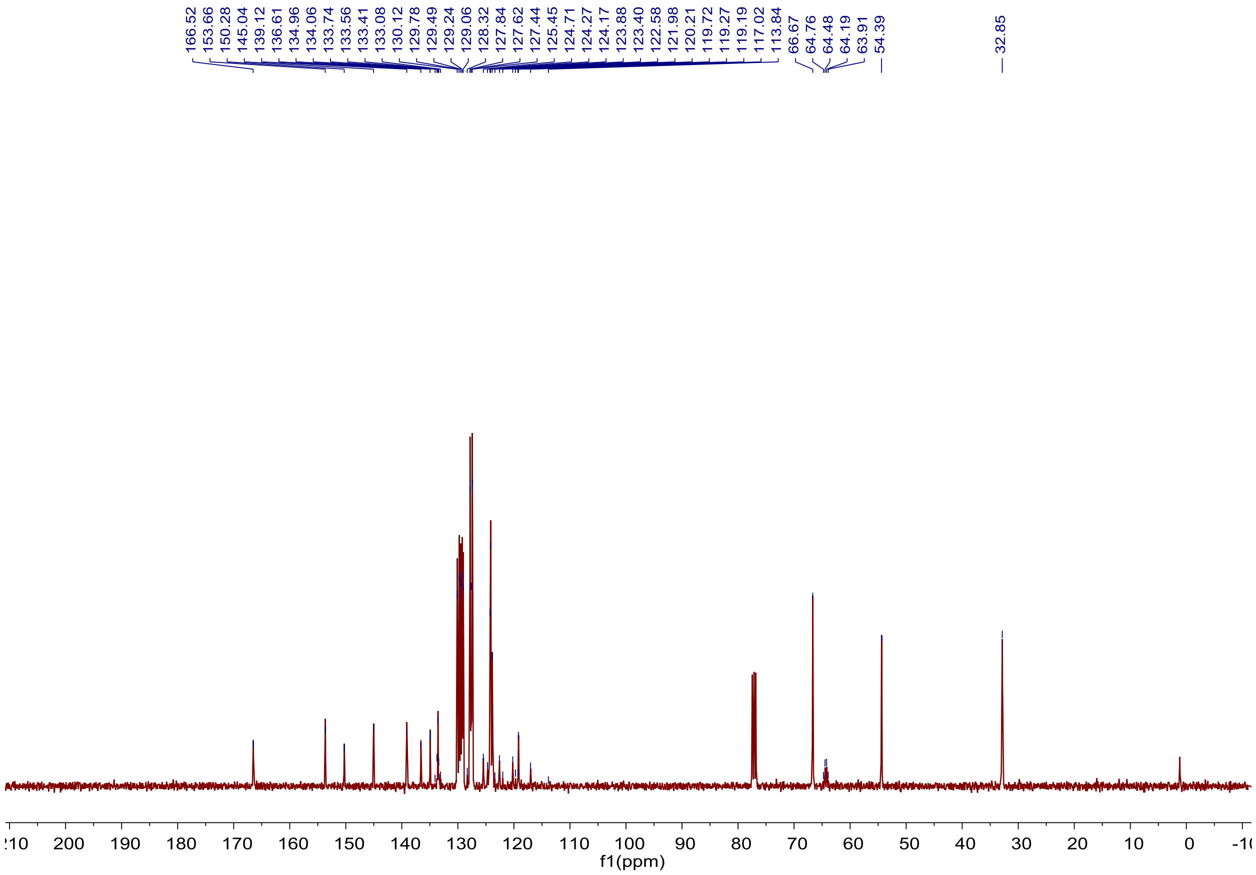

Figure S66. ^{13}C spectrum of **4l** in $CDCl_3$

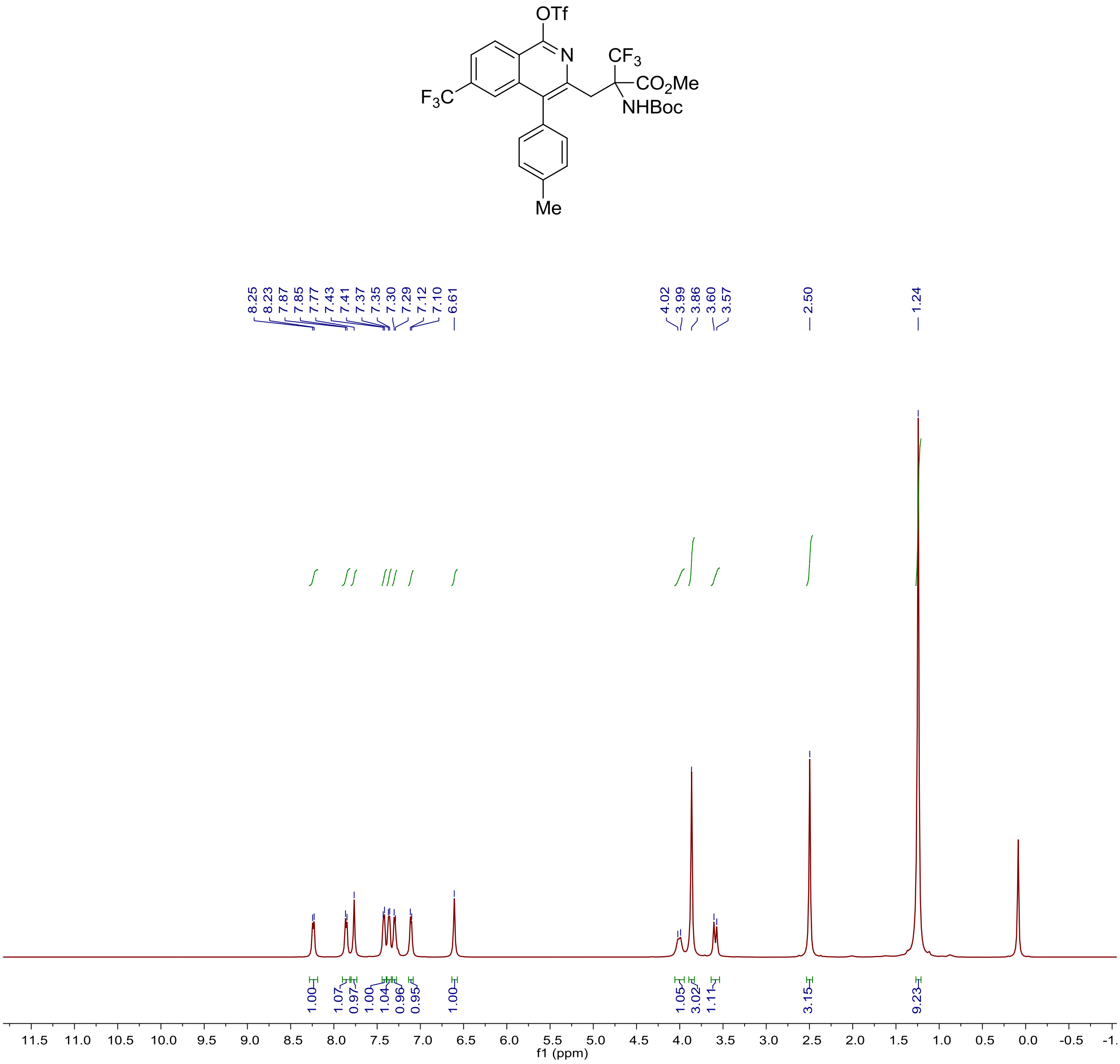

Figure S67. ^{1}H spectrum of **4m** in $CDCl_3$

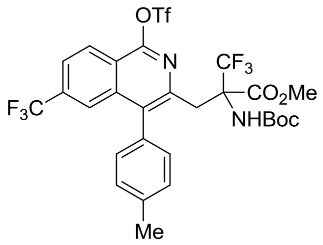

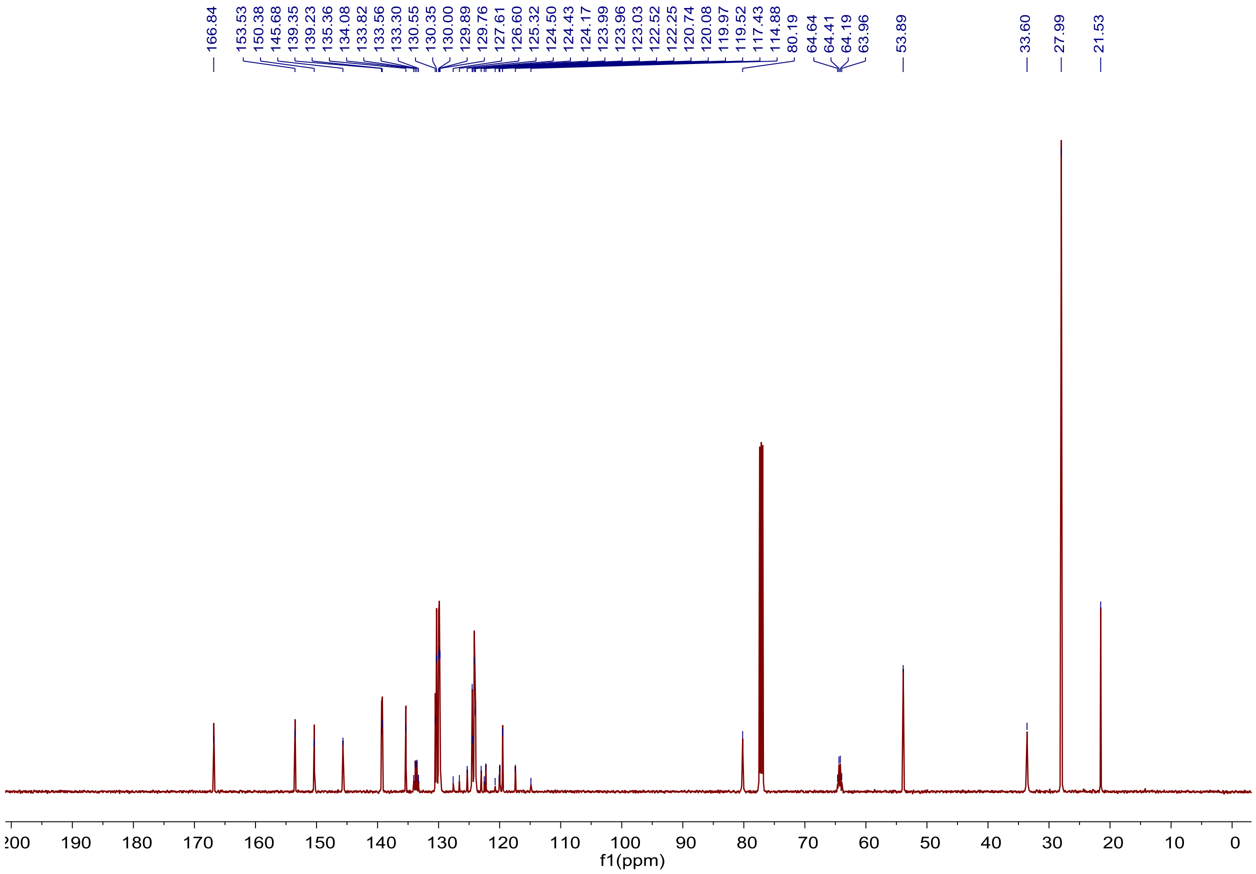

Figure S68. ^{13}C spectrum of **4m** in $CDCl_3$

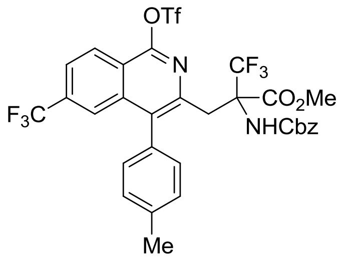

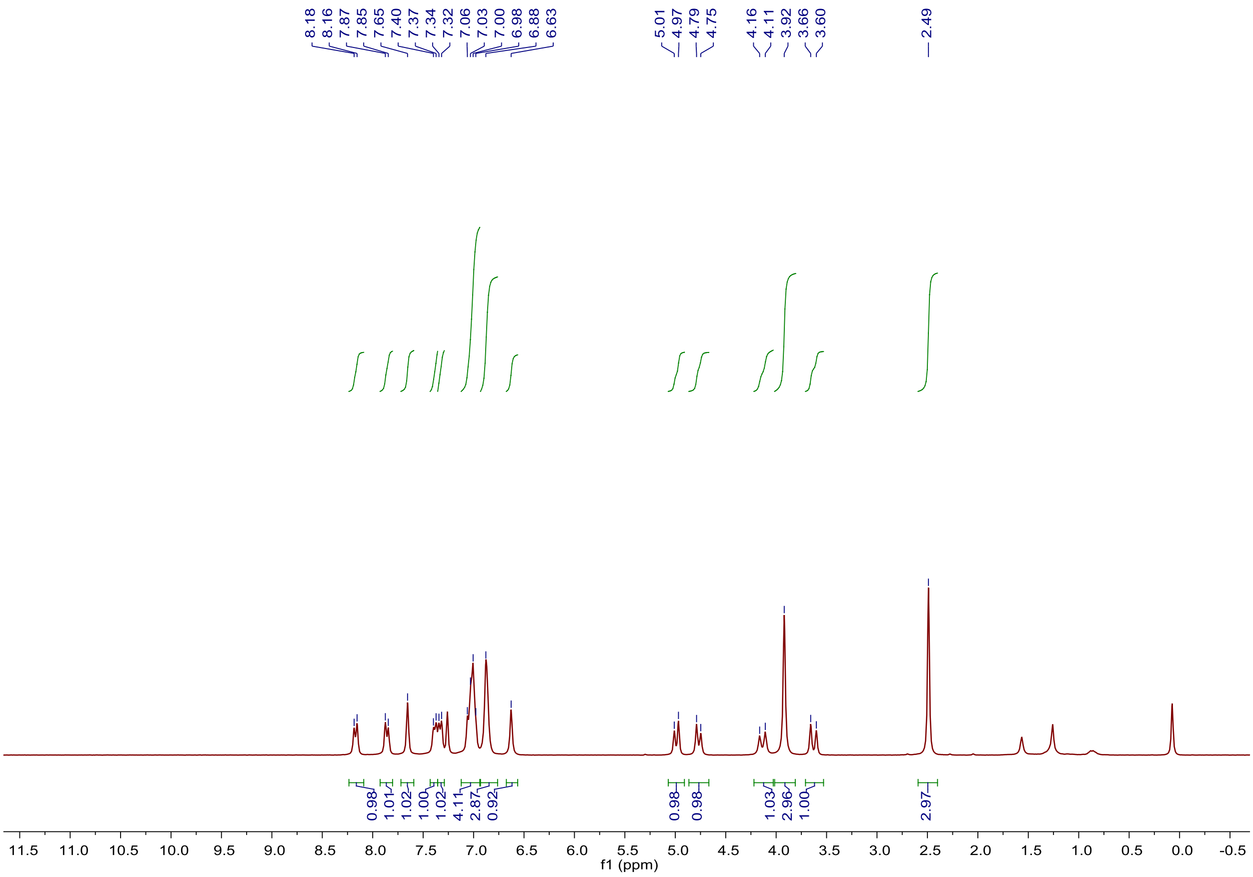

Figure S69. ^{1}H spectrum of **4n** in $CDCl_3$

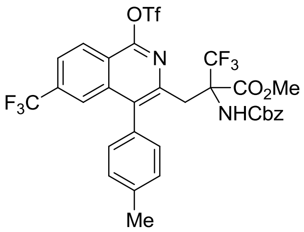

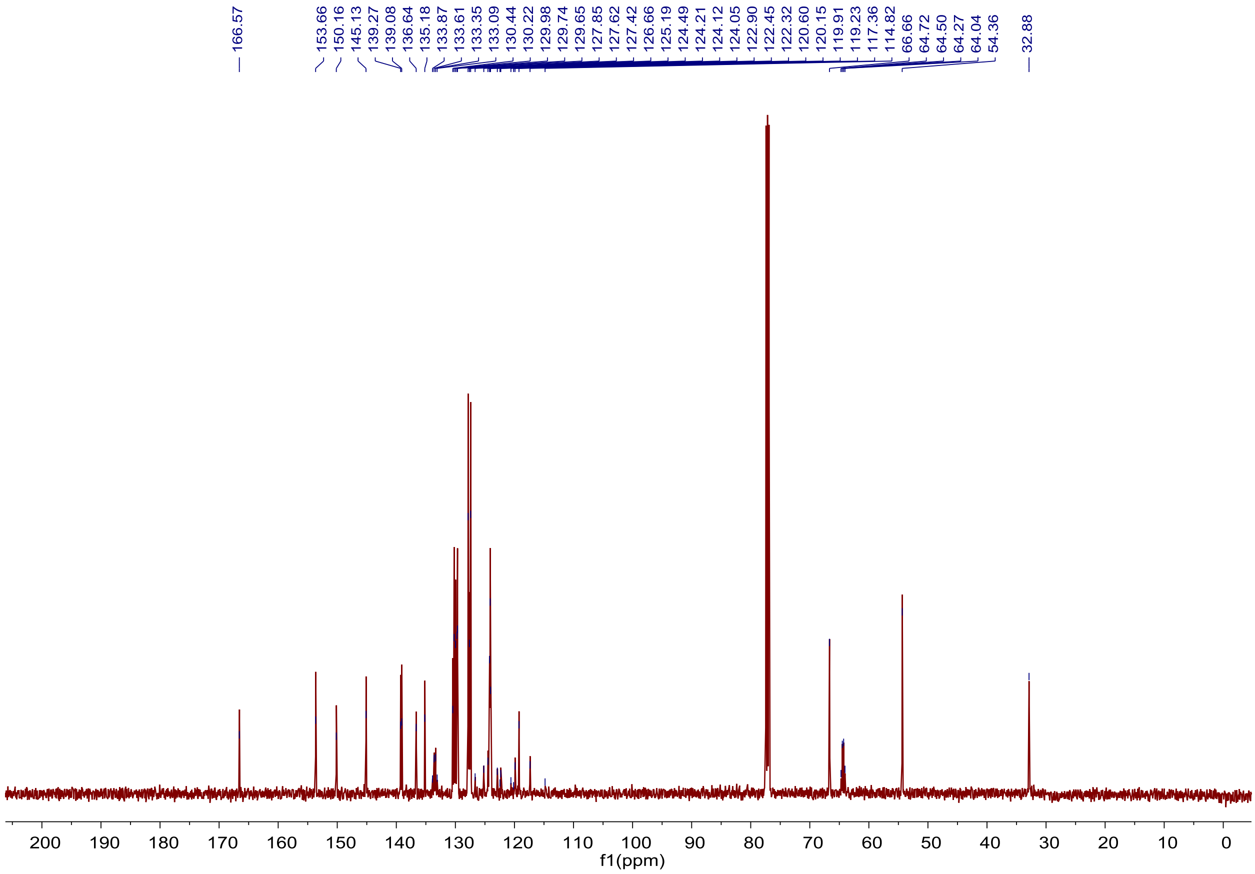

Figure S70. ^{13}C spectrum of **4n** in $CDCl_3$

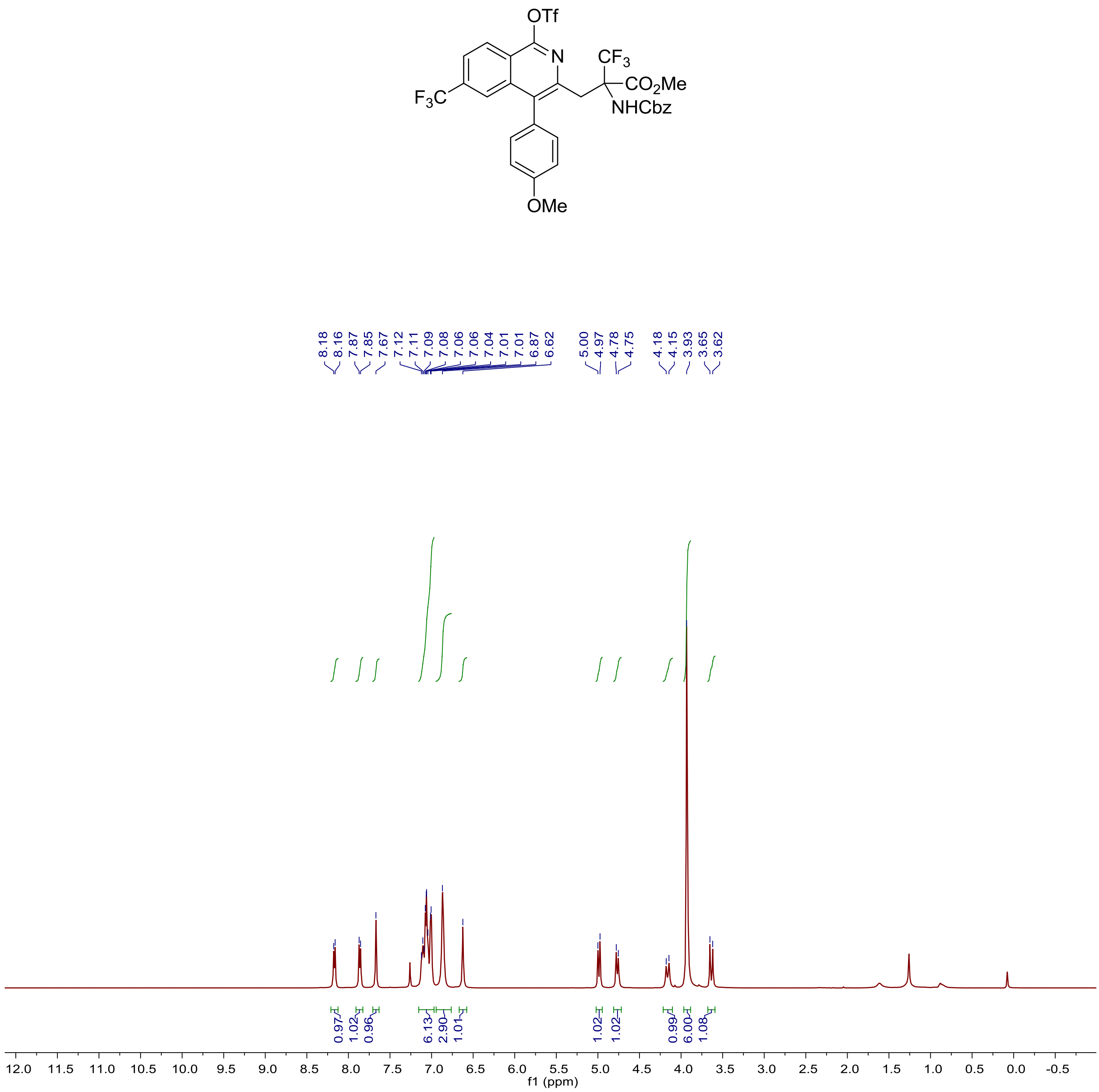

Figure S71. ^{1}H spectrum of **4o** in $CDCl_3$

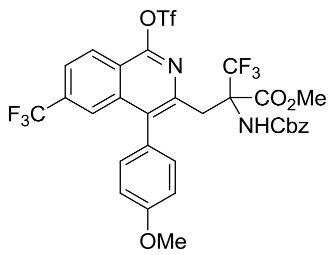

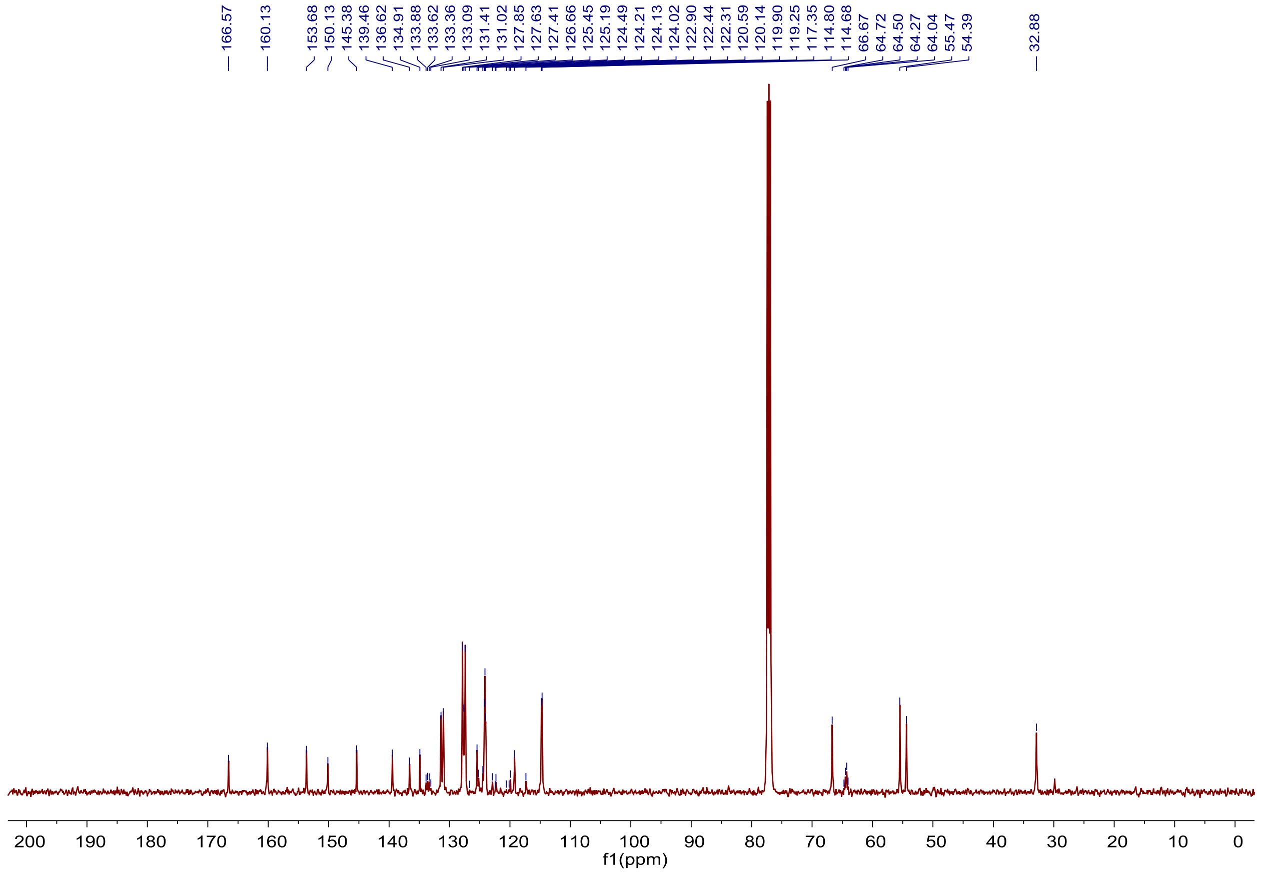

Figure S72. ^{13}C spectrum of **4o** in $CDCl_3$

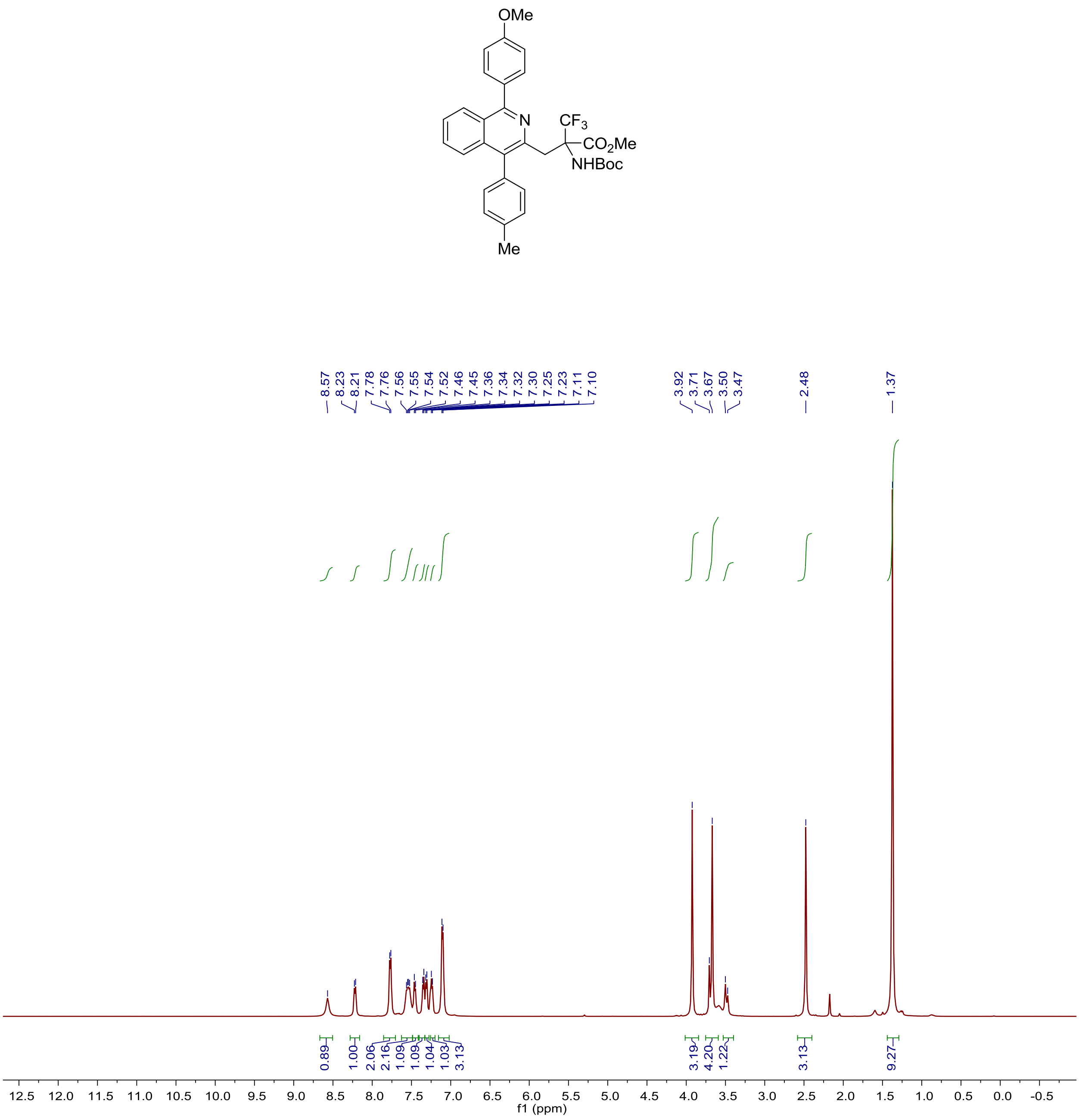

Figure S73. ^{1}H spectrum of **5a** in $CDCl_3$

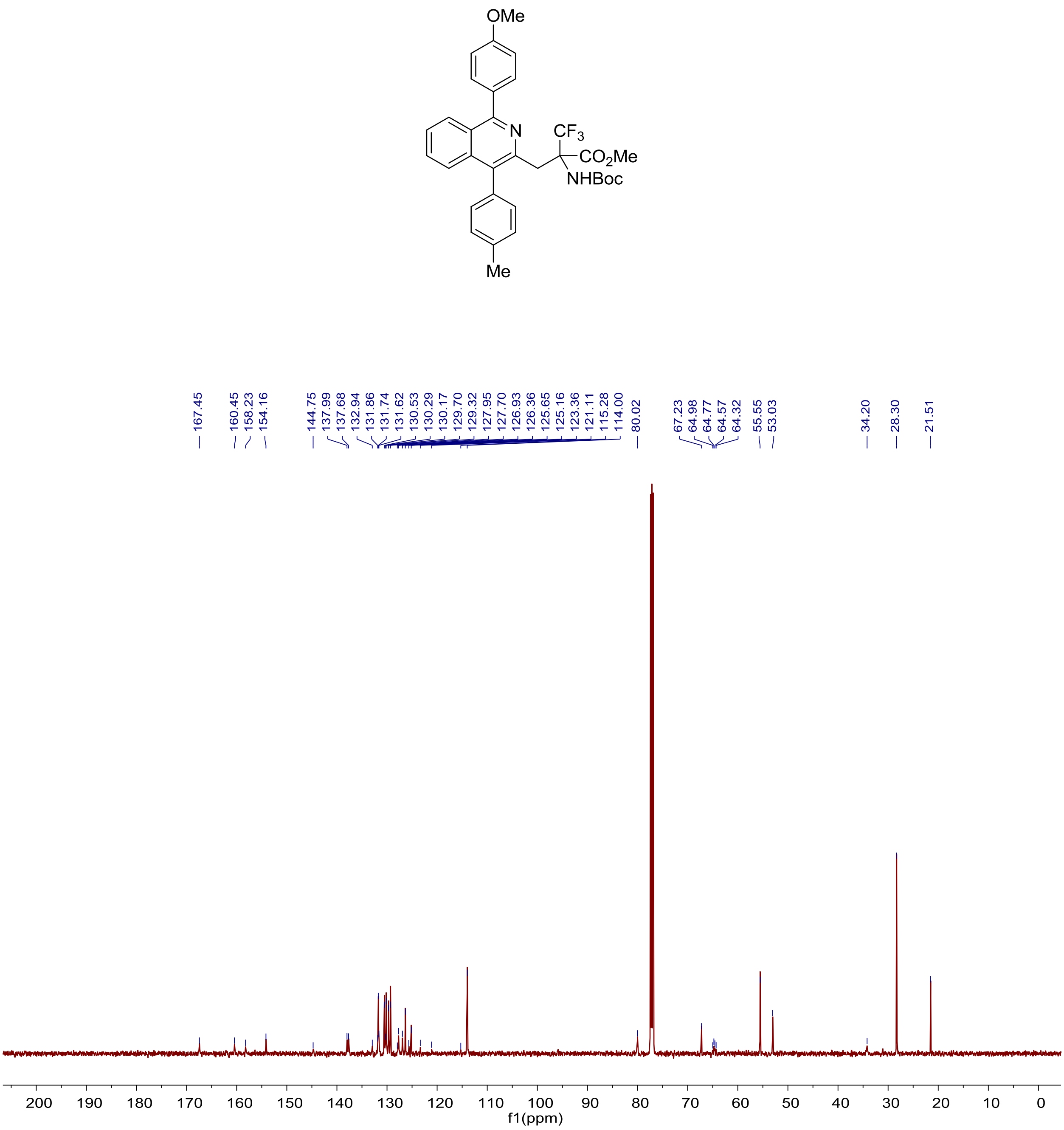

Figure S74. ^{13}C spectrum of **5a** in $CDCl_3$

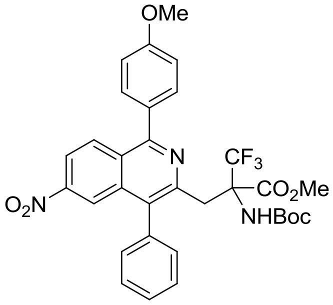

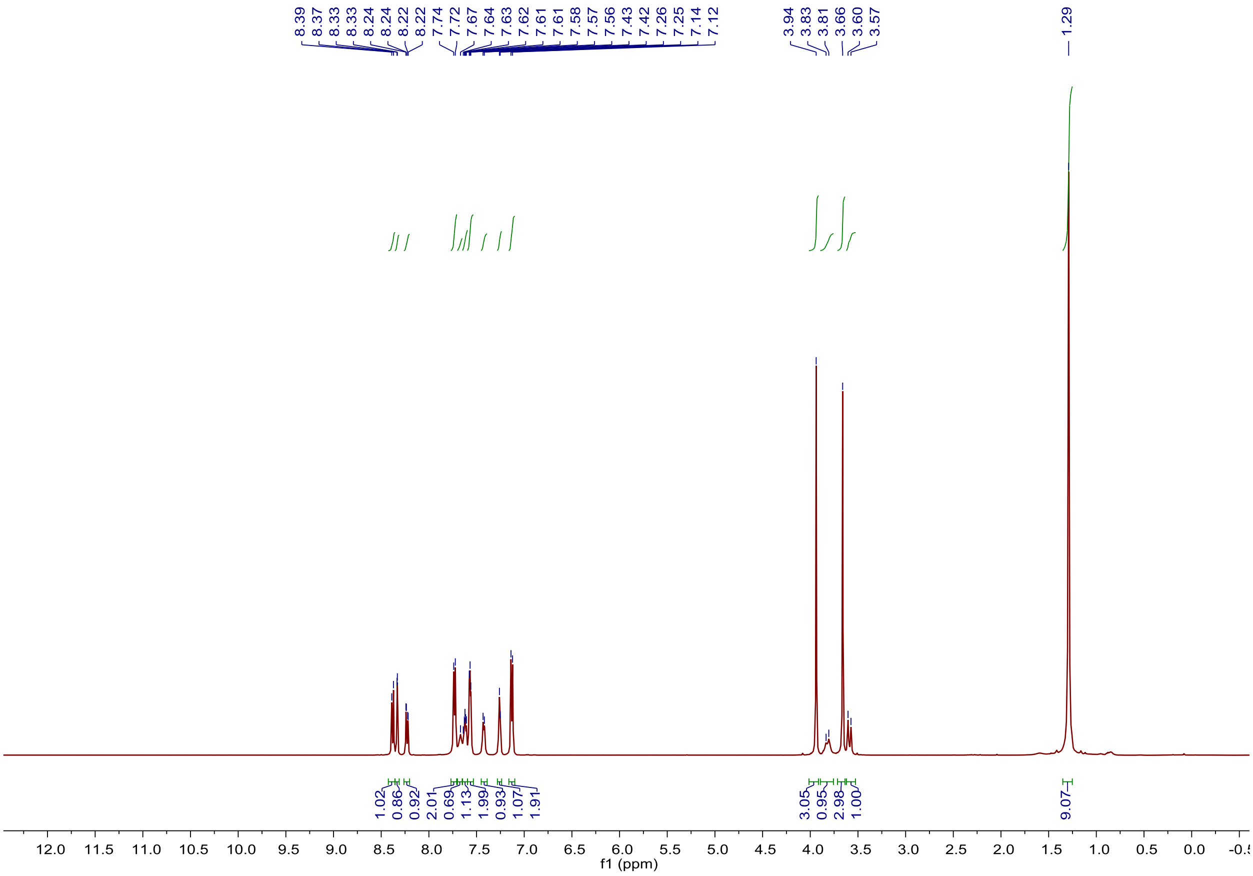

Figure S75. ^{1}H spectrum of **5b** in $CDCl_3$

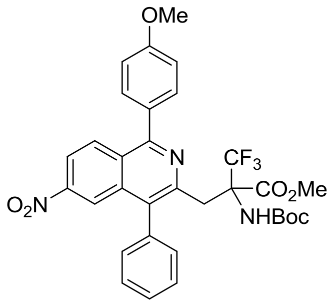

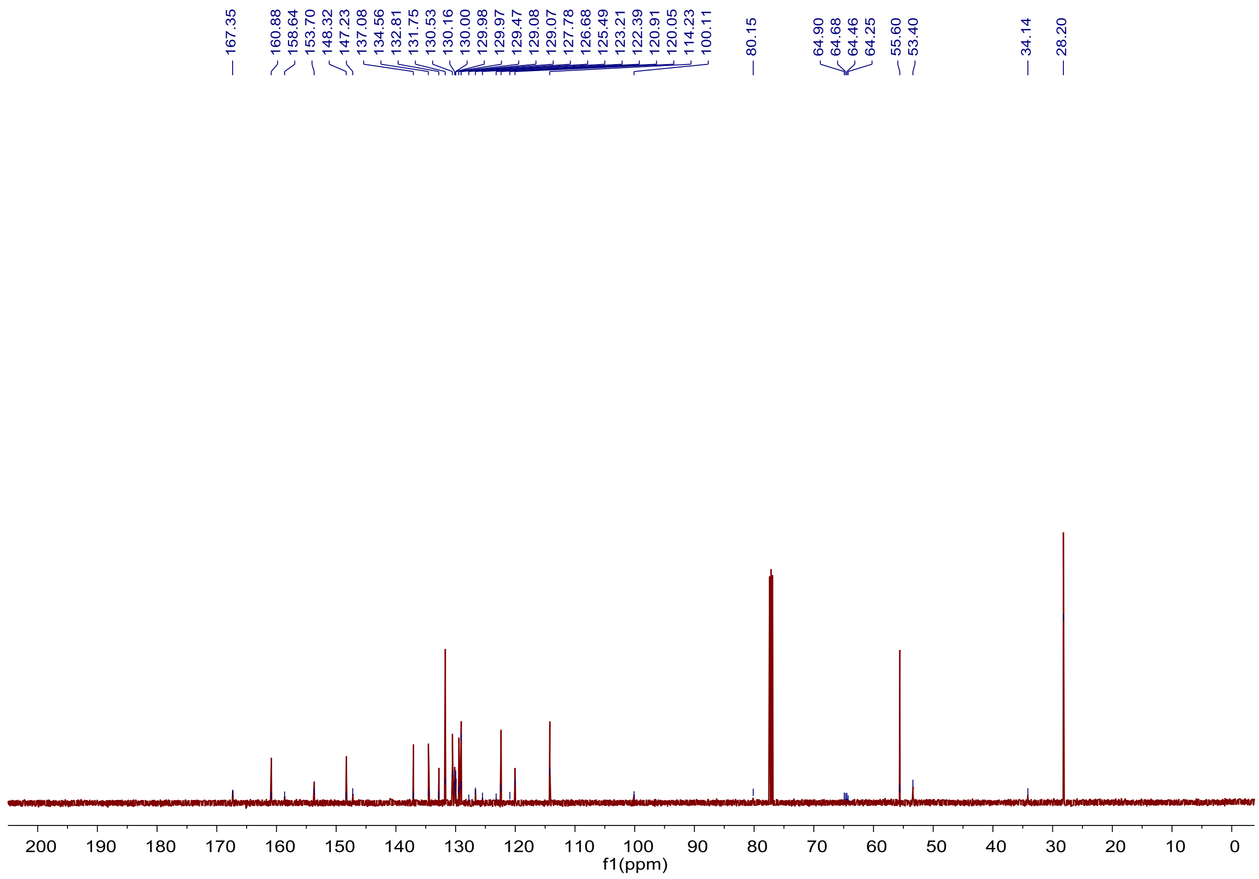

Figure S76. ^{13}C spectrum of **5b** in $CDCl_3$

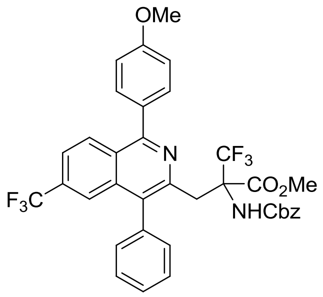

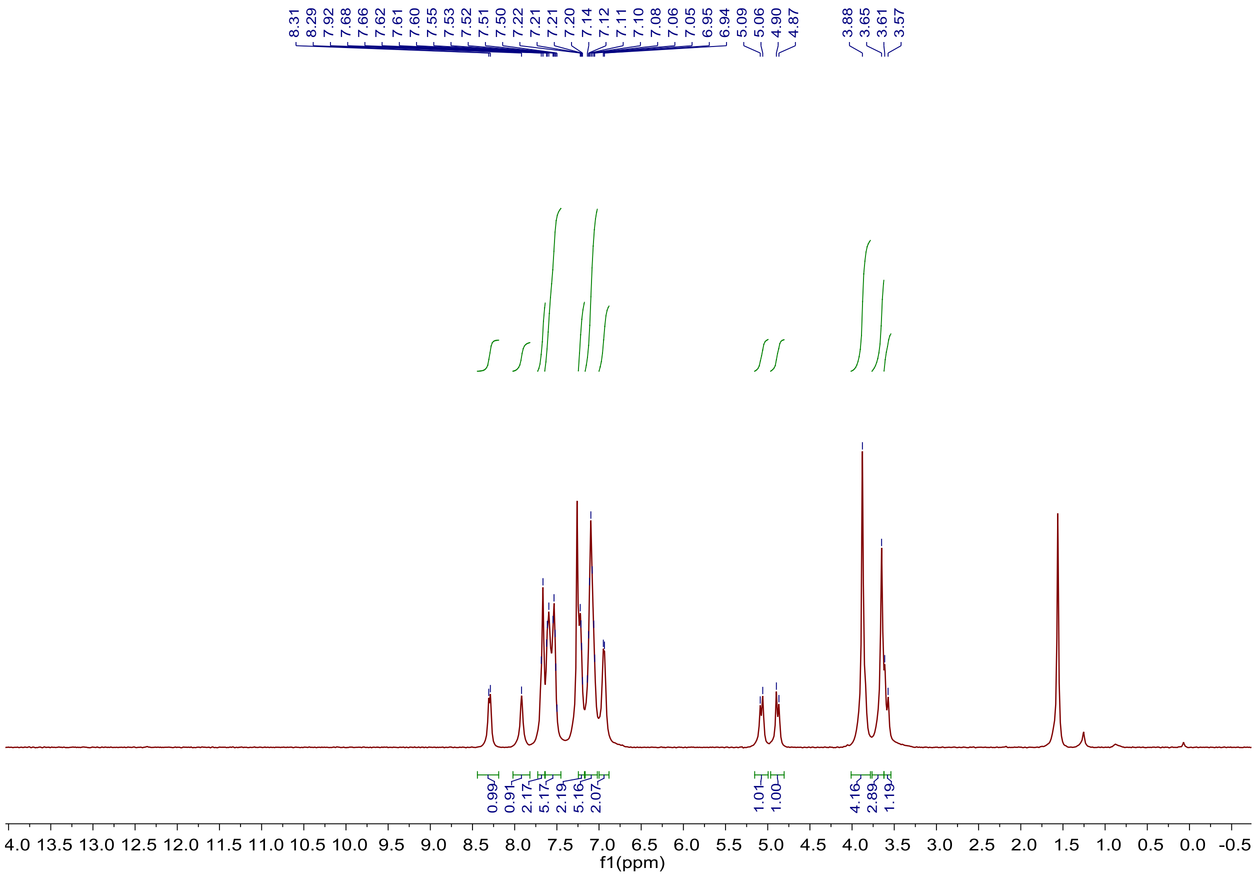

Figure S77. ^{1}H spectrum of **5c** in $CDCl_3$

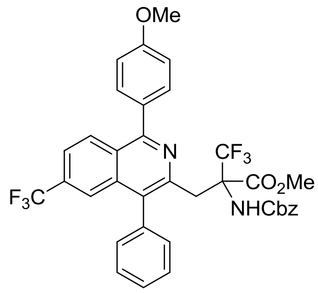

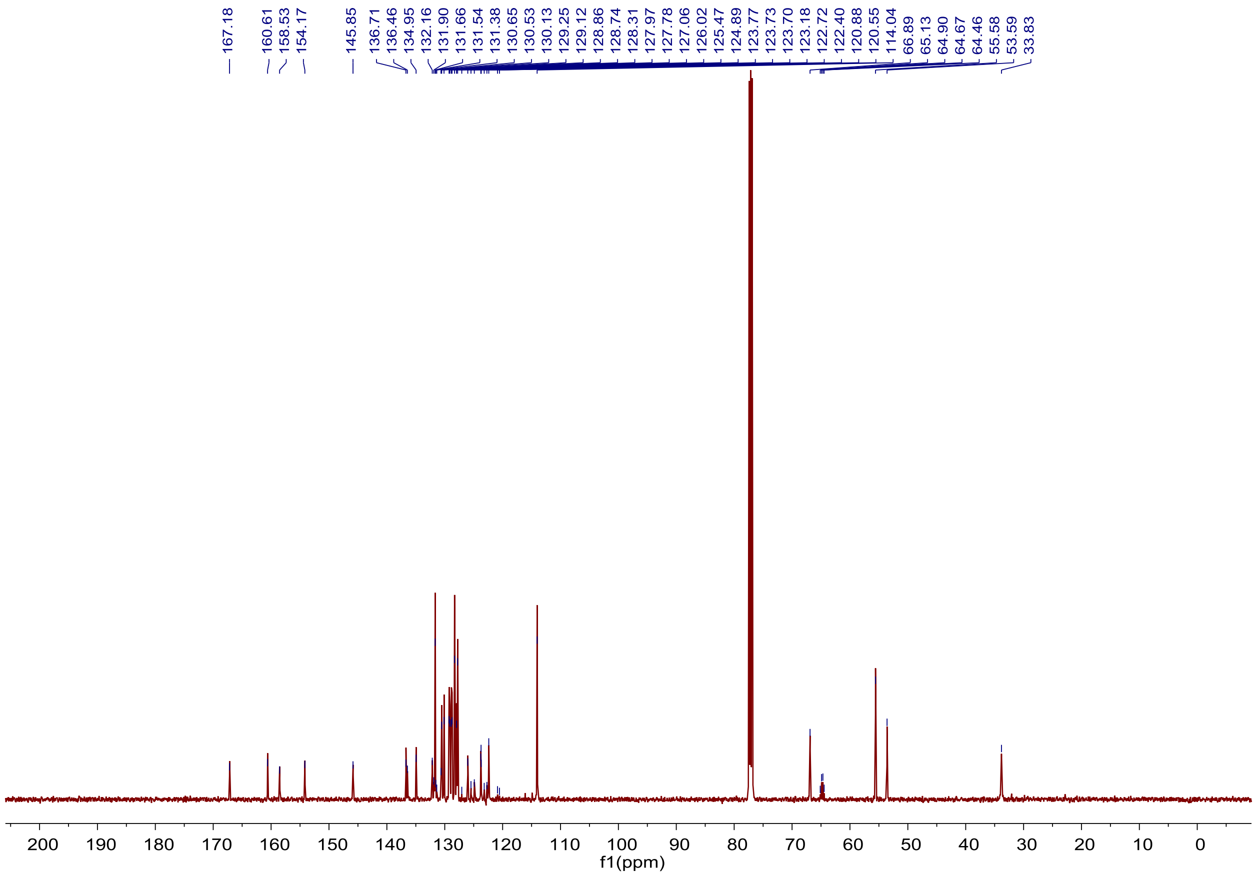

Figure S78. ^{13}C spectrum of **5c** in $CDCl_3$

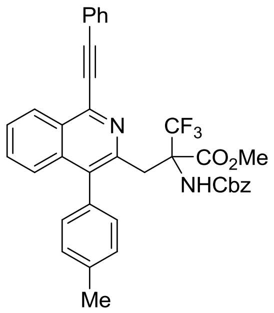

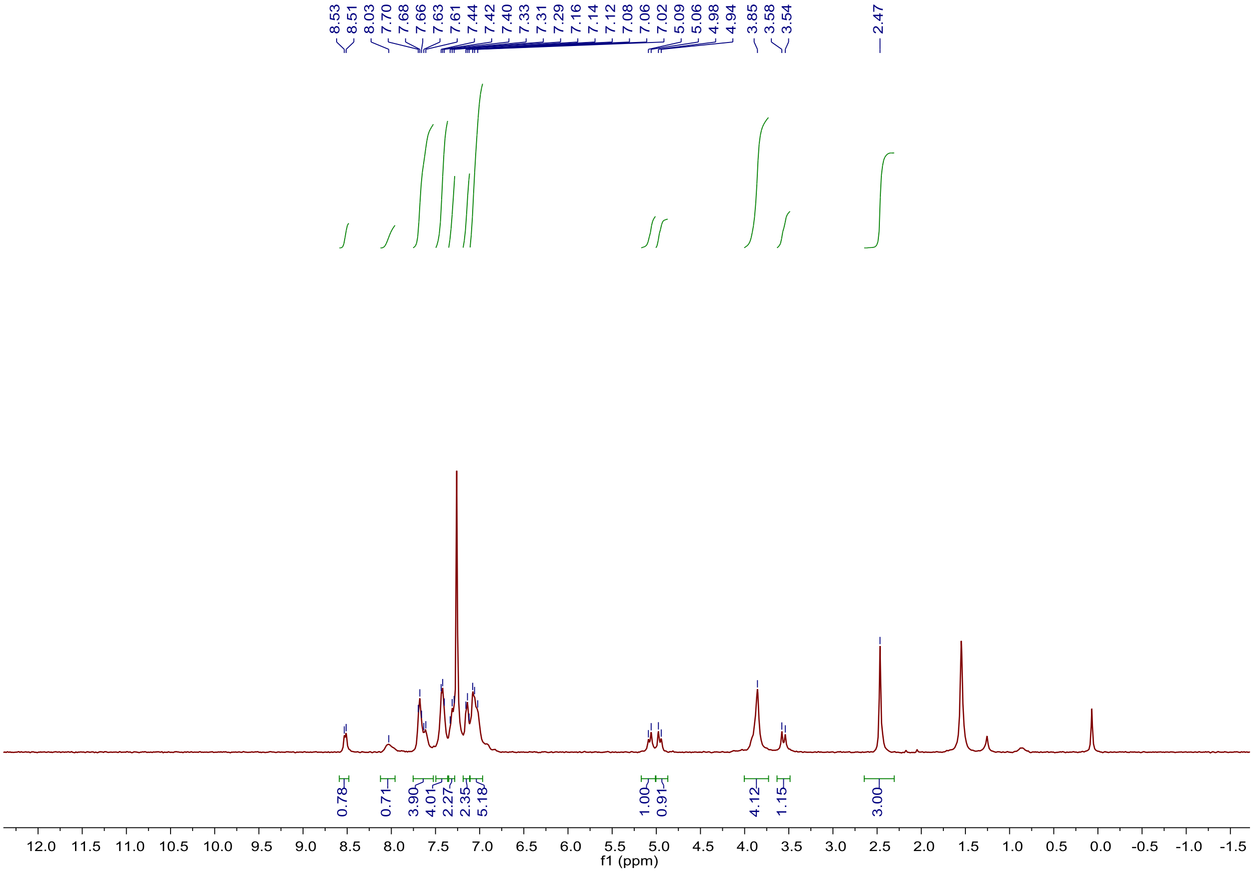

Figure S79. ^{1}H spectrum of **6a** in $CDCl_3$

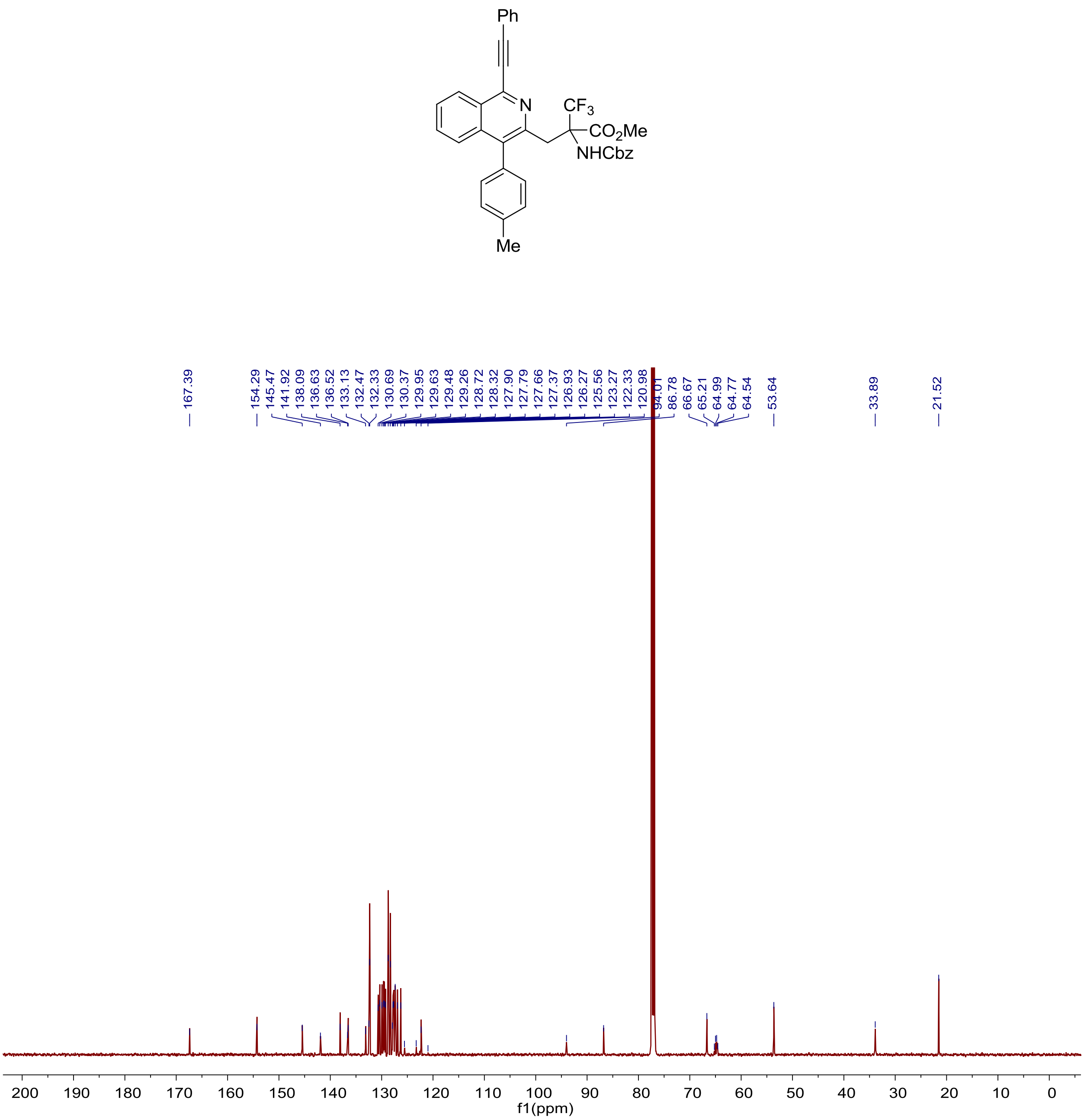

Figure S80. ^{13}C spectrum of **6a** in $CDCl_3$

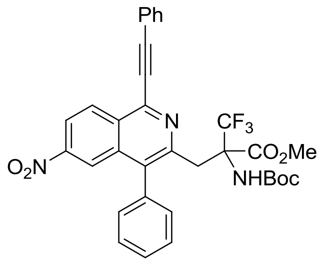

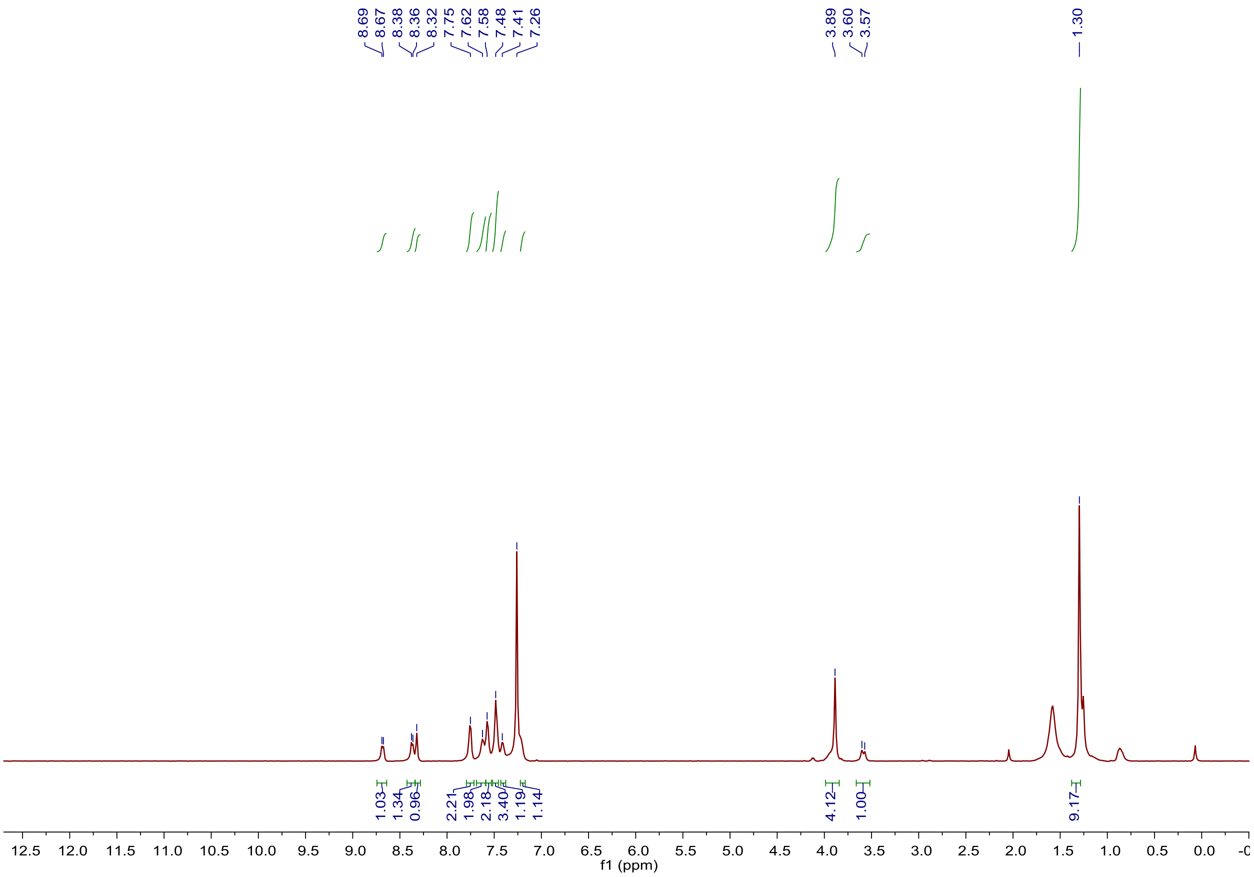

Figure S81. ^{1}H spectrum of **6b** in $CDCl_3$

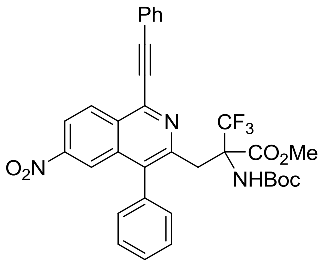

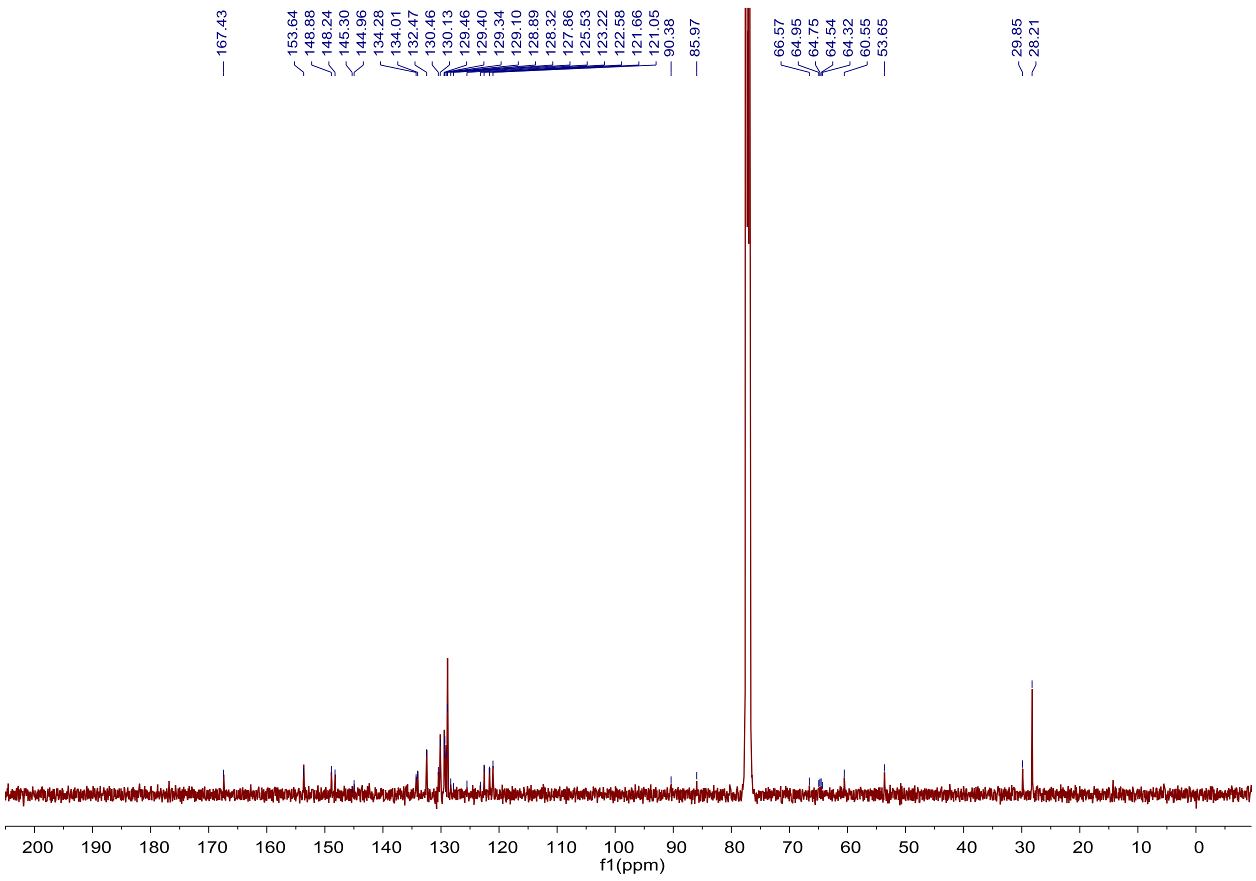

Figure S82. ^{13}C spectrum of **6b** in $CDCl_3$

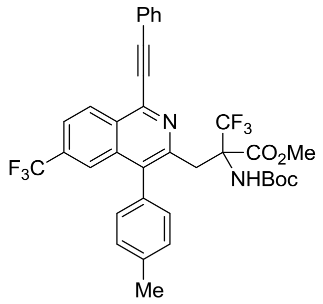

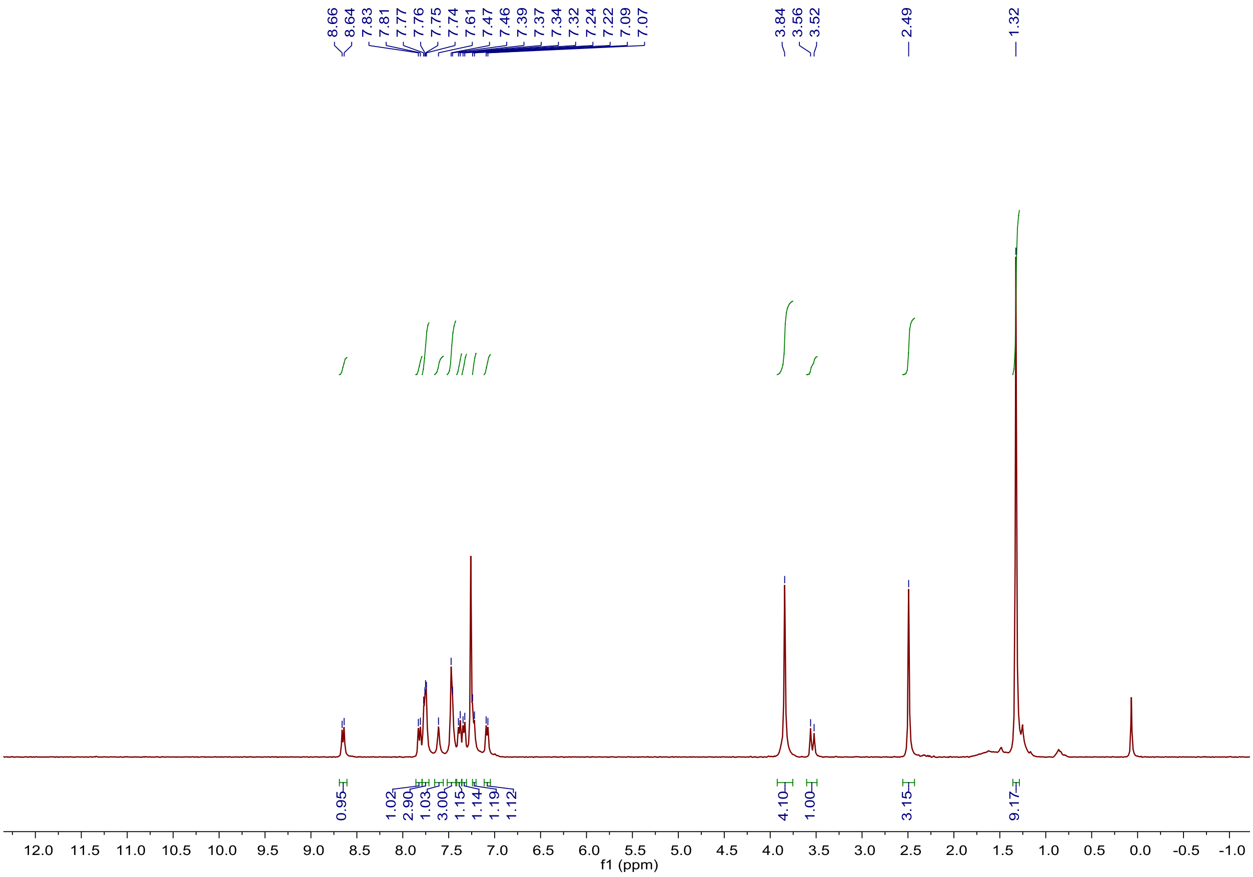

Figure S83. ^{1}H spectrum of **6c** in $CDCl_3$

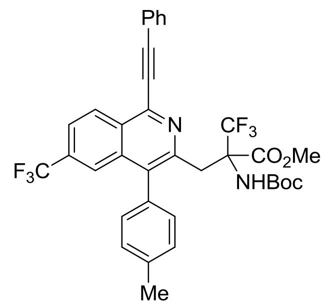

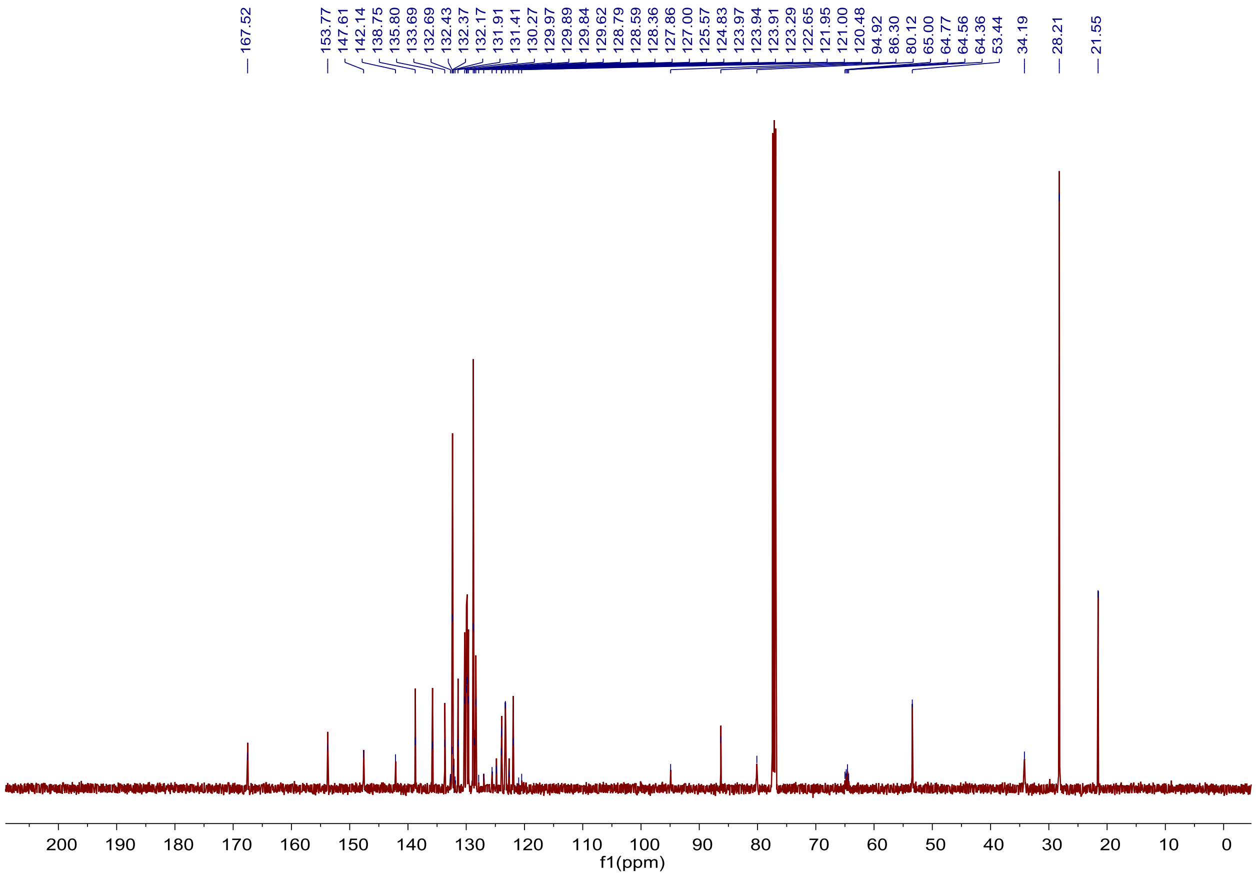

Figure S84. ^{13}C spectrum of **6c** in $CDCl_3$

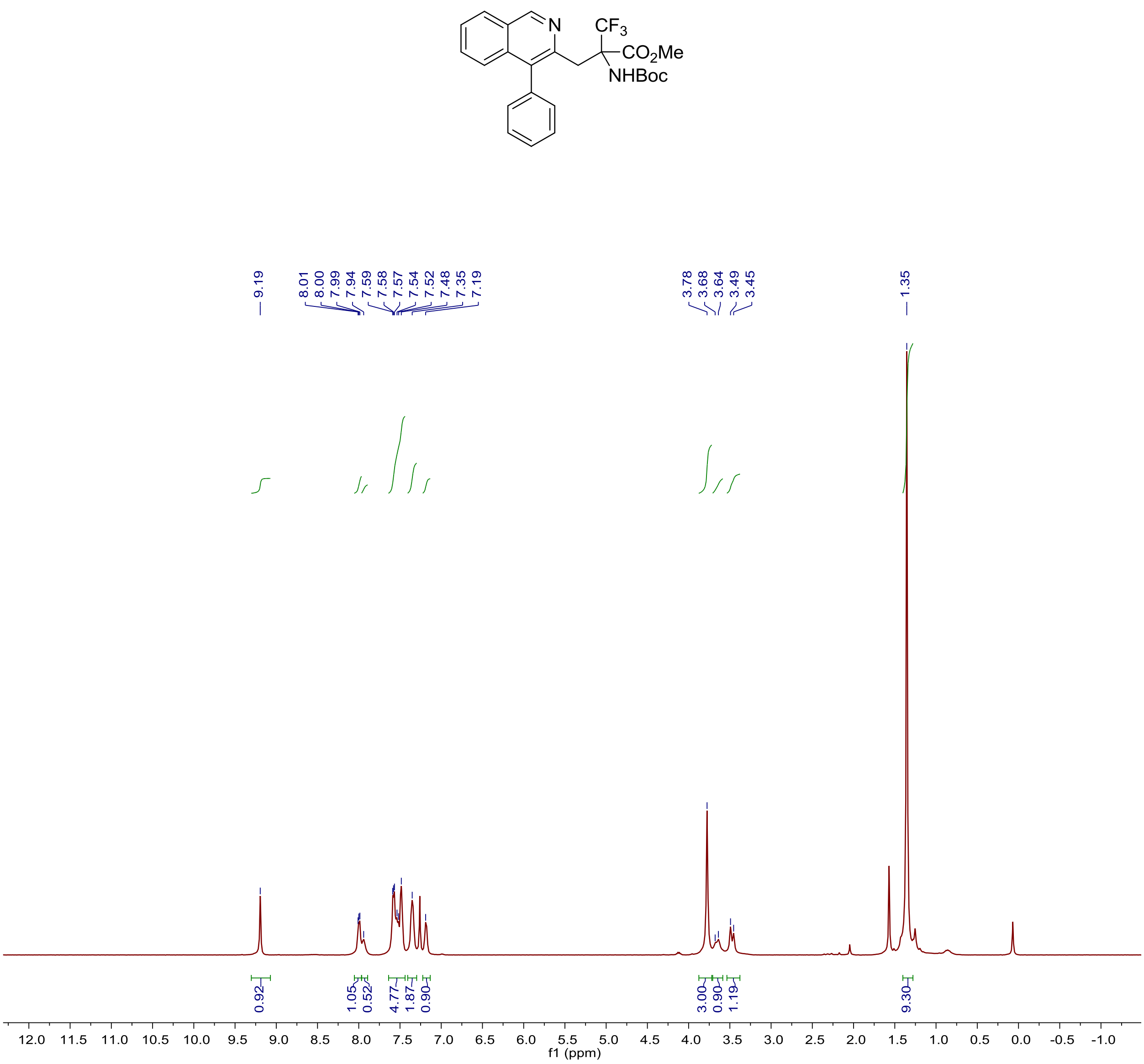

Figure S85. ^{1}H spectrum of **7a** in $CDCl_3$

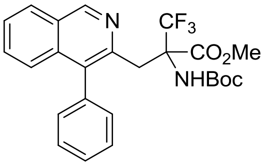

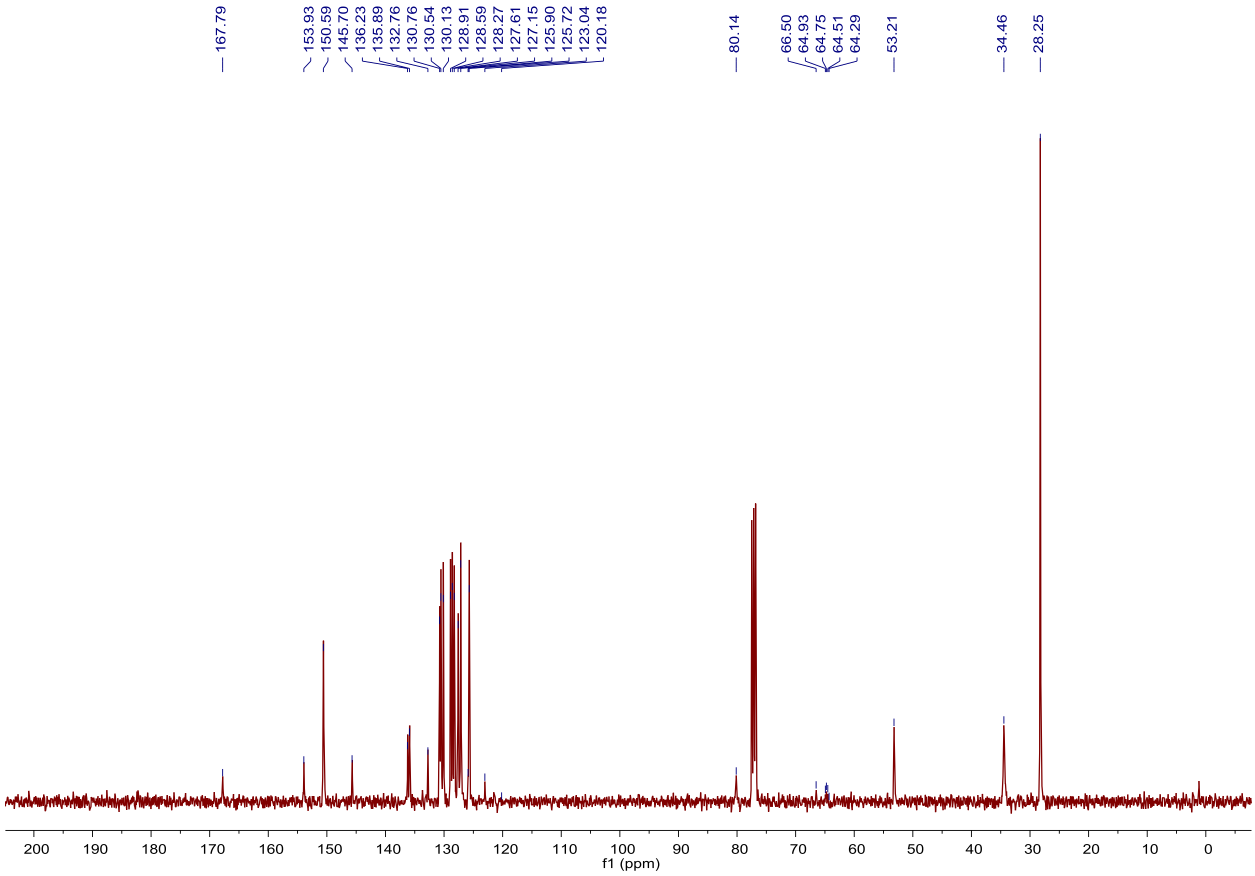

Figure S86. ^{13}C spectrum of **7a** in $CDCl_3$

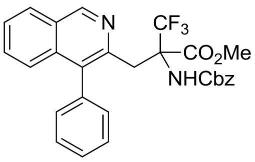

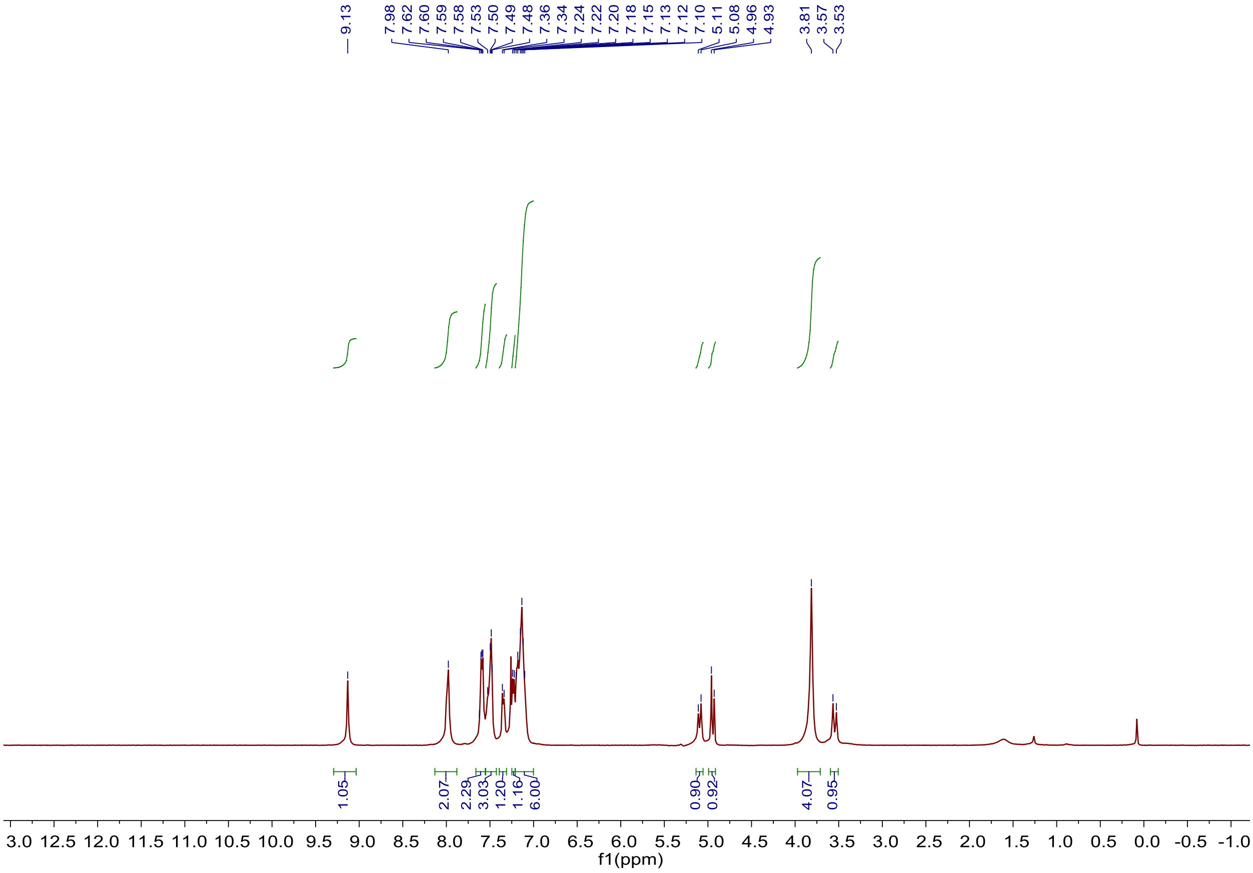

Figure S87. ^{1}H spectrum of **7b** in $CDCl_3$

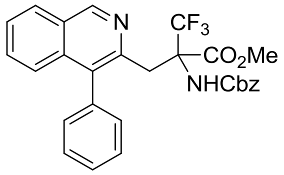

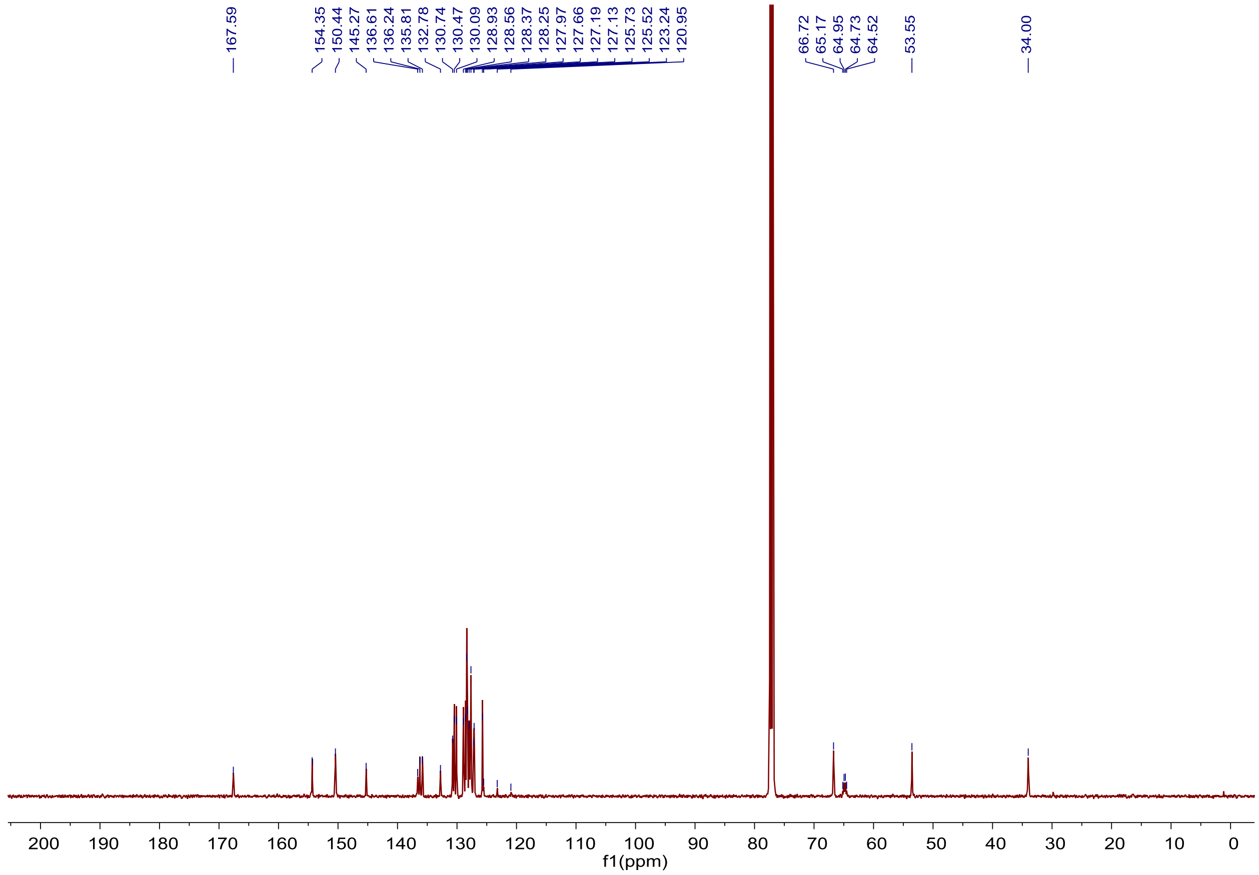

Figure S88. ^{13}C spectrum of **7b** in $CDCl_3$

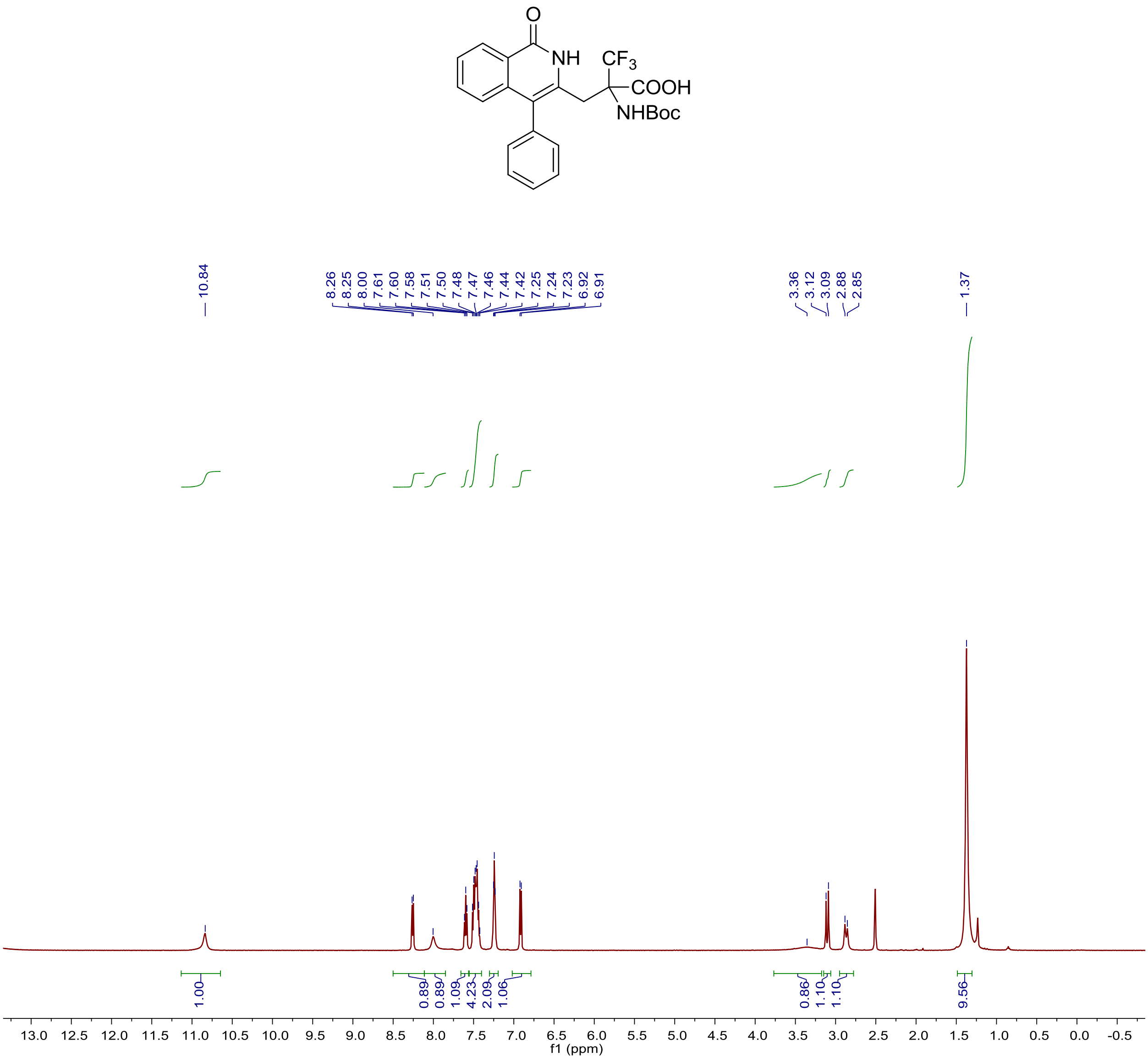

Figure S89. ^{1}H spectrum of **8** in DMSO-d_6

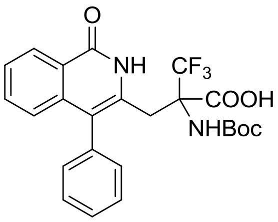

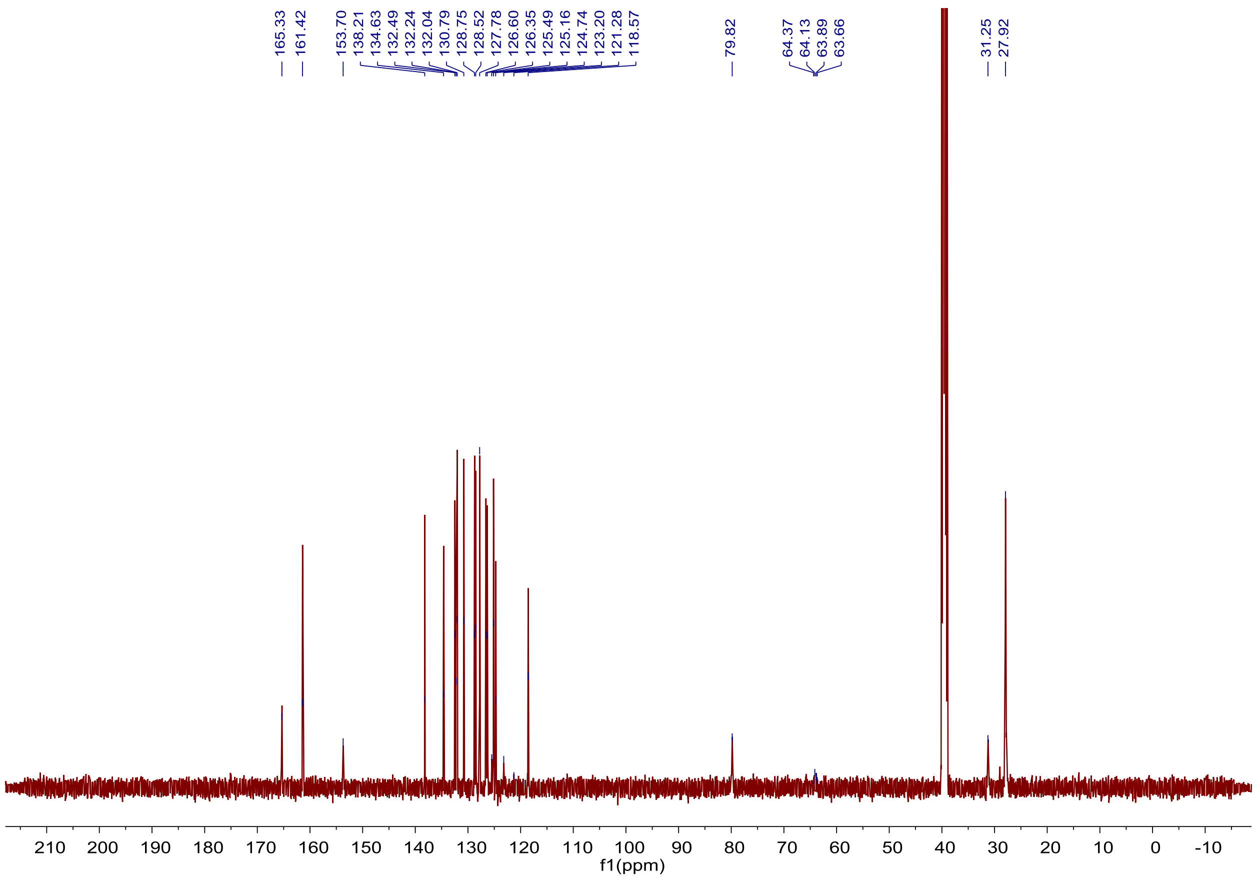

Figure S90. ^{13}C spectrum of **8** in DMSO-d_6

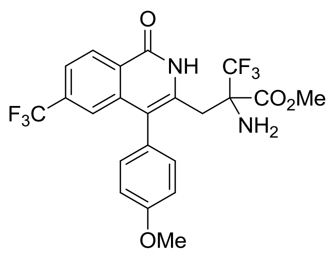

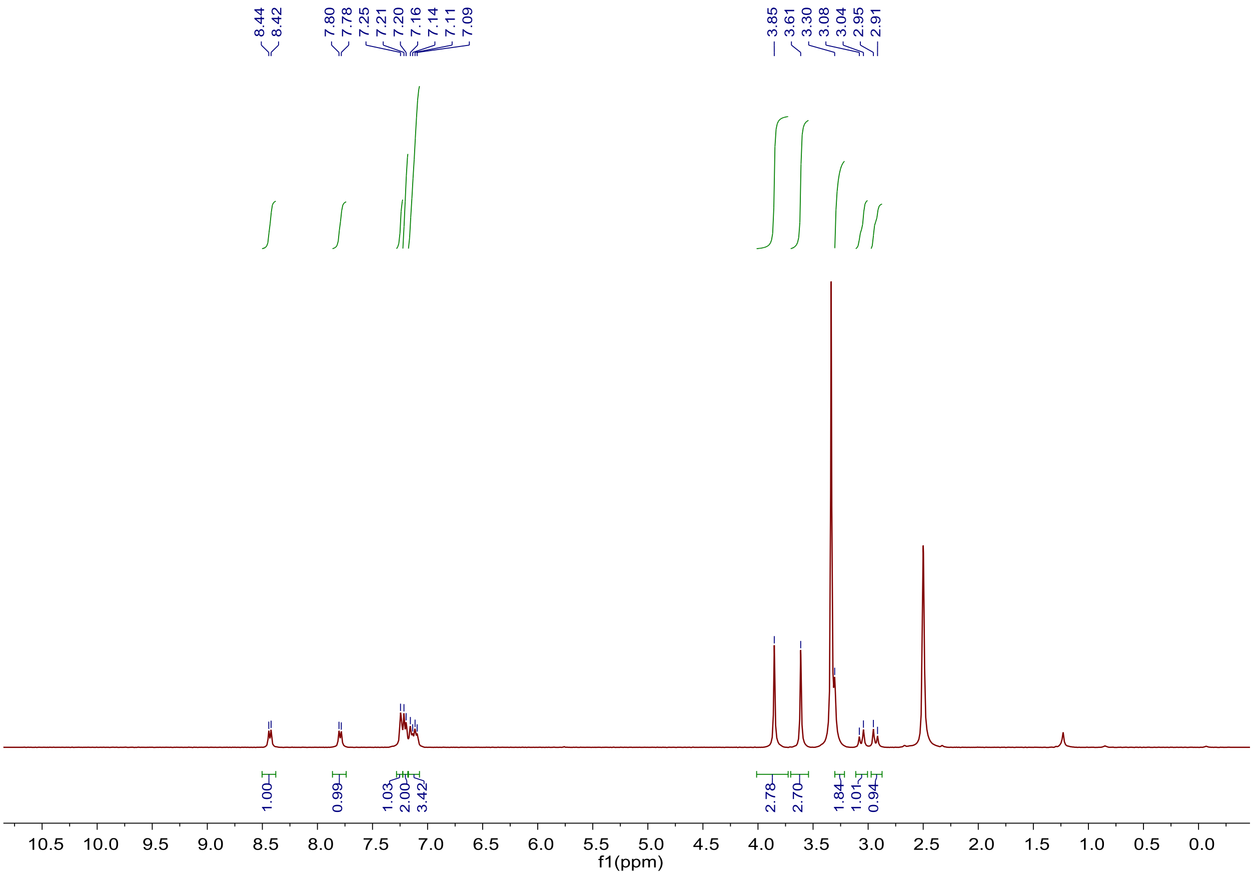

Figure S91. ¹H spectrum of **9** in DMSO-d_6

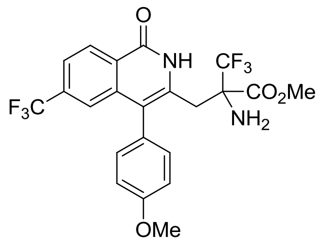

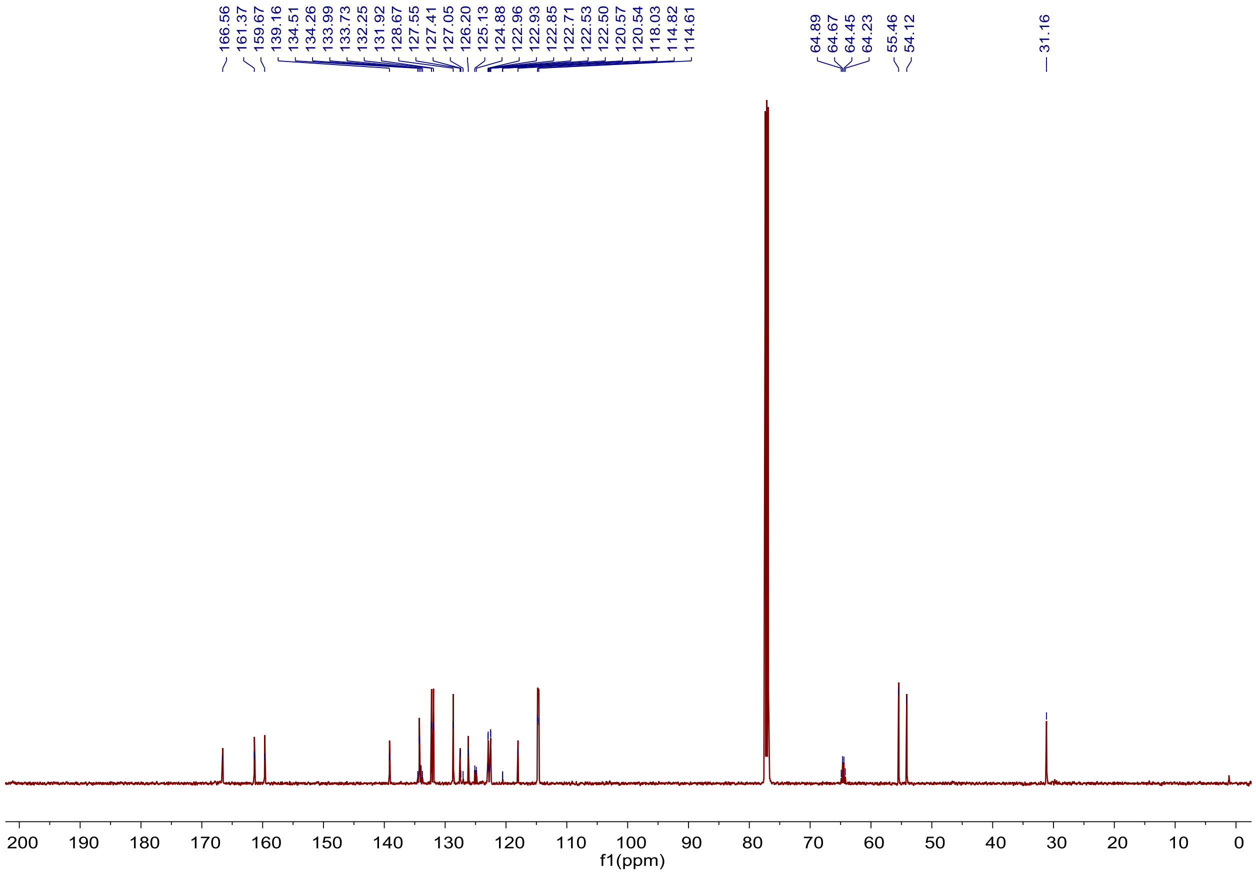

Figure S92. ^{13}C spectrum of **9** in $CDCl_3$

2. Proposed mechanism of [4+2]-annulation

A plausible reaction mechanism of regioselective [4+2]-annulation of aryl hydroxamate with internal acetylene-containing α-CF_3-α-amino esters **2** is depicted in Scheme 1S. After dissociation of the rhodium dimer, ligand exchange of the monomer with the present cesium salts provides a rhodium(III)-carboxylate species. Precomplexation to the directing N-pivaloyloxy amide moiety is followed by C-H activation to form rhodacyle **A**. The regioselective insertion of the alkyne triple bond into the C-Rh bond provides a seven-membered rhodacycle intermediate **B**. The observed selectivity can be probably explained by the coordination of Rh with the donor atom of alkyne according to its inherent polarity. Next, a concerted or stepwise C-N bond forming/N-O bond cleaving event occurs, affording the annulation product **3**, and releasing the Rh(III) catalyst.

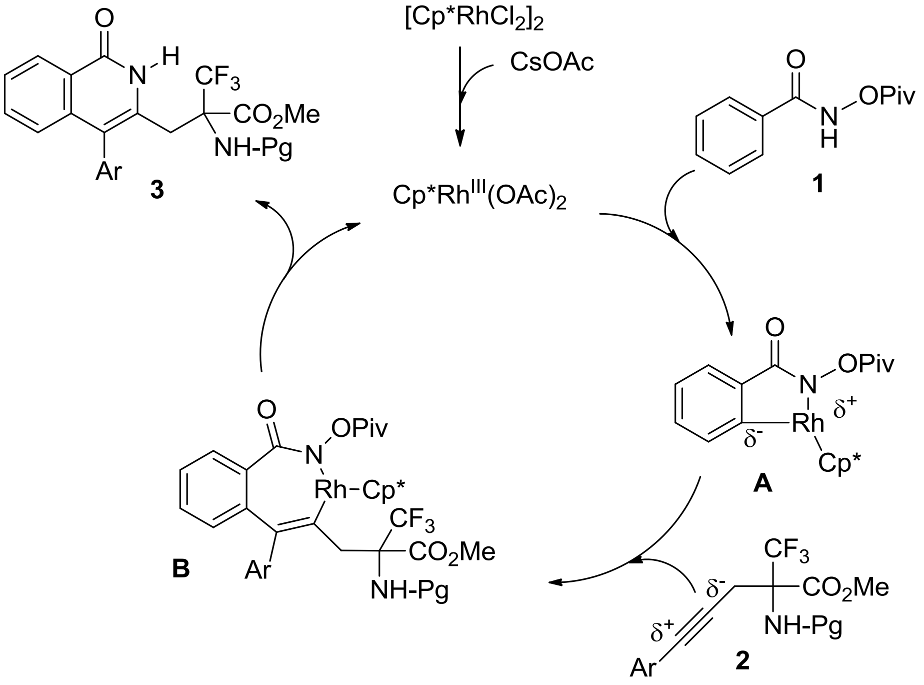

Scheme 1S. Proposed mechanism.

3. X-ray Diffraction Study

Table S1. Crystal data, data collection and structure refinement parameters for **3a**

CCDC	2217085
Formula	$C_{25}H_{25}F_3N_2O_5$
molecular wt	490.47
crystal size (mm)	0.20×0.12×0.02
crystal syst	monoclinic
space group	$C2/c$
Temperature (K)	150(2)
a (Å)	26.096(5)
b (Å)	13.702(4)
c (Å)	13.291(4)
β (deg)	95.018(9)
V (Å^3)	4734(2)
Z	8
d_{calc} (g·cm^{-3})	1.376
linear absorption μ (cm^{-1})	1.11
T_{min}/T_{max}	0.769/0.862
$2\theta_{max}$ (deg)	50
no. of unique reflns (R_{int})	4143 (0.1334)
no. of obs reflns ($I>2\sigma(I)$)	1782
no. of params	329
R_1 (on F for obsd reflns)[a]	0.0693
wR_2 (on F^2 for all reflns)[b]	0.1704
GOOF	0.975
Largest diff. peak/hole (e Å^{-3})	0.263/-0.246

[a] $R_1 = \Sigma||F_o| - |F_c||/\Sigma|F_o|$

[b] $wR_2 = \{\Sigma[w(F_o^2 - F_c^2)^2]/\Sigma w(F_o^2)^2\}^{1/2}$

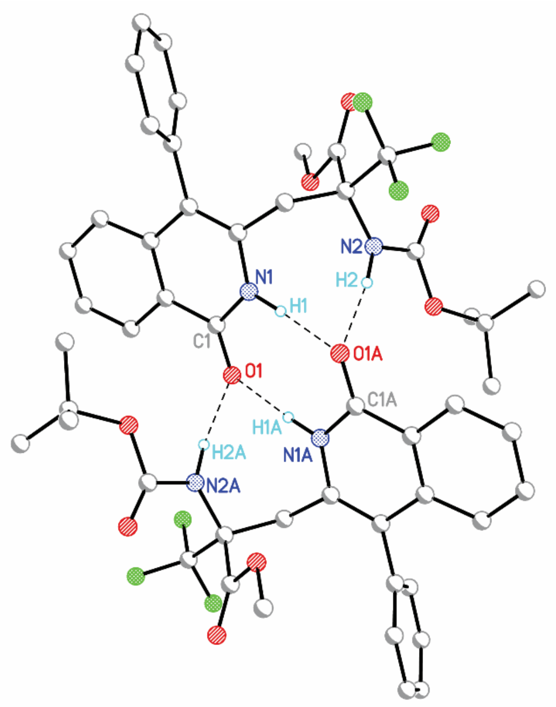

Figure S93. H-bonded dimer in the crystal of **3a**.

D-H...A	d(D-H)	d(H...A)	d(D...A)	<(DHA)
N(1)-H(1)...O(1)#1	0.96(6)	1.91(5)	2.873(5)	175(3)
N(2)-H(2)...O(1)#1	1.00(4)	1.88(4)	2.862(5)	170(4)

Symmetry transformations used to generate equivalent atoms:

#1 -x+1/2,-y+1/2,-z+1
